# Trifluoromethyl Thianthrenium Triflate: A Readily Available Trifluoromethylating Reagent with Formal CF_3_^+^, CF_3_^•^, and CF_3_^–^ Reactivity

**DOI:** 10.1021/jacs.1c02606

**Published:** 2021-05-14

**Authors:** Hao Jia, Andreas P. Häring, Florian Berger, Li Zhang, Tobias Ritter

**Affiliations:** † Max-Planck-Institut für Kohlenforschung, Kaiser-Wilhelm-Platz 1, D-45470 Mülheim an der Ruhr, Germany

## Abstract

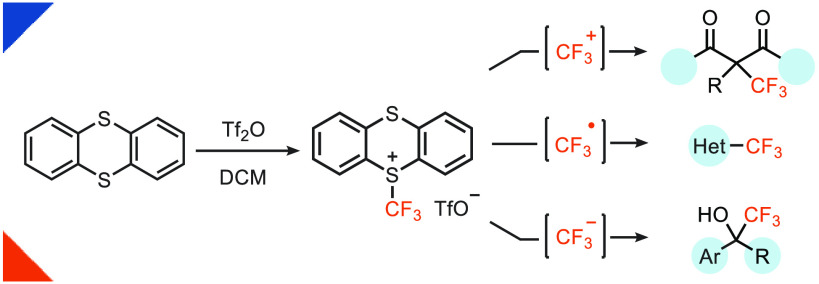

Here we report the synthesis and application of trifluoromethyl thianthrenium triflate (TT-CF_3_^+^OTf^–^) as a novel trifluoromethylating reagent, which is conveniently accessible in a single step from thianthrene and triflic anhydride. We demonstrate the use of TT-CF_3_^+^OTf^–^ in electrophilic, radical, and nucleophilic trifluoromethylation reactions.

More than 65 pharmaceuticals containing the trifluoromethyl substituent have been approved by the FDA so far.^1^ Because no known sources exist in nature, the trifluoromethyl group must be installed into designed molecules by humans. Depending on the desired chemical transformation, different reagents for electrophilic,^2−4^ radical,^5^ or nucleophilic^6^ trifluoromethylation are employed. Given the broad interest in trifluoromethylation chemistry, we introduce here the new reagent trifluoromethyl thianthrenium triflate (**1**, TT-CF_3_^+^OTf^–^). In contrast to other sulfonium-based trifluoromethylating reagents such as the Umemoto reagent,^7^ TT-CF_3_^+^OTf^–^ is readily accessible in a single step from inexpensive starting materials. It shows undiminished reactivity after storage for one year in ambient atmosphere. The new trifluoromethylating reagent engages in electrophilic, radical, and nucleophilic trifluoromethylation. Because of its simple preparation, handling, high reactivity, and broad tolerance of functional groups present in complex molecules, as well as its divergent reactivity, we anticipate that TT-CF_3_^+^OTf^–^ will find widespread utility in future reaction chemistry development.

Syntheses of simple trifluoromethyl-substituted molecules often employ harsh reagents, such as HF, F_2_, or SF_4_, that typically install the fluorine atoms on a (functionalized) methyl group already present in the molecule.^8^ For the functionalization of more complex small molecules, the use of CF_3_ reagents of appropriate reactivity is more common and, depending on the reaction, can be accomplished by nucleophilic, electrophilic, or radical CF_3_ sources. The CF_3_ radical can be produced from diverse reagents, such as TMS-CF_3_,^9,10^ trifluoroiodomethane,^11^ zinc triflinate,^12^ and sodium triflinate,^13^ via single-electron chemistry. Nucleophilic trifluoromethylating reagents function by transfer of the unstable CF_3_ anion.^14−16^ Common CF_3_ anion precursors are borazine CF_3_^–^,^17^ PhSO_2_CF_3_,^18^ and TMS-CF_3_.^19−21^ These species reversibly^16,17,22^ release CF_3_^–^, with some precursors requiring anionic activation by addition of alkoxide or fluoride. Electrophilic trifluoromethylating reagents can often be used as both CF_3_^+^ and CF_3_ radical sources.^23^ Most electrophilic trifluoromethylating reagents are based on hypervalent iodine or chalcogenide reagents.^2−4^ The Togni reagents I and II^24^ and Umemoto reagent^25,26^ (Scheme 1) are the most prominent representatives of these classes, now commercially available, and of large synthetic value.^27^ The main difference of TT-CF_3_^+^OTf^–^ (**1**) when compared to most other sulfonium-based reagents is its simple one-step synthesis protocol; the practical synthesis for the classical Umemoto reagent requires nine steps, although other derivatives such as 2,8-difluoro- and 2,3,7,8-tetrafluoro-*S*-(trifluoromethyl) dibenzothiophenium salts, can be prepared by one-pot methods.^28,29^ The fundamental difference from the Togni reagents is the higher reduction potential of TT-CF_3_^+^OTf^–^, a consequence of the positive charge, which it shares with the Umemoto reagent, which can result in complementary reactivity in single-electron transfer reactions when compared to the λ^3^-iodane compounds.^30^

**Scheme 1:**
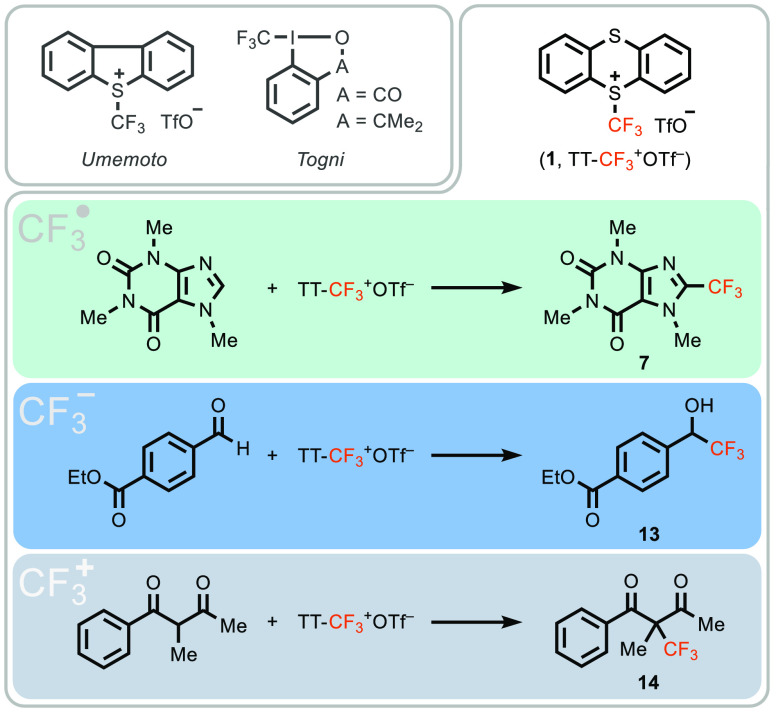
Common Electrophilic CF_3_ Reagents, Trifluoromethyl Thianthrenium Triflate (**1**, TT-CF_3_^+^OTf^–^), and Typical Applications of TT-CF_3_^+^OTf^–^

Aryl thianthrenium salts can be employed as aryl radical precursors in photoredox reactions and perform well as electrophiles in cross-coupling reactions.^31−46^ A lot of the unique reactivity of the thianthrene-based chemistry is a consequence of the stability of the persistent thianthrene radical cation, which may be the reason for yet another unusual and useful reactivity finding: We were surprised and then intrigued when we found that simple mixing of thianthrene with triflic anhydride in DCM under ambient conditions yields trifluoromethylated thianthrene (**1**, TT-CF_3_^+^OTf^–^). The reaction does not require an inert atmosphere and can be scaled up, and the product can be purified by washing with diethyl ether to deliver an easy to handle, free-flowing off-white powder that can be stored under ambient conditions in the absence of light without significant decomposition for at least a year (Scheme 2A). An analogous reaction of triflic anhydride with dibenzothiophene cannot be used for a practical synthesis of the Umemoto reagent. We rationalize the unanticipated reaction for the formation of TT-CF_3_^+^OTf^–^ from triflic anhydride by a mechanism that can proceed with a low barrier due to the stability of the thianthrene radical cation (Scheme 2A): Nucleophilic attack of thianthrene to triflic anhydride results in S–S bond formation,^47^ which can cleave homolytically to release two radicals due to its low bond dissociation energy, computed at 11.1 kcal/mol. Sulfur dioxide expulsion results in formation of the trifluoromethyl radical, which can recombine with the persistent thianthrenium radical cation to form TT-CF_3_^+^OTf^–^. Control experiments support the presence of the thianthrenium radical cation (Scheme 2B) and the presence of the trifluoromethylsulfinyl radical and trifluoromethyl radical before the reagent is formed (Scheme 2C). Cyclic voltammetry measurements for thianthrene (TT, *E*_ox_ = 0.86 V vs Cp_2_Fe) and triflic anhydride indicate that single-electron oxidation of thianthrene by triflic anhydride is unlikely (Scheme 2D).

**Scheme 2:**
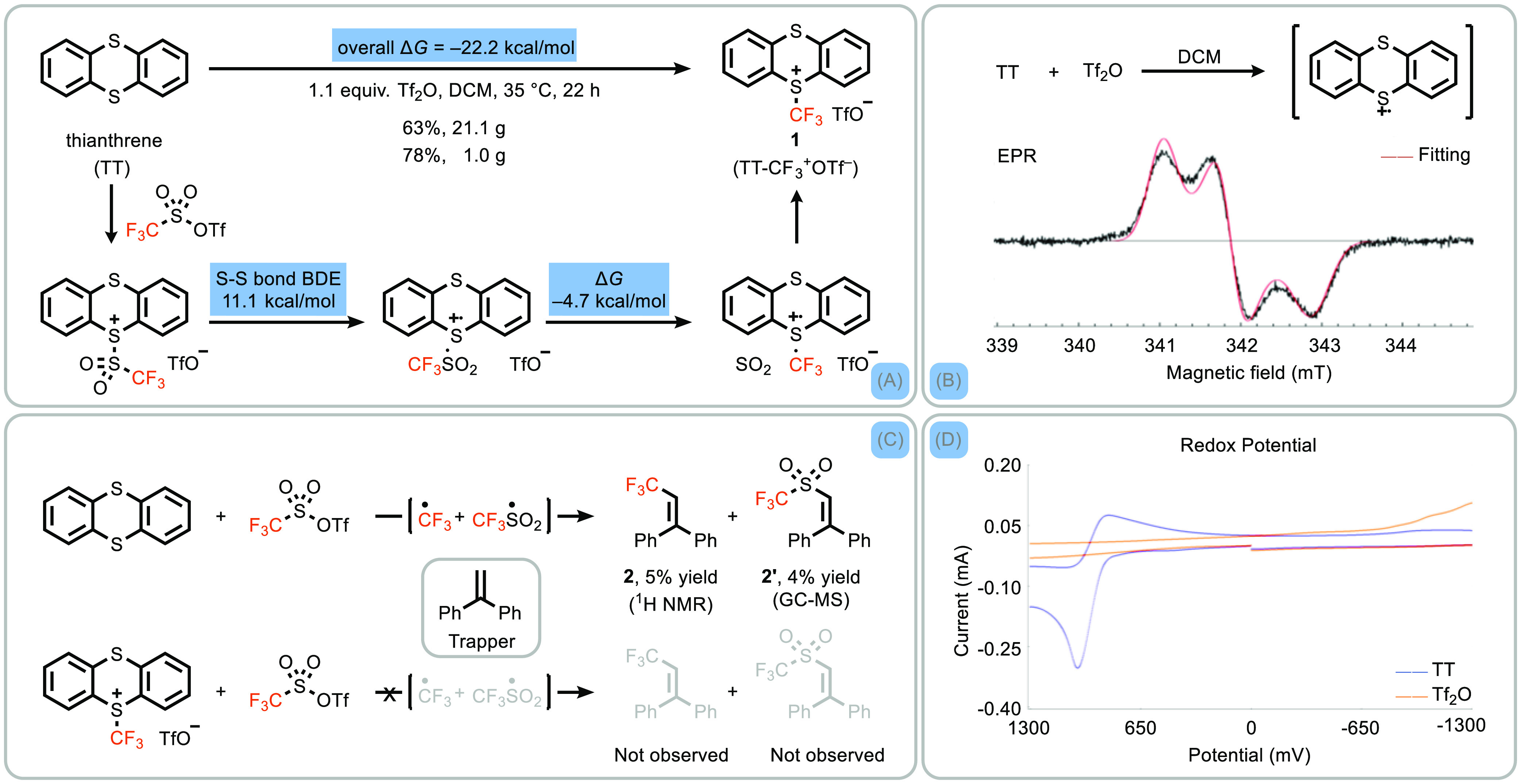
Synthesis of TT-CF_3_^+^OTf^–^ and Reactivity Studies (A) Synthesis of TT-CF_3_^+^OTf^–^ including a proposed mechanism for its formation. Δ*H* and Δ*G* data were derived from DFT studies. The bond dissociation energy (BDE) of the S–S bond is represented by Δ*H*. (B) Electron paramagnetic resonance spectrum under the reaction conditions (magnetic flux density). (C) CF_3_ radical trapping experiments and (D) cyclic voltammogram of thianthrene and triflic anhydride in CH_3_CN (*E* [mV] vs Cp_2_Fe).

After recrystallization, translucent prismatic TT-CF_3_^+^OTf^–^ crystals could be obtained and analyzed by single-crystal X-ray crystallography (Figure 1). The S-CF_3_ bond distance of TT-CF_3_^+^OTf^–^ (1.89 Å) is similar to the analogous distance in the Umemoto reagent (1.87 Å). According to differential scanning calorimetry/thermogravimetric analysis (DSC-TGA), crystalline TT-CF_3_^+^OTf^–^ starts to decompose at 142 °C under melting, which is in the same range as Togni’s and Umemoto’s reagents (Table 1). The Togni reagents decompose exothermically,^48−50^ while decomposition of the Umemoto reagent and TT-CF_3_^+^OTf^–^ (**1**) releases less energy upon decomposition, which attests to the desirable safety profile for **1**. For a detailed analysis of the various transitions upon heating, see the DSC-TGA analysis in the Supporting Information (Figures S15–S17). In addition, we further characterized the electrochemical properties of TT-CF_3_^+^OTf^–^. Cyclic voltammetry of TT-CF_3_^+^OTf^–^ shows an irreversible reduction wave with a cathodic peak potential of −0.99 V vs Cp_2_Fe. Therefore, TT-CF_3_^+^OTf^–^ is a stronger outer-sphere oxidant than both Togni reagents,^51^ which is anticipated due to its cationic charge.

**Table 1:**
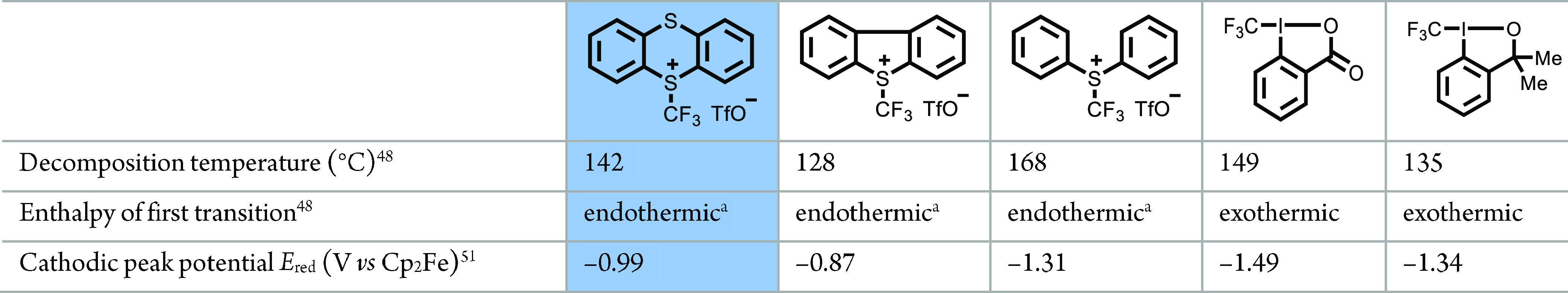
Thermochemical and Electrochemical Properties of Common CF_3_ Reagents

a For detailed DSC-TGA analysis, see Supporting Information Figures S15–S17.

**Figure 1:**
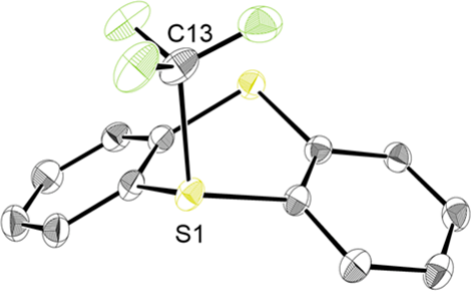
Crystal structure of TT-CF_3_^+^. Thermal ellipsoids are drawn at the 50% probability level. Hydrogen atoms and counterion have been omitted for clarity. S1–C13 bond distance average: 1.89 Å. Two similar cations with slightly different parameters are found in the unit cell; for details, see the SI.

We have established that TT-CF_3_^+^OTf^–^ is a competent electrophilic trifluoromethylating reagent. For example, copper(0)-mediated cross-coupling with arylboronic acids proceeds well (Scheme 4A), as does electrophilic trifluoromethylation of deprotonated 1,3-dicarbonyl compounds (Scheme 4D). As expected, TT-CF_3_^+^OTf^–^ can also perform in radical trifluoromethylation as shown in Scheme 4B^52,53^ and E. Unlike other electrophilic trifluoromethylation reagents, we have shown that TT-CF_3_^+^OTf^–^ is useful for nucleophilic trifluoromethylation^54^ (Scheme 4C) through umpolung with silanolate as nucleophile. We explain this reactivity by formal expulsion of a trifluoromethyl anion with thianthrene oxide as a side product upon addition of silanolate to the reagent to form a metastable sulfurane (Scheme 3A).

**Scheme 3:**
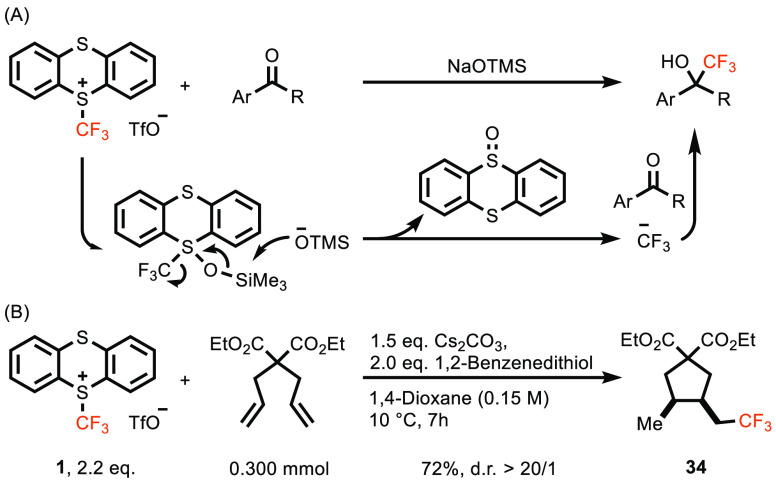
(A) Proposed Mechanism for Nucleophilic Addition and (B) Radical Clock Control Experiment The d.r. value was determined by ^1^H NMR spectroscopy.

**Scheme 4:**
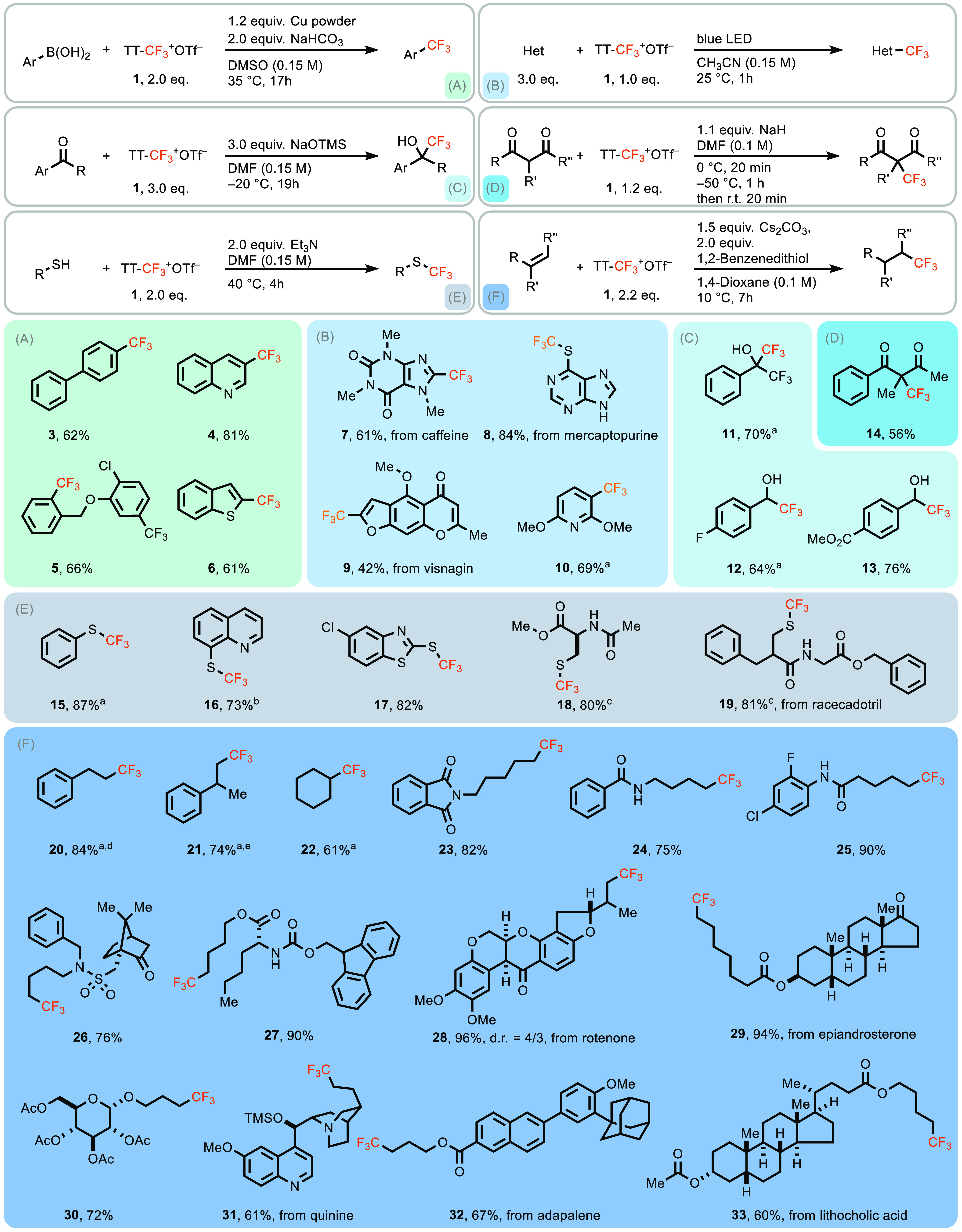
(A) Cross-Coupling Reaction, (B) Radical Trifluoromethylation, (C) Nucleophilic Trifluoromethylation, (D) Electrophilic Trifluoromethylation, (E) Trifluoromethylation of Thiols, and (F) Hydrotrifluoromethylation of Olefins with TT-CF_3_^+^OTf^–^ Yields of volatile trifluoromethylation products are reported based on ^19^F NMR integration of reaction mixtures with internal standard Ph–CF_3_. 3.0 equiv of Et_3_N was used, on account of starting hydrochloride material. Aliphatic thiol (0.30 mmol, 1.0 equiv), TT-CF_3_^+^OTf^–^ (1.2 equiv), 1,1,3,3-tetramethylguanidine (TMG, 1.2 equiv), CH_3_CN (2.0 mL, *c* = 0.15 M), rt, 6 h. Styrene (1.00 mmol, 1.0 equiv), TT-CF_3_^+^OTf^–^ (1.5 equiv), Cs_2_CO_3_ (2.0 equiv), 1,2-benzenedithiol (2.0 equiv), isobutanol (10.0 mL, *c* = 0.10 M), 0 °C, 30 min. 2-Phenylpropene (0.2 mmol, 1.0 equiv), TT-CF_3_^+^OTf^–^ (1.5 equiv), Cs_2_CO_3_ (2.0 equiv), 1,2-benzenedithiol (2.0 equiv), isobutanol (1.0 mL, *c* = 0.10 M), 0 °C, 30 min. The reaction conditions are marked in the reaction equation. All nonvolatile trifluoromethylation products were isolated and characterized as analytically pure samples.

To evaluate TT-CF_3_^+^OTf^–^ in a transformation that has not already been disclosed with other trifluoromethylating reagents, we explored the hydrotrifluoromethylation of olefins in the absence of catalyst. Hydrotrifluoromethylation with conventional trifluoromethylating reagents is known but only with catalysts, for example photoredox catalysts.^55−57^ We show here that hydrotrifluoromethylation of olefins can be accomplished by simply adding olefin and TT-CF_3_^+^OTf^–^ in the presence of 1,2-benzenedithiol as hydrogen atom donor (Scheme 4F). The substrate scope of the hydrotrifluoromethylation and the functional group tolerance of the new reagent are large: esters, ethers, nitrogen heterocycles, and amides, as well as complex small molecules, are all tolerated. Moreover, styrene derivatives, which often engage in unproductive polymerization, isomerization, and dimerization,^56,58^ are tolerated in the transformation. The reaction proceeds well with monosubstituted as well as 1,1- and 1,2-disubstituted olefins, while trisubstituted olefins did not participate. Counterion effects seem to play a limited role: hydrotrifluoromethylation of styrene proceeded in virtually identical yields with both triflate and tetrafluoroborate counterions of the TT-CF_3_^+^ reagent. On the basis of the observed regioselectivity and radical clock cyclization product (Scheme 3B), we propose that the transformation proceeds by radical trifluoromethylation of the olefin followed by hydrogen atom abstraction of the intermediate secondary carbon radical from the hydrogen donor.

In conclusion, we have developed a new trifluoromethylating reagent, trifluoromethyl thianthrenium triflate (**1**, TT-CF_3_^+^OTf^–^), which is easily accessible from commercial starting materials in a single step. The new reagent can engage in electrophilic, nucleophilic, and radical reactions and promises to be of synthetic utility.

## Supporting Information Available

Experimental procedures, characterization data, and crystallographic data (PDF)

## Accession Codes

CCDC 2046667–2046669 contain the supplementary crystallographic data for this paper. These data can be obtained free of charge via www.ccdc.cam.ac.uk/data_request/cif, or by emailing data_request@ccdc.cam.ac.uk, or by contacting The Cambridge Crystallographic Data Centre, 12 Union Road, Cambridge CB2 1EZ, UK; fax: +44 1223 336033.
