# Supplementary material for: Trifluoromethyl Thianthrenium Triflate: A Readily Available Trifluoromethylating Reagent with Formal CF3+, CF3•, and CF3– Reactivity
Source: J Am Chem Soc. 2021 May 14;143(20):7623–8. doi: 10.1021/jacs.1c02606 (PMC8297735; doi:10.1021/jacs.1c02606)
Supplement: Supplementary file 1 — ja1c02606_si_001.pdf [file ja1c02606_si_001.pdf]

SUPPORTING INFORMATION

Trifluoromethyl Thianthrenium Triflate: A Readily Available Trifluoromethylating Reagent with Formal CF_3^+, $CF_3^•$, and $CF_3^−$ Reactivity

Hao Jia, Andreas P. Häring, Florian Berger, Li Zhang, and Tobias Ritter*

Max-Planck-Institut für Kohlenforschung
Kaiser-Wilhelm-Platz 1, D-45470 Mülheim an der Ruhr, Germany

*E-mail: ritter@mpi-muelheim.mpg.de

TABLE OF CONTENTS

TABLE OF CONTENTS 1

MATERIALS AND METHODS 8

EXPERIMENTAL DATA 10

S-(Trifluoromethyl)thianthrenium salts synthesis 10

S-(Trifluoromethyl)thianthrenium triflate (**1**, $TTCF_3^+OTf^-$) 10

S-(Trifluoromethyl)thianthrenium tetrafluoroborate (**1'**, $TTCF_3^+BF_4^-$) 11

Trifluoromethylation of aryl boronic acid 13

4-(Trifluoromethyl)-1,1'-biphenyl (**3**) 13

3-(Trifluoromethyl)quinoline (**4**) 13

1-Chloro-4-(trifluoromethyl)-2-((2-(trifluoromethyl)benzyl)oxy)benzene (**5**) 14

2-(Trifluoromethyl)benzo[*b*]thiophene (**6**) 15

Trifluoromethylation of caffeine 16

Caffeine derivative **7** 16

Mercaptopurine derivative **8** 16

Visnagin derivative **9** 17

2,6-Dimethoxy-3-(trifluoromethyl)pyridine (**10**) 17

Trifluoromethylation of aryl aldehyde 19

1,1,1,3,3,3-Hexafluoro-2-phenyl-2-propanol (**11**) 19

2,2,2-Trifluoro-1-(4-fluorophenyl)ethan-1-ol (**12**) 19

Methyl 4-(2,2,2-trifluoro-1-hydroxyethyl)benzoate (**13**) 20

Trifluoromethylation of 1,3-dicarbonyl compound 22

2-Methyl-1-phenyl-2-(trifluoromethyl)butane-1,3-dione (**14**) 22

Trifluoromethylation of thiols 23

Phenyl(trifluoromethyl)sulfane (**15**) 23

8-((Trifluoromethyl)thio)quinolone (**16**) 24

5-Chloro-2-((trifluoromethyl)thio)benzo[*d*]thiazole (**17**) 24

Methyl *N*-acetyl-*S*-(trifluoromethyl)-*L*-cysteinate (**18**) 25

Racecadotril derivative **19** 26

Hydrotrifluoromethylation of olefins 27

(3,3,3-Trifluoropropyl)benzene (**20**) 27

(4,4,4-Trifluorobutan-2-yl)benzene (**21**) 28

(Trifluoromethyl)cyclohexane (**22**) 29

2-(6,6,6-Trifluorohexyl)isoindoline-1,3-dione (**23**) 30

N-(5,5,5-Trifluoropentyl)benzamide (**24**) 31

N-(4-Chloro-2-fluorophenyl)-6,6,6-trifluorohexanamide (**25**) 32

CF_3-(1*S*)-10-camphorsulfonamide (**26**) ... 33

CF_3-Fmoc-L-Nle-OH derivative **27** ... 33

Rotenone derivative **28** ... 34

Epiandrosterone derivative **29** ... 36

D-Glucose derivative **30** ... 37

Quinine derivative **31** ... 37

Adapalene derivative **32** ... 38

Lithocholic acid derivative **33** ... 39

Diethyl 3-methyl-4-(2,2,2-trifluoroethyl)cyclopentane-1,1-dicarboxylate (**34**) ... 40

Substrate synthesis ... 41

Racecadotril derivative **S19** ... 41

N-(4-Chloro-2-fluorophenyl)pent-4-enamide (**S25**) ... 41

(1*S*)-10-Camphorsulfonamide (**S26**) ... 42

Fmoc-L-Nle-OH derivative **S27** ... 43

Allyl adapalene derivative **S32** ... 43

Reaction condition optimization for $TTCF_3^+OTf^-$ (**1**) synthesis ... 45

General procedure for $TTCF_3^+OTf^-$ (**1**) synthesis ... 45

Table S1. Evaluation of solvent ... 45

Table S2. Evaluation of reaction time ... 46

Table S3. Evaluation of concentration ... 46

Table S4. Evaluation of additive ... 46

Table S5. Evaluation of temperature and reaction time ... 47

Reaction condition optimization for hydrotrifluoromethylation of olefins ... 48

General procedure for hydrotrifluoromethylation of styrene ... 48

Table S6. Preliminary condition screening ... 48

Table S7. Evaluation of H donor ... 48

Table S8. Evaluation of base ... 49

Table S9. Second evaluation of H donor ... 49

Table S10. Evaluation of solvent and temperature ... 50

Table S11. Evaluation of reagents' amount ... 51

Table S12. Evaluation of counterions ... 51

General procedure for hydrotrifluoromethylation of aliphatic olefin ... 51

Table S13. Evaluation of solvent ... 52

Table S14. Evaluation of reagents' amount ... 52

Reaction condition optimization for trifluoromethylation of thiols ... 53

General procedure for trifluoromethylation of thiophenol ... 53

Table S15. Preliminary condition screening ... 53

Table S16. Evaluation of reagents' amount ... 53

General procedure for trifluoromethylation of aliphatic thiol ... 54

Table S17. Evaluation of base 54

Reaction condition optimization for trifluoromethylation of aryl boronic acid 55

General procedure for trifluoromethylation of aryl boronic acid 55

Table S18. Preliminary condition screening 55

Table S19. Evaluation of base 55

Table S20. Evaluation of solvent 56

Table S21. Evaluation of base and temperature 56

Table S22. Evaluation of reagents' amount 57

Reaction condition optimization for nucleophilic trifluoromethylation of aryl aldehyde 58

General procedure for nucleophilic trifluoromethylation of aryl aldehyde 58

Table S23. Evaluation of reaction temperature and reagents' amount 58

Reaction condition optimization for radical trifluoromethylation of heterocyclies 59

General procedure for radical trifluoromethylation of caffeine 59

Table S24. Evaluation of solvent and radical activation mode 59

Elemental analysis 60

Formal reports 60

Simultaneous thermal analysis (DSC-TGA) 62

Measurement details 62

$Ph_2S^+CF_3OTf^-$ data 62

Umemoto's reagent data 63

$TTCF_3^+OTf^-$ (**1**) data 63

Cyclic voltammograms of $TTCF_3^+OTf^-$ (**1**) 65

Cyclic voltammograms of *Umemoto*'s reagent 66

Cyclic voltammograms of $Ph_2SCF_3^+OTf^-$ 67

Mechanistic investigations for $TTCF_3^+OTf^-$ (**1**) synthesis 68

Cyclic voltammograms of thianthrene and triflic anhydride 68

CF_3 radical and SO_2CF_3 radical trapping experiments 68

CF_3 radical and SO_2CF_3 radical control experiment 74

EPR Measurements 74

DFT Calculation 76

Methods 76

X-RAY CRYSTALLOGRAPHIC ANALYSIS 80

S-(Trifluoromethyl)thianthrenium triflate (**1**, $TTCF_3^+OTf^-$) (CCDC 2046668) 80

Experimental 80

Table 25. Crystal data and structure refinement 80

Analysis 81

S-(Trifluoromethyl)thianthrenium tetrafluoroborate (**1'**, $TTCF_3^+BF_4^-$) (CCDC 2046669) 82

Experimental 82

Table 26. Crystal data and structure refinement 82

5-(Trifluoromethyl)dibenzothiophenium triflate (*Umemoto*'s reagent) (CCDC 2046667) 84

Experimental 84

Table 27. Crystal data and structure refinement 84

SPECTROSCOPIC DATA 86

^{1}H NMR of *S*-(trifluoromethyl)thianthrenium triflate (**1**) 86

^{19}F NMR of *S*-(trifluoromethyl)thianthrenium triflate (**1**) 87

^{13}C NMR of *S*-(trifluoromethyl)thianthrenium triflate (**1**) 88

^{1}H NMR of *S*-(trifluoromethyl)thianthrenium tetrafluoroborate (**1'**) 89

^{19}F NMR of *S*-(trifluoromethyl)thianthrenium tetrafluoroborate (**1'**) 90

^{13}C NMR of *S*-(trifluoromethyl)thianthrenium tetrafluoroborate (**1'**) 91

^{1}H NMR of racecadotril derivative **S19** 92

^{13}C NMR of racecadotril derivative **S19** 93

$CDCl_3$, 25 ºC ^{1}H NMR of *N*-(4-chloro-2-fluorophenyl)pent-4-enamide (**S25**) 93

^{19}F NMR of *N*-(4-chloro-2-fluorophenyl)pent-4-enamide (**S25**) 95

^{13}C NMR of *N*-(4-chloro-2-fluorophenyl)pent-4-enamide (**S25**) 96

^{1}H NMR of (1*S*)-10-camphorsulfonamide (**S26**) 97

^{13}C NMR of (1*S*)-10-camphorsulfonamide (**S26**) 98

^{1}H NMR of Fmoc-L-Nle-OH derivative **S27** 99

^{13}C NMR of Fmoc-L-Nle-OH derivative **S27** 100

^{1}H NMR of allyl adapalene derivative **S32** 101

^{13}C NMR of allyl adapalene derivative **S32** 102

^{1}H NMR of 4-(trifluoromethyl)-1,1'-biphenyl (**3**) 103

^{19}F NMR of 4-(trifluoromethyl)-1,1'-biphenyl (**3**) 104

^{13}C NMR of 4-(trifluoromethyl)-1,1'-biphenyl (**3**) 105

^{1}H NMR of 3-(trifluoromethyl)quinoline (**4**) 106

^{19}F NMR of 3-(trifluoromethyl)quinoline (**4**) 107

^{13}C NMR of 3-(trifluoromethyl)quinoline (**4**) 108

^{1}H NMR of 1-chloro-4-(trifluoromethyl)-2-((2-(trifluoromethyl)benzyl)oxy)benzene (**5**) 109

^{19}F NMR of 1-chloro-4-(trifluoromethyl)-2-((2-(trifluoromethyl)benzyl)oxy)benzene (**5**) 110

^{13}C NMR of 1-chloro-4-(trifluoromethyl)-2-((2-(trifluoromethyl)benzyl)oxy)benzene (**5**) 111

^{1}H NMR of 2-(trifluoromethyl)benzo[*b*]thiophene (**6**) 112

^{19}F NMR of 2-(trifluoromethyl)benzo[*b*]thiophene (**6**) 113

^{13}C NMR of 2-(trifluoromethyl)benzo[*b*]thiophene (**6**) 114

^{1}H NMR of caffeine derivative **7** 115

^{19}F NMR of caffeine derivative **7** 116

^{13}C NMR of caffeine derivative **7** 117

^{1}H NMR of methyl 4-(2,2,2-trifluoro-1-hydroxyethyl)benzoate (**13**) 127

^{19}F NMR of methyl 4-(2,2,2-trifluoro-1-hydroxyethyl)benzoate (**13**) 128

^{13}C NMR of methyl 4-(2,2,2-trifluoro-1-hydroxyethyl)benzoate (**13**) 129

^{1}H NMR of 8-((trifluoromethyl)thio)quinolone (**16**) 133

^{19}F NMR of 8-((trifluoromethyl)thio)quinolone (**16**) 134

^{13}C NMR of 8-((trifluoromethyl)thio)quinolone (**16**) 135

^{1}H NMR of 5-chloro-2-((trifluoromethyl)thio)benzo[*d*]thiazole (**17**) 136

^{19}F NMR of 5-chloro-2-((trifluoromethyl)thio)benzo[*d*]thiazole (**17**) 137

^{13}C NMR of 5-chloro-2-((trifluoromethyl)thio)benzo[*d*]thiazole (**17**) 138

^{1}H NMR of methyl *N*-acetyl-*S*-(trifluoromethyl)-*L*-cysteinate (**18**) 139

^{19}F NMR of methyl *N*-acetyl-*S*-(trifluoromethyl)-*L*-cysteinate (**18**) 140

^{13}C NMR of methyl *N*-acetyl-*S*-(trifluoromethyl)-*L*-cysteinate (**18**) 141

^{1}H NMR of racecadotril derivative **19** 142

^{19}F NMR of racecadotril derivative **19** 143

^{13}C NMR of racecadotril derivative **19** 144

1H NMR of 2-(6,6,6-trifluorohexyl)isoindoline-1,3-dione (**23**) 145

^{19}F NMR of 2-(6,6,6-trifluorohexyl)isoindoline-1,3-dione (**23**) 146

^{13}C NMR of 2-(6,6,6-trifluorohexyl)isoindoline-1,3-dione (**23**) 147

^{1}H NMR of *N*-(5,5,5-trifluoropentyl)benzamide (**24**) 148

^{19}F NMR of *N*-(5,5,5-trifluoropentyl)benzamide (**24**) 149

^{13}C NMR of *N*-(5,5,5-trifluoropentyl)benzamide (**24**) 150

^{1}H NMR of *N*-(4-chloro-2-fluorophenyl)-6,6,6-trifluorohexanamide (**25**) 151

^{19}F NMR of *N*-(4-chloro-2-fluorophenyl)-6,6,6-trifluorohexanamide (**25**) 152

^{13}C NMR of *N*-(4-chloro-2-fluorophenyl)-6,6,6-trifluorohexanamide (**25**) 153

^{1}H NMR of CF_3-(1*S*)-10-camphorsulfonamide (**26**) 154

^{19}F NMR of CF_3-(1*S*)-10-camphorsulfonamide (**26**) 155

^{13}C NMR of CF_3-(1*S*)-10-camphorsulfonamide (**26**) 156

^{1}H NMR of CF_3-Fmoc-L-Nle-OH derivative **27** 156

^{19}F NMR of CF_3-Fmoc-L-Nle-OH derivative **27** 158

^{13}C NMR of CF_3-Fmoc-L-Nle-OH derivative **27** 159

^{1}H NMR of rotenone derivative **28a** 160

^{19}F NMR of rotenone derivative **28a** 161

^{13}C NMR of rotenone derivative **28a** 162

HMQC of rotenone derivative **28a** 163

HSQC of rotenone derivative **28a** 163

COSY of rotenone derivative **28a** 165

NOESY of rotenone derivative **28a** 166

HOESY of rotenone derivative **28a** 167

^{1}H NMR of rotenone derivative **28b** 168

^{19}F NMR of rotenone derivative **28b** 169

^{13}C NMR of rotenone derivative **28b** 170

HSQC of rotenone derivative **28b** 171

HMQC of rotenone derivative **28b** 172

COSY of rotenone derivative **28b** 173

NOESY of rotenone derivative **28b** 174

HOESY of rotenone derivative **28b** 175

^{1}H NMR of epiandrosterone derivative **29** 176

^{19}F NMR of epiandrosterone derivative **29** 177

^{13}C NMR of epiandrosterone derivative **29** 178

^{1}H NMR of D-glucose derivative **30** 179

^{19}F NMR of D-glucose derivative **30** 180

^{13}C NMR of D-glucose derivative **30** 181

^{1}H NMR of quinine derivative **31** 182

^{19}F NMR of quinine derivative **31** 183

^{13}C NMR of quinine derivative **31** 184

^{1}H NMR of adapalene derivative **32** 185

^{19}F NMR of adapalene derivative **32** 186

^{13}C NMR of adapalene derivative **32** 187

^{1}H NMR of lithocholic acid derivative **33** 188

^{19}F NMR of lithocholic acid derivative **33** 189

^{13}C NMR of lithocholic acid derivative **33** 190

^{1}H NMR of diethyl 3-methyl-4-(2,2,2-trifluoroethyl)cyclopentane-1,1-dicarboxylate (**34**) 191

^{19}F NMR of diethyl 3-methyl-4-(2,2,2-trifluoroethyl)cyclopentane-1,1-dicarboxylate (**34**) 192

^{13}C NMR of diethyl 3-methyl-4-(2,2,2-trifluoroethyl)cyclopentane-1,1-dicarboxylate (**34**) 193

REFERENCES 194

MATERIALS AND METHODS

All reactions were carried out under ambient atmosphere unless otherwise stated. High-resolution mass spectra were obtained using *Q Exactive Plus* from *Thermo*. Concentration under reduced pressure was performed by rotary evaporation at 23–40 °C at an appropriate pressure. Purified compounds were further dried under vacuum (10^{-6}–10^{-3} bar). Yields refer to purified and spectroscopically pure compounds or mixtures of constitutional isomers. All air- and moisture-sensitive manipulations were performed using standard Schlenk- and glove-box techniques under an atmosphere of argon or dinitrogen.

Solvents

Dichloromethane, anhydrous DMSO, and anhydrous DMF were purchased from Fisher Scientific GmbH. Anhydrous 1,4-dioxane and anhydrous acetonitrile were obtained from Phoenix Solvent Drying Systems. *i*-Butanol was degassed by three freeze-pump-thaw cycles. All deuterated solvents were purchased from Eur*iso*-Top.

Chromatography

Thin layer chromatography (TLC) was performed using EMD TLC silica gel 60 F_{254} plates pre-coated with 250 µm thickness silica gel 60 F_{254} and visualized by fluorescence quenching under UV light, $KMnO_4$ stain, or vanillin-H_2SO_4 stain. Flash column chromatography was performed using silica gel (40–63 µm particle size) purchased from Geduran. Preparatory high-performance liquid chromatographic separation was executed on a Shimadzu Prominence Preparative HPLC system with an YMC Pack Pro C18 HPLC column.

Spectroscopy and instruments

NMR spectra were recorded on a Bruker *Ascend™* 500 spectrometer operating at 500 MHz, 471 MHz, and 126 MHz, for ^{1}H, ^{19}F, and ^{13}C acquisitions, respectively, or on a Bruker *Ascend™* 300 spectrometer operating at 300 MHz, 282 MHz, and 75 MHz, for ^{1}H, ^{19}F, and ^{13}C acquisitions, respectively. Chemical shifts are reported in ppm with the solvent residual peak as the internal standard.[1] For ^{1}H NMR: $CDCl_3$, δ 7.26; CD_3OD, δ 3.31; $(CD_3)_2SO$, δ 2.50; CD_3CN, δ 1.94, CD_2Cl_2, δ 5.32. For ^{13}C NMR: $CDCl_3$, δ 77.16; CD_3OD, δ 49.00; $(CD_3)_2SO$, δ 39.52; CD_3CN, δ 1.32, CD_2Cl_2, δ 53.84. ^{19}F NMR spectra were referenced using a unified chemical shift scale based on the ^{1}H NMR resonance of tetramethylsilane (1% (v/v) solution in the respective solvent).[2] Data is reported as follows: s = singlet, d = doublet, t = triplet, q = quartet, m = multiplet, br = broad; coupling constants in Hz; integration. For cyclic voltammetry a *MF-2013* electrode (99.95% Pt, 1.6 mm diameter) from *Bioanalytic systems Inc.* was used as working electrode.

Starting materials

All substrates and materials were used as received from commercial suppliers, unless otherwise stated. For convenient operation, Cs_2CO_3 was stored in a N_2-filled glovebox after being dried at 150 °C for 36 h under high-vacuum. Substrates **S29**,[3] **S30**,[4] **S31**,[5] and **S33**[3] were prepared according to published procedures.

X-ray crystallographic Analysis

A crystal was mounted on a nylon loop using perfluoropolyether, and transferred to a Bruker AXS Enraf-

Nonius KappaCCD diffractometer (either MoKα radiation, λ=0.71073 Å or CuKα radiation, λ=1.54178 Å) equipped with an Oxford Cryosystems nitrogen flow apparatus. The sample was held at 100(2) K during the experiment. The structures were solved by the direct methods procedure and refined by invariom tool.[6] Non-hydrogen atoms were refined anisotropically, and hydrogen atoms were allowed to ride on the respective atoms.

EXPERIMENTAL DATA

S-(Trifluoromethyl)thianthrenium salts synthesis

S-(Trifluoromethyl)thianthrenium triflate (1, $TTCF_3^+OTf^-$)

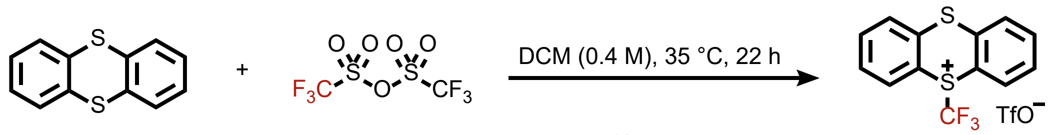

thianthrene 1.0 equiv. 1.1 equiv. *S*-(trifluoromethyl)thianthrenium triflate (**1**, $TTCF_3^+OTf^-$)

Under an ambient atmosphere, a 500 mL two-neck round-bottom flask equipped with a teflon-coated magnetic stirring bar, was charged with thianthrene (17.3 g, 80.0 mmol, 1.00 equiv.) and dichloromethane (200 mL, c = 0.4 M). Subsequently, triflic anhydride (24.8 g, 14.8 mL, 88.0 mmol, 1.10 equiv.) was added in one portion at room temperature. Upon addition of triflic anhydride, the reaction mixture rapidly turned light purple and gradually deepened, accompanied by formation of suspended particles. The reaction mixture was stirred at 35 ºC for 22 h. Subsequently, a saturated aqueous $NaHCO_3$ solution (ca. 100 mL) was added carefully. At this point, the purple color faded away, and the suspension turned light brown. The suspension was poured into a 500 mL separatory funnel, and the aqueous layer was discarded. The organic layer (light brown solution) was concentrated to dryness under reduced pressure, resulting in the formation of a light brown residue. Diethyl ether (100 mL) was added to the residue and the suspension was stirred vigorously at room temperature for 30 min. The mixture was allowed to stand for 5 min, subsequently, the solvent was decanted carefully. In order to obtain an analytically pure compound, the decanting process was repeated four times with diethyl ether. The resulting yellow slurry was concentrated to dryness under reduced pressure, accumulating to a total of 21.1 g (63%) of *S*-(trifluoromethyl)thianthrenium triflate (**1**, $TTCF_3^+OTf^-$) as pale yellow solid. In most cases, *S*-(trifluoromethyl)thianthrenium triflate (**1**, $TTCF_3^+OTf^-$) can be used directly in subsequent transformations without chromatographic purification or recrystallization.

S-(Trifluoromethyl)thianthrenium triflate (**1**, $TTCF_3^+OTf^-$) can be purified by recrystallization using a mixture of DCM and *n*-pentane (approximately 1:1 (v:v)) or by chromatography on silica gel eluting with DCM:MeOH (30:1 (v:v)) to afford a colorless solid.

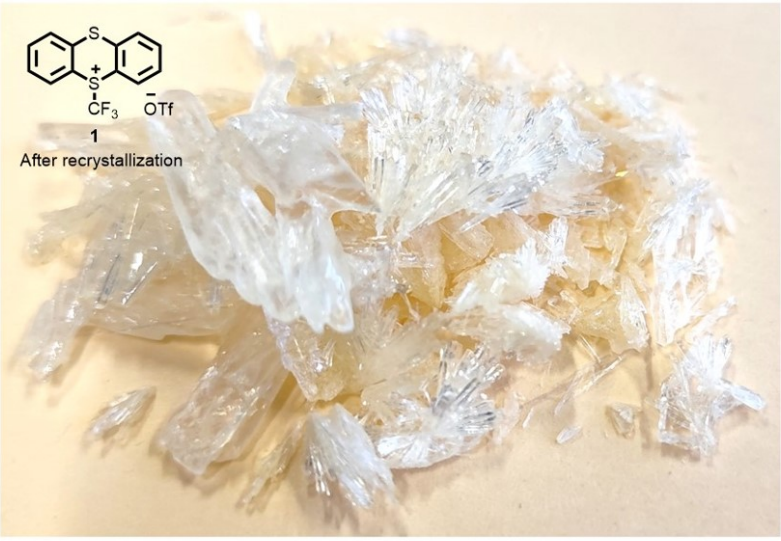

R*f* (DCM:MeOH, 10:1 (v:v)) = 0.35 (UV).

NMR Spectroscopy:

^{1}H NMR (500 MHz, $CDCl_3$, 25 ºC, δ): 8.56 (dd, *J* = 8.1, 1.4 Hz, 1H), 7.90 (ddd, *J* = 8.4, 7.4, 1.3 Hz, 1H), 7.82 (dd, *J* = 8.1, 1.3 Hz, 1H), 7.76 (ddd, *J* = 8.4, 7.4, 1.3 Hz, 1H).

^{13}C NMR (126 MHz, $CDCl_3$, 25 ºC, δ): 137.1, 136.7, 136.6, 130.3, 129.6, 124.4 (q, *J* = 337.3 Hz), 120.7 (q, *J* = 320.2 Hz), 108.7.

^{19}F NMR (471 MHz, $CDCl_3$, 25 ºC, δ): –51.15, –78.40.

HRMS ESI^{+} (m/z) calc'd for $C_{13}H_8S_2F_3$ [M-$C_1S_1O_2F_3$]$^{+}$, 285.0013; found, 285.0014, deviation: 0.4 ppm.

IR (powder sample, using attenuated total reflectance (ATR) spectroscopy) (neat, thin film): ν_{max}(cm^{-1}) = 3064, 1568, 1456, 1279, 1221, 1153, 1082, 1026, 781, 758, 633, 540, 517, 461.

Melting point: 142–143 °C. The melting process is accompanied by decomposition, see *Simultaneous thermal analysis* section for discussion.

Elemental analysis: C%, theoretical value: 38.71%, measured value: 38.67%. H%, theoretical value: 1.86%, measured value: 1.88%.

UV-Vis Spectrum

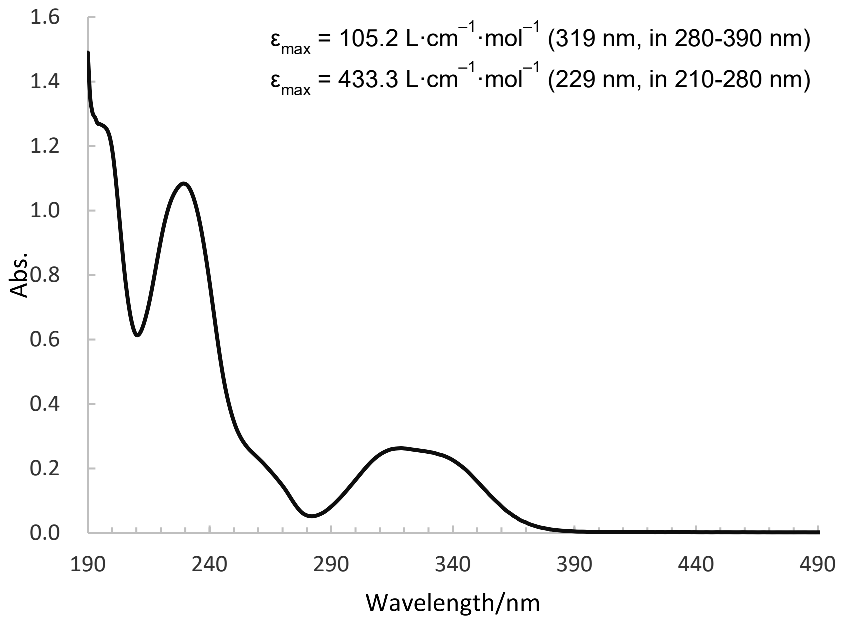

Figure S1. UV-Vis spectrum of $TTCF_3^{+}OTf^{-}$ in water

***S*-(Trifluoromethyl)thianthrenium tetrafluoroborate (1', $TTCF_3^{+}BF_4^{-}$)**

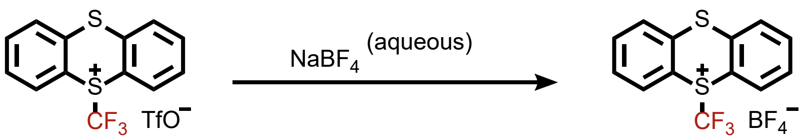

S-(trifluoromethyl)thianthrenium triflate (**1**, $TTCF_3^{+}OTf^{-}$)

92%, 1.03 g

S-(trifluoromethyl)thianthrenium tetrafluoroborate (**1'**, $TTCF_3^{+}BF_4^{-}$)

Under an ambient atmosphere, *S*-(trifluoromethyl)thianthrenium triflate (**1**, $TTCF_3^+OTf^–$, 1.30 g, 3.00 mmol) was dissolved in dichloromethane (20 mL, c = 0.15 M). The dichloromethane solution was washed with aqueous $NaBF_4$ solution (c = 10% (w/w), 4 × 20 mL), dried over anhydrous $MgSO_4$, filtered, and the solvent was removed under reduced pressure. The title product (**1'** $TTCF_3^+BF_4^–$) was obtained as yellow solid (1.03 g, 92%).

$\mathbf{R}_f$ (DCM:MeOH, 10:1 (v:v)) = 0.35 (UV).

NMR Spectroscopy:

^{1}**H NMR** (500 MHz, CD_3CN, 25 ºC, δ): 8.36 (dt, *J* = 7.8, 0.9 Hz, 1H), 8.03 – 7.98 (m, 2H), 7.83 (ddd, *J* = 8.5, 5.2, 3.5 Hz, 1H).

^{13}**C NMR** (126 MHz, CD_3CN, 25 ºC, δ): 138.2, 137.8, 137.3, 131.3, 131.0, 125.2 (q, *J* = 336.2 Hz, CF_3), 109.7.

^{19}**F NMR** (471 MHz, CD_3CN, 25 ºC, δ): –51.28, –151.31, –151.36.

HRMS ESI$^+$ (m/z) calc'd for $C_{13}H_8S_2F_3$ $[M-BF_4]^+$, 285.0013; found, 285.0014, deviation: 0.3 ppm.

IR (neat, thin film): $\nu_{max}(cm^{-1})$ = 3082, 1568, 1452,1433, 1314, 1232, 1217, 1179, 1049, 876, 772, 700, 520, 433.

Melting point: 179–181 °C

Elemental analysis: C%, theoretical value: 41.96%, measured value: 41.95%. H%, theoretical value: 2.17%, measured value: 2.16%.

Trifluoromethylation of aryl boronic acid

4-(Trifluoromethyl)-1,1'-biphenyl (3)

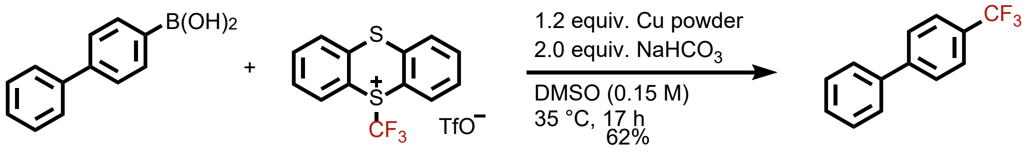

Under an ambient atmosphere, a 4 mL vial equipped with a teflon-coated magnetic stirring bar, was charged with 4-biphenylboronic acid (**S3**, 59.4 mg, 0.300 mmol, 1.00 equiv.), trifluoromethyl thianthrenium triflate (**1**, 261 mg, 0.600 mmol, 2.00 equiv.), Cu powder (22.9 mg, 0.360 mmol, 1.20 equiv.), and $NaHCO_3$ (50.4 mg, 0.600 mmol, 2.00 equiv.). The vial was transferred into a N_2-filled glovebox. Subsequently, dry DMSO (2 mL, c = 0.15 M) was added into the vial. The vial was capped, then it was transferred out of the glovebox. The reaction mixture was stirred at 35 °C for 17 h. The reaction mixture was added into 20 mL EtOAc in a separatory funnel, then the organic phase was washed with water (3 × 10 mL), dried over Na_2SO_4, and concentrated to roughly 3 mL under reduced pressure. Silica gel (approximately 500 mg) was added, and the mixture was evaporated to dryness. The residue was purified by column chromatography on silica gel eluting with pentane to afford 41.0 mg of the title compound (**3**) as colorless solid (62% yield).

R_f (pentane) = 0.55 (UV).

NMR Spectroscopy:

^{1}H NMR (500 MHz, $CDCl_3$, 25 °C, δ): 7.70 (s, 4H), 7.62 – 7.59 (m, 2H), 7.50 – 7.47 (m, 2H), 7.44 – 7.37 (m, 1H).

^{13}C NMR 126 MHz, $CDCl_3$, 25 °C, δ): 144.9, 139.9, 129.5 (q, J = 32.4 Hz), 129.1, 128.3, 127.6, 127.4, 125.9 (q, J = 3.8 Hz), 124.5 (q, J = 271.9 Hz, CF_3).

^{19}F NMR (471 MHz, $CDCl_3$, 25 °C, δ): –62.40.

HRMS GC-CI Isobutan (m/z) calc'd for $C_{13}H_{10}F_3$ $[M+H]^+$, 223.0728; found, 223.0729, deviation: 0.3 ppm.

3-(Trifluoromethyl)quinoline (4)

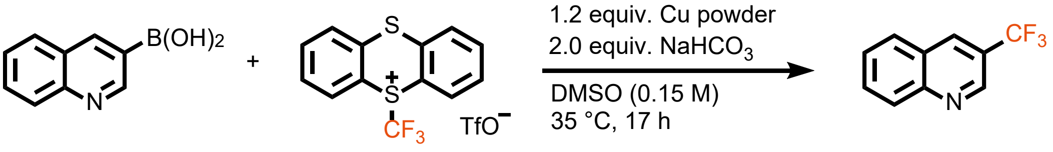

Under an ambient atmosphere, a 4 mL vial equipped with a teflon-coated magnetic stirring bar, was charged with quinolin-3-ylboronic acid (**S4**, 51.9 mg, 0.300 mmol, 1.00 equiv.), trifluoromethyl thianthrenium triflate (**1**, 261 mg, 0.600 mmol, 2.00 equiv.), Cu powder (22.9 mg, 0.360 mmol, 1.20 equiv.), and $NaHCO_3$ (50.4 mg, 0.600 mmol, 2.00 equiv.). The vial was transferred into a N_2-filled glovebox. Subsequently, dry DMSO (2 mL, c = 0.15 M) was added into the vial. The vial was capped, then it was transferred out of the glovebox. The reaction mixture was stirred at 35 °C for 17 h. The reaction mixture

was added into 20 mL EtOAc in a separatory funnel, then the organic phase was washed with water (3 × 10 mL), dried over Na_2SO_4, then filtered. The solvent was removed under reduced pressure. The residue was purified by column chromatography on silica gel eluting with a solvent mixture of EtOAc:pentane (1:25 (v:v)) to afford 47.9 mg of the title compound (**4**) as colorless solid (81% yield).

R*f* (EtOAc:pentane, 1:4 (v:v)) = 0.21 (UV).

NMR Spectroscopy:

^{1}H NMR (500 MHz, $CDCl_3$, 25 °C, δ): 9.11 (d, *J* = 2.1 Hz, 1H), 8.45 (s, 1H), 8.19 (d, *J* = 8.5 Hz, 1H), 7.93 (d, *J* = 8.2 Hz, 1H), 7.86 (ddd, *J* = 8.4, 6.8, 1.3 Hz, 1H), 7.67 (ddd, *J* = 8.0, 6.8, 0.9 Hz, 1H).

^{13}C NMR (75 MHz, $CDCl_3$, 25 °C, δ): 149.5 (q, *J* = 1.0 Hz), 146.2 (q, *J* = 3.3 Hz), 134.1 (q, *J* = 4.2 Hz), 131.9, 129.8, 128.7, 128.1, 126.4, 123.8 (q, *J* = 272.4 Hz, CF_3), 123.7 (q, *J* = 32.9 Hz).

^{19}F NMR (471 MHz, $CDCl_3$, 25 °C, δ): –61.80.

HRMS ESIpos (m/z) calc'd for $C_{10}H_7N_1F_3$ $[M+H]^+$, 198.0527; found, 198.0525, deviation: –0.7 ppm.

1-Chloro-4-(trifluoromethyl)-2-((2-(trifluoromethyl)benzyl)oxy)benzene (5)

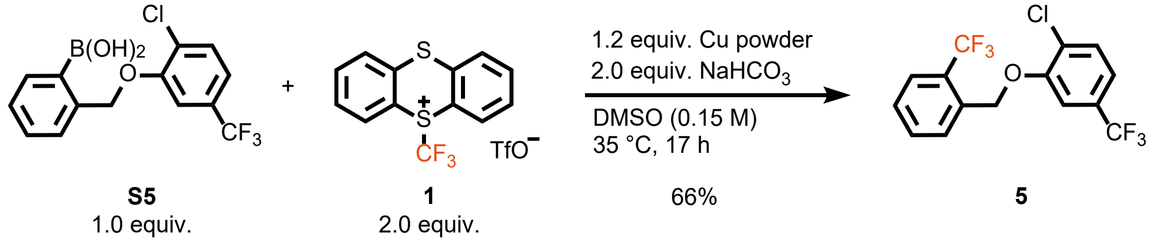

Under an ambient atmosphere, a 4 mL vial equipped with a teflon-coated magnetic stirring bar, was charged with (2-((2-chloro-5-(trifluoromethyl)phenoxy)methyl)phenyl)boronic acid (**S5**, 99.1 mg, 0.300 mmol, 1.00 equiv.), trifluoromethyl thianthrenium triflate (**1**, 261 mg, 0.600 mmol, 2.00 equiv.), Cu powder (22.9 mg, 0.360 mmol, 1.20 equiv.), and $NaHCO_3$ (50.4 mg, 0.600 mmol, 2.00 equiv.). The vial was transferred into a N_2-filled glovebox. Subsequently, dry DMSO (2 mL, c = 0.15 M) was added into the vial. The vial was capped, then it was and transferred out from the glovebox. The reaction mixture was stirred at 35 °C for 17 h. The reaction mixture was added into 20 mL EtOAc in a separatory funnel, then the organic phase was washed with water (3 × 10 mL), dried over Na_2SO_4, and concentrated to roughly 3 mL under reduced pressure. Silica gel (approximately 500 mg) was added, and the mixture was evaporated to dryness. The residue was purified by column chromatography on silica gel eluting with pentane to afford 70.1 mg of the title compound (**5**) as colorless solid (66% yield).

R*f* (pentane) = 0.54 (UV).

NMR Spectroscopy:

^{1}H NMR (300 MHz, $CDCl_3$, 25 °C, δ): 7.87 (d, *J* = 7.8 Hz, 1H), 7.72 (dd, *J* = 7.9, 1.3 Hz, 1H), 7.62 (t, *J* = 7.6 Hz, 1H), 7.52 (dd, *J* = 8.1, 1.0 Hz, 1H), 7.46 (t, *J* = 7.7 Hz, 1H), 7.24 – 7.16 (m, 1H), 5.37 (s, 2H).

^{13}C NMR 126 MHz, $CDCl_3$, 25 °C, δ): 154.0, 134.2 (q, *J* = 1.2 Hz), 132.3, 130.8, 130.2 (q, *J* = 32.8 Hz), 128.4, 128.1, 127.3 (d, *J* = 31.0 Hz), 127.2 (q, *J* = 1.4 Hz), 126.0 (q, *J* = 5.6 Hz), 124.3 (q, *J* = 273.6 Hz, CF_3), 123.5 (d, *J* = 272.2 Hz, CF_3), 11.8 (q, *J* = 4.1 Hz), 110.4 (q, *J* = 3.8 Hz), 67.1 (q, *J* = 3.2 Hz).

^{19}F NMR {1H} (282 MHz, $CDCl_3$, 25 ºC, δ): –60.35, –62.66.

HRMS GC-EI (m/z) calc'd for $C_{15}H_9O_1Cl_1F_3$ $[M]^+$, 354.0242; found, 354.0241, deviation: –0.3 ppm.

2-(Trifluoromethyl)benzo[*b*]thiophene (6)

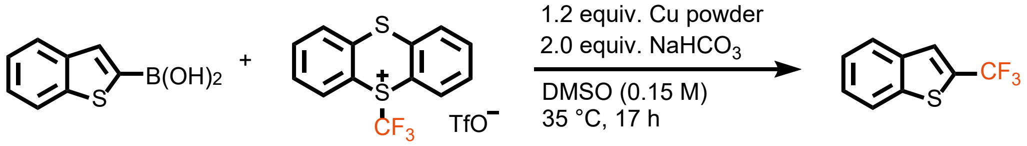

S6 1.0 equiv. **1** 2.0 equiv. 61% **6**

Under an ambient atmosphere, a 4 mL vial equipped with a teflon-coated magnetic stirring bar, was charged with benzo[*b*]thiophen-2-ylboronic acid (**S6**, 53.4 mg, 0.300 mmol, 1.00 equiv.), trifluoromethyl thianthrenium triflate (**1**, 261 mg, 0.600 mmol, 2.00 equiv.), Cu powder (22.9 mg, 0.360 mmol, 1.20 equiv.), and $NaHCO_3$ (50.4 mg, 0.600 mmol, 2.00 equiv.). The vial was transferred into a N_2-filled glovebox. Subsequently, dry DMSO (2 mL, c = 0.15 M) was added into the vial. The vial was capped, then it was transferred out of the glovebox. The reaction mixture was stirred at 35 ºC for 17 h. The reaction mixture was added into 20 mL EtOAc in a separatory funnel, then the organic phase was washed with water (3 × 10 mL), dried over Na_2SO_4, and concentrated to roughly 3 mL under reduced pressure. Silica gel (approximately 500 mg) was added, and the mixture was evaporated to dryness. The residue was purified by column chromatography on silica gel eluting with pentane to afford 37.0 mg of the title compound (**6**) as colorless solid (61% yield).

R_f (pentane) = 0.62 (UV).

NMR Spectroscopy:

^{1}H NMR (500 MHz, $CDCl_3$, 25 ºC, δ): 7.91 – 7.85 (m, 2H), 7.70 (s, 1H), 7.49 – 7.42 (m, 2H).

^{13}C NMR (75 MHz, $CDCl_3$, 25 ºC, δ): 140.3, 137.9, 131.4 (q, J = 37.9 Hz), 126.7, 125.8 (q, J = 3.9 Hz), 125.4, 125.3, 122.8, 122.7 (d, J = 269.4 Hz, CF_3).

^{19}F NMR (471 MHz, $CDCl_3$, 25 ºC, δ): –56.30.

HRMS EI (m/z) calc'd for $C_9H_5S_1F_3$ $[M]^+$, 202.0059; found, 202.0059, deviation: –0.3 ppm.

Trifluoromethylation of caffeine

Caffeine derivative 7

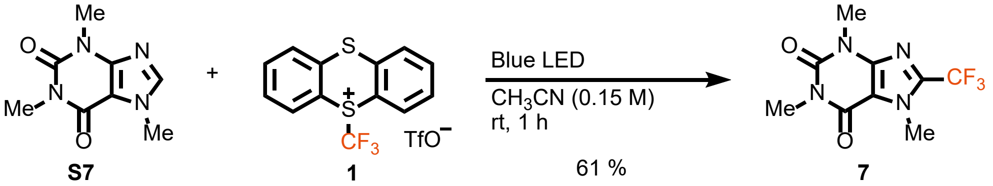

S7 3.0 equiv. **1** 1.0 equiv. **7**

Under an ambient atmosphere, a 4 mL vial containing a teflon-coated magnetic stirring bar, was charged with caffeine (**S7**, 0.900 mmol, 174 mg, 3.00 equiv.) and trifluoromethyl thianthrenium triflate (**1**, 0.300 mmol, 130 mg, 1.00 equiv.). The vial was transferred into a N_2-filled glovebox. After adding dry CH_3CN (2 mL, c = 0.15 M), the vial was capped, then it was transferred out of the glovebox. The reaction mixture was stirred under blue LED (34 W) at room temperature for 1 h. The solvent was removed under reduced pressure. The residue was purified by column chromatography on silica gel eluting with a solvent mixture of EtOAc:pentane (1:15 gradient to 1:5 (v:v)) to afford 48.0 mg of the title compound (**7**) as colorless solid (61% yield).

R*f* (EtOAc:pentane, 1:3 (v:v)) = 0.32 (UV).

NMR Spectroscopy:

^{1}H NMR (500 MHz, $CDCl_3$, 25 °C, δ): 4.15 (q, *J* = 1.3 Hz, 3H), 3.59 (s, 3H), 3.42 (s, 3H).

^{13}C NMR (126 MHz, $CDCl_3$, 25 °C, δ): 155.6, 151.4, 146.6, 139.0 (q, *J* = 40.0 Hz), 118.3 (q, *J* = 271.3 Hz, CF_3), 109.7, 33.3, 30.0, 28.3.

^{19}F NMR (471 MHz, $CDCl_3$, 25 °C, δ): –62.39.

HRMS GC-EI (m/z) calc'd for $C_9H_9N_4O_2F_3$ $[M]^+$,262.0675; found, 262.0672 deviation: –1.2 ppm.

Mercaptopurine derivative 8

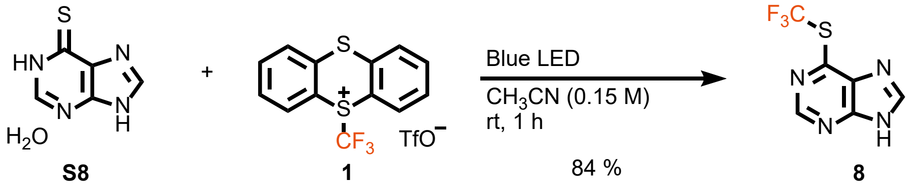

S8 3.0 equiv. **1** 1.0 equiv. **8**

Under an ambient atmosphere, a 4 mL vial containing a teflon-coated magnetic stirring bar, was charged with mercaptopurine monohydrate (**S8**, 0.900 mmol, 153 mg, 3.00 equiv.) and trifluoromethyl thianthrenium triflate (**1**, 0.300 mmol, 130 mg, 1.00 equiv.). The vial was transferred into a N_2-filled glovebox. After adding dry CH_3CN (2 mL, c = 0.15 M), the vial was capped, then it was transferred out of the glovebox. The reaction mixture was stirred under blue LED (34 W) at room temperature for 1 h. The solvent was removed under reduced pressure. The residue was purified by column chromatography on silica gel eluting with a solvent mixture of EtOAc:pentane (1:10 gradient to 7:3 (v:v)) to afford 55.4 mg of the title compound (**8**) as white solid (84% yield).

R*f* (pure EtOAc) = 0.44 (UV).

NMR Spectroscopy:

^{1}H NMR (500 MHz, CD_3CN, 25 ºC, δ): 11.33 (br, 1H), 8.81 (s, 1H), 8.30 (s, 1H).

^{13}C NMR (126 MHz, CD_3CN, 25 ºC, δ): 153.0, 152.0, 150.9, 145.0, 132.4, 129.6 (q, J = 308.1 Hz, CF_3).

^{19}F NMR (471 MHz, CD_3CN, 25 ºC, δ): –38.76.

HRMS GC-EI (m/z) calc'd for $C_6H_3N_4F_3S$ $[M]^+$,220.0028; found, 220.0025 deviation: –0.2 ppm.

Visnagin derivative 9

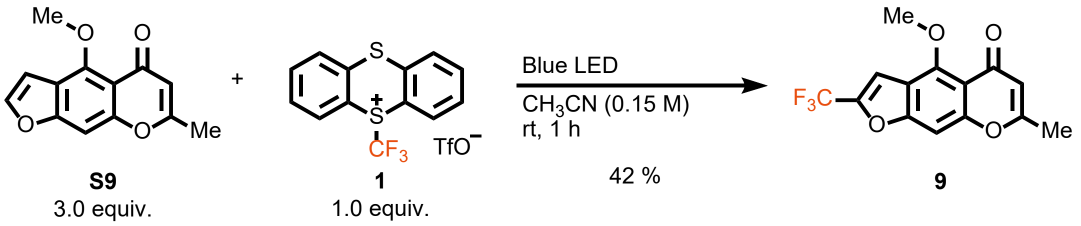

Under an ambient atmosphere, a 4 mL vial containing a teflon-coated magnetic stirring bar, was charged with visnagin (**S9**, 0.900 mmol, 207 mg, 3.00 equiv.) and trifluoromethyl thianthrenium triflate (**1**, 0.300 mmol, 130 mg, 1.00 equiv.). The vial was transferred into a N_2-filled glovebox. After adding dry CH_3CN (2 mL, c = 0.15 M), the vial was capped, then it was transferred out of the glovebox. The reaction mixture was stirred under blue LED (34 W) at room temperature for 1 h.The solvent was removed under reduced pressure. The residue was purified by column chromatography on silica gel eluting with a solvent mixture of EtOAc:pentane (1:1 gradient to 5:2 (v:v)) to afford 37.5 mg of the title compound (**9**) as white solid (42% yield).

R_f (pure EtOAc) = 0.42 (UV).

NMR Spectroscopy:

^{1}H NMR (500 MHz, $CDCl_3$, 25 ºC, δ): 7.43 (t, J = 1.2 Hz, 1H), 7.28 (d, J = 1.0 Hz, 1H), 6.07 (d, J = 0.9 Hz, 1H), 4.19 (s, 3H), 2.35 (d, J = 0.8 Hz, 3H).

^{13}C NMR (126 MHz, CDCl3, 25 ºC, δ): 177.9, 164.3, 157.7, 157.5, 155.3, 143.8 (q, J = 42.6 Hz), 119.0 (q, J = 268.2 Hz, CF_3), 115.8, 113.4, 111.3, 107.3 (q, J = 3.2 Hz), 95.8, 62.4, 20.1.

^{19}F NMR (471 MHz, $CDCl_3$, 25 ºC, δ): –65.10.

HRMS GC-EI (m/z) calc'd for $C_{14}H_9O_4F_3$ $[M]^+$,298.0451; found, 298.0447 deviation: –1.3 ppm.

2,6-Dimethoxy-3-(trifluoromethyl)pyridine (10)

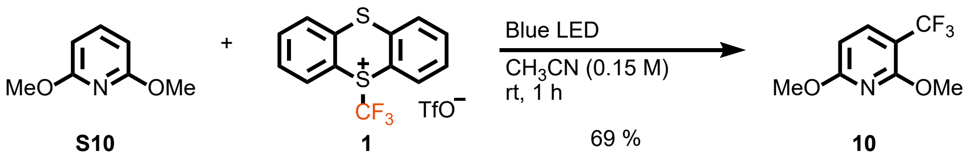

Under an ambient atmosphere, a 4 mL vial containing a teflon-coated magnetic stirring bar, was charged with trifluoromethyl thianthrenium triflate (**1**, 0.300 mmol, 130 mg, 1.00 equiv.). The vial was transferred into a N_2-filled glovebox. Subsequently, dry CH_3CN (2 mL, c = 0.15 M) was added into the vial. The vial was

sealed with a teflon-lined screw cap and removed from the glovebox. After adding 2,6-dimethoxypyridine (**S10**, 125 mg, 119 μL, 0.300 mmol, 1.00 equiv.), the reaction mixture was stirred under blue LED (34 W) at room temperature for 1 h. Subsequently, trifluorotoluene (43.8 mg, 40.0 μL, 0.300 mmol, 1.00 equiv.) was added as an internal standard. The reaction mixture was diluted with $CDCl_3$, and the yield was determined by ^{19}F NMR spectroscopy by integration of the peak at δ –62.91 ppm of the internal standard and the peak at δ –61.89 ppm of the product (**10**, 69% yield, Figure S2). The identity of the product was further confirmed by GC-MS analysis (Figure S3). The constitutional isomer (2,6-dimethoxy-4-(trifluoromethyl)pyridine) was observed at –62.37 ppm, the yield of 2,6-dimethoxy-4-(trifluoromethyl)pyridine is less than 1%.

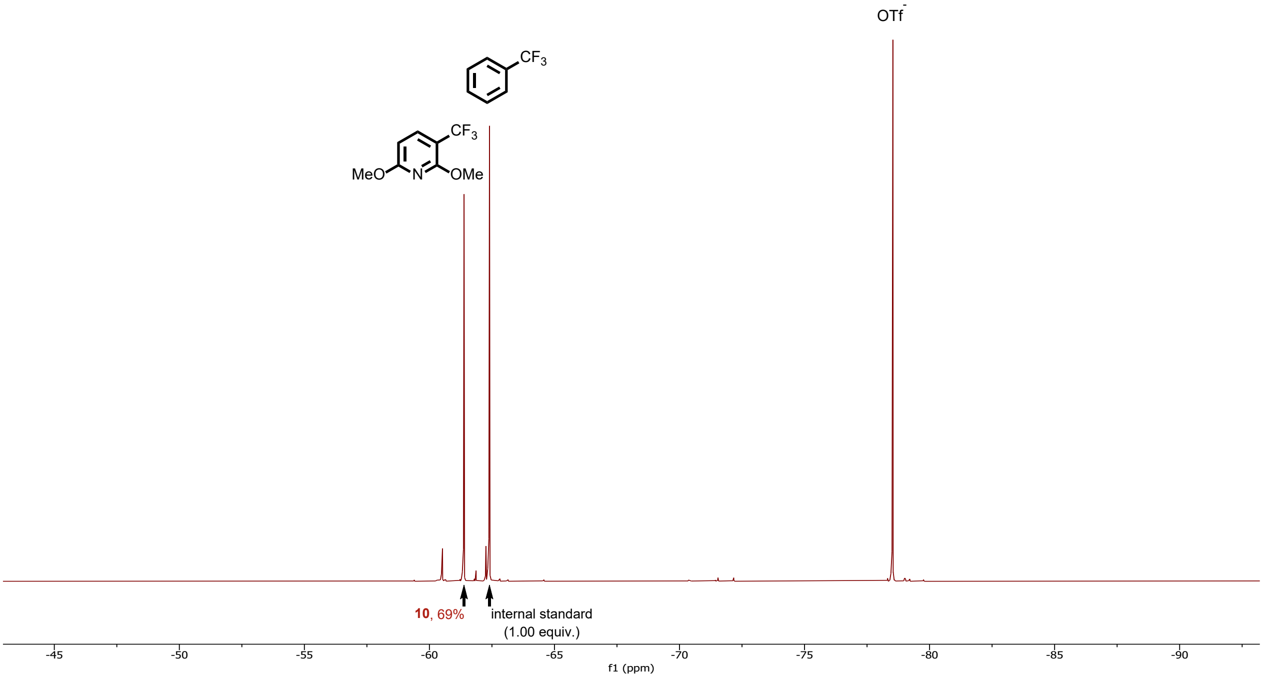

Figure S2. ^{19}F NMR of resulting mixtures for product (**10**).

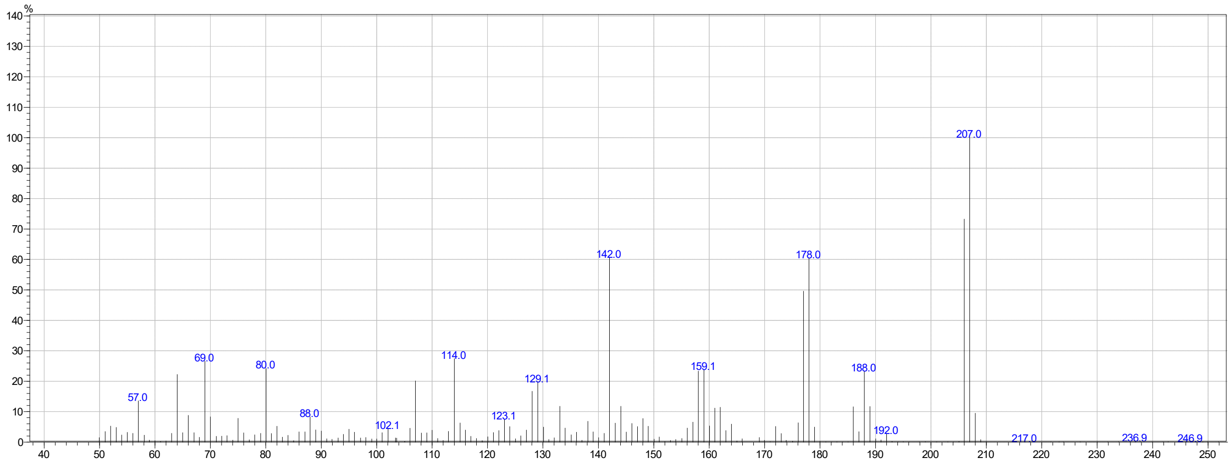

Figure S3. Mass spectrum of product (**10**).

Trifluoromethylation of aryl aldehyde

1,1,1,3,3,3-Hexafluoro-2-phenyl-2-propanol (11)

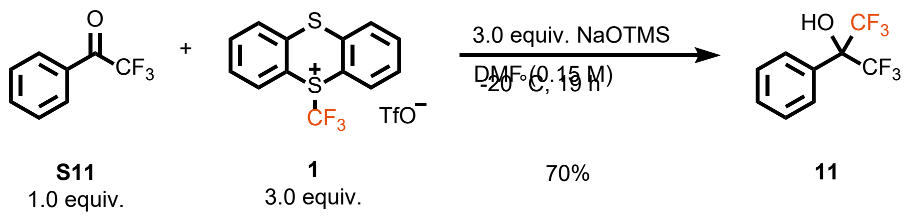

Under an ambient atmosphere, a 4 mL vial containing a teflon-coated magnetic stirring bar, was charged with trifluoromethyl thianthrenium triflate (**1**, 391 mg, 0.900 mmol, 3.00 equiv.). The vial was transferred into a N_2-filled glovebox. Subsequently, NaOTMS (101 mg, 0.900 mmol, 3.00 equiv.) was added into the vial. The vial was capped, then it was transferred out of the glovebox. After adding dry DMF (2 mL, c = 0.15 M) and 2,2,2-trifluoroacetophenone (**S11**, 52.2 mg, 42.1 µL, 0.300 mmol, 1.00 equiv.), the reaction mixture was stirred at –20 ºC for 19 h. EtOAc (20 mL) was added, and the resulting mixture was transferred to a separating funnel. The organic phase was washed with saturated aqueous NaCl (3 × 10 mL), dried over Na_2SO_4, and concentrated to roughly 0.2 mL under reduced pressure carefully. The solution was purified by column chromatography on silica gel eluting with EtOAc:pentane (pure pentane gradient to 1:20 (v:v)) to afford 23 mg of the title compound (**11**) as slight yellow liquid (32% yield). The yield was also be determined by ^{19}F NMR spectroscopy due to high volatility of the product by integration of the peak at δ –62.91 ppm of the internal standard and the peak at δ –76.41 ppm of the product (**11**, 70% yield).

R*f* (EtOAc:pentane, 1:4 (v:v)) = 0.61 (UV).

NMR Spectroscopy:

^{1}H NMR (500 MHz, $CDCl_3$, 25 ºC, δ): 7.73 (d, *J* = 9.2 Hz, 2H), 7.52 – 7.44 (m, 3H), 3.41 (s, 1H).

^{13}C NMR (126 MHz, $CDCl_3$, 25 ºC, δ): 130.4, 129.4, 128.8, 126.6, 122.8 (q, *J* = 287.3 Hz, CF_3), 77.2 (septet, *J* = 30.1 Hz).

^{19}F NMR (471 MHz, $CDCl_3$, 25 ºC, δ): –75.61.

HRMS EI (m/z) calc'd for $C_9H_6O_1F_6$ $[M]^+$, 244.0320; found, 244.0317 deviation: –0.9 ppm.

2,2,2-Trifluoro-1-(4-fluorophenyl)ethan-1-ol (12)

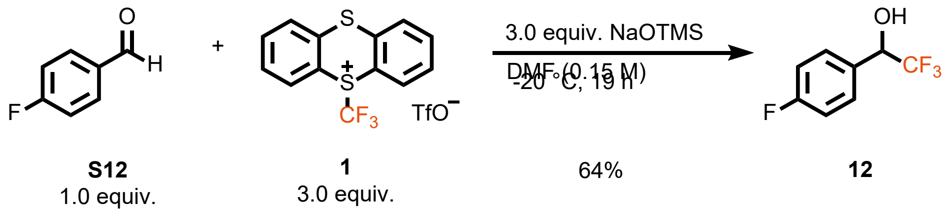

Under an ambient atmosphere, a 4 mL vial containing a teflon-coated magnetic stirring bar, was charged with trifluoromethyl thianthrenium triflate (**1**, 391 mg, 0.900 mmol, 3.00 equiv.), and NaOTMS (101 mg, 0.900 mmol, 3.00 equiv.). The vial was transferred into a N_2-filled glovebox. Subsequently the vial was capped, then it was transferred out of the glovebox. After adding dry DMF (2 mL, c = 0.15 M) and 4-fluorobenzaldehyde (**S12**, 37.2 mg, 32.2 µL, 0.300 mmol, 1.00 equiv.), the reaction mixture was stirred at

–20 ºC for 19 h. Subsequently, trifluorotoluene (43.8 mg, 40.0 µL, 0.300 mmol, 1.00 equiv.) was added as an internal standard. The reaction mixture was diluted with $CDCl_3$, and the yield was determined by ^{19}F NMR spectroscopy by integration of the peak at δ –62.91 ppm of the internal standard and the peak at δ –78.73 ppm of the product (**12**, 64% yield, Figure S4). The identity of the product was further confirmed by GC-MS analysis (Figure S5).

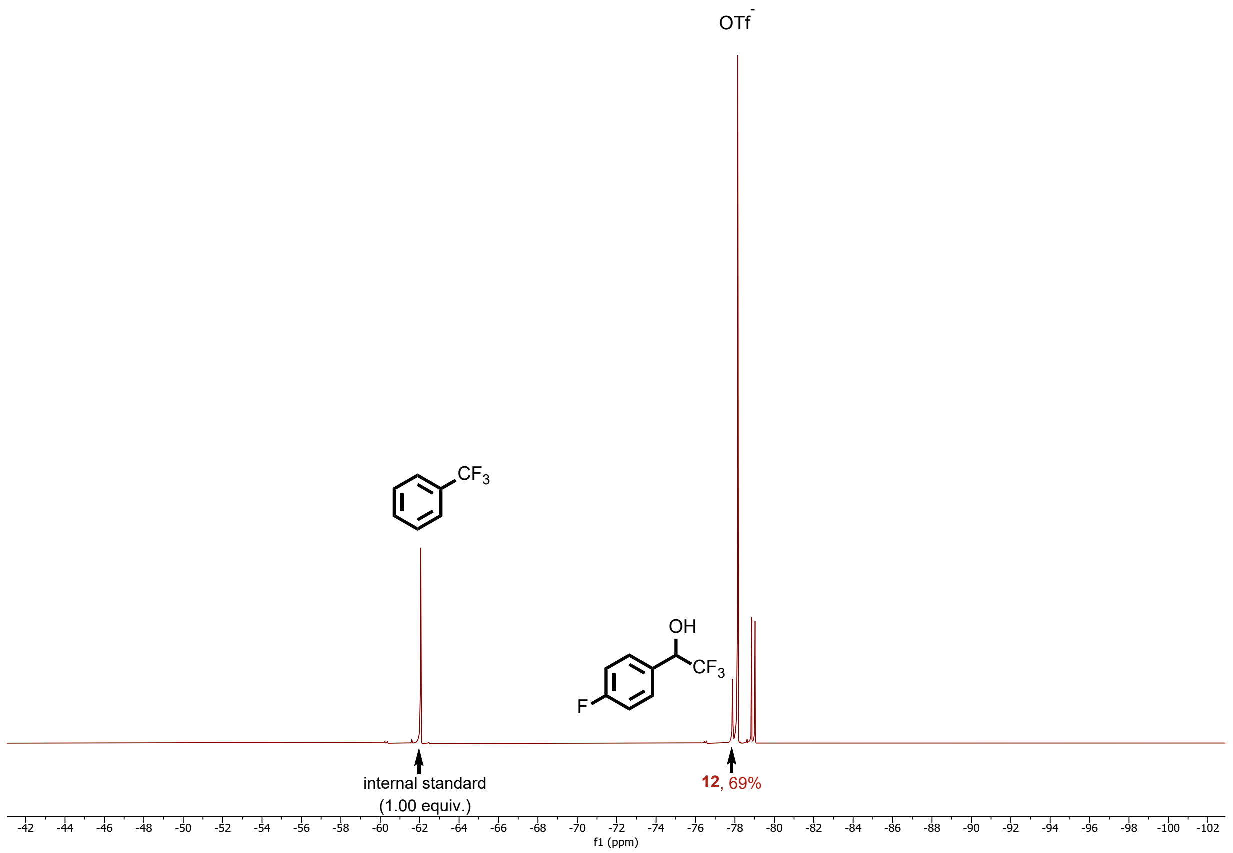

Figure S4. ^{19}F NMR of resulting mixtures for product (**12**).

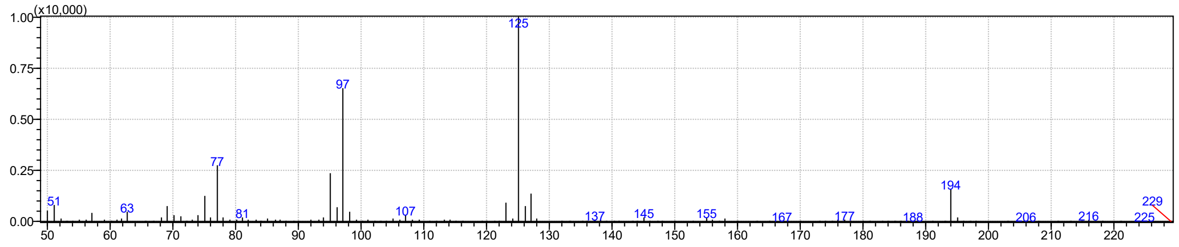

Figure S5. Mass spectrum of product (**12**).

Methyl 4-(2,2,2-trifluoro-1-hydroxyethyl)benzoate (13)

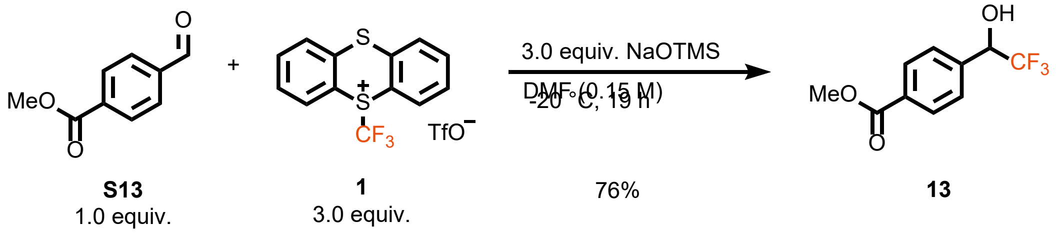

Under an ambient atmosphere, a 4 mL vial containing a teflon-coated magnetic stirring bar, was charged

with methyl 4-formylbenzoate (**S13**, 49.2 mg, 0.300 mmol, 1.00 equiv.), trifluoromethyl thianthrenium triflate (**1**, 391 mg, 0.900 mmol, 3.00 equiv.). The vial was transferred into a N_2-filled glovebox. Subsequently, NaOTMS (101 mg, 0.900 mmol, 3.00 equiv.) was added into the vial. The vial was capped, then it was transferred out of the glovebox. After adding dry DMF (2 mL, c = 0.15 M), the reaction mixture was stirred at –20 ºC for 19 h. EtOAc (20 mL) was added and the resulting mixture was transferred to a separating funnel. The organic phase was washed with water (3 × 10 mL), dried over Na_2SO_4, and concentrated to roughly 3 mL under reduced pressure. Silica gel (approximately 500 mg) was added, and the mixture was evaporated to dryness. The residue was purified by column chromatography on silica gel eluting with EtOAc:pentane (1:70 gradient to 1:10 (v:v)) to afford 53.4 mg of the title compound (**13**) as colorless solid (76% yield).

R_f (EtOAc:pentane, 1:4 (v:v)) = 0.26 (UV).

NMR Spectroscopy:

^{1}H NMR (500 MHz, $CDCl_3$, 25 ºC, δ): 8.04 (d, *J* = 8.5 Hz, 2H), 7.55 (d, *J* = 8.0 Hz, 2H), 5.09 (q, *J* = 6.7 Hz, 1H), 3.91 (s, 3H), 3.09 (br, 1H).

^{13}C NMR (126 MHz, $CDCl_3$, 25 ºC, δ): 167.0, 139.1, 130.9, 129.7, 127.6, 124.1 (q, *J* = 282.1 Hz, CF_3), 72.3 (q, *J* = 32.0 Hz), 52.4.

^{19}F NMR (471 MHz, $CDCl_3$, 25 ºC, δ): –78.17 (d, *J* = 5.7 Hz).

HRMS EI (m/z) calc'd for $C_{10}H_9O_3F_3$ $[M]^+$, 234.0499; found, 234.0498 deviation: –0.2 ppm.

TRIFLUOROMETHYLATION OF 1,3-DICARBONYL COMPOUND

2-Methyl-1-phenyl-2-(trifluoromethyl)butane-1,3-dione (14)

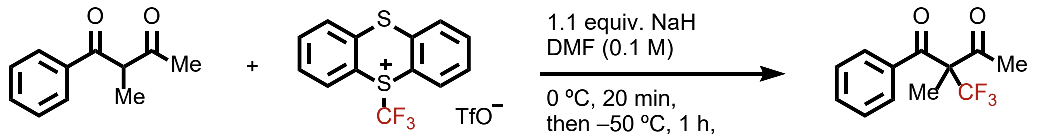

S14 1.0 equiv. **1** 1.2 equiv. **14**

Under an ambient atmosphere, a 4 mL vial containing a teflon-coated magnetic stirring bar, was charged with NaH (13 mg, 0.33 mmol, 1.1 equiv., 60 % dispersion in mineral oil). The vial was transferred into a N_2-filled glovebox. Subsequently, dry DMF was added into the vial. The vial was capped, then it was transferred out of the glovebox. After adding 2-methyl-1-phenylbutane-1,3-dione (**S14**, 52.8 mg, 49.5 µL, 0.300 mmol, 1.00 equiv.) with a Hamilton syringe, the reaction was stirred at 0 °C for 20 min and then cooled to –50 °C. trifluoromethyl thianthrenium triflate solution (**1**, 156 mg, 0.330 mmol, 1.20 equiv. in 1 dry DMF) was added into the vial. The mixture was stirred at –50 °C for 1 h and then at rt for 20 min. EtOAc (20 mL) was added and the resulting mixture was transferred to a separating funnel. The organic phase was washed with water (3 × 10 mL), dried over Na_2SO_4, and concentrated to roughly 3 mL under reduced pressure. Silica gel (approximately 500 mg) was added, and the mixture was evaporated to dryness. The residue was purified by column chromatography on silica gel eluting with pentane to afford 41.0 mg of the title compound (**14**) as colorless liquid (56% yield).

R_f (EtOAc:pentane, 1:7 (v:v)) = 0.51 (UV).

NMR Spectroscopy:

^{1}H NMR (300 MHz, $CDCl_3$, 25 ºC, δ): 7.76 (dd, *J* = 8.5, 1.3 Hz, 2H), 7.62 – 7.55 (m, 1H), 7.49 – 7.41 (m, 2H), 2.29 (s, 3H), 1.76 (s, 3H).

^{13}C NMR (75 MHz, $CDCl_3$, 25 ºC, δ): 200.2, 192.0, 135.0, 134.0, 129.2, 129.0, 124.4 (q, *J* = 284.2 Hz, CF_3), 69.3 (q, *J* = 23.2 Hz), 28.9 (q, *J* = 2.2 Hz), 16.9 (q, *J* = 2.3 Hz).

^{19}F NMR (282 MHz, $CDCl_3$, 25 ºC, δ): –67.24.

HRMS ESIpos (m/z) calc'd for $C_{12}H_{11}O_2Na_1F_3$ $[M+Na]^+$, 267.0603; found, 267.0603, deviation: 0.1 ppm.

Trifluoromethylation of thiols

Phenyl(trifluoromethyl)sulfane (15)

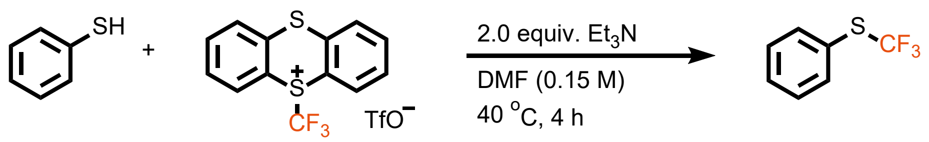

**S15**	**1**		**15**
1.0 equiv.	2.0 equiv.		

Under an ambient atmosphere, a 4 mL vial containing a teflon-coated magnetic stirring bar, was charged with trifluoromethyl thianthrenium triflate (**1**, 261 mg, 0.600 mmol, 2.00 equiv.). The vial was transferred into a N_2-filled glovebox. Subsequently, dry DMF (2 mL, c = 0.15 M) was added into the vial. The vial was sealed with a teflon-lined screw cap and removed from the glovebox. After adding thiophenol (**S15**, 33.1 mg, 30.8 μL, 0.300 mmol, 1.00 equiv.) and Et_3N (91.1 mg, 125 μL, 0.900 mmol, 3.00 equiv.), the reaction mixture was stirred at 40 ºC for 4 h. Subsequently, trifluorotoluene (43.8 mg, 40.0 μL, 0.300 mmol, 1.00 equiv.) was added as an internal standard. The reaction mixture was diluted with $CDCl_3$, and the yield was determined by ^{19}F NMR spectroscopy by integration of the peak at δ –62.91 ppm of the internal standard and the peak at δ –43.28 ppm of the product (**15**, 87% yield, Figure S6). The identity of the product was further confirmed by GC-MS analysis (Figure S7).

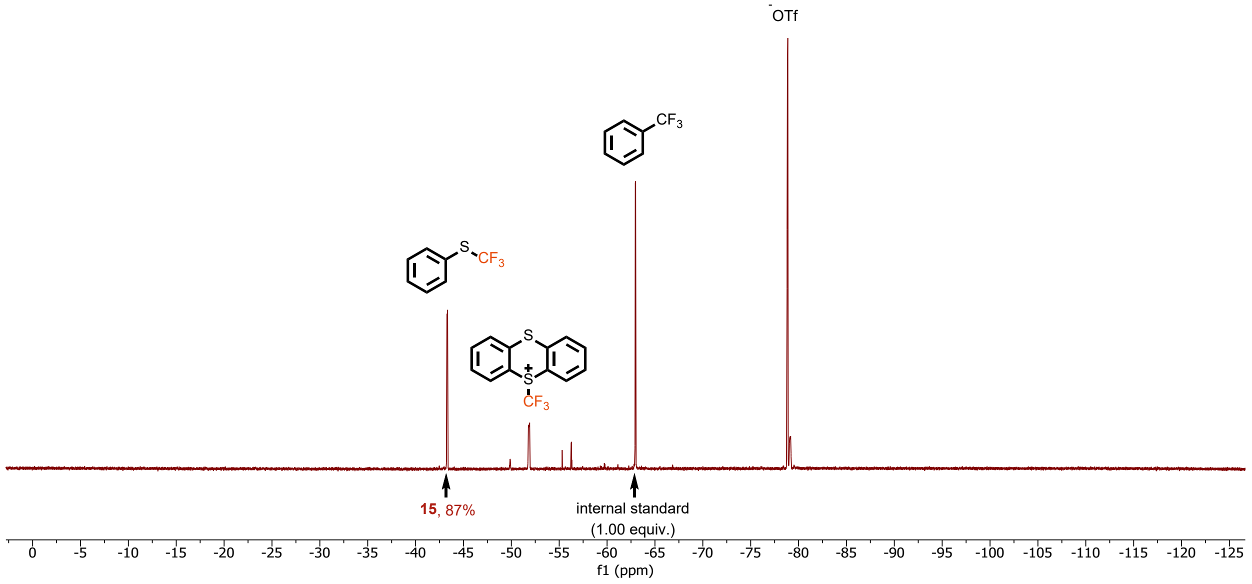

Figure S6. ^{19}F NMR of resulting mixtures for product (**15**).

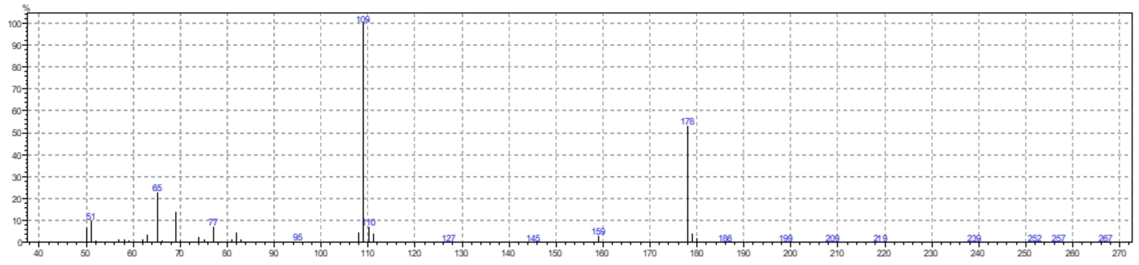

Figure S7. Mass spectrum of product (**15**).

8-((Trifluoromethyl)thio)quinolone (16)

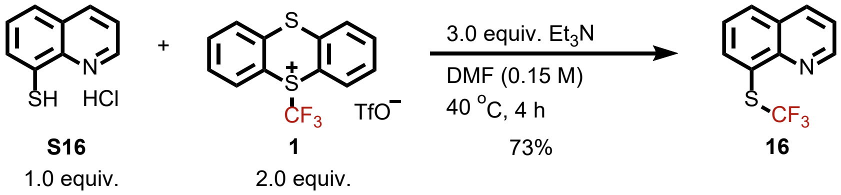

Under an ambient atmosphere, a 4 mL vial containing a teflon-coated magnetic stirring bar, was charged with thiooxine hydrochloride (**S16**, 59.3 mg, 0.300 mmol, 1.00 equiv.) and trifluoromethyl thianthrenium triflate (**1**, 261 mg, 0.600 mmol, 2.00 equiv.). The vial was transferred into a N_2-filled glovebox. Subsequently, dry DMF (2 mL, c = 0.15 M) and Et_3N (91.1 mg, 125 µL, 0.900 mmol, 3.00 equiv.) was added into the vial. The vial was sealed with a teflon-lined screw cap and removed from the glovebox. The reaction mixture was stirred at 40 °C for 4 h. EtOAc (20 mL) was added and the resulting mixture was transferred to a separating funnel. The organic phase was washed with water (3 × 10 mL). The organic phase was dried over Na_2SO_4, and the solvent was removed under reduced pressure. The residue was purified by column chromatography on silica gel eluting with a solvent mixture of EtOAc:pentane (1:20 (v:v)) to afford 49.9 mg of the title compound (**16**) as colorless liquid (73% yield).

R_f (EtOAc:pentane, 1:5 (v:v)) = 0.48 (UV).

NMR Spectroscopy:

^{1}H NMR (300 MHz, $CDCl_3$, 25 °C, δ): 9.00 (dd, *J* = 4.2, 1.7 Hz, 1H), 8.17 (dd, *J* = 8.3, 1.7 Hz, 1H), 8.06 (d, *J* = 7.4 Hz, 1H), 7.86 (dd, *J* = 8.2, 1.2 Hz, 1H), 7.61 – 7.52 (m, 1H), 7.47 (dd, *J* = 8.3, 4.2 Hz, 1H).

^{13}C NMR (75 MHz, $CDCl_3$, 25 °C, δ): 150.8, 146.8, 136.7, 134.1 (q, *J* = 1.6 Hz), 130.1 (q, *J* = 308.4 Hz, CF_3), 129.7, 128.9, 127.0, 126.7, 122.2.

^{19}F NMR (282 MHz, $CDCl_3$, 25 °C, δ): –41.09.

HRMS GC-EI (m/z) calc'd for $C_{10}H_6N_1S_1F_3$ $[M]^+$, 229.0170; found, 229.0168, deviation: –1.0 ppm.

5-Chloro-2-((trifluoromethyl)thio)benzo[*d*]thiazole (17)

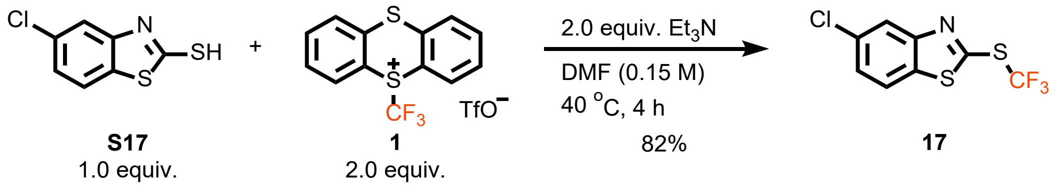

Under an ambient atmosphere, a 4 mL vial containing a teflon-coated magnetic stirring bar, was charged with 5-chloro-2-benzothiazolethiol (**S17**, 60.5 mg, 0.300 mmol, 1.00 equiv.) and trifluoromethyl thianthrenium triflate (**1**, 261 mg, 0.600 mmol, 2.00 equiv.). The vial was transferred into a N_2-filled glovebox. Subsequently, dry DMF (2 mL, c = 0.15 M) and Et_3N (60.7 mg, 83.6 µL, 0.600 mmol, 2.00 equiv.) were added into the vial. The vial was sealed with a teflon-lined screw cap and removed from the glovebox. The reaction mixture was stirred at 40 °C for 4 h. EtOAc (20 mL) was added and the resulting mixture was transferred to a separating funnel. The organic phase was washed with water (3 × 10 mL), dried over

Na_2SO_4, and concentrated to roughly 3 mL under reduced pressure. Silica gel (approximately 500 mg) was added, and the mixture was evaporated to dryness. The residue was purified by column chromatography on silica gel eluting with pentane to afford 66.1 mg of the title compound (**17**) as colorless solid (82% yield).

R*f* (pentane) = 0.18 (UV).

NMR Spectroscopy:

^{1}H NMR (300 MHz, $CDCl_3$, 25 ºC, δ): 8.10 (dd, *J* = 2.0, 0.7 Hz, 1H), 7.80 (d, *J* = 8.6 Hz, 1H), 7.46 (dd, *J* = 8.6, 2.0 Hz, 1H).

^{13}C NMR (75 MHz, $CDCl_3$, 25 ºC, δ): 154.0 (q, *J* = 3.1 Hz), 153.7, 135.4, 133.2, 128.1 (q, *J* = 311.2 Hz, CF_3), 127.2, 123.8, 122.0.

^{19}F NMR {1H} (282 MHz, $CDCl_3$, 25 ºC, δ): –39.97.

HRMS GC-EI (m/z) calc'd for $C_8H_3N_1S_2Cl_1F_3$ $[M]^+$, 268.9347; found, 268.9342, deviation: –1.7 ppm.

Methyl *N*-acetyl-*S*-(trifluoromethyl)-*L*-cysteinate (18)

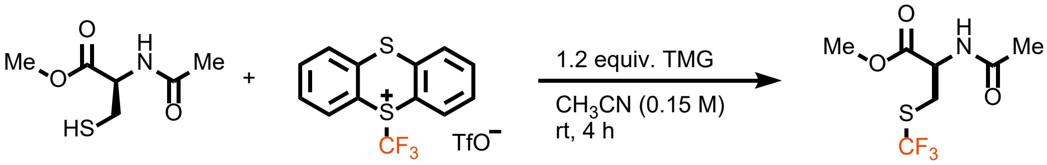

S18 1.0 equiv. | **1** 1.2 equiv. | 80% | **18**

Under an ambient atmosphere, a 4 mL vial containing a teflon-coated magnetic stirring bar, was charged with methyl acetyl-*L*-cysteinate (**S18**, 53.2 mg, 0.300 mmol, 1.00 equiv.) and trifluoromethyl thianthrenium triflate (**1**, 156 mg, 0.360 mmol, 1.20 equiv.). The vial was transferred into a N_2-filled glovebox. Subsequently, dry CH_3CN (2 mL, c = 0.15 M) and 1,1,3,3-tetramethylguanidin (TMG, 41.5 mg, 45.2 µL, 0.360 mmol, 1.20 equiv.) were added into the vial. The vial was sealed with a teflon-lined screw cap and removed from the glovebox. The reaction mixture was stirred at room temperature for 4 h. The solvent was removed under reduced pressure. The residue was purified by column chromatography on silica gel eluting with a solvent mixture of EtOAc:pentane (1:5 gradient to 1:2 (v:v)) to afford 59.4 mg of the title compound (**18**) as colorless solid (80% yield).

R*f* (EtOAc:pentane, 1:1 (v:v)) = 0.24 ($KMnO_4$).

NMR Spectroscopy:

^{1}H NMR (500 MHz, $CDCl_3$, 25 ºC, δ): 6.37 (br, 1H), 4.97 – 4.82 (m, 1H), 3.80 (s, 1H), 3.51 (dd, *J* = 14.4, 4.7 Hz, 1H), 3.35 (dd, *J* = 14.4, 4.5 Hz, 1H), 2.05 (s, 3H).

^{13}C NMR (126 MHz, $CDCl_3$, 25 ºC, δ): 170.2, 170.13, 130.6 (d, *J* = 306.4 Hz, CF_3), 53.1, 51.9, 31.8 (q, *J* = 2.0 Hz), 23.0.

^{19}F NMR {1H} (471 MHz, $CDCl_3$, 25 ºC, δ): –40.92.

HRMS ESIpos (m/z) calc'd for $C_7H_{10}N_1O_3S_1F_3Na_1$ $[M+Na]^+$, 268.0228; found, 268.0226 deviation: –0.7 ppm.

Racecadotril derivative 19

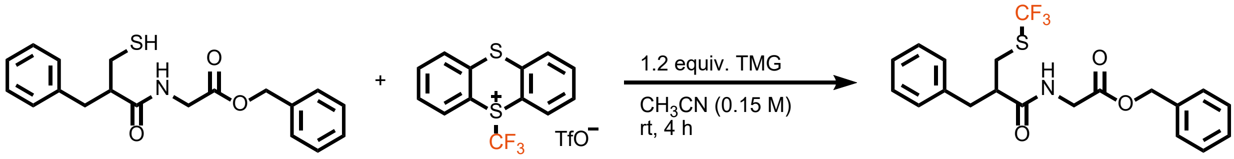

Under an ambient atmosphere, a 4 mL vial containing a teflon-coated magnetic stirring bar, was charged with racecadotril derived thiol (**S13**, 103 mg, 0.300 mmol, 1.00 equiv.) and trifluoromethyl thianthrenium triflate (**1**, 156 mg, 0.360 mmol, 1.20 equiv.). The vial was transferred into a N_2-filled glovebox. Subsequently, dry CH_3CN (2 mL, c = 0.15 M) and 1,1,3,3-tetramethylguanidin (TMG, 41.5 mg, 45.2 µL, 0.360 mmol, 1.20 equiv.) were added into the vial. The vial was sealed with a teflon-lined screw cap and removed from the glovebox. The reaction mixture was stirred at room temperature for 4 h. The solvent was removed under reduced pressure. The residue was purified by column chromatography on silica gel eluting with a solvent mixture of EtOAc:pentane (1:15 gradient to 1:8 (v:v)) to afford 80.8 mg of the title compound (**13**) as colorless solid (81% yield).

R_f (EtOAc:pentane, 1:2 (v:v)) = 0.59 (UV).

NMR Spectroscopy:

^{1}H NMR (500 MHz, $CDCl_3$, 25 °C, δ): 7.41 – 7.30 (m, 5H), 7.28 (q, *J* = 6.6, 6.2 Hz, 2H), 7.22 (ddd, *J* = 7.4, 5.2, 1.2 Hz, 1H), 7.20 – 7.13 (m, 2H), 5.93 (t, *J* = 4.5 Hz, 1H), 5.16 (d, *J* = 2.2 Hz, 2H), 4.06 (dd, *J* = 18.4, 5.3 Hz, 1H), 3.92 (dd, *J* = 18.4, 5.0 Hz, 1H), 3.17 (dd, *J* = 14.0, 9.1 Hz, 1H), 2.99 (dd, *J* = 14.0, 5.3 Hz, 1H), 2.99 – 2.85 (m, 2H), 2.77 (tt, *J* = 8.9, 5.8 Hz, 1H).

^{13}C NMR (126 MHz, $CDCl_3$, 25 °C, δ): 172.6, 169.3, 137.9, 135.1, 131.0 (q, *J* = 306.4 Hz, CF_3), 128.8, 128.7, 128.7, 128.6, 128.4, 126.9, 67.2, 49.4, 41.4, 38.4, 31.2 (q, *J* = 1.9 Hz).

^{19}F NMR {1H} (471 MHz, $CDCl_3$, 25 °C, δ): –41.05.

HRMS ESI$^+$ (m/z) calc'd for $C_{20}H_{20}N_1O_3S_1F_3Na_1$ $[M+Na]^+$, 434.1010; found, 434.1008 deviation: –0.4 ppm.

Hydrotrifluoromethylation of olefins

(3,3,3-Trifluoropropyl)benzene (20)

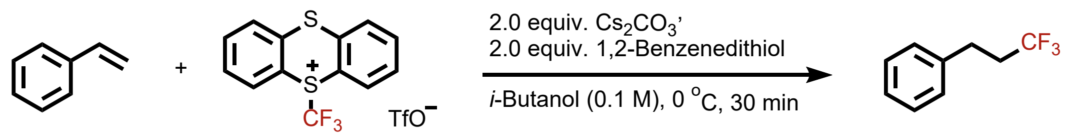

Under an ambient atmosphere, a 20 mL vial containing a teflon-coated magnetic stirring bar, was charged with trifluoromethyl thianthrenium triflate (**1**, 651 mg, 1.50 mmol, 1.50 equiv.). The vial was transferred into a N_2-filled glovebox. Subsequently, Cs_2CO_3 (651 mg, 2.00 mmol, 2.00 equiv.) was added into the vial. The vial was capped, then it was transferred out of the glovebox. After adding dry *i*-butanol (10 mL, c = 0.1 M), the reaction mixture was stirred at 0 ºC for 2 min, followed by addition of styrene (**S20**, 104 mg, 115 µL, 1.00 mmol, 1.00 equiv.) and 1,2-benzenedithiol (284 mg, 230 µL, 2.00 mmol, 2.00 equiv.). The reaction mixture was stirred at 0 ºC for 30 min. Subsequently, trifluorotoluene (292 mg, 246 µL, 1.00 mmol, 1.00 equiv.) was added as an internal standard. The reaction mixture was diluted with $CDCl_3$, and the yield was determined by ^{19}F NMR spectroscopy by integration of the peak at δ –62.91 ppm of the internal standard and the peak at δ –66.88 ppm (t, *J* = 10.2 Hz) of the product (Figure S8, **20**, 75% yield). The identity of the product was further confirmed by GC-MS analysis (Figure S9).

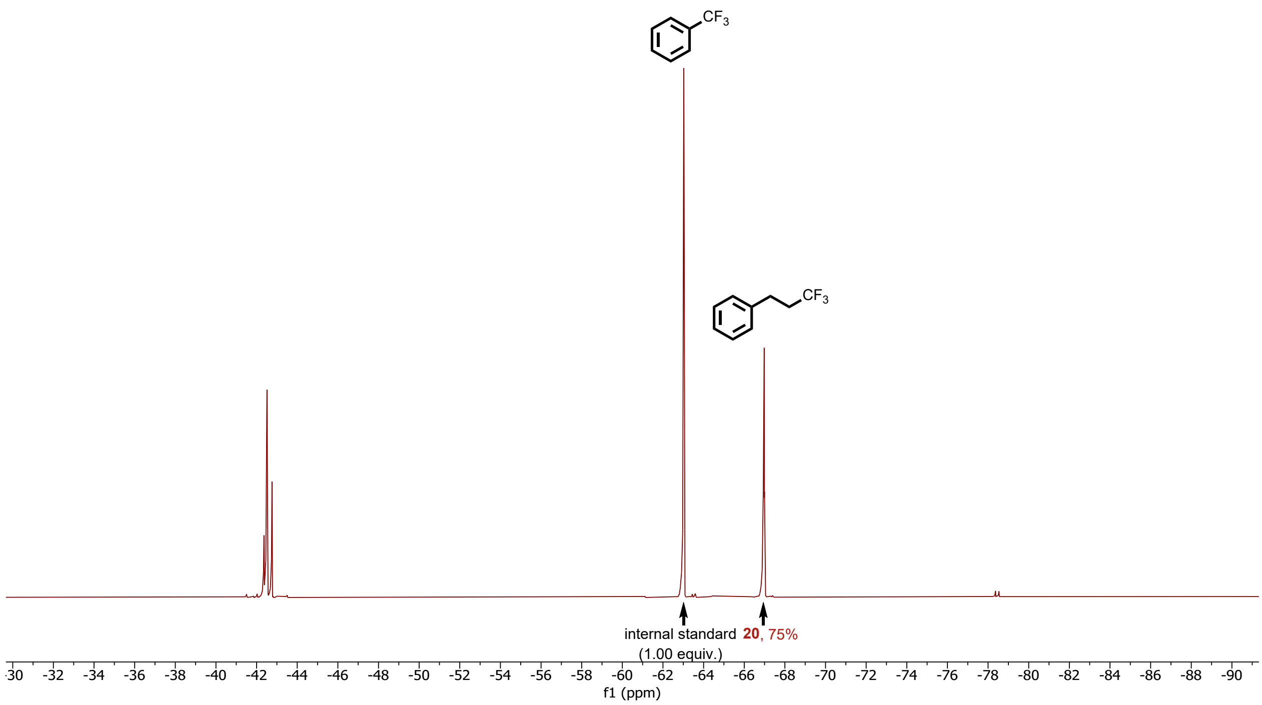

Figure S8. ^{19}F NMR of resulting mixtures for product (**20**)

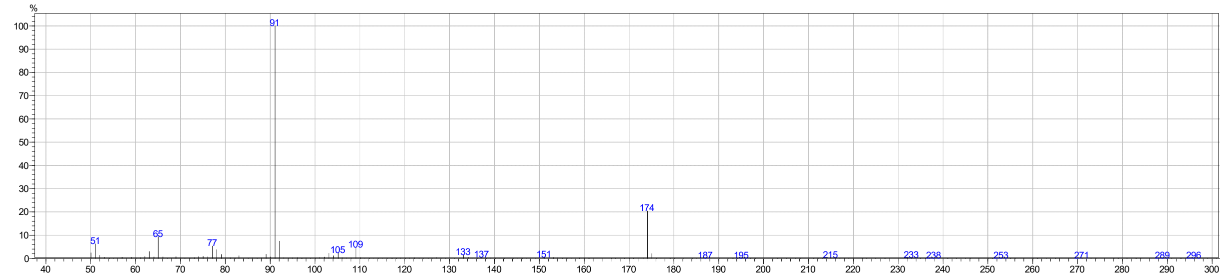

Figure S9. Mass spectrum of product (**20**).

(4,4,4-Trifluorobutan-2-yl)benzene (21)

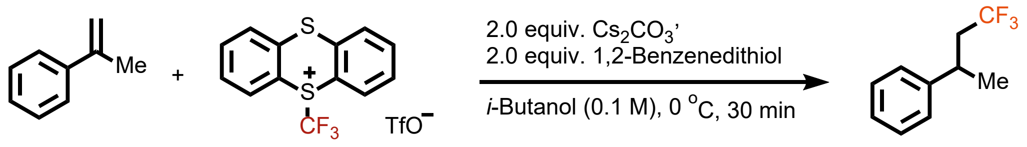

Under an ambient atmosphere, a 4 mL vial containing a teflon-coated magnetic stirring bar, was charged with trifluoromethyl thianthrenium triflate (**1**, 130 mg, 0.300 mmol, 1.50 equiv.). The vial was transferred into a N_2-filled glovebox. Subsequently, Cs_2CO_3 (130 mg, 0.400 mmol, 2.00 equiv.) was added into the vial. The vial was capped, then it was transferred out of the glovebox. After adding dry *i*-butanol (2 mL, c = 0.1 M), the reaction mixture was stirred at 0 ºC for 2 min, followed by addition of alpha-methylstyrene (**S21**, 23.6 mg, 26.0 µL, 0.200 mmol, 1.00 equiv.) and 1,2-benzenedithiol (56.9 mg, 46.0 µL, 0.40 mmol, 2.00 equiv.). The reaction mixture was stirred at 0 ºC for 30 min. Subsequently, trifluorotoluene (29.2 mg, 24.5 µL, 0.200 mmol, 1.00 equiv.) was added as an internal standard. The reaction mixture was diluted with $CDCl_3$, and the yield was determined by ^{19}F NMR spectroscopy by integration of the peak at δ –62.91 ppm of the internal standard and the peak at δ –64.08 ppm (t, J = 10.9 Hz) of the product (Figure S10, **21**, 74% yield). The identity of the product was further confirmed by GC-MS analysis (Figure S11).

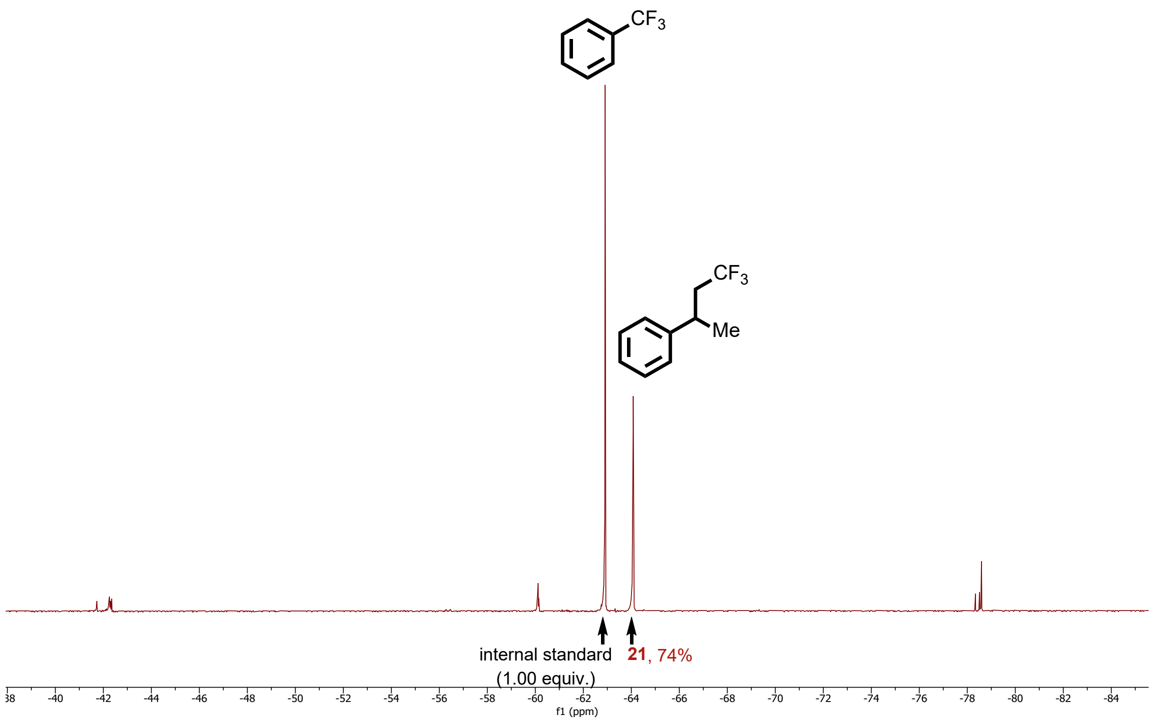

Figure S10. ^{19}F NMR of resulting mixtures for product (**21**)

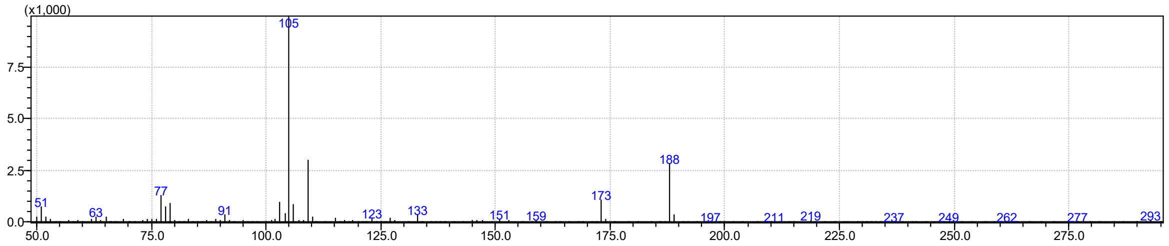

Figure S11. Mass spectrum of product (**21**) peak from GC-MS

(Trifluoromethyl)cyclohexane (22)

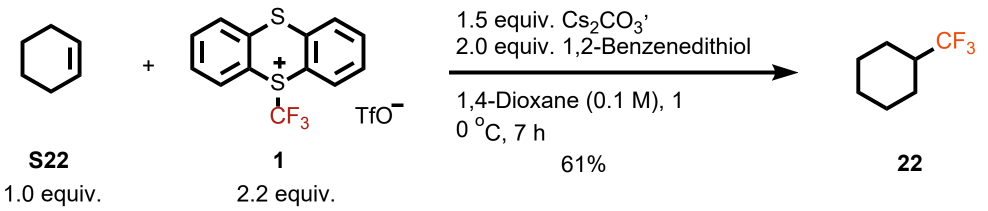

Under an ambient atmosphere, a 4 mL vial containing a teflon-coated magnetic stirring bar, was charged with trifluoromethyl thianthrenium triflate (**1**, 286 mg, 0.660 mmol, 2.20 equiv.). The vial was transferred into a N_2-filled glovebox. Subsequently, Cs_2CO_3 (146 mg, 0.450 mmol, 1.50 equiv.) was added into the vial. The vial was capped, then it was transferred out of the glovebox. After adding dry 1,4-dioxane (3 mL, c = 0.1 M), the reaction mixture was stirred at 0 ºC for 2 min, followed by addition of cyclohexene (**S22**, 24.6 mg, 30.5 μL, 0.300 mmol, 1.00 equiv.) and 1,2-benzenedithiol (85.3 mg, 69.0 μL, 0.60 mmol, 2.00 equiv.). The reaction mixture was stirred at 10 ºC for 7 h. Subsequently, trifluorotoluene (43.8 mg, 37.0 μL, 0.300 mmol, 1.00 equiv.) was added as an internal standard. The reaction mixture was diluted with $CDCl_3$, and the yield

was determined by ^{19}F NMR spectroscopy by integration of the peak at δ –62.91 ppm of the internal standard and the peak at δ –74.29 ppm (d, *J* = 9.0 Hz) of the product (Figure S12, **22**, 61% yield). The identity of the product was further confirmed by GC-MS analysis (Figure S13).

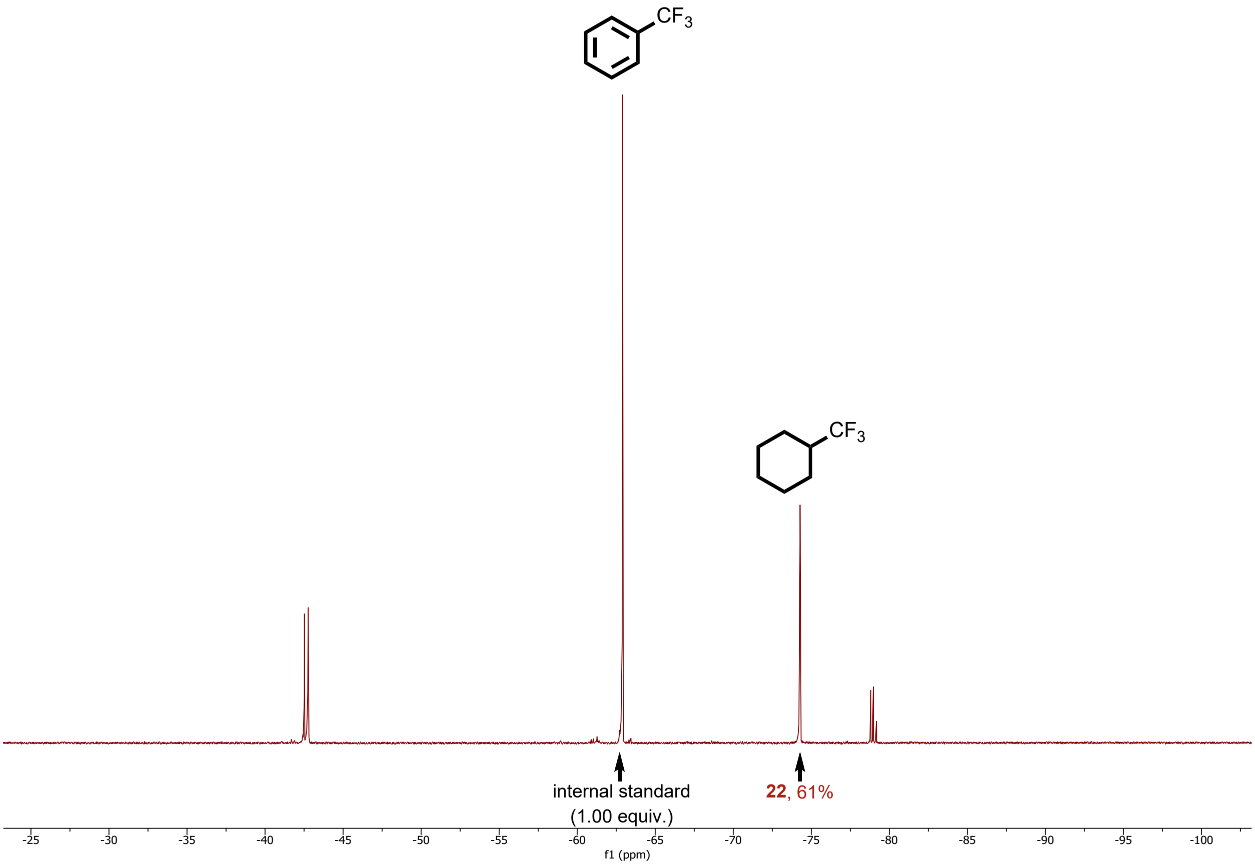

Figure S12. ^{19}F NMR of resulting mixtures for product (**22**)

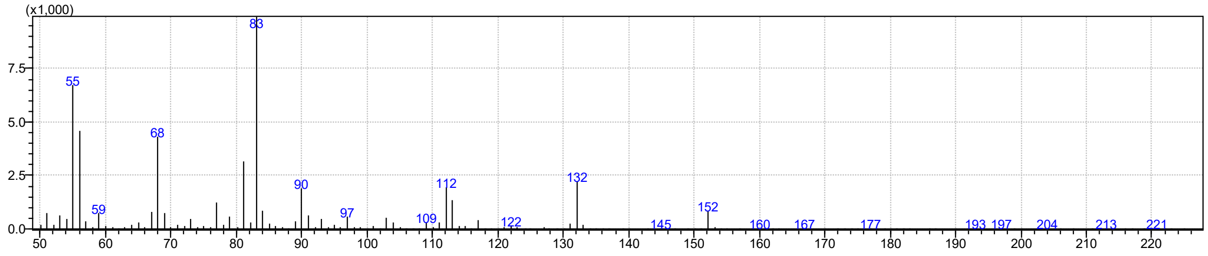

Figure S13. Mass spectrum of product (**22**).

2-(6,6,6-Trifluorohexyl)isoindoline-1,3-dione (23)

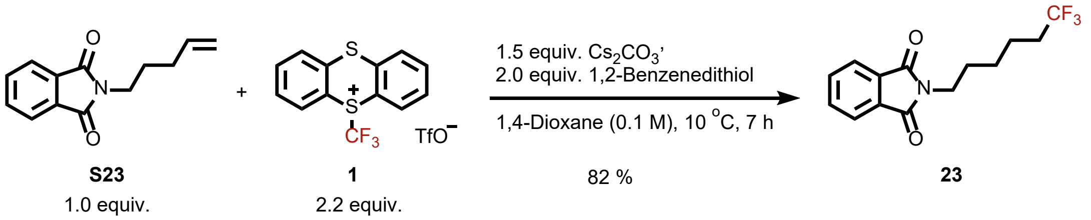

Under an ambient atmosphere, a 4 mL vial containing a teflon-coated magnetic stirring bar, was charged with phthalimide-derived olefin (**S23**, 43.0 mg, 0.200 mmol, 1.00 equiv.) and trifluoromethyl thianthrenium

triflate (**1**, 191 mg, 0.440 mmol, 2.20 equiv.). The vial was transferred into a N_2-filled glovebox. Subsequently, Cs_2CO_3 (97.7 mg, 0.300 mmol, 1.50 equiv.) was added into the vial. The vial was capped, then it was transferred out of the glovebox. After adding dry 1,4-dioxane (2 mL, c = 0.1 M), the reaction mixture was stirred at 10 ºC for 1 min, followed by addition of 1,2-benzenedithiol (56.9 mg, 46.0 µL, 0.400 mmol, 2.00 equiv.). The reaction mixture was stirred at 10 ºC for 7 h. Subsequently, the reaction mixture was filtered (0.2 µm, PTFE), and the residue was washed with DCM (0.5 mL). Silica gel (approximately 500 mg) was added into the combined transparent organic phase. Then this mixture was concentrated to dryness under reduced pressure. The residue was purified by column chromatography on silica gel eluting with a solvent mixture of EtOAc:pentane (1:30 (v:v)) to afford 47.0 mg of the title compound (**23**) as yellow liquid (82% yield).

R*f* (EtOAc:pentane, 1:8 (v:v)) = 0.34 (UV).

NMR Spectroscopy:

^{1}**H NMR** (300 MHz, $CDCl_3$, 25 ºC, δ): 7.82 (dd, *J* = 5.4, 3.1 Hz, 2H), 7.69 (dd, *J* = 5.4, 3.1 Hz, 2H), 3.68 (t, *J* = 7.1 Hz, 1H). 2.22 – 1.90 (m, 2H), 1.74 – 1.67 (m, 2H), 1.64 – 1.54 (m, 2H), 1.45 – 1.36 (m, 2H).

^{13}**C NMR** (75 MHz, $CDCl_3$, 25 ºC, δ): 168.5, 134.0, 132.2, 127.2 (q, *J* = 276.3 Hz, CF_3), 123.3, 37.7, 33.7 (q, *J* = 28.5 Hz), 28.3, 26.1, 21.6 (q, *J* = 3.0 Hz).

^{19}**F NMR {1H}** (282 MHz, $CDCl_3$, 25 ºC, δ): –66.40.

HRMS GC-EI (m/z) calc'd for $C_{14}H_{14}N_1O_2F_3$ $[M]^+$, 285.0974; found, 285.0971, deviation: –1.1 ppm.

***N*-(5,5,5-Trifluoropentyl)benzamide (24)**

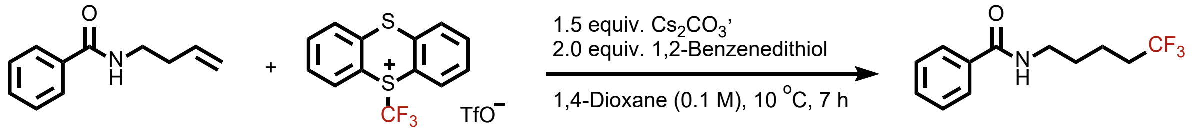

S24 1.0 equiv. **1** 2.2 equiv. **24**

Under an ambient atmosphere, a 4 mL vial containing a teflon-coated magnetic stirring bar, was charged with benzamide derived olefin (**S24**, 35.0 mg, 0.200 mmol, 1.00 equiv.) and trifluoromethyl thianthrenium triflate (**1**, 191 mg, 0.440 mmol, 2.20 equiv.). The vial was transferred into a N_2-filled glovebox. Subsequently, Cs_2CO_3 (97.7 mg, 0.300 mmol, 1.50 equiv.) was added into the vial. The vial was capped, then it was transferred out of the glovebox. After adding dry 1,4-dioxane (2 mL, c = 0.1 M), the reaction mixture was stirred at 10 ºC for 1 min, followed by addition of 1,2-benzenedithiol (56.9 mg, 46.0 µL, 0.400 mmol, 2.00 equiv.) with a Hamilton syringe. The reaction mixture was stirred at 10 ºC for 7 h. Subsequently, the reaction mixture was filtered (0.2 µm, PTFE), and the residue was washed with DCM (0.5 mL). The combined solvent was removed under reduced pressure. The residue was purified by column chromatography on silica gel eluting with a solvent mixture of EtOAc:pentane (1:30 gradient to 1:5 (v:v)) to afford 36.7 mg of the title compound (**24**) as colorless solid (75% yield).

R*f* (EtOAc:pentane, 1:2 (v:v)) = 0.29 (UV).

NMR Spectroscopy:

^{1}H NMR (500 MHz, $CDCl_3$, 25 ºC, δ): 7.79 – 7.70 (m, 2H), 7.50 – 7.44 (m, 1H), 7.39 (td, *J* = 7.7, 1.9 Hz, 2H), 6.34 (br, 1H), 3.43 (qd, *J* = 6.7, 2.3 Hz, 2H), 2.14 – 2.04 (m, 2H), 1.69 – 1.60 (m, 4H).

^{13}C NMR (126 MHz, $CDCl_3$, 25 ºC, δ): 167.9, 134.6, 131.6, 128.6, 127.1 (q, *J* = 276.3 Hz, CF_3), 127.0, 39.6, 33.4 (q, *J* = 28.6 Hz), 28.9, 19.5 (q, *J* = 3.1 Hz).

^{19}F NMR (471 MHz, $CDCl_3$, 25 ºC, δ): –66.32 (t, *J* = 10.8 Hz).

HRMS GC-EI (m/z) calc'd for $C_{12}H_{14}N_1O_1F_3$ $[M]^+$, 245.1022; found, 245.1022 deviation: 0.2 ppm.

***N*-(4-Chloro-2-fluorophenyl)-6,6,6-trifluorohexanamide (25)**

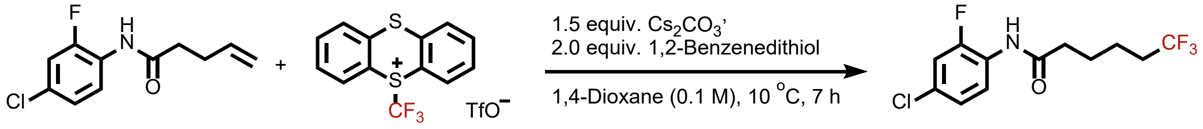

S25 1.0 equiv. | **1** 2.2 equiv. | 90 % | **25**

Under an ambient atmosphere, a 4 mL vial containing a teflon-coated magnetic stirring bar, was charged with *N*-(4-chloro-2-fluorophenyl)pent-4-enamide (**S25**, 35.0 mg, 0.200 mmol, 1.00 equiv.) and trifluoromethyl thianthrenium triflate (**1**, 191 mg, 0.440 mmol, 2.20 equiv.). The vial was transferred into a N_2-filled glovebox. Subsequently, Cs_2CO_3 (97.7 mg, 0.300 mmol, 1.50 equiv.) was added into the vial. The vial was capped, then it was transferred out of the glovebox. After adding dry 1,4-dioxane (2 mL, c = 0.1 M), the reaction mixture was stirred at 10 ºC for 1 min, followed by addition of 1,2-benzenedithiol (56.9 mg, 46.0 µL, 0.400 mmol, 2.00 equiv.) with a Hamilton syringe. The reaction mixture was stirred at 10 ºC for 7 h. Subsequently, the reaction mixture was filtered (0.2 µm, PTFE), and the residue was washed with DCM (0.5 mL). The combined solvent was removed under reduced pressure. The residue was purified by column chromatography on silica gel eluting with a solvent mixture of EtOAc:pentane (1:30 gradient to 1:15 (v:v)) to afford 53.4 mg of the title compound (**25**) as colorless solid (90% yield).

R_f (EtOAc:pentane, 1:4 (v:v)) = 0.39 (UV).

NMR Spectroscopy:

^{1}H NMR (500 MHz, $CDCl_3$, 25 ºC, δ): 8.23 (t, *J* = 8.5 Hz, 1H), 7.39 (s, 1H), 7.11 (q, *J* = 2.3 Hz, 1H), 7.09 (s, 2H), 2.43 (t, *J* = 7.4 Hz, 2H), 2.17 – 2.06 (m, 2H), 1.80 (p, *J* = 7.5 Hz, 2H), 1.64 (tt, *J* = 10.7, 6.3 Hz, 2H).

^{13}C NMR (126 MHz, $CDCl_3$, 25 ºC, δ): 170.6, 152.2 (d, *J* = 246.6 Hz, CF), 129.0 (d, *J* = 9.6 Hz), 127.1 (q, *J* = 276.6 Hz, CF_3), 125.1 (d, *J* = 10.0 Hz), 124.9 (d, *J* = 3.7 Hz), 122.7, 115.7 (d, *J* = 22.8 Hz), 37.0, 33.6 (q, *J* = 28.7 Hz), 24.4, 21.7 (q, *J* = 3.1 Hz).

^{19}F NMR (471 MHz, $CDCl_3$, 25 ºC, δ): –66.34 (t, *J* = 10.9 Hz, CF_3), –128.36 (t, *J* = 9.9 Hz).

HRMS GC-EI (m/z) calc'd for $C_{12}H_{12}N_1O_1Cl_4F_4$ $[M]^+$, 297.0537; found, 297.0538 deviation: 0.3 ppm.

CF_3-(1*S*)-10-camphorsulfonamide (26)

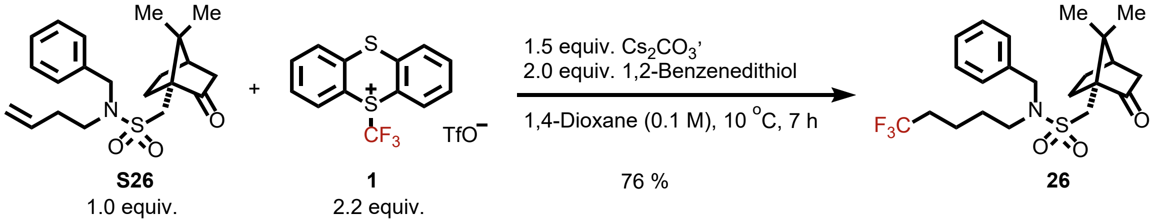

Under an ambient atmosphere, a 4 mL vial containing a teflon-coated magnetic stirring bar, was charged with trifluoromethyl thianthrenium triflate (**1**, 191 mg, 0.440 mmol, 2.20 equiv.). The vial was transferred into a N_2-filled glovebox. Subsequently, Cs_2CO_3 (97.7 mg, 0.300 mmol, 1.50 equiv.) was added into the vial. The vial was capped, then it was transferred out of the glovebox. After adding dry 1,4-dioxane (2 mL, c = 0.1 M), the reaction mixture was stirred at 10 °C for 1 min, followed by addition of (1*S*)-10-camphorsulfonamide derived olefin (**S26**, 75.1 mg, 0.200 mmol, 1.00 equiv.) and 1,2-benzenedithiol (56.9 mg, 46.0 μL, 0.400 mmol, 2.00 equiv.) with a Hamilton syringe. The reaction mixture was stirred at 10 °C for 7 h. Subsequently, the reaction mixture was filtered (0.2 μm, PTFE), and the residue was washed with DCM (0.5 mL). The combined solvent was removed under reduced pressure. The residue was purified by column chromatography on silica gel eluting with a solvent mixture of EtOAc:pentane (1:20 (v:v)) to afford 67.7 mg of the title compound (**26**) as colorless liquid (76% yield).

R_f (EtOAc:pentane, 1:4 (v:v)) = 0.34 (UV).

NMR Spectroscopy:

^{1}H NMR (500 MHz, $CDCl_3$, 25 °C, δ): 7.42 – 7.28 (m, 5H), 4.47 (d, *J* = 15.0 Hz, 1H), 4.35 (d, *J* = 15.0 Hz, 1H), 3.34 (d, *J* = 10.0 Hz, 1H) 3.34 – 3.15 (m, 2H), 2.76 (d, *J* = 14.5 Hz, 1H), 2.54 (ddd, *J* = 14.6, 11.8, 3.8 Hz, 1H), 2.38 (dt, *J* = 18.4, 3.9 Hz, 1H), 2.10 (t, *J* = 4.5 Hz,1H), 2.08 – 2.03 (m, 1H), 1.94 (d, *J* = 18.5 Hz, 1H), 1.99 – 1.88 (m, 2H), 1.67 (ddd, *J* = 14.0, 9.3, 4.6 Hz, 1H), 1.51 – 1.40 (m, 5H), 1.14 (s, 3H), 0.86 (s,3H).

^{13}C NMR (126 MHz, $CDCl_3$, 25 °C, δ): 215.5, 136.7, 128.8, 128.6, 128.0, 127.0 (q, *J* = 276.5 Hz, CF_3), 58.6, 52.2, 48.0, 47.9, 47.9, 42.9, 42.7, 33.2 (q, *J* = 28.5 Hz), 27.8, 27.0, 25.5, 20.1, 19.8, 19.1 (q, *J* = 3.3 Hz).

^{19}F NMR (471 MHz, $CDCl_3$, 25 °C, δ): –66.32 (t, *J* = 11.0 Hz).

HRMS ESI$^+$ (m/z) calc'd for $C_{22}H_{30}N_1O_3S_1F_3Na_1$ [M+Na]$^+$, 468.1791; found, 468.1791, deviation: –0.1 ppm.

CF_3-Fmoc-L-Nle-OH derivative 27

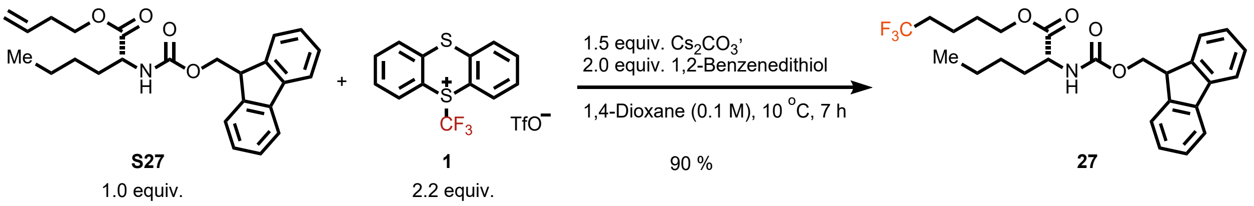

Under an ambient atmosphere, a 4 mL vial containing a teflon-coated magnetic stirring bar, was charged with Fmoc-L-Nle-OH-ester derived olefin (**S27**, 78.3 mg, 0.200 mmol, 1.00 equiv.) and trifluoromethyl thianthrenium triflate (**1**, 191 mg, 0.440 mmol, 2.20 equiv.). The vial was transferred into a N_2-filled glovebox. Subsequently, Cs_2CO_3 (97.7 mg, 0.300 mmol, 1.50 equiv.) was added into the vial. The vial was capped, then it was transferred out of the glovebox. After adding dry 1,4-dioxane (2 mL, c = 0.1 M), the reaction

mixture was stirred at 10 ºC for 1 min, followed by addition of 1,2-benzenedithiol (56.9 mg, 46.0 µL, 0.400 mmol, 2.00 equiv.) with a Hamilton syringe. The reaction mixture was stirred at 10 ºC for 7 h. Subsequently, the reaction mixture was filtered (0.2 µm, PTFE), and the residue was washed with DCM (0.5 mL). The combined solvent was removed under reduced pressure. The residue was purified by column chromatography on silica gel eluting with a solvent mixture of EtOAc:pentane (1:30 (v:v)) to afford 67.7 mg of the title compound (**27**) as slightly yellow solid (90% yield).

R*f* (EtOAc:pentane, 1:6 (v:v)) = 0.24 (UV).

NMR Spectroscopy:

^{1}H NMR (500 MHz, $CDCl_3$, 25 ºC, δ): 7.77 (d, *J* = 7.5 Hz, 2H), 7.62 (t, *J* = 6.2 Hz, 2H), 7.41 (t, *J* = 7.5 Hz, 2H), 7.32 (t, *J* = 7.4 Hz, 2H), 5.36 (d, *J* = 8.5 Hz, 1H), 4.42 – 4.38 (m, 3H), 4.24 (t, *J* = 7.1 Hz, 1H), 4.18 (t, *J* = 5.3 Hz, 2H), 2.19 – 2.04 (m, 2H), 1.90 – 1.83 (m, 1H), 1.79 – 1.57 (m, 5H), 1.45 – 1.21 (m, 4H), 0.92 (t, *J* = 7.1 Hz, 3H).

^{13}C NMR (126 MHz, $CDCl_3$, 25 ºC, δ): 172.8, 156.0, 144.0, 143.9, 141.4, 127.8, 127.1, 127.0 (q, *J* = 276.5 Hz, CF_3), 125.2, 120.1, 120.0, 67.1, 64.6, 54.0, 47.3, 33.3 (q, *J* = 28.8 Hz), 32.4, 27.7, 27.4, 22.4, 18.7 (d, *J* = 2.9 Hz), 13.9.

^{19}F NMR (471 MHz, $CDCl_3$, 25 ºC, δ): –66.34 (t, *J* = 10.8 Hz).

HRMS ESI^{+} (m/z) calc'd for $C_{26}H_{30}N_1O_4F_3Na_1$ $[M+Na]^{+}$, 500.2021; found, 500.2019 deviation: –0.4 ppm.

Rotenone derivative 28

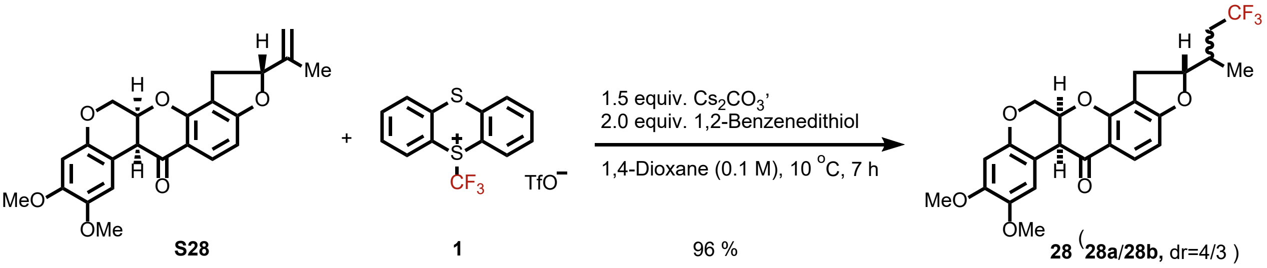

S28 1.0 equiv. + **1** 2.2 equiv. → 96 % **28** (**28a/28b,** dr=4/3)

Under an ambient atmosphere, a 4 mL vial containing a teflon-coated magnetic stirring bar, was charged with rotenone (**S28**, 78.9 mg, 0.200 mmol, 1.00 equiv.) and trifluoromethyl thianthrenium triflate (**1**, 191 mg, 0.440 mmol, 2.20 equiv.). The vial was transferred into a N_2-filled glovebox. Subsequently, Cs_2CO_3 (97.7 mg, 0.300 mmol, 1.50 equiv.) was added into the vial. The vial was capped, then it was transferred out of the glovebox. After adding dry 1,4-dioxane (2 mL, c = 0.1 M), the reaction mixture was stirred at 10 ºC for 1 min, followed by addition of 1,2-benzenedithiol (56.9 mg, 46.0 µL, 0.400 mmol, 2.00 equiv.) with a Hamilton syringe. The reaction mixture was stirred at 10 ºC for 7 h, then filtered (0.2 µm, PTFE), and the residue was washed with DCM (0.5 mL). The combined solvent was removed under reduced pressure. The residue was purified by column chromatography on silica gel eluting with a solvent mixture of EtOAc:pentane (1:15 gradient to 1:5 (v:v)) to afford 89.4 mg of the title compound (**28**) as yellow solid (96% yield, a pair of diastereoisomers **28a** and **28b**), the d.r. value (4/3) was determined by ^{19}F NMR. (Figure S14) Further purification of **28** by preparative HPLC (YMC-Actus Pro C18 (30×150 mm: 5 µm), CH_3CN/H_2O = 48:52, flow rate = 42.5 mL/min, 23 °C) provided **28a** (retention time: 47.6 min) and **28b** (retention time:

49.9 min). **28a** or **28b** cannot be separated under this HPLC method completely. Pure **28a** and **28b** were confirmed by NMR and MS. Attempts have been made to determine the absolute configuration by NMR and growing single crystals, but without success.

R*f* (EtOAc:pentane, 1:2 (v:v)) = 0.56 (UV).

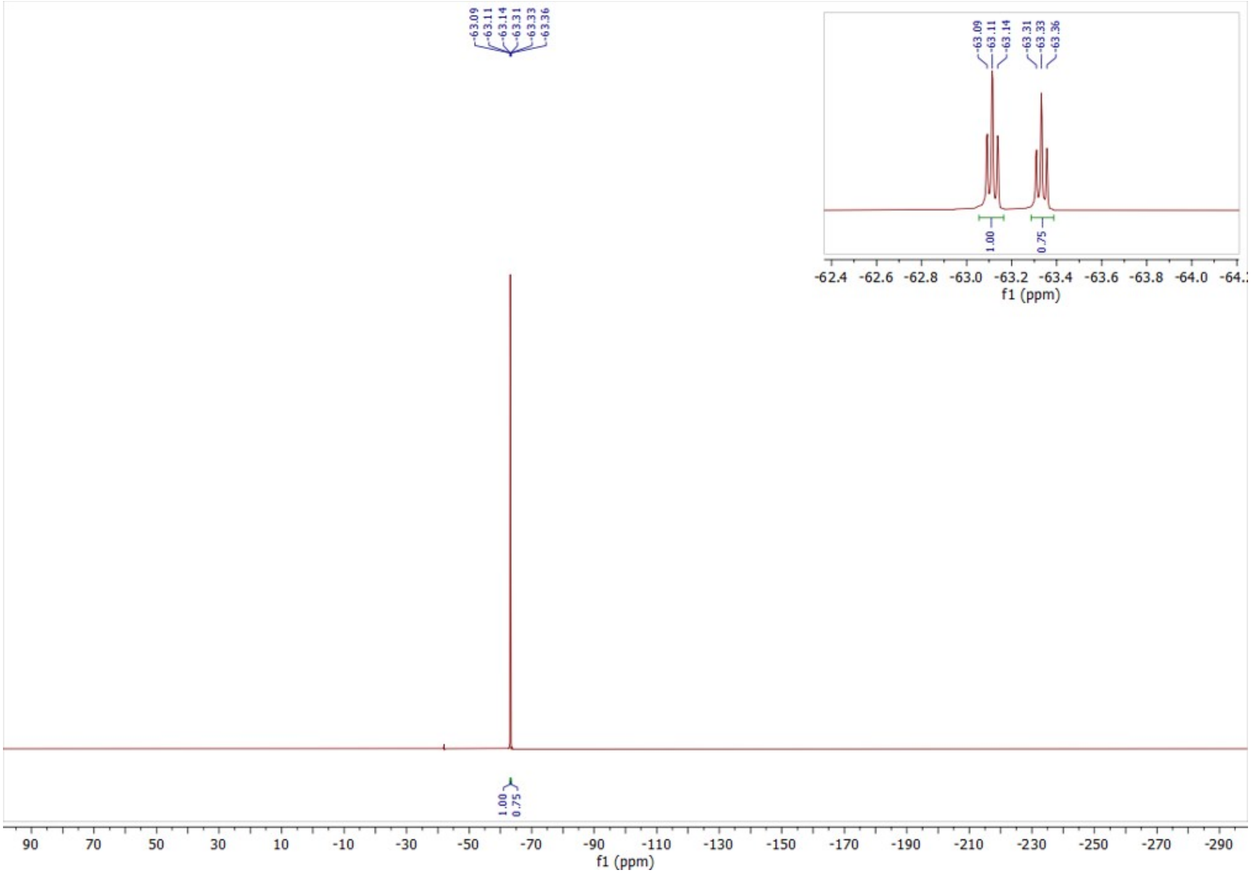

Figure S14. The radio of **28a** and **28b** was determined by ^{19}F NMR spectroscopy

NMR Spectroscopy of 28a:

^{1}H NMR (500 MHz, $CDCl_3$, 25 ºC, δ): 7.83 (dd, *J* = 8.5, 0.7 Hz, 1H), 6.76 (d, *J* = 1.0 Hz, 1H), 6.47 (d, *J* = 8.6 Hz, 1H), 6.45 (s, 1H), 4.94 (ddd, *J* = 4.0, 3.0, 1.1 Hz, 1H), 4.69 – 4.57 (m, 2H), 4.19 (dt, *J* = 12.0, 1.0 Hz, 1H), 3.85 (dt, *J* = 4.1, 0.9 Hz, 1H), 3.81 (s, 3H), 3.76 (s, 3H), 3.26 (dd, *J* = 15.8, 9.3 Hz, 1H), 2.86 (dd, *J* = 15.8, 8.4 Hz, 1H), 2.50 (dd, *J* = 15.0, 3.3 Hz, 1H), 2.19 (dqd, *J* = 10.0, 6.8, 3.2 Hz, 1H), 1.99 (dd, *J* = 15.0, 9.6 Hz, 1H), 1.13 (d, *J* = 6.8 Hz, 3H).

^{13}C NMR (126 MHz, $CDCl_3$, 25 ºC, δ): 189.1, 167.1, 158.1, 149.7, 147.5, 144.0, 130.2, 127.2 (q, *J* = 277.0 Hz, CF_3), 112.9, 110.4, 105.0, 104.9, 101.1, 88.3, 72.4, 66.4, 56.5, 56.0, 44.8, 36.1 (q, *J* = 27.8 Hz), 33.5 (q, *J* = 2.2 Hz), 30.1, 15.6.

^{19}F NMR {1H} (471 MHz, $CDCl_3$, 25 ºC, δ): –63.16.

HRMS of 28a ESI$^+$ (m/z) calc'd for $C_{24}H_{24}O_6F_3$ $[M+H]^+$, 465.1523; found, 465.1520 deviation: –0.8 ppm.

NMR Spectroscopy of 28b:

^{1}H NMR (500 MHz, $CDCl_3$, 25 ºC, δ): 7.83 (d, *J* = 8.5 Hz, 1H), 6.76 (d, *J* = 1.0 Hz, 1H), 6.47 (d, *J* = 8.6 Hz, 1H), 6.45 (s, 1H), 4.94 (ddd, *J* = 4.1, 3.1, 1.2 Hz, 1H), 4.84 (ddd, *J* = 9.7, 8.2, 4.4 Hz, 1H), 4.62

(dd, J = 12.1, 3.1 Hz, 1H), 4.19 (dt, J = 12.1, 1.1 Hz, 1H), 3.85 (dt, J = 4.1, 0.9 Hz, 1H), 3.81 (s, 3H), 3.76 (s, 3H), 3.22 (dd, J = 15.9, 9.7 Hz, 1H), 2.86 (dd, J = 15.9, 8.1 Hz, 1H), 2.32 (dd, J = 14.8, 3.9 Hz, 1H), 2.25 (dddt, J = 11.2, 6.7, 4.4, 2.3 Hz, 1H), 2.06 (dd, J = 14.7, 8.9 Hz, 1H), 1.09 (d, J = 6.7 Hz, 3H).

^{13}C NMR (126 MHz, $CDCl_3$, 25 ºC, δ): 189.0, 167.4, 158.0, 149.7, 147.5, 144.1, 130.3, 127.1 (q, J = 276.9 Hz, CF_3), 113.5, 112.8, 110.5, 104.9, 104.9, 101.1, 87.7, 72.4, 66.4, 56.5, 56.0, 44.7, 36.4 (q, J = 28.0 Hz), 32.7 (q, J = 2.4 Hz), 29.1, 14.2.

^{19}F NMR {1H} (471 MHz, $CDCl_3$, 25 ºC, δ): –63.38.

HRMS of 28b ESI$^+$ (m/z) calc'd for $C_{24}H_{24}O_6F_3$ [M+H]$^+$, 465.1524; found, 465.1520 deviation: –0.9 ppm.

Epiandrosterone derivative 29

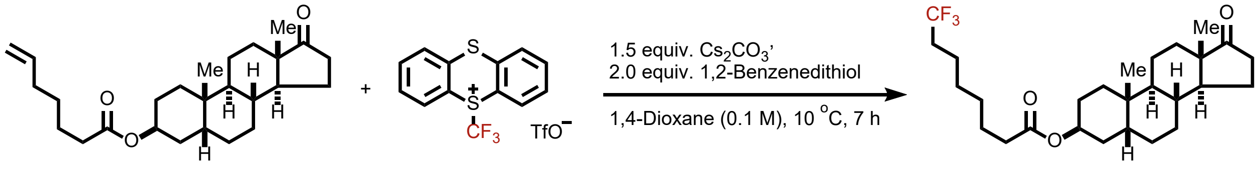

S29 1.0 equiv. | **1** 2.2 equiv. | 94% | **29**

Under an ambient atmosphere, a 4 mL vial containing a teflon-coated magnetic stirring bar, was charged with trifluoromethyl thianthrenium triflate (**1**, 191 mg, 0.440 mmol, 2.20 equiv.). The vial was transferred into a N_2-filled glovebox. Subsequently, Cs_2CO_3 (97.7 mg, 0.300 mmol, 1.50 equiv.) was added into the vial. The vial was capped, then it was transferred out of the glovebox. After adding dry 1,4-dioxane (2 mL, c = 0.1 M), the reaction mixture was stirred at 10 ºC for 1 min, followed by addition of epiandrosterone esterderived olefin (**S29**, 80.1 mg, 0.200 mmol, 1.00 equiv.) and 1,2-benzenedithiol (56.9 mg, 46.0 µL, 0.400 mmol, 2.00 equiv.) with a Hamilton syringe. The reaction mixture was stirred at 10 ºC for 7 h, then filtered (0.2 µm, PTFE), and the residue was washed with DCM (0.5 mL). The combined solvent was removed under reduced pressure. The residue was purified by column chromatography on silica gel eluting with a solvent mixture of EtOAc:pentane (1:40 gradient to 1:13 (v:v)) to afford 88.5 mg of the title compound (**29**) as colorless liquid (94% yield).

R_f (EtOAc:pentane, 1:7 (v:v)) = 0.39 (Vanillin-H_2SO_4).

NMR Spectroscopy:

^{1}H NMR (500 MHz, $CDCl_3$, 25 ºC, δ): 4.68 (tt, J = 11.4, 4.9 Hz, 1H), 2.42 (dd, J = 19.2, 8.5 Hz, 1H), 2.25 (t, J = 7.4 Hz, 2H), 2.11 – 1.98 (m, 3H), 1.91 (ddd, J = 12.1, 8.8, 5.8 Hz, 1H), 1.84 – 1.69 (m, 4H), 1.67 – 1.43 (m, 9H), 1.41 – 1.15 (m, 11H), 1.08 – 0.91 (m, 2H), 0.84 (d, J = 2.7 Hz, 6H), 0.70 (td, J = 12.1, 11.5, 4.0 Hz, 1H).

^{13}C NMR (126 MHz, $CDCl_3$, 25 ºC, δ): 221.2, 173.2, 127.3 (d, J = 276.1 Hz, CF_3), 73.4, 54.4, 51.4, 47.8, 44.7, 36.8, 35.9, 35.7, 35.1, 34.6, 34.06, 33.7 (q, J = 28.4 Hz), 31.6, 30.9, 28.7, 28.4, 28.4, 27.5, 24.8, 21.8, 21.8 (q, J = 2.9 Hz), 20.5, 13.9, 12.3.

^{19}F NMR (471 MHz, $CDCl_3$, 25 ºC, δ): –66.40 (t, J = 10.7 Hz).

HRMS ESI⁺ (m/z) calc'd for $C_{27}H_{41}O_3F_4Na_1$ [M+Na]⁺, 493.2900; found, 493.2900 deviation: –0.1 ppm.

D-Glucose derivative 30

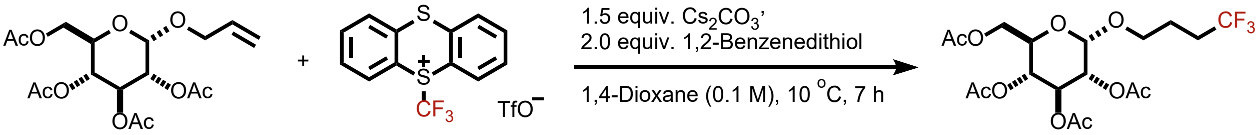

S30 1.0 equiv. **1** 2.2 equiv. 72% **30**

Under an ambient atmosphere, a 4 mL vial containing a teflon-coated magnetic stirring bar, was charged with D-glucose ester (**S30**, 77.7 mg, 0.200 mmol, 1.00 equiv.) and trifluoromethyl thianthrenium triflate (**1**, 191 mg, 0.440 mmol, 2.20 equiv.). The vial was transferred into a N_2-filled glovebox. Subsequently, Cs_2CO_3 (97.7 mg, 0.300 mmol, 1.50 equiv.) was added into the vial. The vial was capped, then it was transferred out of the glovebox. After adding dry 1,4-dioxane (2 mL, c = 0.1 M), the reaction mixture was stirred at 10 °C for 1 min, followed by addition of 1,2-benzenedithiol (56.9 mg, 46.0 µL, 0.400 mmol, 2.00 equiv.) with a Hamilton syringe. The reaction mixture was stirred at 10 °C for 7 h, then filtered (0.2 µm, PTFE), and the residue was washed with DCM (0.5 mL). The combined solvent was removed under reduced pressure. The residue was purified by column chromatography on silica gel eluting with a solvent mixture of EtOAc:pentane (1:15 gradient to 1:5 (v:v)) to afford 66.0 mg of the title compound (**30**) as colorless oil (72% yield).

R_f (EtOAc:pentane, 1:2 (v:v)) = 0.35 ($KMnO_4$).

NMR Spectroscopy:

¹H NMR (300 MHz, $CDCl_3$, 25 °C, δ): 5.44 (t, *J* = 20.0 Hz, 1H), 5.05 (t, *J* = 10.0 Hz, 1H), 5.02 (d, *J* = 20.0 Hz, 1H), 4.85 (dd, *J* = 10.2, 3.8 Hz, 1H), 4.23 (dd, *J* = 12.3, 4.7 Hz, 1H), 4.07 (dd, *J* = 12.3, 2.4 Hz, 1H), 3.97 (ddd, *J* = 10.2, 4.7, 2.3 Hz, 1H), 3.77 (dt, *J* = 11.6, 6.0 Hz, 1H), 3.47 (dt, *J* = 10.0, 6.2 Hz, 1H), 2.29 – 2.14 (m, 2H), 2.07 (s, 2H), 2.04 (s, 2H), 2.02 (s, 2H), 2.00 (s, 2H), 1.87 (dq, *J* = 9.5, 6.2 Hz, 2H).

¹³C NMR (75 MHz, $CDCl_3$, 25 °C, δ): 170.7, 170.2, 170.2, 169.7, 127.2 (q, *J* = 276.2 Hz, CF_3), 96.0, 70.9, 70.19, 68.7, 67.6, 67.0, 62.1, 30.5 (q, *J* = 29.1 Hz), 22.3 (q, *J* = 3.1 Hz), 20.8, 20.8, 20.7, 20.6.

¹⁹F NMR {1H} (282 MHz, $CDCl_3$, 25 °C, δ): –66.14.

HRMS ESI⁺ (m/z) calc'd for $C_{18}H_{25}O_{10}F_3Na_1$ [M+Na]⁺, 481.1292; found, 481.1292 deviation: –0.1 ppm

Quinine derivative 31

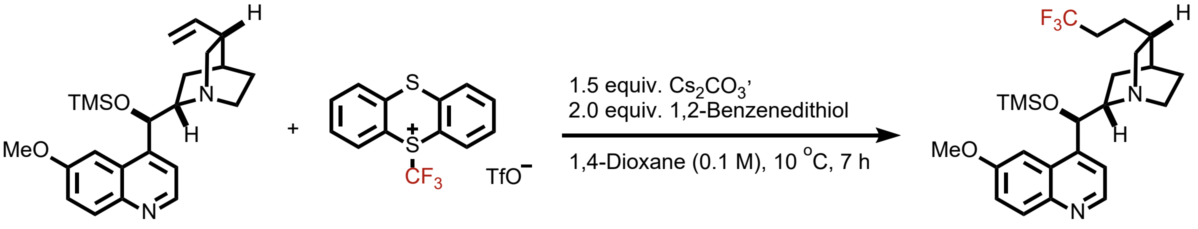

S31 1.0 equiv. **1** 2.2 equiv. 61% **31**

Under an ambient atmosphere, a 4 mL vial containing a teflon-coated magnetic stirring bar, was charged with TMS-quinine (**S31**, 67.7 mg, 0.200 mmol, 1.00 equiv.) and trifluoromethyl thianthrenium triflate (**1**, 191 mg, 0.440 mmol, 2.20 equiv.). The vial was transferred into a N_2-filled glovebox. Subsequently, Cs_2CO_3 (97.7 mg, 0.300 mmol, 1.50 equiv.) was added into the vial. The vial was capped, then it was transferred out of the glovebox. After adding dry 1,4-dioxane (2 mL, c = 0.1 M), the reaction mixture was stirred at 10 ºC for 1 min, followed by addition of 1,2-benzenedithiol (56.9 mg, 46.0 µL, 0.400 mmol, 2.00 equiv.) with a Hamilton syringe. The reaction mixture was stirred at 10 ºC for 7 h, then filtered (0.2 µm, PTFE), and the residue was washed with DCM (0.5 mL). The combined solvent was removed under reduced pressure. The residue was purified by column chromatography on silica gel eluting with a solvent mixture of CH_3OH:CH_3CN (1:40 (v:v)) to afford 60.6 mg of the title compound (**31**) as colorless solid (61% yield).

R_f (CH_3OH:CH_3CN, 1:9 (v:v)) = 0.50 (UV).

NMR Spectroscopy:

^{1}H NMR (600 MHz, CD_3CN, 25 ºC, δ): δ 8.71 (d, *J* = 4.5 Hz, 1H), 7.95 (d, *J* = 9.2 Hz, 1H), 7.79 – 7.74 (m, 1H), 7.51 (d, *J* = 4.5 Hz, 1H), 7.35 (dd, *J* = 9.2, 2.6 Hz, 1H), 6.73 (s, 1H), 4.12 (s, 3H), 3.93 (s, 1H), 3.46 – 3.38 (m, 1H), 3.35 (dd, *J* = 16.7, 7.4 Hz, 1H), 3.07 (s, 1H), 2.75 (d, *J* = 13.4 Hz, 1H), 2.24 – 2.19 (m, 1H), 2.07 – 1.95 (m, 5H), 1.85 – 1.76 (m, 1H), 1.45 (dddd, *J* = 25.9, 13.7, 5.7, 3.2 Hz, 3H), 0.12 (s, 9H).

^{13}C NMR (151 MHz, CD_3CN, 25 ºC, δ): 158.1, 146.1, 143.6, 143.3, 130.7, 126.1 (q, *J* = 276.0 Hz, CF_3), 125.1, 121.9, 118.5, 100.6, 67.1, 59.2, 56.8, 54.7, 42.2, 31.7, 30.2 (q, *J* = 28.7 Hz), 24.6, 23.9, 23.7, 16.9, -0.84 (TMS).

^{19}F NMR {1H} (282 MHz, CD_3CN, 25 ºC, δ): –66.48.

HRMS ESI$^+$ (m/z) calc'd for $C_{24}H_{34}O_2N_2F_3Si_1$ $[M+H]^+$, 467.2332; found, 467.2336 deviation: 0.8 ppm

Adapalene derivative 32

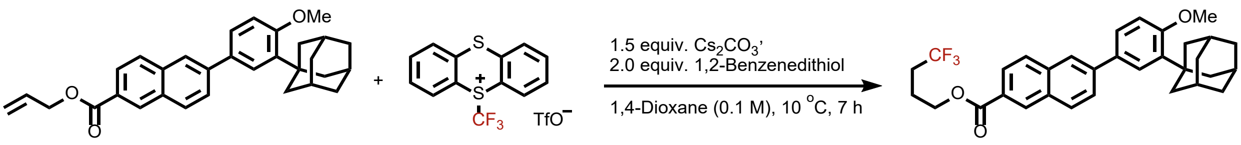

S32 1.0 equiv. | **1** 2.2 equiv. | 67% | **32**

Under an ambient atmosphere, a 4 mL vial containing a teflon-coated magnetic stirring bar, was charged with adapalene allyl ester (**S32**, 90.5 mg, 0.200 mmol, 1.00 equiv.) and trifluoromethyl thianthrenium triflate (**1**, 191 mg, 0.440 mmol, 2.20 equiv.). The vial was transferred into a N_2-filled glovebox. Subsequently, Cs_2CO_3 (97.7 mg, 0.300 mmol, 1.50 equiv.) was added into the vial. The vial was capped, then it was transferred out of the glovebox. After adding dry 1,4-dioxane (2 mL, c = 0.1 M), the reaction mixture was stirred at 10 ºC for 1 min, followed by addition of 1,2-benzenedithiol (56.9 mg, 46.0 µL, 0.400 mmol, 2.00 equiv.) with a Hamilton syringe. The reaction mixture was stirred at 10 ºC for 7 h, then filtered (0.2 µm, PTFE), and the residue was washed with DCM (0.5 mL). To the flask, add silica gel (approximately 500 mg), and concentrated to dryness under reduced pressure. The residue was purified by column chromatography on silica gel eluting with a solvent mixture of EtOAc:pentane (1:50 (v:v)) to afford 70.0 mg of the title compound (**32**) as colorless solid (67% yield).

R$_f$ (EtOAc:pentane, 1:9 (v:v)) = 0.38 (UV).

NMR Spectroscopy:

^{1}H NMR (500 MHz, $CDCl_3$, 25 ºC, δ): 8.60 (s, 1H), 8.06 (d, *J* = 8.6 Hz, 2H), 8.03 (s, 1H), 8.01 (d, *J* = 8.6 Hz, 1H), 7.93 (d, *J* = 8.7 Hz, 1H), 7.82 (d, *J* = 8.6 Hz, 1H), 7.62 (d, *J* = 2.4 Hz, 1H), 7.55 (dd, *J* = 8.5, 2.7 Hz, 1H), 7.01 (d, *J* = 8.3 Hz, 1H), 4.46 (t, *J* = 6.3 Hz, 2H), 3.91 (d, *J* = 1.1 Hz, 3H), 2.37 – 2.31 (m, 2H), 2.19 (s, 6H), 2.14 – 2.10 (m, 5H), 1.81 (s, 6H).

^{13}C NMR (126 MHz, $CDCl_3$, 25 ºC, δ): 166.7, 159.1, 141.7, 139.2, 136.2, 132.6, 131.4, 131.0, 129.9, 128.5, 127.1 (q, *J* = 276.1 Hz, CF_3), 126.7, 126.7, 126.1, 125.9, 125.6, 124.9, 112.3, 63.4, 55.3, 40.8, 37.4, 37.3, 31.1 (q, *J* = 29.3 Hz), 29.3, 21.9 (q, *J* = 3.3 Hz).

^{19}F NMR {1H} (282 MHz, CD_3CN, 25 ºC, δ): –66.32.

HRMS ESI$^+$ (m/z) calc'd for $C_{32}H_{33}O_3F_4Na_1$ $[M+Na]^+$, 545.2282; found, 545.2274 deviation: –1.5 ppm.

Lithocholic acid derivative 33

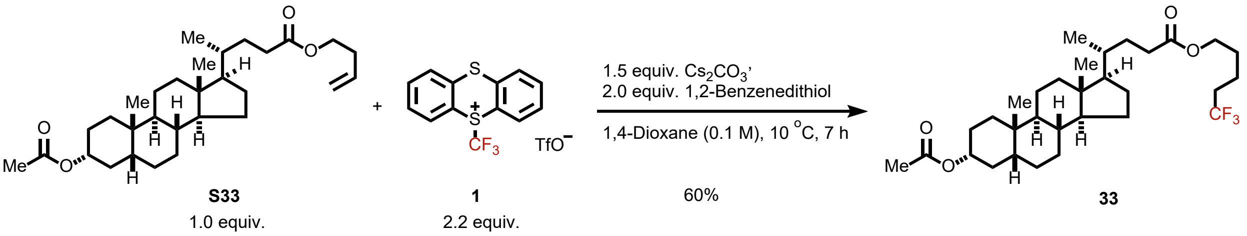

Under an ambient atmosphere, a 4 mL vial containing a teflon-coated magnetic stirring bar, was charged with trifluoromethyl thianthrenium triflate (**1**, 191 mg, 0.440 mmol, 2.20 equiv.). The vial was transferred into a N_2-filled glovebox. Subsequently, Cs_2CO_3 (97.7 mg, 0.300 mmol, 1.50 equiv.) was added into the vial. The vial was capped, then it was transferred out of the glovebox. After adding dry 1,4-dioxane (2 mL, c = 0.1 M), the reaction mixture was stirred at 10 ºC for 1 min, followed by addition of lithocholic acid derived olefin (**S33**, 94.5 mg, 0.200 mmol, 1.00 equiv.) and 1,2-benzenedithiol (56.9 mg, 46.0 μL, 0.400 mmol, 2.00 equiv.) with a Hamilton syringe. The reaction mixture was stirred at 10 ºC for 7 h, then filtered (0.2 μm, PTFE), and the residue was washed with DCM (0.5 mL). To the flask, add silica gel (approximately 500 mg), and concentrated to dryness under reduced pressure. The residue was purified by column chromatography on silica gel eluting with a solvent mixture of EtOAc:pentane (1:40 gradient to 1:20 (v:v)) to afford 67.5 mg of the title compound (**33**) as yellow oil (60% yield).

R$_f$ (EtOAc:pentane, 1:0 (v:v)) = 0.42 (vanillin-H_2SO_4).

NMR Spectroscopy:

^{1}H NMR (300 MHz, $CDCl_3$, 25 ºC, δ): 4.84 – 4.59 (m, 1H), 4.06 (t, *J* = 6.2 Hz, 2H), 2.32 (ddd, *J* = 15.4, 10.0, 5.2 Hz, 1H), 2.20 (ddd, *J* = 15.4, 9.6, 6.6 Hz, 1H), 2.15 – 2.03 (m, 2H), 2.00 (s, 3H), 1.94 (dt, *J* = 12.5, 3.1 Hz, 1H), 1.87 – 1.73 (m, 5H), 1.72 – 1.58 (m, 5H), 1.58 – 1.48 (m, 2H), 1.45 – 1.34 (m, 7H), 1.32 – 1.17 (m, 4H), 1.15 – 0.96 (m, 6H), 0.92 – 0.84 (m, 6H), 0.62 (s, 3H).

^{13}C NMR (75 MHz, $CDCl_3$, 25 ºC, δ): 174.3, 170.7, 127.1 (q, *J* = 276.5 Hz, CF_3), 74.5, 63.5, 56.6, 56.1, 42.8, 42.0, 40.5, 40.3, 35.9, 35.4, 35.1, 34.7, 33.5 (q, *J* = 28.6 Hz), 32.3, 31.3, 31.1, 28.3, 27.8, 27.1, 26.7, 26.4, 24.3, 23.4, 21.5, 20.9, 18.8 (q, *J* = 3.1 Hz), 18.3, 12.1.

^{19}F NMR (282 MHz, $CDCl_3$, 25 °C, δ): –66.43 (t, *J* = 10.8 Hz).

HRMS ESI$^+$ (m/z) calc'd for $C_{31}H_{49}O_4F_3Na_1$ $[M+Na]^+$,565.3480; found, 565.3480 deviation: –0.8 ppm

Diethyl 3-methyl-4-(2,2,2-trifluoroethyl)cyclopentane-1,1-dicarboxylate (34)

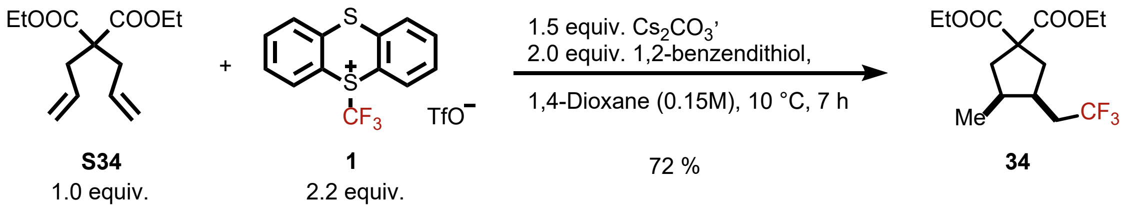

Under an ambient atmosphere, a 4 mL vial containing a teflon-coated magnetic stirring bar, was charged with trifluoromethyl thianthrenium triflate (**1**, 287 mg, 0.660 mmol, 2.20 equiv.). The vial was transferred into a N_2-filled glovebox. Subsequently, Cs_2CO_3 (147 mg, 0.450 mmol, 1.50 equiv.) was added into the vial. The vial was capped, then it was transferred out of the glovebox. After adding dry 1,4-dioxane (2 mL, c = 0.15 M), the reaction mixture was stirred at 10 ºC for 1 min, followed by addition of radical clock reagent (**S34**, 72.1 mg, 0.300 mmol, 1.00 equiv.) and 1,2-benzenedithiol (85.3 mg, 69.1 µL, 0.600 mmol, 3.00 equiv.) with a Hamilton syringe. The reaction mixture was stirred at 10 ºC for 7 h. Subsequently, the reaction mixture was filtered (0.2 µm PTFE), and the residue was washed with DCM (0.5 mL). Silica gel (approximately 600 mg) was added into the combined transparent organic phase. Then the mixture was concentrated to dryness under reduced pressure. The residue was purified by column chromatography on silica gel eluting with a solvent mixture of EtOAc:pentane (pure pentane gradient to 1:50 (v:v)) to afford 66.6 mg of the title compound (**34**) as yellow liquid (72% yield). The d.r. (>20:1) value was determined by ^{1}H NMR. The configuration of the product was determined by comparing with the literature.[7]

R*f* (EtOAc:pentane, 1:9 (v:v)) = 0.42 ($KMnO_4$, very weak).

NMR Spectroscopy:

^{1}H NMR (500 MHz, $CDCl_3$, 25 ºC, δ): 4.19 (q, *J* = 7.1, 2H), 4.18 (q, *J* = 7.1, 2H), 2.49 – 2.42 (m 2H), 2.35 – 2.23 (m, 2H), 2.22 – 2.08 (m, 2H), 2.07 – 1.99 (m, 2H), 1.24 (t, *J* = 7.1, 3H), 1.23 (t, *J* = 7.1, 3H), 0.86 (d, *J* = 6.9 Hz, 3H).

^{13}C NMR (75 MHz, $CDCl_3$, 25 ºC, δ): 172.7, 172.7, 127.3 (q, *J* = 276.8 Hz, CF_3), 61.7, 61.7, 58.8, 41.3, 38.1, 36.6 (q, *J* = 2.2 Hz), 36.1, 34.1 (q, *J* = 28.0 Hz), 15.0, 14.2.

^{19}F NMR {1H} (471 MHz, $CDCl_3$, 25 ºC, δ): –64.63.

HRMS CI Orbitrap (m/z) calc'd for $C_{14}H_{22}O_4F_3$ $[M+H]^+$, 311.1463; found, 311.1465 deviation: 0.5 ppm.

Substrate synthesis

Racecadotril derivative S19

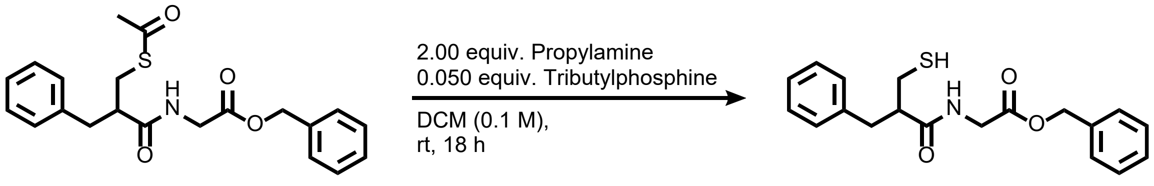

1.00 equiv. 88% **S19**

Under ambient atmosphere, a 10 mL round-bottom flask containing a teflon-coated magnetic stirring bar, was charged with racecadotril (192 mg, 0.500 mmol, 1.00 equiv.), DCM (5 mL, c = 0.1 M), propylamine (59.0 mg, 82.0 µL 1.00 mmol, 2.00 equiv.), and tributylphosphine (5.06 mg, 25.0 µmol, 5.0 mol%). The solution was stirred at room temperature for 18 h. Subsequently, the transparent solution was concentrated to dryness under reduced pressure. The residue was purified by column chromatography on silica gel eluting with a solvent mixture of EtOAc:pentane (1:5 gradient to 1:3 (v:v)) to afford 151 mg of the title compound (**S19**) as colorless liquid (88% yield).

R*f* (EtOAc:pentane, 1:2 (v:v)) = 0.47 (UV).

NMR Spectroscopy:

^{1}H NMR (500 MHz, $CDCl_3$, 25 ºC, δ): 8.65 (s, 1H), 7.39 – 7.30 (m, 4H), 7.30 – 7.24 (m, 2H), 7.23 – 7.14 (m, 3H), 6.28 (t, *J* = 5.0 Hz, 2H), 5.30 – 5.05 (m, 2H), 4.12 (dd, *J* = 18.2, 5.7 Hz, 1H), 3.89 (dd, *J* = 18.2, 5.1 Hz, 1H), 2.98 (dd, *J* = 13.6, 8.0 Hz, 1H), 2.91 – 2.78 (m, 3H), 2.70 – 2.60 (m, 2H), 2.54 (ddd, *J* = 13.8, 9.7, 4.4 Hz, 1H), 1.68 (dd, *J* = 9.6, 7.8 Hz, 1H).

^{13}C NMR (126 MHz, $CDCl_3$, 25 ºC, δ): 173.5, 169.6, 138.6, 135.1, 128.9, 128.6, 128.6, 128.5, 128.4, 126.6, 67.2, 53.0, 41.4, 38.3, 26.2.

HRMS ESI$^+$ (m/z) calc'd for $C_{19}H_{21}N_1O_3S_1Na_1$ $[M+Na]^+$, 366.1138; found, 366.1134 deviation: –1.0 ppm.

N-(4-Chloro-2-fluorophenyl)pent-4-enamide (S25)

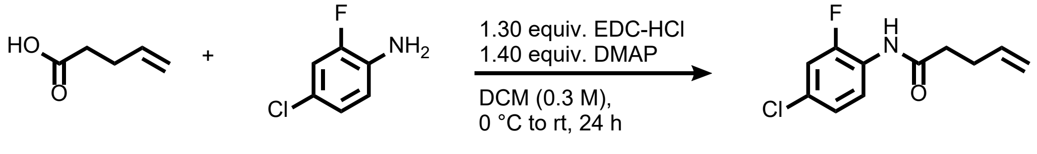

1.00 equiv. 1.20 equiv. 79% **S25**

Under ambient atmosphere, a 50 mL round-bottom flask containing a teflon-coated magnetic stirring bar, was charged with 1-(3-dimethylaminopropyl)-3-ethylcarbodiimide hydrochloride (EDC-HCl, 1.50 g, 7.80 mmol, 1.30 equiv.) and DMAP (1.03 g, 8.4 mmol, 1.40 equiv.). The flask was transferred into a N_2-filled glovebox. Subsequently, dry DCM (20 mL, c = 0.3 M) was added into the flask. The flask was capped, then it was transferred out of the glovebox. After adding 4-pentenoic acid (600 mg, 0.612 mL, 6.00 mmol, 1.00 equiv.), the reaction mixture was stirred at 0 ºC for 5 min, followed by addition of 4-chloro-2-fluoroaniline (1.05 g, 0.799 mL, 7.20 mmol, 1.20 equiv.), the reaction mixture was stirred at room temperature for 24 h. Subsequently hydrochloric acid (20 mL, 10% (v/v)) was added, and the organic layer was separated from the aqueous layer. The aqueous layer was then extracted with DCM (2 × 25 mL). The

combined organic layer was dried over Na_2SO_4. After removing the solvent under reduced pressure, the residue was purified by column chromatography on silica gel eluting with a solvent mixture of EtOAc:pentane (1:20 (v:v)) to afford 1.07 g of the title compound (**S25**) as colorless liquid (79% yield).

R*f* (EtOAc:pentane, 1:4 (v:v)) = 0.50 ($KMnO_4$)

NMR Spectroscopy:

^{1}H NMR (500 MHz, $CDCl_3$, 25 ºC, δ): 8.29 (t, *J* = 8.5 Hz, 1H), 7.36 (br, 1H), 7.13 – 7.09 (m, 2H), 5.94 – 5.82 (m, 1H), 5.13 (dq, *J* = 17.1, 1.6 Hz, 1H), 5.07 (dq, *J* = 10.4, 1.5 Hz, 1H), 2.59 – 2.42 (m, 4H).

^{13}C NMR (126 MHz, $CDCl_3$, 25 ºC, δ): 170.7, 152.1 (d, *J* = 246.3 Hz, CF), 136.6, 128.8 (d, *J* = 9.7 Hz), 125.2 (d, *J* = 10.3 Hz), 124.9 (d, *J* = 3.6 Hz), 122.6, 116.3, 115.7 (d, *J* = 22.8 Hz), 36.9, 29.3.

^{19}F NMR (471 MHz, $CDCl_3$, 25 ºC, δ): –128.69 (t, *J* = 9.8 Hz).

HRMS EI (m/z) calc'd for $C_{11}H_{11}N_1O_1Cl_1F_1$ $[M]^+$, 227.0509; found, 227.0508 deviation: –0.7 ppm.

(1*S*)-10-Camphorsulfonamide (S26)

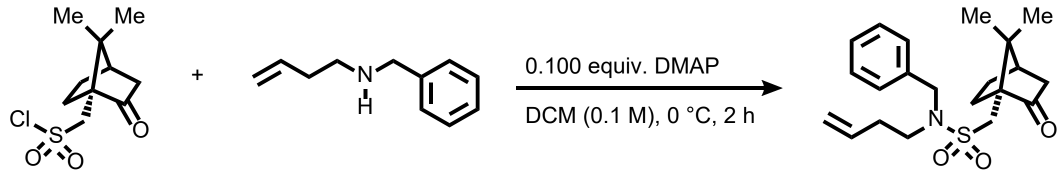

1.00 equiv. 2.00 equiv. 35% **S26**

Under ambient atmosphere, a 25 mL round-bottom flask containing a teflon-coated magnetic stirring bar, was charged with (1*S*)-(+)-10-camphorsulfonyl chloride (250 mg, 1.00 mmol, 1.00 equiv.) and DCM (2 mL). *N*-benzyl-3-butenylamine (322 mg, 2.00 mmol, 2.00 equiv.) and 4-dimethylaminopyridine (DMAP, 12.2 mg, 0.100 mmol, 0.100 equiv.) in DCM (8 mL) was added dropwise while stirring the reaction mixture at 0 ºC. The reaction mixture was allowed to stir for 2 h at 0 ºC. Subsequently, aqueous citric acid solution (c = 10% (w/v), 5 mL) was added at room temperature, extracted with DCM (3 × 10 mL), the combined transparent organic phase was dried over Na_2SO_4. After filtering, silica gel (approximately 1.0 g) was added into this organic phase. Then the mixture was concentrated to dryness under reduced pressure. The residue was purified by column chromatography on silica gel eluting with a solvent mixture of EtOAc:pentane (1:20 gradient to 1:10 (v:v)) to afford 131 mg of the title compound (**S26**) as yellow oil (35% yield).

R*f* (EtOAc:pentane, 1:6 (v:v)) = 0.40 ($KMnO_4$)

NMR Spectroscopy:

^{1}H NMR (300 MHz, $CDCl_3$, 25 ºC, δ): 7.44 – 7.26 (m, 5H), 5.69 (ddt, *J* = 17.6, 9.8, 6.8 Hz, 1H), 5.08 – 4.92 (m, 2H), 4.50 (d, *J* = 15.1 Hz, 1H), 4.37 (d, *J* = 15.1 Hz, 1H), 3.45 – 3.16 (m, 3H), 2.78 (d, *J* = 14.6 Hz, 1H), 2.62 – 2.48 (m, 1H), 2.43 – 2.31 (m, 1H), 2.24 (dtt, *J* = 7.4, 6.0, 1.2 Hz, 2H), 2.14 – 1.97 (m, 2H), 1.93 (d, *J* = 18.4 Hz, 1H), 1.68 (dt, *J* = 9.2, 4.7 Hz, 1H), 1.42 (ddd, *J* = 12.2, 9.4, 4.1 Hz, 1H), 1.14 (s,31H), 0.85 (s, 3H).

^{13}C NMR (75 MHz, $CDCl_3$, 25 ºC, δ): 215.3, 136.5, 134.7, 128.6, 128.5, 127.9, 117.1, 58.5, 51.6, 48.5, 47.8, 47.2, 42.8, 42.6, 33.1, 26.9, 25.3, 20.0, 19.7.

HRMS ESIpos (m/z) calc'd for $C_{21}H_{30}N_1O_3S_1$ $[M+H]^+$, 376.1947; found, 376.1941 deviation: –1.5 ppm.

Fmoc-L-Nle-OH derivative S27

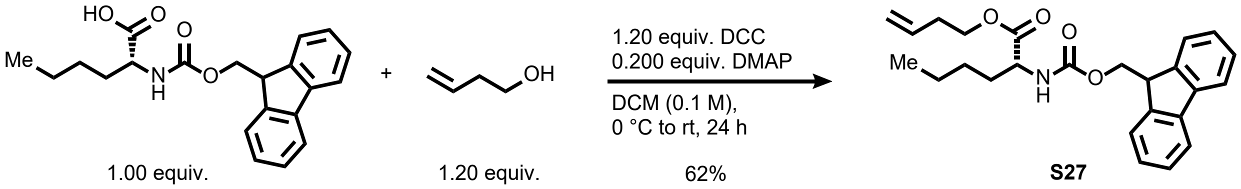

S27

Under ambient atmosphere, a 50 mL round-bottom flask containing a teflon-coated magnetic stirring bar, was charged with Fmoc-L-Nle-OH (706 mg, 2.00 mmol, 1.00 equiv.), allylcarbinol (173 mg, 206 µL, 2.40 mmol, 1.20 equiv.), and dry DCM (20 mL, c = 0.1 M). After stirring at 0 ºC for 2 min, *N,N'*-Dicyclohexylcarbodiimide (DCC, 495 mg, 2.40 mmol, 1.20 equiv.) and DMAP (48.0 mg, 0.400 mmol, 0.200 equiv.) were added into vial. The reaction mixture was stirred at room temperature for 24 h, then filtered. The transparent solution was washed successively with hydrochloric acid (20 mL, 10% (v/v)) and saturated aqueous $NaHCO_3$ solution (10 mL), and then concentrated to dryness. The residue was purified by column chromatography on silica gel eluting with a solvent mixture of EtOAc:pentane (1:15 (v:v)) to afford 504 mg of the title compound (**S27**) as colorless solid (62% yield).

R*f* (EtOAc:pentane, 1:5 (v:v)) = 0.59 ($KMnO_4$)

NMR Spectroscopy:

^{1}H NMR (500 MHz, $CDCl_3$, 25 ºC, δ): 7.77 (d, *J* = 7.5 Hz, 2H), 7.61 (dd, *J* = 7.6, 4.2 Hz, 2H), 7.40 (d, *J* = 7.5 Hz, 2H), 7.32 (t, *J* = 7.5 Hz, 2H), 5.78 (ddt, *J* = 17.1, 10.2, 6.7 Hz, 1H), 5.30 (d, *J* = 8.3 Hz, 1H), 5.19 – 5.00 (m, 2H), 4.41 – 4.37 (m, 3H), 4.29 – 4.14 (m, 3H), 2.42 (q, *J* = 6.7 Hz, 2H), 1.88 – 1.82 (m, 1H), 1.75 – 1.60 (m, 1H), 1.43 – 1.26 (m, 4H), 0.91 (t, *J* = 6.9 Hz, 3H).

^{13}C NMR (126 MHz, $CDCl_3$, 25 ºC, δ): 172.8, 156.0, 144.1, 143.9, 141.4, 133.7, 127.8, 127.2, 125.2, 120.1, 117.7, 67.1, 64.4, 54.0, 47.3, 33.1, 32.6, 27.4, 22.4, 14.0.

HRMS ESI$^+$ (m/z) calc'd for $C_{25}H_{29}N_1O_4Na_1$ $[M+Na]^+$, 430.1994; found, 430.1989 deviation: –1.2 ppm.

Allyl adapalene derivative S32

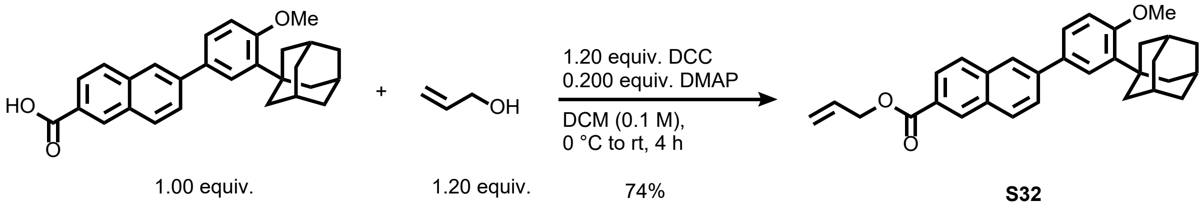

S32

Under ambient atmosphere, a 10 mL round-bottom flask containing a teflon-coated magnetic stirring bar, was charged with adapalene (206 mg, 0.500 mmol, 1.00 equiv.), allylalkohol (34.8 mg, 40.8 µL, 0.60 mmol, 1.20 equiv.), and dry DCM (5 mL, c = 0.1 M). After stirring at 0 ºC for 2 min, the reaction mixture was added *N,N'*-dicyclohexylcarbodiimide (DCC, 124 mg, 0.600 mmol, 1.20 equiv.) and DMAP (12.2 mg, 0.100 mmol, 0.200 equiv.). The reaction mixture was stirred at room temperature for 4 h, then filtered. The transparent solution was washed successively with hydrochloric acid (5 mL, 10% (v/v)) and saturated aqueous $NaHCO_3$ solution (5 mL), and then concentrated to dryness. The residue was purified by column chromatography on

silica gel eluting with a solvent mixture of EtOAc:pentane (1:30 (v:v)) to afford 168 mg of the title compound (**S32**) as colorless solid (74% yield).

R*f* (EtOAc:pentane, 1:6 (v:v)) = 0.69 ($KMnO_4$)

NMR Spectroscopy:

^{1}**H NMR** (500 MHz, $CDCl_3$, 25 ºC, δ): 8.65 (s, 1H), 8.11 (dd, *J* = 8.7, 1.7 Hz, 1H), 8.03 (s, 1H), 8.00 (d, *J* = 8.5 Hz, 1H), 7.93 (d, *J* = 8.6 Hz, 1H), 7.81 (dd, *J* = 8.5, 1.8 Hz, 1H), 7.63 (d, *J* = 2.4 Hz, 1H), 7.55 (dd, *J* = 8.4, 2.4 Hz, 1H), 7.00 (d, *J* = 8.4 Hz, 1H), 6.13 (ddt, *J* = 16.4, 10.8, 5.7 Hz, 1H), 5.49 (dq, *J* = 17.2, 1.7 Hz, 1H), 5.35 (dq, *J* = 10.4, 1.5 Hz, 1H), 4.92 (dt, *J* = 5.9, 1.6 Hz, 2H), 3.91 (s, 3H), 2.22 (s, 6H), 2.13 (s, 3H), 1.83 (s, 6H).

^{13}**C NMR** (126 MHz, $CDCl_3$, 25 ºC, δ): 166.6, 159.1, 141.5, 139.1, 136.1, 132.7, 132.5, 131.4, 131.2, 129.8, 128.4, 127.0, 126.6, 126.1, 125.9, 125.7, 124.8, 118.4, 112.2, 65.8, 55.3, 40.7, 37.3, 37.2, 29.2.

HRMS ESI^{+} (m/z) calc'd for $C_{31}H_{32}O_3Na_1$ $[M+Na]^+$, 475.2247; found, 475.2244 deviation: –0.7 ppm.

Reaction condition optimization for $TTCF_3^+OTf^-$ (1) synthesis

General procedure for $TTCF_3^+OTf^-$ (1) synthesis

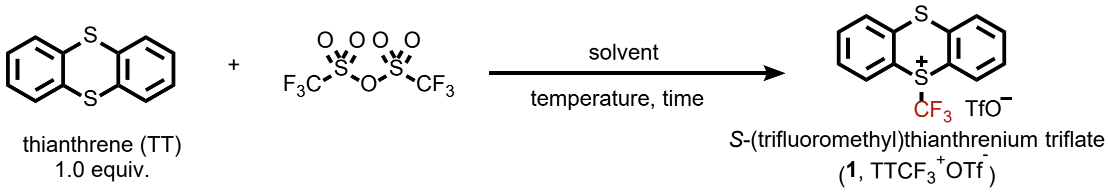

Under an ambient atmosphere, a 20 mL vial containing a teflon-coated magnetic stirring bar, was charged with thianthrene (TT, 1.00 equiv.) and solvent. After adding triflic anhydride, the vial was closed with a teflon-lined screw cap, then the reaction mixture was stirred at a given temperature for a given time. In most cases, the yield was determined by ^{19}F NMR integration relative to the internal standard (1.00 equiv. trifluorotoluene). The isolated yield was determined according to the following procedure: saturated aqueous $NaHCO_3$ solution was added carefully. The mixture was poured into a separatory funnel, and the aqueous layer was discarded. The organic layer was concentrated to dryness under reduced pressure. Subsequently the residue was dispersed in diethyl ether as a suspension under ultrasonication conditions, after centrifugation, the yellow diethyl ether was removed. The above mentioned washing procedure was repeated five times. The resulting yellow slurry was concentrated to dryness as title product (**1**).

Table S1. Evaluation of solvent

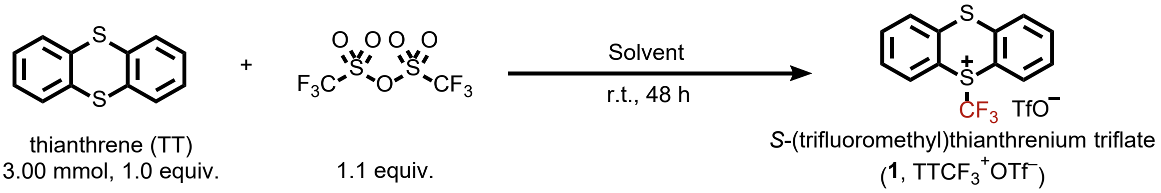

Solvent	Yield/% (^{19}F NMR)	Note
DCM	70[a]	
$CHCl_3$	7	
CH_3CN	trace	Some unknown precipitates
THF	0	The whole system becomes gray waxy solid
$PhCH_3$	0	
DMF	0	Some unknown precipitates
DMSO	0	Some unknown precipitates
EtOAc	0	
Pentane	0	TT was not consumed
Diethyl ether	trace	TT was not consumed

[a]Control experiments showed that yields were within error of measurement if the reaction was carried out using inert conditions.

Table S2. Evaluation of reaction time

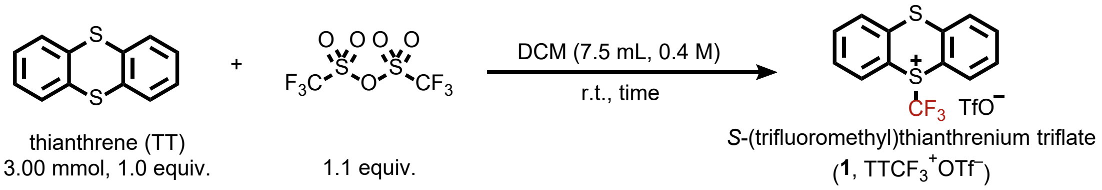

Reaction time/h	Yield/% (^{19}F NMR)
6	56
22	76
29	74

Table S3. Evaluation of concentration

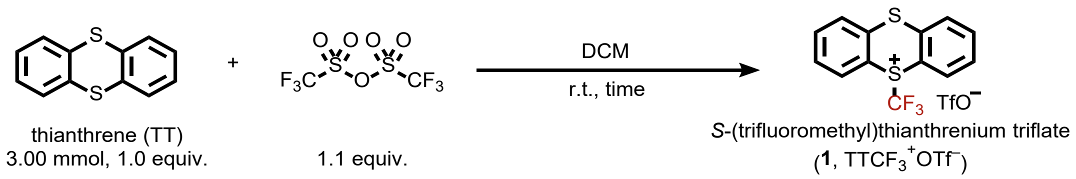

Concentration/molarity	Reaction time/h	Yield/% (isolated)
1.0	23	56[a]
0.7	25	55
0.4	25	65
0.25	22	61

[a]Yield was determined by ^{19}F NMR.

Table S4. Evaluation of additive

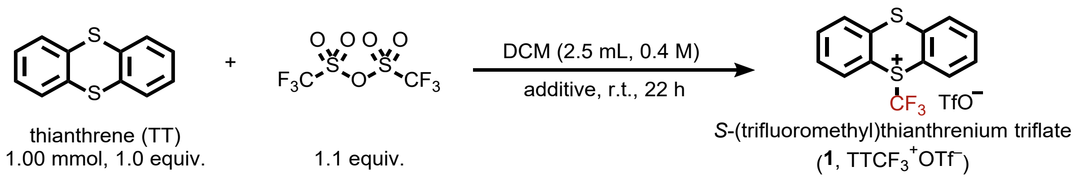

Additive	Yield/% (^{19}F NMR)	Note
none	75	
CuBr (1 mol%)	74	
$(CuOTf)_2$•Toluene (1 mol%)	68	Isolated yield 66%
$Cu(OTf)_2$ (1 mol%)	73	Isolated yield 63%
TfOH[a] (1.0 equiv.)	33	

[a]Reaction condition: thianthrene (TT, 3.00 mmol, 1.0 equiv.), Tf_2O (3.30 mmol, 1.1 equiv.), TfOH (3.00 mmol, 1.0 equiv.), room temperature, 22 h.

Table S5. Evaluation of temperature and reaction time

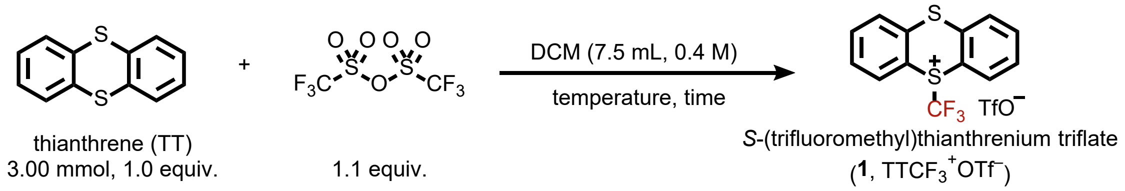

Temperature/ºC	Reaction time/h	Yield/% (^{19}F NMR)	Note
35	22	/	Isolated yield 77%
40	22	81	
40	14	/	Isolated yield 78%

Reaction condition optimization for hydrotrifluoromethylation of olefins

General procedure for hydrotrifluoromethylation of styrene

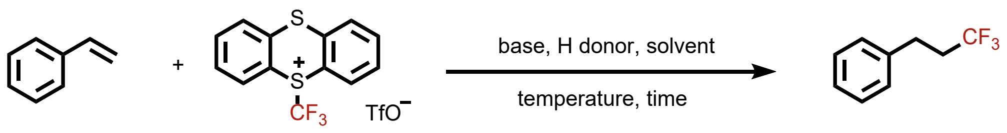

1.0 equiv. **1** **20**

Under an ambient atmosphere, a 4 mL vial containing a teflon-coated magnetic stirring bar, was charged with trifluoromethyl thianthrenium triflate (**1**). The vial was transferred into a N_2-filled glovebox. Subsequently, solid bases and H donors were added into the vial (liquid base was added after adding solvent). The vial was closed with a teflon-lined screw cap and removed from the glovebox. After adding dry solvent and styrene (1.00 equiv.), the reaction mixture was stirred at a given temperature for a given time. The yield was determined by ^{19}F NMR integration relative to the internal standard (1.00 equiv. trifluorotoluene).

Table S6. Preliminary condition screening

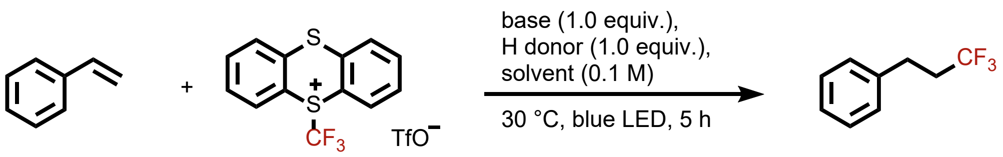

0.1 mmol, 1.0 equiv. **1**, 1.0 equiv. **20**

Base	H donor	Solvent	Yield/% (^{19}F NMR)
K_2CO_3	Et_3SiH	Dioxane	no product
TMEDA	Et_3SiH	CH_3CN	no product
DBU	Et_3SiH	CH_3CN	no product
/	Hantzsch ester	DMF	trace
/	Hantzsch ester	MeOH	no product
Cs_2CO_3[a]	/	THF	2

[a]Reaction temperature is 60 °C without blue LED.

Table S7. Evaluation of H donor

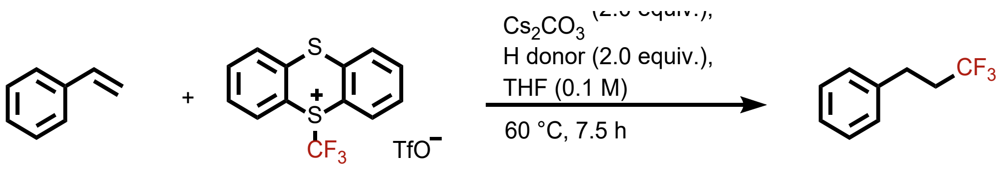

0.05 mmol, 1.0 equiv. **1**, 2.0 equiv. **20**

H donor	Yield/% (^{19}F NMR)
PhSH	29
CF_3CH_2OH	4
Hantzsch ester	7
PhSH[a]	8

[a]Reaction was set up in DCM at 40 ºC, 0.1 mL CF_3CH_2OH as additive.

Table S8. Evaluation of base

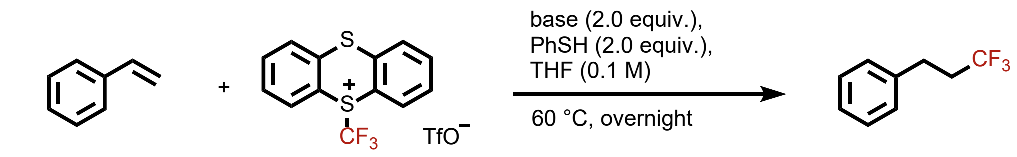

0.05 mmol, 1.0 equiv. **1**, 2.0 equiv. **20**

Base	Yield/% (^{19}F NMR)	Base	Yield/% (^{19}F NMR)
Cs_2CO_3	29	TEA	7
K_2CO_3	22	DBU	3
KOAc	12	DMAP	5
$NaOCH_2CH_3$	19	TMG	2
Rb_2CO_3	trace	NMMO	12
CsOAc	5	DBN	4
$Ca(OH)_2$	5	DABCO	5
CsOPiv	8	TMEDA	9
Na_2CO_3	trace	MTBD	7
NaOAc	no product	BTMG	5
$KOCH_3$	7	TBD	5
NaOH	trace	Cs_2CO_3[a]	no product

[a]PhOH replaced PhSH.

Table S9. Second evaluation of H donor

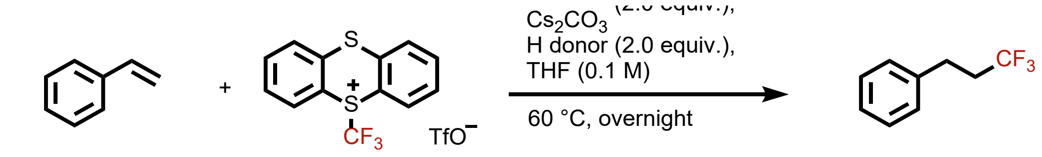

0.05 mmol, 1.0 equiv. **1**, 2.0 equiv. **20**

H donor	Yield/% (^{19}F NMR)	H donor	Yield/% (^{19}F NMR)
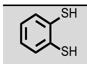	45	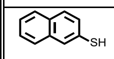	15
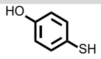	12	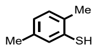	11
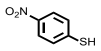	14	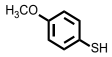	14
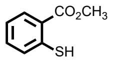	6	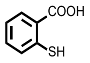	no product
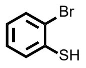	14	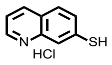	27[a]
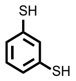	31	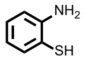	14
Schwartz's reagent	no product	Shvo catalyst	trace

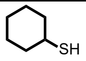	2	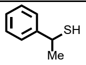	3
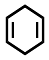	5	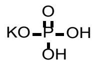	no product
TMS3SiH	3	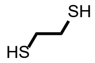	2
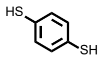	31	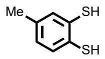	38

[a]The loading of quinoline-7-thiol hydrochloride is 0.05 mmol.

Table S10. Evaluation of solvent and temperature

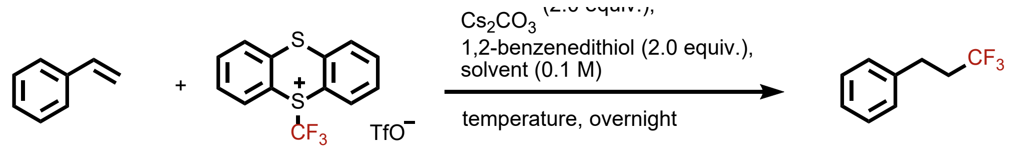

0.05 mmol, 1.0 equiv. **1**, 2.0 equiv. **20**

Solvent	Temperature/ºC	Yield/% (^{19}F NMR)
1,4-Dioxane	100	27
DCM	40	40
EtOH	80	41
CH_3CN	80	28
THF	80	34
THF	60	45
THF	40	50
THF	30	61
THF	10	31
THF	0	73
THF	–10	65
THF	–20	26
DCM	0	51
EtOH	0	57
CF3CH2OH	0	12
HFIP	0	no product
i-Propanol	0	70
n-Propanol	0	86
s-Butanol	0	45
n-Butanol	0	83
i-Butanol	0	91
i-Butanol	10	32
i-Butanol	–10	71

Table S11. Evaluation of reagents' amount

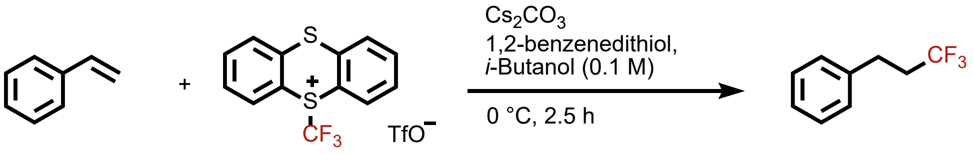

0.05 mmol, 1.0 equiv. **1** **20**

$TTCF_3^+OTf^-$/equiv. (**1**)	Cs_2CO_3/equiv.	1,2-Benzenedithiol/equiv.	Yield/% (^{19}F NMR)
2.0	2.0	2.0	90
1.5	2.0	2.0	88
2.0	1.5	2.0	71
2.0	2.0	1.5	81
1.2	2.0	2.0	76
1.5	1.8	1.8	83
1.5	2.0	2.0	87[a]

[a]Reaction time 30 min

Table S12. Evaluation of counterions

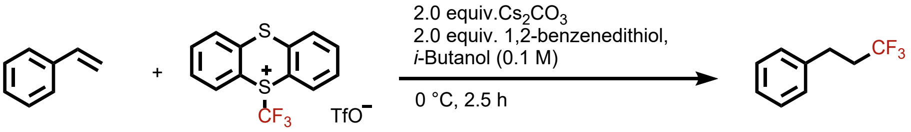

0.05 mmol, 1.0 equiv. 2.0 equiv. 90 %

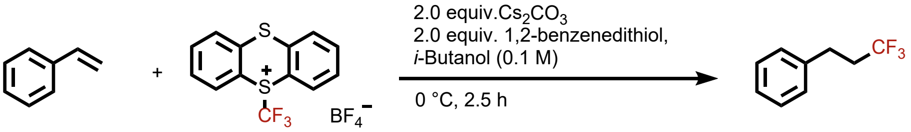

0.05 mmol, 1.0 equiv. 2.0 equiv. 92 %

General procedure for hydrotrifluoromethylation of aliphatic olefin

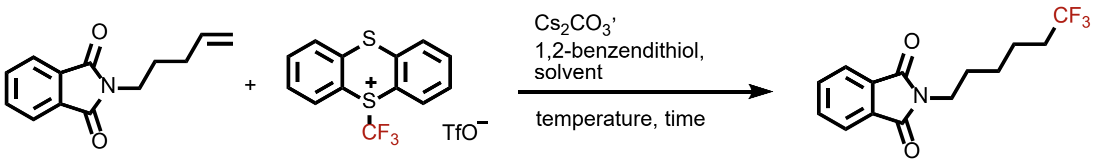

1.0 equiv. **1** **23**

Under an ambient atmosphere, a 4 mL vial containing a teflon-coated magnetic stirring bar was charged with phthalimide derived olefin (1.00 equiv.) and trifluoromethyl thianthrenium triflate (**1**). The vial was transferred into a N_2-filled glovebox. Subsequently, Cs_2CO_3 was added into the vial. The vial was closed with a teflon-lined screw cap and removed from the glovebox. After adding dry solvent and 1,2-benzenedithiol, the reaction mixture was stirred at a given temperature for a given time. The yield was determined by ^{19}F NMR integration relative to the internal standard (1.00 equiv. trifluorotoluene).

Table S13. Evaluation of solvent

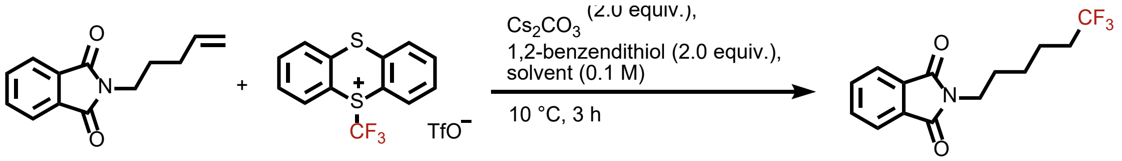

Solvent	Yield/% (^{19}F NMR)
DCM	41
THF	43
1,4-Dioxane	81
EtOH	27
i-Propanol	28
i-Butanol	46
2-Methyl-1-butanol	23
n-Butanol	50
n-Propanol	50

Table S14. Evaluation of reagents' amount

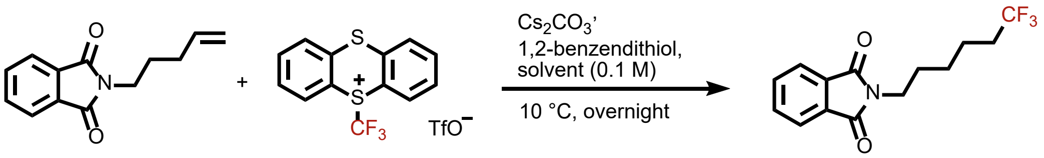

$TTCF_3^+OTf^-$/equiv. (**1**)	Cs_2CO_3/equiv.	1,2-Benzenedithiol/equiv.	Yield/% (^{19}F NMR)
2.0	1.5	2.0	90
2.0	2.0	1.5	71
2.2	1.5	2.0	94[a]
2.2	1.2	2.0	87[a]

[a]Reaction time 5h.

Reaction condition optimization for trifluoromethylation of thiols

General procedure for trifluoromethylation of thiophenol

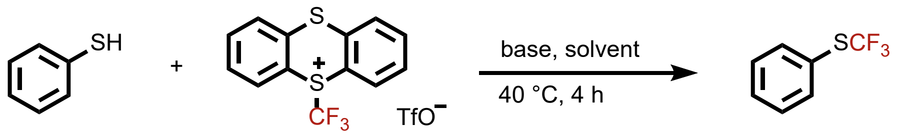

1.0 equiv. **1** **15**

Under an ambient atmosphere, a 4 mL vial containing a teflon-coated magnetic stirring bar was charged with trifluoromethyl thianthrenium triflate (**1**). The vial was transferred into a N_2-filled glovebox. Subsequently, solid base was added into the vial (liquid base was added after adding solvent). The vial was closed with a teflon-lined screw cap and removed from the glovebox. After adding dry solvent and thiophenol (1.00 equiv.), the reaction mixture was stirred at 40 ºC for 4 h. The yield was determined by ^{19}F NMR integration relative to the internal standard (1.00 equiv. trifluorotoluene).

Table S15. Preliminary condition screening

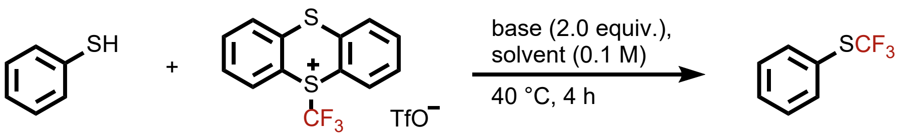

0.05 mmol,1.0 equiv. **1**, 2.0 equiv. **15**

Base	Solvent	Yield/% (^{19}F NMR)
Et_3N	MeOH	79
Et_3N	DMF	87
DBU	THF	43
Cs_2CO_3	DCM	40
Cs_2CO_3	CH_3CN	68

Table S16. Evaluation of reagents' amount

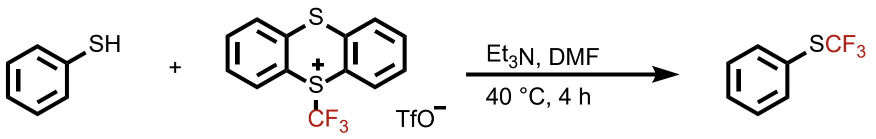

0.05 mmol,1.0 equiv. **1** **15**

$TTCF_3^+OTf^-$/equiv. (**1**)	Et_3N/equiv.	Yield/% (^{19}F NMR)
2.0	2.0	87
1.5	2.0	71
2.0	1.5	81
1.8	1.8	51

General procedure for trifluoromethylation of aliphatic thiol

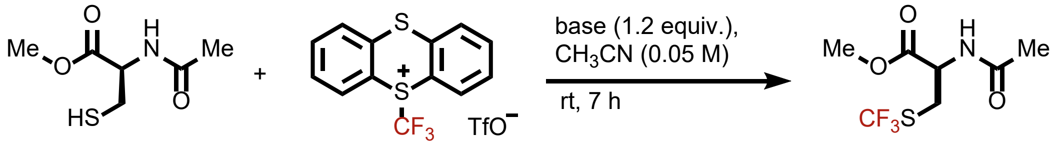

1.0 equiv. **1**, 1.2 equiv. **18**

Under an ambient atmosphere, a 4 mL vial containing a teflon-coated magnetic stirring bar was charged with *N*-Acetyl-L-cysteine methyl ester (1.00 equiv.) and trifluoromethyl thianthrenium triflate (**1**). The vial was transferred into a N_2-filled glovebox. Subsequently, solid base was added into the vial (liquid base was added after adding solvent). The vial was closed with a teflon-lined screw cap and removed from the glovebox. After adding dry CH_3CN, the reaction mixture was stirred at room temperature for 7 h. The yield was determined by ^{19}F NMR integration relative to the internal standard (1.00 equiv. trifluorotoluene).

Table S17. Evaluation of base

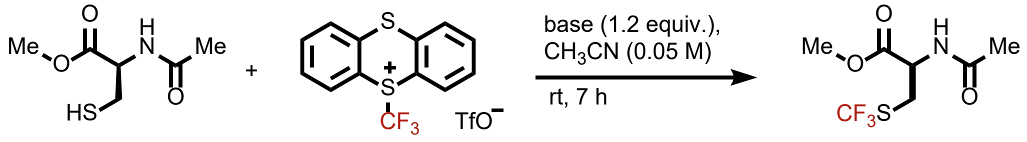

0.05 mmol, 1.0 equiv. **1**, 1.2 equiv. **18**

Base	Yield/% (^{19}F NMR)
DMAP	41
$KHCO_3$	10
Cs_2CO_3	61
pyridine	no product
DBU	61
TMG	94
TMG	75[a]
TMG	94[b]

[a]$TTCF_3^+OTf^-$ 1.1 equiv. and TMG 1.1 equiv.. [b]Reaction time 4 h.

Reaction condition optimization for trifluoromethylation of aryl boronic acid

General procedure for trifluoromethylation of aryl boronic acid

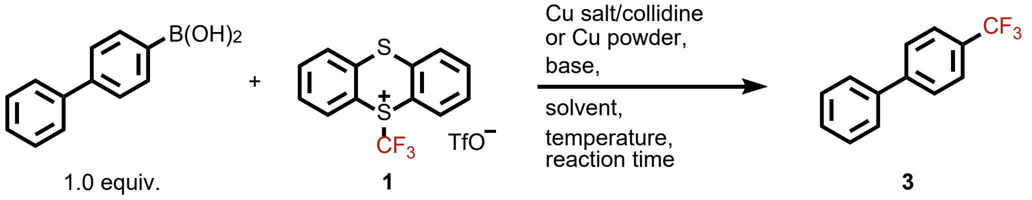

Under an ambient atmosphere, a 4 mL vial containing a teflon-coated magnetic stirring bar was charged with 4-biphenylboronic acid (1.00 equiv.), trifluoromethyl thianthrenium triflate (**1**), Cu salt (or Cu powder), and base. The vial was transferred into a N_2-filled glovebox. Subsequently, dry solvent was added into the vial. The vial was closed with a teflon-lined screw cap and removed from the glovebox. After adding dry 2,4,6-collidine (Cu powder doesn't need 2,4,6-collidine as ligand), the reaction mixture was stirred at a given temperature for a given time. The yield was determined by ^{19}F NMR integration relative to the internal standard (1.00 equiv. trifluorotoluene).

Table S18. Preliminary condition screening

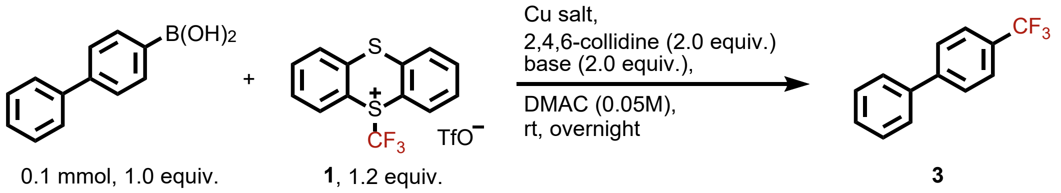

Cu salt	Amount of Cu salt	Base	Yield/% (^{19}F NMR)
CuCl	20 mol%	Cs_2CO_3	trace
CuO_2	20 mol%	Cs_2CO_3	no product
$(CuOTf)_2$-tuluene	20 mol%	Cs_2CO_3	1
TcCu	20 mol%	Cs_2CO_3	7
Cu(OAc)	20 mol%	Cs_2CO_3	2
Cu(OAc)	20 mol%	NaOAc	no product
TcCu	20 mol%	Cs_2CO_3	7[a]
Cu(OAc)	20 mol%	Cs_2CO_3	11[a]
Cu(OAc)	approximately 0.5 equiv.	Cs_2CO_3	55[b]
Cu(OAc)	approximately 0.5 equiv.	Cs_2CO_3	47[bc]

[a]Amount of $TTCF_3^+OTf^-$ is 2.0 equiv.. [b]Amount of 4-biphenylboronic acid is 0.05 mmol. [c]Solvent is ethyl ester.

Table S19. Evaluation of base

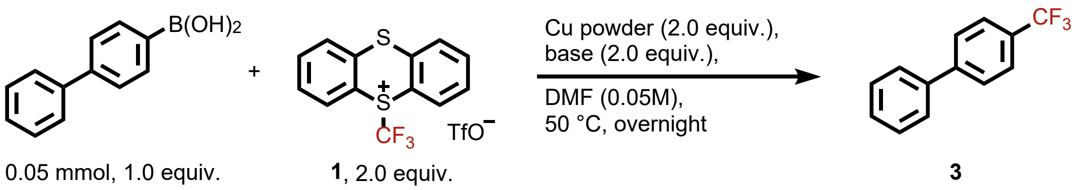

Base	Yield/% (^{19}F NMR)
K_2CO_3	trace
KOAc	1
Na_2CO_3	2
NaOAc	15
EtONa	3
Li_2CO_3	49
$KHCO_3$	14
LiOAc	15
Rb_2CO_3	no product

Table S20. Evaluation of solvent

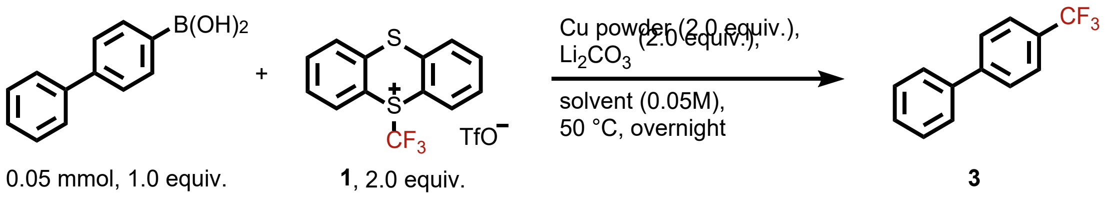

Solvent	Yield/% (^{19}F NMR)
DMF	51
CH_3CN	29
DCM	no product[a]
DMSO	60
THF	no product
Dioxane	2
DMAc	Trace
NMP	23

[a]Reaction temperature is 40 °C.

Table S21. Evaluation of base and temperature

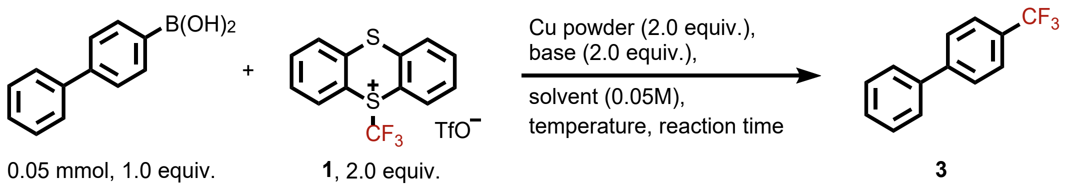

Base	Solvent	Temperature/°C	Reaction time/h	Yield/% (^{19}F NMR)
Li_2CO_3	DMSO	50	overnight	60
Li_2CO_3	DMSO	30	overnight	43
$NaHCO_3$	DMSO	30	overnight	70
$NaHCO_3$	DMSO	40	18	81
$NaHCO_3$	DMSO	50	17	66
$NaHCO_3$	DMSO	60	17	63

$NaHCO_3$	DMSO	35	overnight	83
$NaHCO_3$	DMSO	45	overnight	83

Table S22. Evaluation of reagents' amount

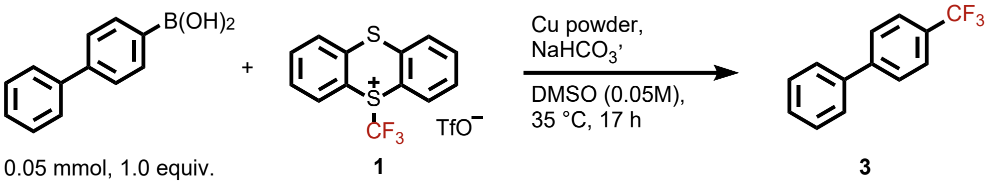

$TTCF_3^+OTf^-$/equiv. (**1**)	Cu powder/equiv.	$NaHCO_3$/equiv.	Yield/% (^{19}F NMR)
1.5	2.0	2.0	87
2.0	1.5	2.0	81
2.0	2.0	1.5	63
2.0	2.5	2.0	28
1.5	1.5	2.0	27
2.0	1.2	2.0	90

Reaction condition optimization for nucleophilic trifluoromethylation of aryl aldehyde

General procedure for nucleophilic trifluoromethylation of aryl aldehyde

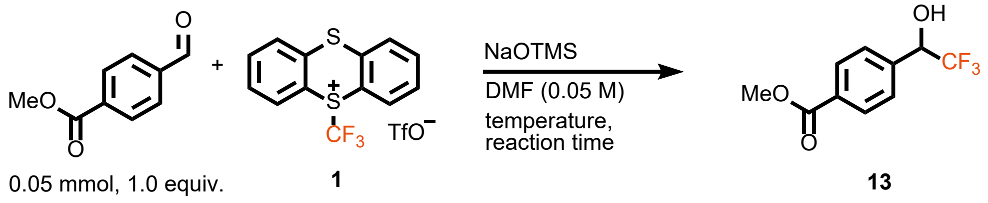

Under an ambient atmosphere, a 4 mL vial containing a teflon-coated magnetic stirring bar was charged with methyl 4-formylbenzoate (0.05 mmol, 8.20 mg, 1.00 equiv.), trifluoromethyl thianthrenium triflate (**1**), and NaOTMS. The vial was transferred into a N_2-filled glovebox. The vial was closed with a teflon-lined screw cap and removed from the glovebox. After adding cool dry DMF (1 mL, c = 0.05 M), the reaction mixture was stirred at a given temperature for a given time. The yield was determined by ^{19}F NMR integration relative to the internal standard (1.00 equiv. trifluorotoluene).

Table S23. Evaluation of reaction temperature and reagents' amount

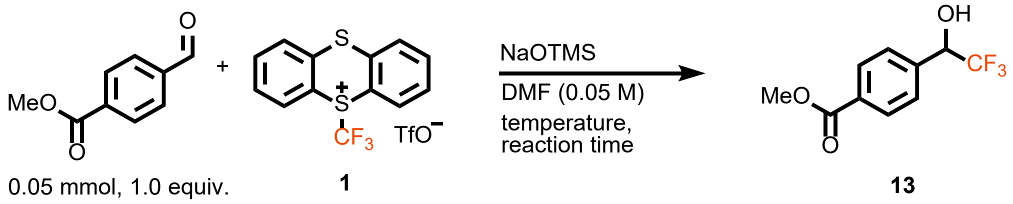

$TTCF_3^+OTf^-$ (**1**)/equiv.	NaOTMS/equiv.	Temperature/ºC	Reaction time/h	Yield/% (^{19}F NMR)
3.00	3.00	0	6	24
3.00	3.00	–15	18	32
3.00	3.00	–20	23	85
3.00	3.00	–25	19	67
3.00	3.00	–35	15	59
3.00	3.00	–40	4	35
3.00	2.00	–20	19	47
2.00	3.00	–20	19	25
3.00	3.00[a]	–20	20	< 5%

[a]KOTMS instead of NaOTMS.

Reaction condition optimization for radical trifluoromethylation of heterocycles

General procedure for radical trifluoromethylation of caffeine

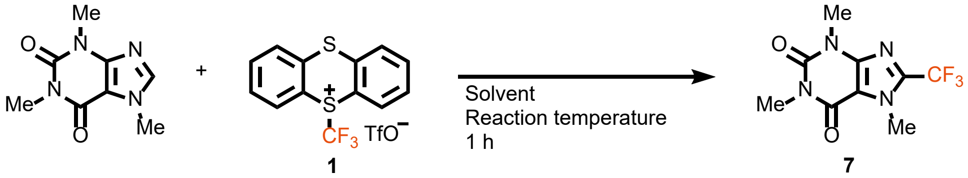

Under an ambient atmosphere, to a 2 mL vial containing a teflon-coated magnetic stirring bar was charged with caffeine (0.15 mmol, 29.4 mg, 3.00 equiv.) and trifluoromethyl thianthrenium triflate (**1**, 0.05 mmol, 21.7 mg, 1.00 equiv.). The vial was transferred into a N_2-filled glovebox. The vial was closed with a teflon-lined screw cap and removed from the glovebox. After adding dry solvent (1 mL, c = 0.05 M), the reaction mixture was stirred under a given condition for 1h. The yield was determined by ^{19}F NMR integration relative to the internal standard (1.00 equiv. trifluorotoluene).

Table S24. Evaluation of solvent and radical activation mode

Base.	Solvent	Radical activation mode	Yield/% (^{19}F NMR)
Cs_2CO_3 (2.00 equiv.)	DMSO	80 °C	No product
Cs_2CO_3 (2.00 equiv.)	DMF	80 °C	No product
Cs_2CO_3 (2.00 equiv.)	DMSO	Blue LED	No product
/	DMSO	80 °C	No starting materials consumed
/	DMSO	Blue LED	41
/	Toluene	Blue LED	No product
/	CH_3CN	Blue LED	53
	CH_3CN	Blue LED	39[a]
/	DCM	Blue LED	43
/	THF	Blue LED	No product
/	$CHCl_3$	Blue LED	53
/	DCE	Blue LED	50
/	DMAc	Blue LED	3
/	CH_3CN	Purple LED	45

[a]2.0 equiv. caffeine was used.

Elemental analysis

	Carbon / %		Hydrogen / %	
	theoretical value	measured value	theoretical value	measured value
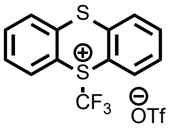 $C_{14}H_8F_6O_3S_3$	38.71	38.67	1.86	1.88
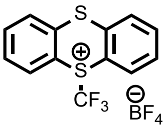 $C_{13}H_8BF_7S_2$	41.96	41.95	2.17	2.16

Formal reports

Hao Jin

Max-Planck-Institut für Kohlenforschung
Prof. Dr. Tobias Ritter
Organische Synthese
D-45470 Mülheim an der Ruhr

Anschrift : **Osterfelder Str. 3**
D-46047 Oberhausen
Telefon : **+49 - (0)208 - 32502**
Telefax : **+49 - (0)208 - 382314**
Email : **info@mikro-lab.de**
Webseite : **www.mikro-lab.de**

Datum : **12.04.2021**

Probenbezeichnung	% C	% H										V2O5
JII - JA - 069 - 02 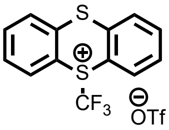	38,67	1,88										x

Mit freundlichen Grüssen

Patrick Springer

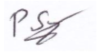

Hao Jin

Max-Planck-Institut für Kohlenforschung
Prof. Dr. Tobias Ritter
Organische Synthese
D-45470 Mülheim an der Ruhr

Anschrift : **Osterfelder Str. 3**
D-46047 Oberhausen
Telefon : **+49 - (0)208 - 32502**
Telefax : **+49 - (0)208 - 382314**
Email : **info@mikro-lab.de**
Webseite : **www.mikro-lab.de**

Datum : **12.04.2021**

**Probenbezeichnung**	**% C**	**% H**										**V2O5**
JII - JA - 069 - 03 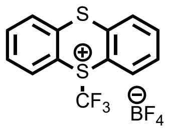	41,95	2,16										x

Mit freundlichen Grüssen

Patrick Springer

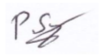

Simultaneous thermal analysis (DSC-TGA)

Simultaneous Thermal Analysis (STA) refers to the simultaneous application of Thermogravimetry (TGA) and Differential Scanning Calorimetry (DSC) to one and the same sample in a single instrument. Here, we measured three different sulfonium CF_3 reagents: $TTCF_3^+OTf^–$, *Umemoto*'s reagent ($OTf^–$ as counterion, CAS registry number 129946-88-9), and $Ph_2S^+CF_3OTf^–$ (CAS registry number 147531-11-1) under the same condition.

Measurement details

A blank group and three experimental groups were tested under Ar purge. The whole process included heating and cooling regions. The blank group and measurement groups used aluminum crucibles with pierced lids (open crucibles) as a container.

Ar gas flow velocity: 50 mL/min

Heating region: from 25 ºC to 300 ºC

Heating rate: 2 K/min

Cooling region: from 300 ºC to 25 ºC

$Ph_2S^+CF_3OTf^–$ data

$Ph_2S^+CF_3OTf^–$ (brown powder, 5.1960 mg, 0.012851 mmol) was placed in an Al-crucible for the STA measurement. The first endothermic peak from 68 ºC to 86 ºC is a reflection of the melting process (Figure S15, bottom). The second endothermic peak from 168 ºC to 288 ºC reflects a decomposition process, due to sample weight loss (Figure S15, top). During the whole decomposition process, the sample loses 95.6626% mass, corresponding to 4.9706 mg. ΔH_D = –121.9 J/g.

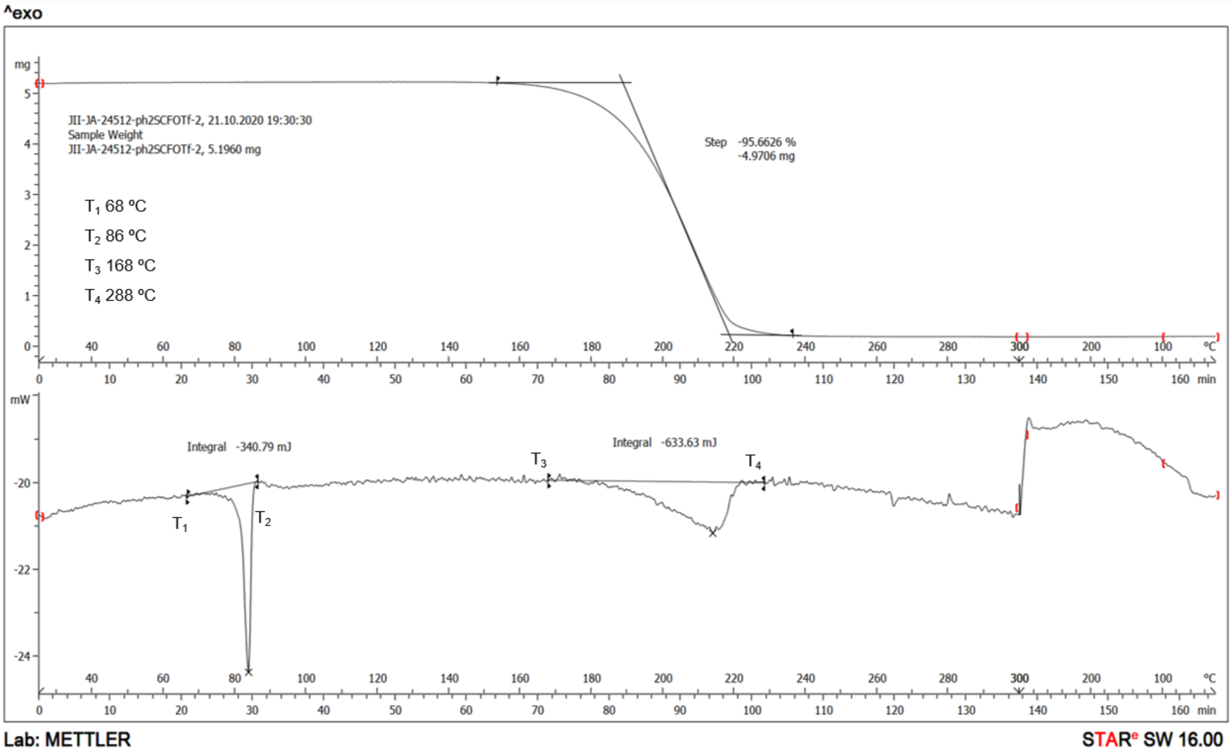

Figure S15. Simultaneous thermal analysis of $Ph_2S^+CF_3OTf^–$.

***Umemoto*'s reagent data**

Umemoto's reagent (crystal, 5.4290 mg, 0.013494 mmol) was placed in an Al-crucible for the STA measurement. Decomposition of *Umemoto*'s reagent is a two-step process. At 128 °C, melting starts, which corresponds to the first endothermic peak in the DSC curve (Figure S16, bottom), after that, the sample has an exothermic peak which ends at 175 °C. The enthalpy change of the first step is +2.6 J/g. Then the sample continues to decompose regarded as the second decomposition step corresponding to the second endothermic peak shown in Figure S16. The enthalpy change of the second step is –154.8 J/g. Thus, the enthalpy change for the whole decomposition process of *Umemoto*' reagent is –152.2 J/g, which is an endothermic process.

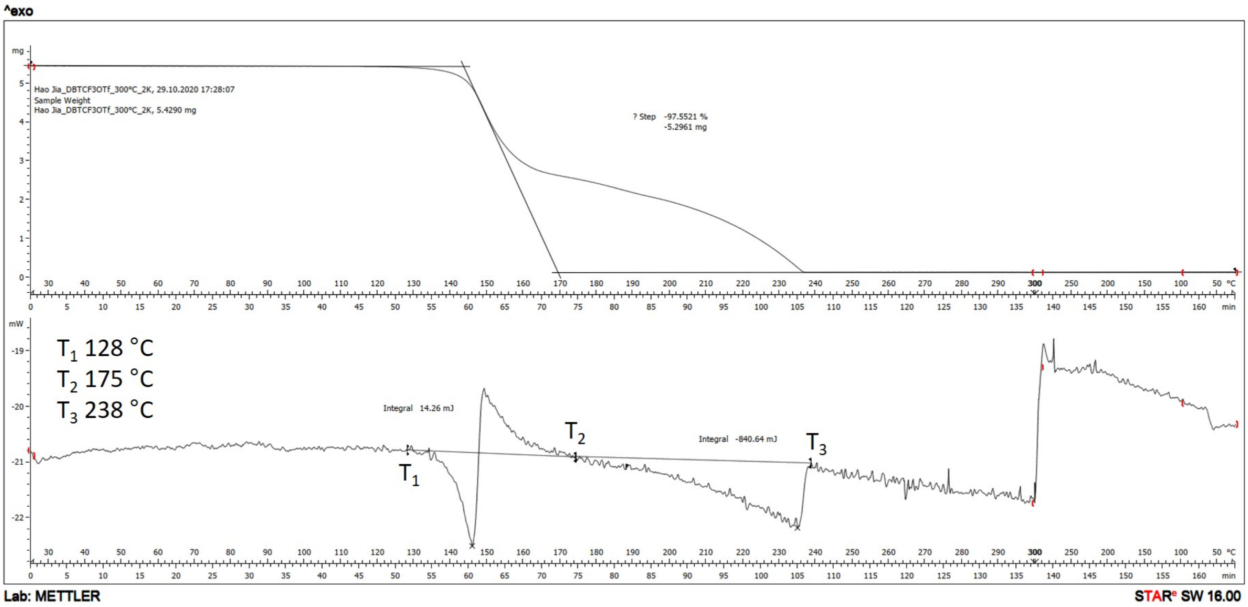

Figure S16. Simultaneous thermal analysis of *Umemoto*'s reagent.

$TTCF_3^+OTf^–$ (1) data

$TTCF_3^+OTf^–$ (**1**, crystal, 5.3360 mg, 0.012284 mmol) was placed in an Al-crucible for the STA measurement. Decomposition of $TTCF_3^+OTf^–$ is a two-step process. At 142 °C, melting begins, which corresponds to the first endothermic peak in the DSC curve (Figure S17, bottom), after that, the sample has two exothermic transitions. This represents a two-step decomposition process, which ends at 190 °C. The enthalpy change for the whole decomposition process of $TTCF_3^+OTf^–$ is +80.6 J/g, which is an exothermic process.

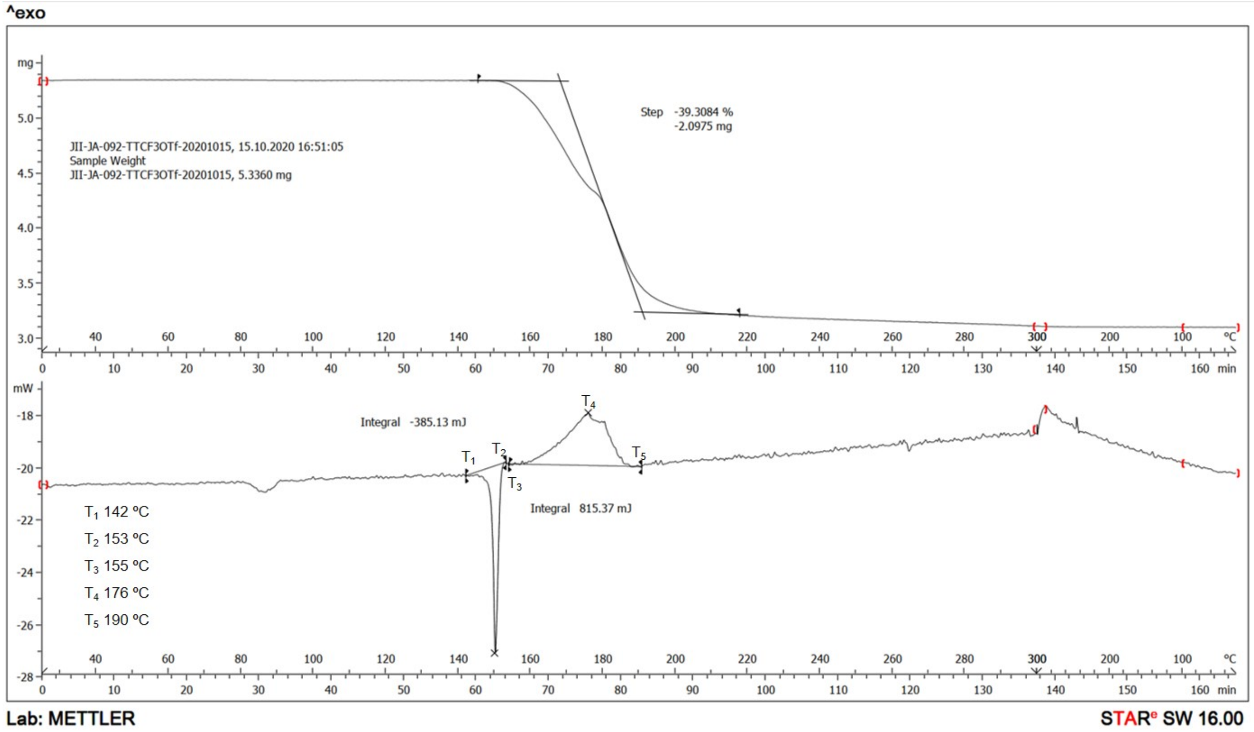

Figure S17. Simultaneous thermal analysis of $TTCF_3^+OTf^–$.

Cyclic voltammograms of $TTCF_3^+OTf^-$ (1)

Cyclic voltammograms were recorded using an Autolab PGSTAT204 potentiostat and a carbon working electrode, a Ag/AgCl reference electrode and a Pt auxiliary electrode. The voltammograms were recorded at room temperature in 0.1 M tetrabutylammonium hexafluorophosphate with CH_3CN (3 mL) containing $TTCF_3^+OTf^-$ (**1**, 13.0 mg, 0.030 mmol). The scan rate was 100 mV s^{-1}. The redox potential of ferrocene was also measured under this standard condition to determine the redox potential *vs*. $Fc^{+/0}$. (Figure S18)

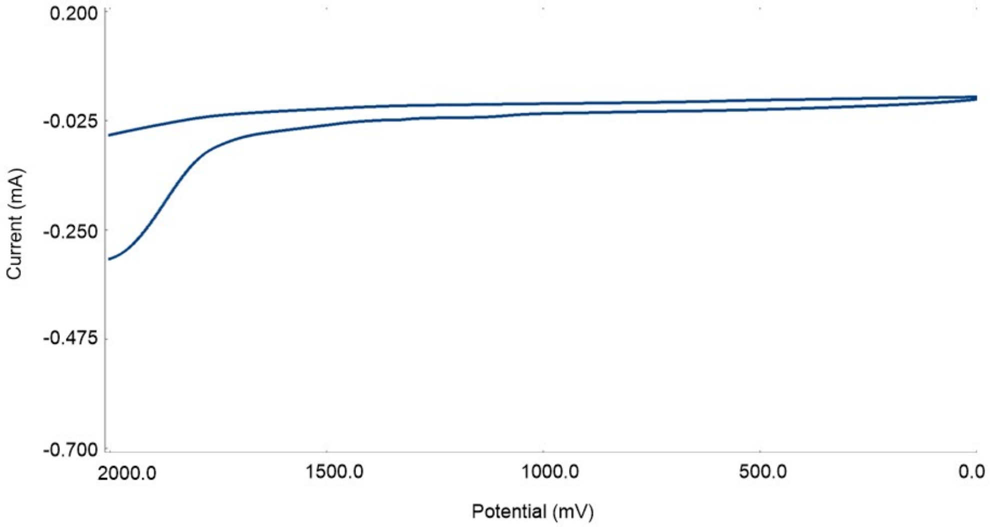

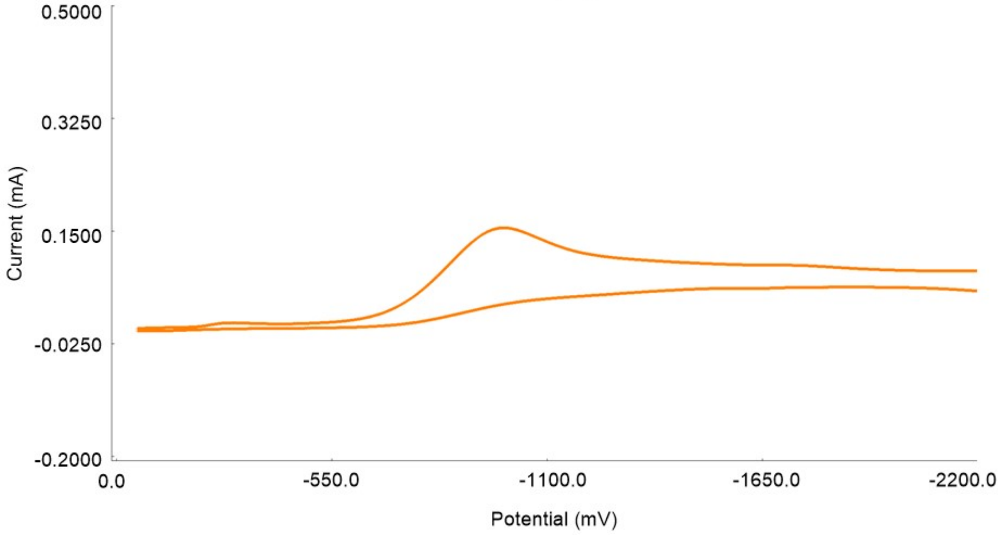

Figure S18. Cyclic Voltammetry for oxidation (top) and reduction (bottom) of $TTCF_3^+OTf^-$ (**1**) in CH_3CN with tetrabutylammonium hexafluorophosphate. E_p = –990 mV.

Cyclic voltammograms of *Umemoto*'s reagent

Cyclic voltammograms were recorded using an Autolab PGSTAT204 potentiostat and a carbon working electrode, a Ag/AgCl reference electrode and a Pt auxiliary electrode. The voltammograms were recorded at room temperature in 0.1 M tetrabutylammonium hexafluorophosphate with CH_3CN (3 mL) containing *Umemoto*'s reagent (CAS registry number 129946-88-9) (12.1 mg, 0.030 mmol, c = 0.01 M). The scan rate was 100 mV s^{-1}. The redox potential of ferrocene was also measured under this standard condition to determine the redox potential *vs*. $Fc^{+/0}$. (Figure S19)

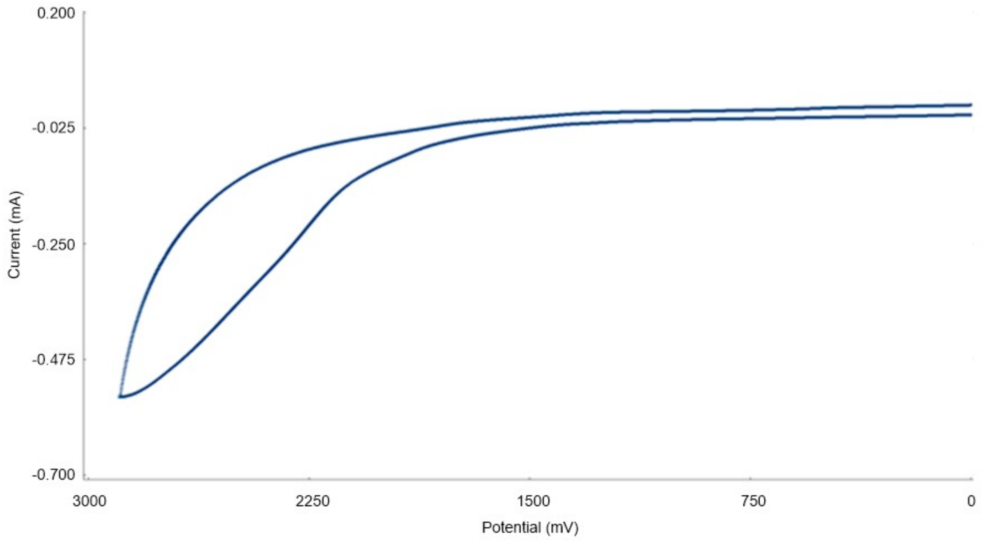

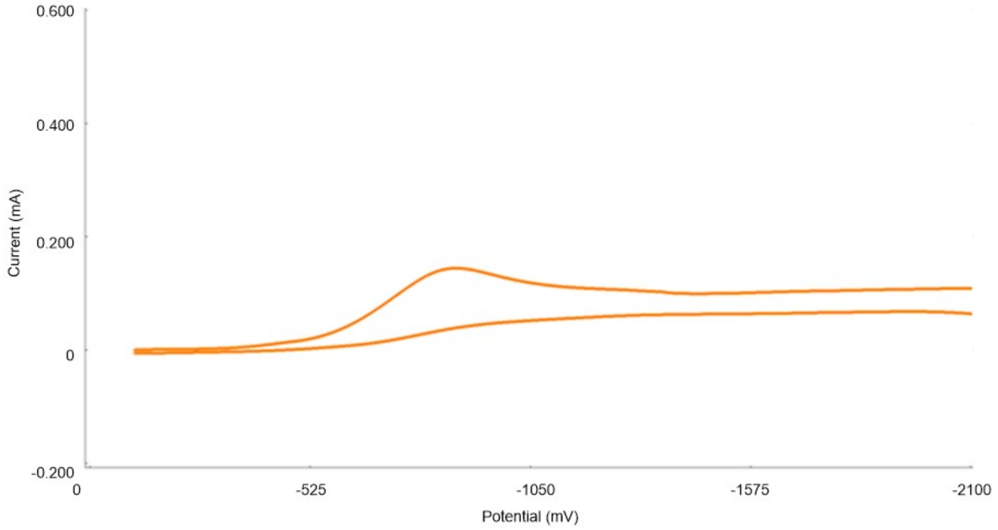

Figure S19. Cyclic Voltammetry for oxidation (top) and reduction (bottom) of *Umemoto*'s reagent in CH_3CN with tetrabutylammonium hexafluorophosphate. E_p = –870 mV.

Cyclic voltammograms of $Ph_2SCF_3^+OTf^-$

Cyclic voltammograms were recorded using an Autolab PGSTAT204 potentiostat and a carbon working electrode, a Ag/AgCl reference electrode and a Pt auxiliary electrode. The voltammograms were recorded at room temperature in 0.1 M tetrabutylammonium hexafluorophosphate with CH_3CN (3 mL) containing $Ph_2SCF_3^+OTf^-$ (CAS registry number 147531-11-1) (12.1 mg, 0.030 mmol, c = 0.01 M). The scan rate was 100 mV s^{-1}. The redox potential of ferrocene was also measured under this standard condition to determine the redox potential *vs*. $Fc^{+/0}$. (Figure S20)

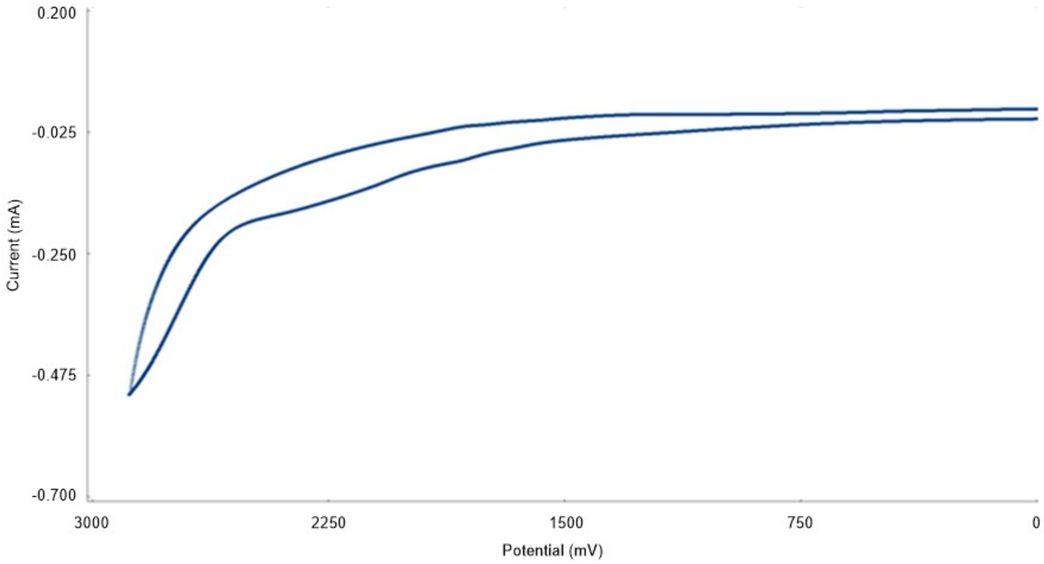

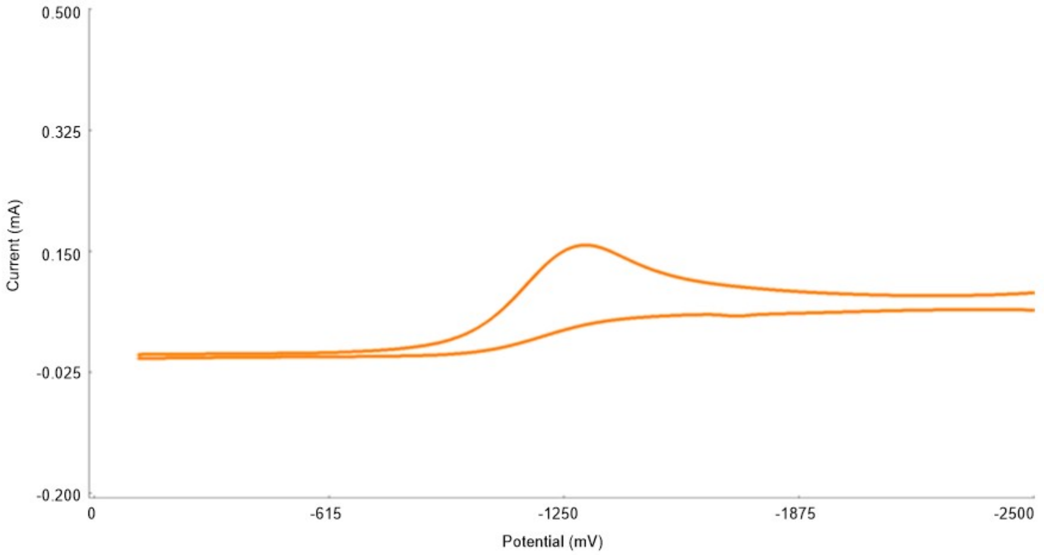

Figure S20. Cyclic Voltammetry for oxidation (top) and reduction (bottom) of $Ph_2SCF_3^+OTf^-$ in CH_3CN with tetrabutylammonium hexafluorophosphate. E_p = –1308 mV.

Mechanistic investigations for $TTCF_3^+OTf^-$ (1) synthesis

Cyclic voltammograms of thianthrene and triflic anhydride

Cyclic voltammograms were recorded using an Autolab PGSTAT204 potentiostat in combination with a glass carbon working electrode, a Ag/AgCl reference electrode, and a Pt auxiliary electrode. The voltammograms were recorded at room temperature in 0.1 M tetrabutylammonium hexafluorophosphate with CH_3CN (3 mL) containing thianthrene (6.5 mg, 0.030 mmol, c = 0.01 M) or triflic anhydride (8.5 mg, 0.030 mmol, c = 0.01 M) respectively. The scan rate was 100 mV s^{-1}. The redox potential of ferrocene was also measured under this standard condition to determine the redox potential *vs*. $Fc^{+/0}$. (Figure S21)

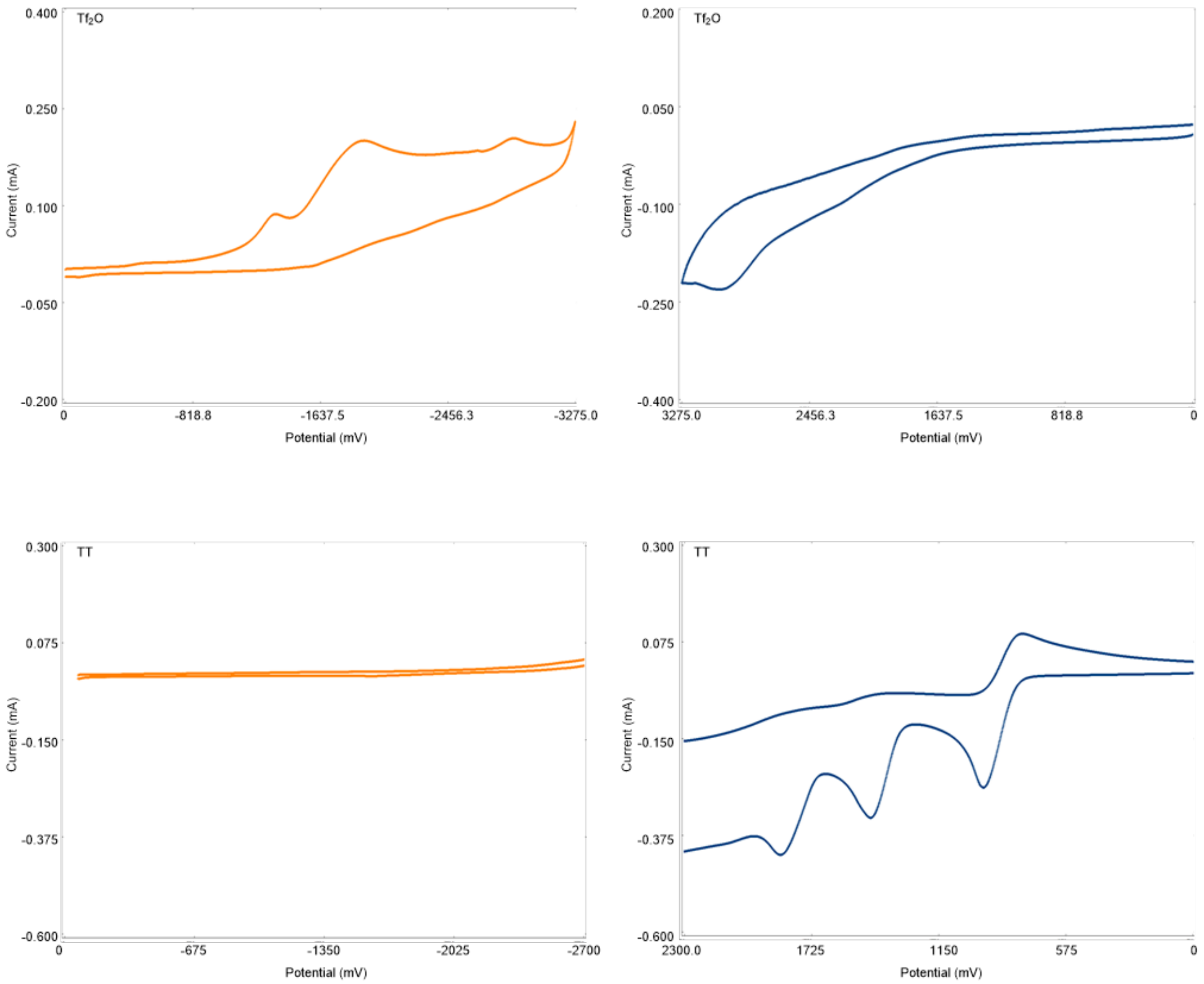

Figure S21. Cyclic Voltammetry for redox potential of triflic anhydride (top) and thianthrene (bottom) in CH_3CN. An oxidation peak of thianthrene at E_{ox} = 859 mV is observed

CF_3 radical and SO_2CF_3 radical trapping experiments

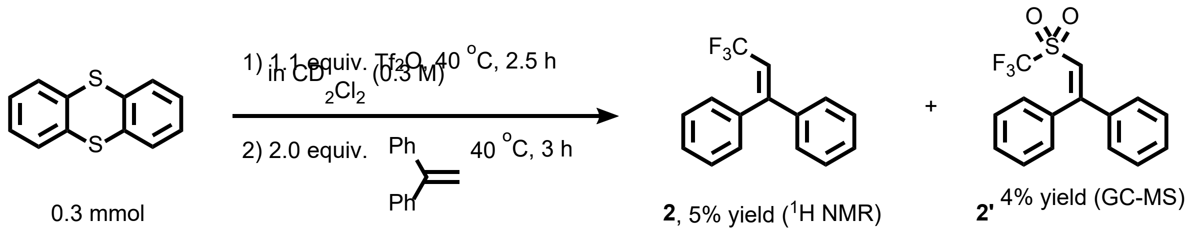

Under an ambient atmosphere, a 4 mL vial equipped with a teflon-coated magnetic stirring bar, was charged

with thianthrene (64.9 mg, 0.300 mmol, 1.00 equiv.). The vial was transferred into a N_2-filled glovebox. Subsequently, dry deuterated DCM (1 mL, c = 0.3 M) and triflic anhydride (93.2 mg, 0.330 mmol, 1.10 equiv.) were added into the vial. The vial was closed with a teflon-lined screw cap and removed from the glovebox. The reaction mixture was stirred at 40 ºC for 2.5 h, followed by addition of 1,1-diphenylethylene (108 mg, 106 μL, 0.600 mmol, 2.00 equiv.) with a Hamilton syringe. The reaction mixture was then stirred at 40 ºC for 3 h to obtain product **2** with 5% yield (determined by ^{1}H NMR integration relative to the internal standard (dibromomethane, 21.0 μL, 0.300 mmol, 1.00 equiv.)).

To confirm product **2** in NMR and MS, a standard specimen of **2** was prepared according to the literature method.[9] Product **2** was confirmed by comparison with the ^{1}H NMR, ^{19}F NMR spectrum, and GC-MS of the standard specimen. (Figure S22, S23, S24 and S25)

2' was observed in GC-MS. Molecular ion and fragments pointed out **2'**. (Figure S25) The yield of product **2'** (4% yield) was calculated according to following information:

1. The yield (5%) of product **2** was determined by ^{1}H NMR spectroscopy with dibromomethane as an internal standard. (Figure S22)
2. The molecular ions and corresponding fragments of product **2** and **2'** were observed by GC-MS, their retention times were 7.086 (product **2**) and 9.707 (product **2'**) minutes, respectively. (Figure S25).
3. According to the GC-FID shown below, the area ratio of product **2**/product **2'** is 1.3/1 (Figure S26).

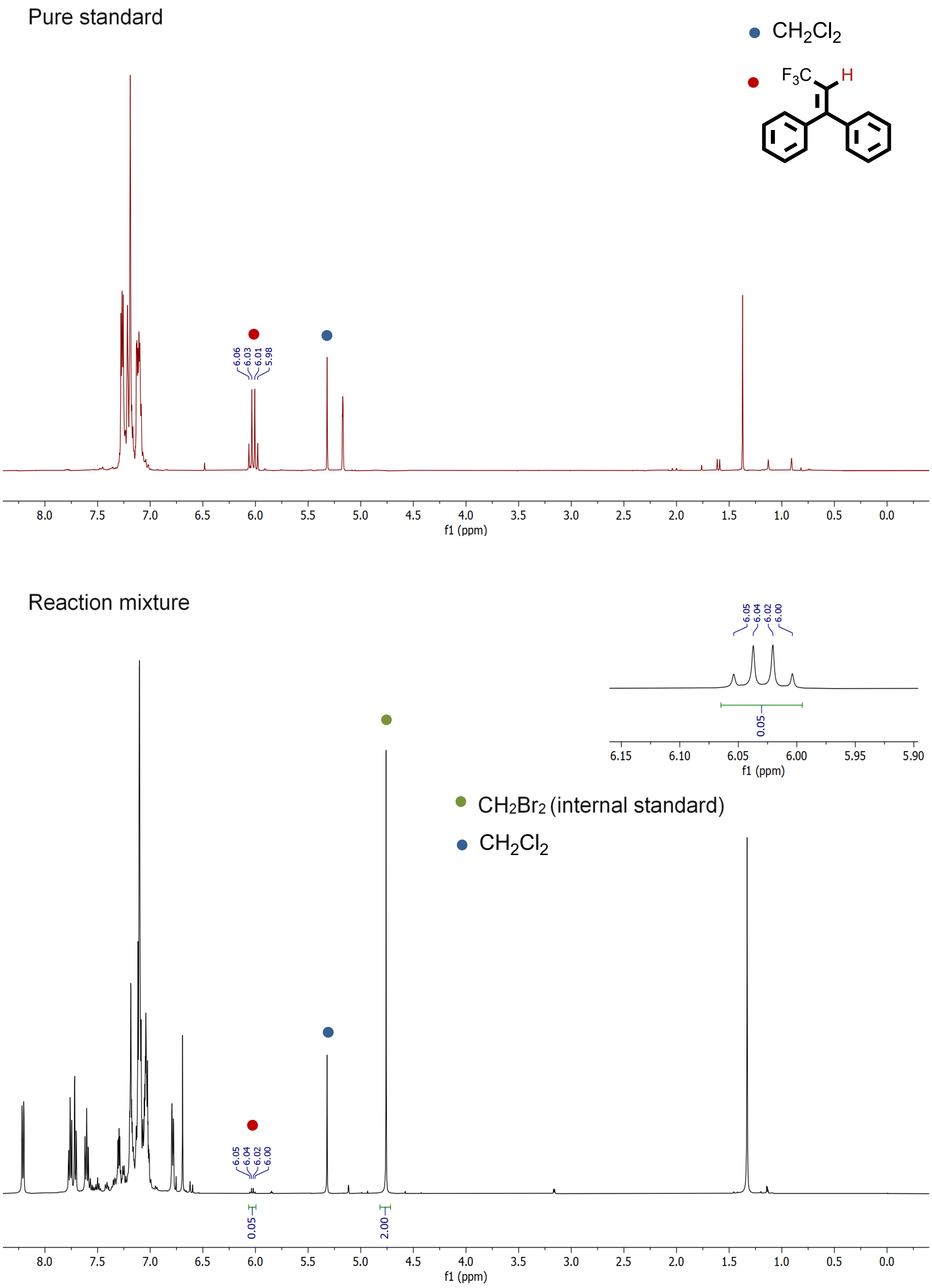

Figure S22. The ^{1}H NMR spectrum (in CD_2Cl_2) of pure (3,3,3-trifluoroprop-1-ene-1,1-diyl)dibenzene (top) and trapping experiment mixture (bottom).

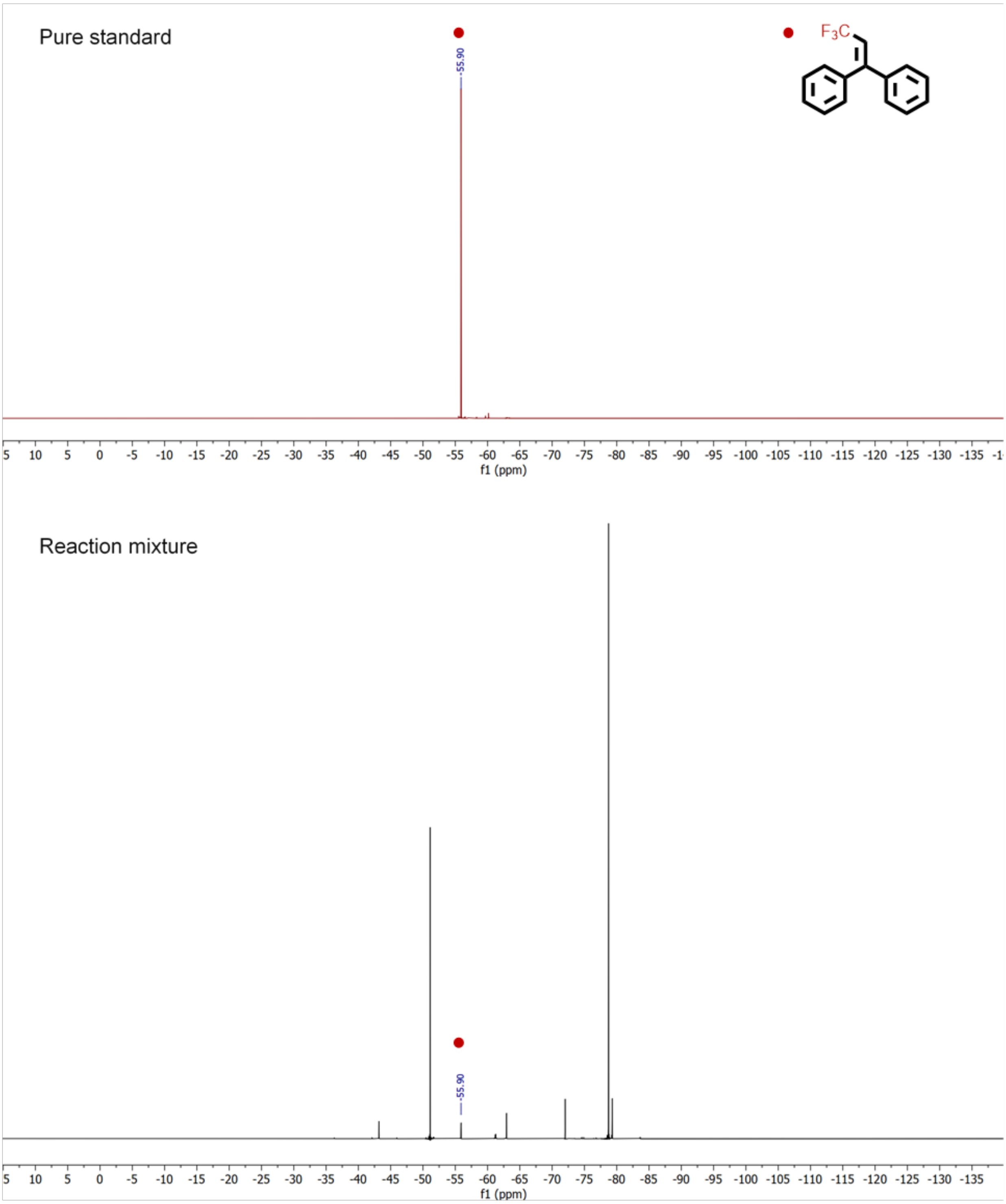

Figure S23. The ^{19}F NMR spectrum (CD_2Cl_2) of pure (3,3,3-trifluoroprop-1-ene-1,1-diyl)dibenzene (top) and trapping experiment mixture (bottom).

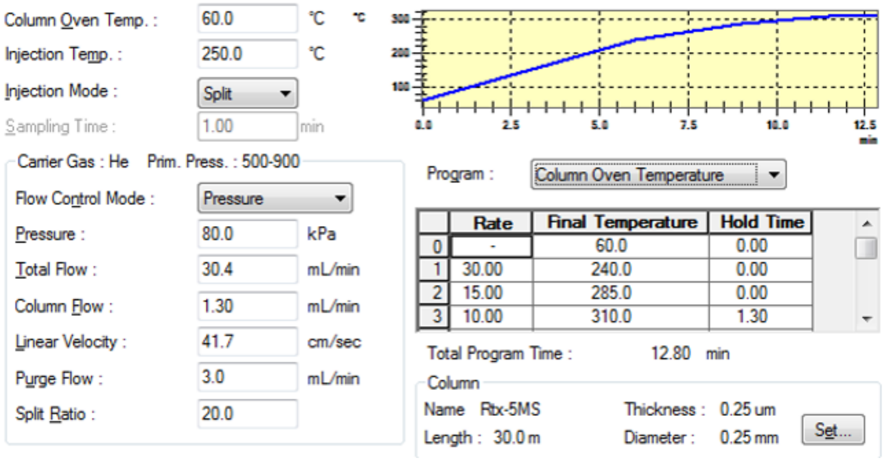

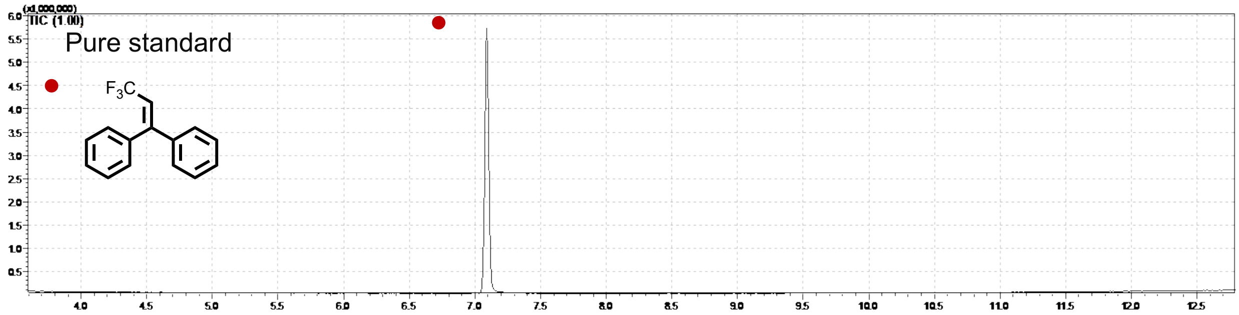

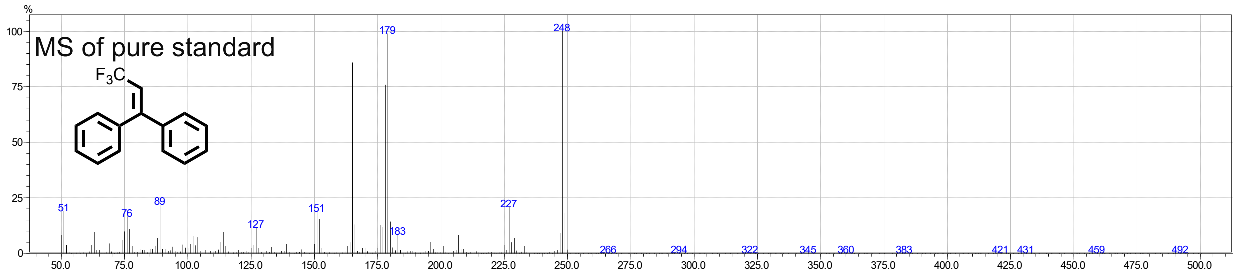

Figure S24. GC-MS data of pure standard.

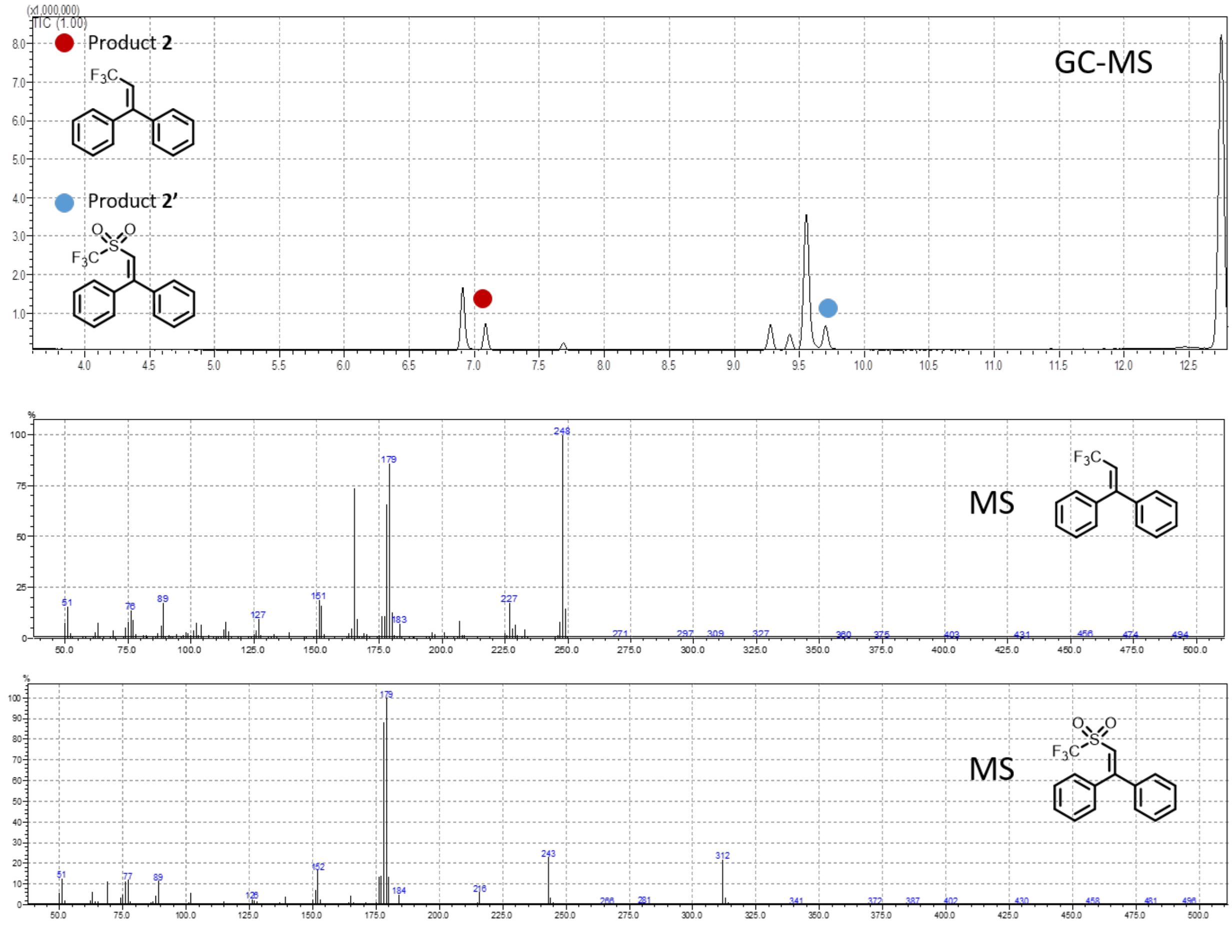

Figure S25. GC-MS data of trapping reaction mixture.

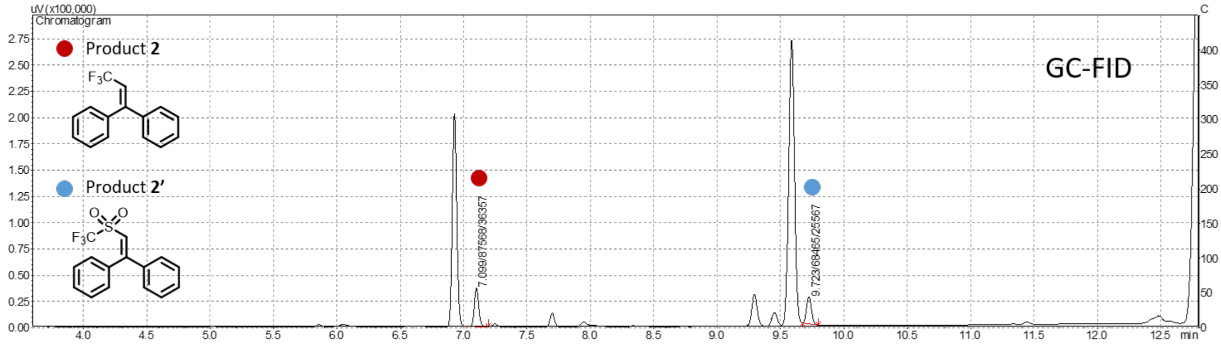

Peak Table | Compound | Group | Calibration Curve

Peak#	Ret.Time	Initial Time	Final Time	Area	Height	Tailing F.	k'	Separation F.	T.Plate	Resolution	A/H
1	7.099	7.033	7.192	87567.8	36357.2	1.110	0.000	0.000	203831.913	--	2.41
2	9.723	9.675	9.796	68465.3	25566.7	1.068	0.370	0.000	291379.195	38.882	2.68

Figure S26. GC-FID data of trapping reaction mixture.

CF_3 radical and SO_2CF_3 radical control experiment

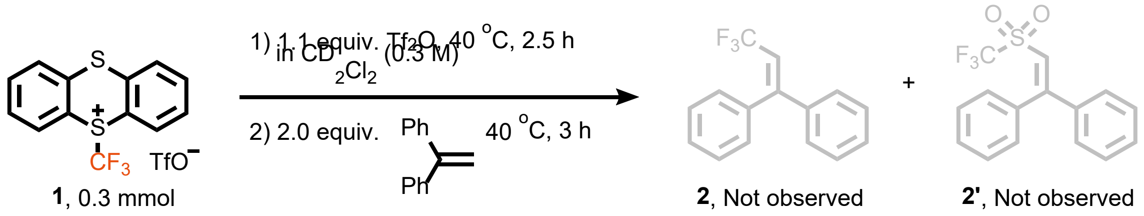

Under an ambient atmosphere, a 4 mL vial equipped with a teflon-coated magnetic stirring bar, was charged with $TTCF_3^+OTf^-$ (**1**, 130 mg, 0.300 mmol, 1.00 equiv.). The vial was transferred into a N_2-filled glovebox. Subsequently, dry deuterated DCM (1 mL, c = 0.3 M) and triflic anhydride (93.2 mg, 0.330 mmol, 1.10 equiv.) were added into the vial. The vial was closed with a teflon-lined screw cap and removed from the glovebox. The reaction mixture was stirred at 40 ºC for 2.5 h, followed by addition of 1,1-diphenylethylene (108 mg, 106 µL, 0.600 mmol, 2.00 equiv.) with a Hamilton syringe. The reaction mixture was then stirred at 40 ºC for 3 h. No characteristic signals of **2** were observed in ^1H NMR, ^{19}F NMR, and GC-MS. Characteristic signals of **2'** were not observed in GC-MS.

EPR Measurements

EPR experiments

Electron paramagnetic resonance (EPR) spectra were obtained using a commercial X-band spectrometer (MS5000, Magnettech GmbH) at 150 K. The EPR spectra were recorded at microwave frequency of 9.626 GHz using 0.1 mW microwave power, 10 mT field sweep centered at 341.9 mT, a modulation amplitude of 0.3 mT and a sweep time 5 min.

In a nitrogen-filled glovebox, thianthrene (86.4 mg, 0.40 mmol) and triflic anhydride (113 mg, 0.40 mmol) were dissolved in anhydrous DCM (10 mL, *c* = 0.04 M) before being transferred into a 4 mm (O.D.) EPR tube. The tube was sealed with a rubber septum and brought out of the glovebox. The tube was placed into a Dewar, liquid nitrogen was poured into the Dewar, freezing the sample. The sample was kept in liquid nitrogen until it could be measured.

Results and discussion

EPR measurements for sample displayed the formation of a radical signal with a rhombic g tensor (2.017, 2.012 and 2.006) (Figure S27). This signal matches very well with the previously observed thianthrene (TT) radical cation signal from the literature.[10] Therefore, it is assigned to TT radical cation.

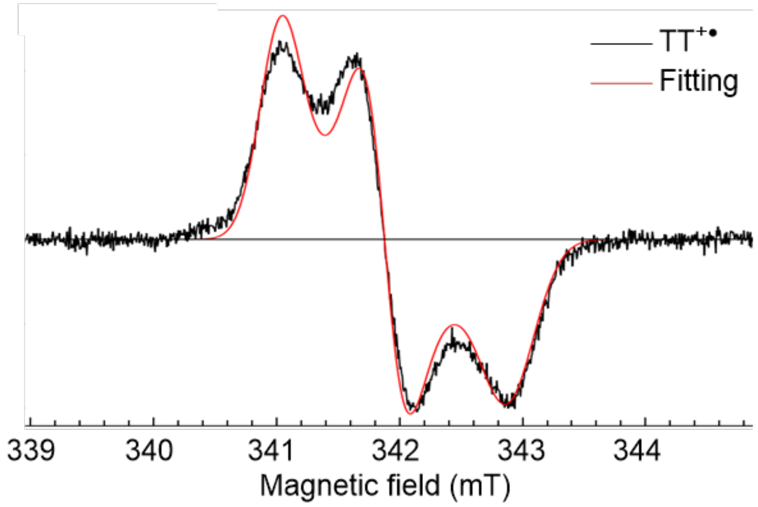

Figure S27. X-band EPR spectra recorded at 150 K are shown for TT radical cation (black).

DFT Calculation

Methods

Density Functional Theory (DFT) calculations were performed on the Max-Planck-Institut für Kohlenforschung computer cluster using the ORCA program package (Version 4.1.x-Stable).[11] Unless denoted otherwise, structural optimizations and frequency calculations to identify all of the stationary points as minima (zero imaginary frequencies) and to obtain thermal and entropic correction were performed with the B3LYP functional[12,13] with D3 dispersion correction[14] and Becke-Johnson damping (BJ)[15] along with RI approximation, utilizing the def2/J auxiliary basis set[16] and the def2-TZVPP basis set[16] on all atoms. The libint2 library was used for the computation of 2-el integrals.[18] Tight SCF convergence and geometry optimization criterions were chosen. Solvent effects of dichloromethane were taken into account using the conductor-like polarized continuum model (CPCM).[19] The reported energies are enthalpies and Gibbs free energies in solution. Input files and images were created using Avogadro 1.2.[20]

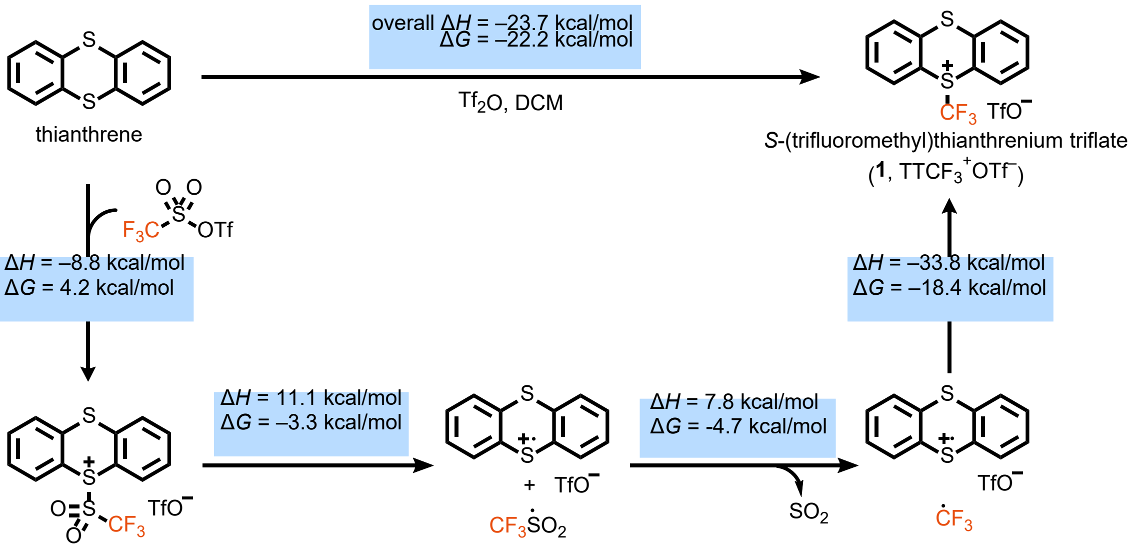

Figure S28. Energy profile for the formation mechanism of $TTCF_3^+OTf^-$.

In this reaction mechanism, the first step is the nucleophilic attack between thianthrene and triflic anhydride. The bond dissociation energy of the S-S bond in the generated $TTTf^+OTf^-$ is low (BDE = 11.1 kcal/mol), and a homolytic cleavage reaction could happen to form a thianthrenium radical cation and a CF_3SO_2 radical. The CF_3SO_2 radical could generate a CF_3 radical together with sulfur dioxide, and this step is exothermic by 4.7 kcal/mol. The CF_3 radical is trapped by the persistent thianthrenium radical cation to form the product $TTCF_3^+OTf^-$. The overall conversion from thianthrene and triflic anhydride to $TTTf^+OTf^-$ is exothermic by 22.2 kcal/mol.

Calculated Coordinates:

Thianthrene

C	-4.45616	3.88513	0.32296
C	-5.83362	3.74381	0.20229
C	-6.37940	2.47887	0.05639
C	-5.57192	1.33848	0.02641

C	-4.18939	1.47924	0.15543
C	-3.64858	2.75943	0.30239
S	-6.45828	-0.17349	-0.19648
C	-5.26569	-1.47819	-0.16935
C	-3.88304	-1.33740	-0.04143
S	-2.99464	0.17631	0.15937
C	-5.80806	-2.75931	-0.30073
C	-5.00148	-3.88590	-0.31093
C	-3.62383	-3.74468	-0.19248
C	-3.07688	-2.47900	-0.05819
H	-4.01043	4.86347	0.43658
H	-6.48150	4.60896	0.21968
H	-7.45119	2.36068	-0.04164
H	-2.57583	2.86458	0.40370
H	-6.88109	-2.86450	-0.39876
H	-5.44818	-4.86496	-0.41402
H	-2.97678	-4.61059	-0.20125
H	-2.00508	-2.36096	0.04007

Tf_2O

C	0.72250	0.09792	-2.07580
F	1.85336	0.10854	-2.77178
F	0.29364	1.33689	-1.88815
F	-0.19726	-0.60708	-2.71915
S	1.09660	-0.70510	-0.42609
O	2.03132	0.10867	0.26837
O	1.25330	-2.09676	-0.64531
O	-0.39139	-0.50021	0.22982
S	-0.69266	0.44879	1.55379
O	-0.09619	-0.15813	2.69002
O	-0.53065	1.81618	1.20612
C	-2.52750	0.04999	1.54785
F	-2.71342	-1.24438	1.74851
F	-3.06293	0.41443	0.39170
F	-3.07315	0.74386	2.53971

[TT-Tf$^+$][OTf$^-$]

C	0.52569	3.64475	0.17370
C	-0.78592	3.65674	0.64640
C	-1.53551	2.49478	0.69018
C	-0.99895	1.28453	0.24276
C	0.30934	1.29280	-0.24878
C	1.07717	2.46022	-0.27091
S	-2.04756	-0.11029	0.35892
C	-0.98291	-1.49654	0.29839
C	0.31028	-1.51121	-0.23313
S	1.10188	-0.11624	-0.90517
C	-1.49711	-2.69559	0.79561
C	-0.74236	-3.85634	0.75452
C	0.55131	-3.85164	0.23998
C	1.08434	-2.67442	-0.24199
H	1.11572	4.54944	0.16867
H	-1.22013	4.57892	1.00673
H	-2.53851	2.50875	1.09314
H	2.10113	2.41385	-0.61082
H	-2.48753	-2.70545	1.22860
H	-1.16000	-4.77047	1.15283
H	1.14986	-4.75033	0.24615
H	2.10092	-2.63333	-0.60410
C	1.48220	0.52870	3.25013

F	1.76463	1.83306	3.10517
F	1.50821	0.23497	4.55147
F	0.21625	0.34068	2.81207
S	2.67661	-0.53662	2.29706
O	3.93797	-0.31549	2.94656
O	2.61189	0.06377	0.93964
O	2.08888	-1.85438	2.37066
S	0.00244	0.10314	-2.98412
C	-0.26428	-1.68162	-3.59883
O	-1.30707	0.68372	-2.85142
O	1.01307	0.67513	-3.83337
F	-0.57405	-1.63102	-4.88626
F	0.86292	-2.37169	-3.43411
F	-1.24708	-2.26265	-2.92283

[TT$^{\bullet+}$][OTf^{-}]

C	0.26954	-2.15304	-0.62301
F	0.06678	-3.47578	-0.69316
F	0.92257	-1.90205	0.53369
F	1.07920	-1.79814	-1.62718
S	-1.34410	-1.22761	-0.68003
O	-2.06109	-1.77405	0.46627
O	-1.89197	-1.53811	-1.97581
O	-0.93043	0.16515	-0.49557
C	0.76175	-1.45801	3.91456
C	-0.08275	-2.47818	4.36791
C	-1.43610	-2.42475	4.11368
C	-1.98532	-1.35124	3.39993
C	-1.13738	-0.32316	2.94884
C	0.24014	-0.39456	3.21524
S	-3.70312	-1.40650	3.18652
C	-4.09882	-0.21838	1.98798
C	-3.24263	0.79493	1.52608
S	-1.62604	1.07107	2.06265
C	-5.39173	-0.31505	1.45741
C	-5.81381	0.55980	0.47992
C	-4.95141	1.55359	0.00314
C	-3.68239	1.67134	0.52235
H	1.82575	-1.51336	4.09457
H	0.32705	-3.32132	4.90606
H	-2.08595	-3.22318	4.44501
H	0.88860	0.37987	2.82929
H	-6.04455	-1.10556	1.80205
H	-6.80636	0.45866	0.06468
H	-5.27038	2.21633	-0.78846
H	-2.99331	2.40580	0.12985

$CF_3SO_2^{\bullet}$

F	-0.01825	-0.42708	1.00227
F	-0.28232	1.22640	-0.37938
F	-0.34593	-0.81677	-1.10963
C	-0.63537	-0.01818	-0.09516
S	-2.55975	-0.06141	0.19879
O	-2.77588	0.80518	1.34268
O	-2.90251	-1.47150	0.21614

$CF_3^{\bullet}$

F	-0.10379	-0.48994	0.98681
F	-0.27990	1.24406	-0.32754

F	-0.34501	-0.77245	-1.16339
C	-0.63819	-0.01172	-0.12306

SO_2

S	1.37561	-0.23735	-2.96259
O	0.86281	0.98114	-3.52984
O	2.78972	-0.49395	-3.02712

[TT-CF_3^+][OTf^-]

C	0.45536	3.59454	0.39937
C	-0.86483	3.59685	0.84065
C	-1.66003	2.46837	0.71208
C	-1.15423	1.31717	0.10834
C	0.16024	1.34519	-0.36040
C	0.97857	2.45926	-0.19407
S	-2.22598	-0.07122	-0.03475
C	-1.12923	-1.44218	0.10706
C	0.18502	-1.44903	-0.36969
S	0.88500	-0.04592	-1.16296
C	-1.61359	-2.59941	0.70979
C	-0.79834	-3.71792	0.82897
C	0.51597	-3.69524	0.38049
C	1.02053	-2.54905	-0.21068
H	1.08118	4.46390	0.53858
H	-1.27100	4.47650	1.32074
H	-2.66774	2.46167	1.10258
H	2.01356	2.41026	-0.49632
H	-2.61846	-2.60991	1.10730
H	-1.18732	-4.60372	1.31189
H	1.16167	-4.54850	0.52435
H	2.05417	-2.48908	-0.51294
C	1.54686	0.28457	3.02614
F	1.57563	1.62872	2.97933
F	1.64692	-0.08751	4.30436
F	0.32533	-0.10029	2.58223
S	2.89947	-0.46559	1.98703
O	4.10993	0.02539	2.58801
O	2.61506	0.10857	0.65689
O	2.63095	-1.88579	2.07077
C	-0.10256	-0.05093	-2.82628
F	0.83120	-0.06711	-3.78217
F	-0.86114	-1.12993	-2.95706
F	-0.84141	1.04089	-2.97280

X-RAY CRYSTALLOGRAPHIC ANALYSIS

S-(Trifluoromethyl)thianthrenium triflate (1, $TTCF_3^+OTf^-$) (CCDC 2046668)

Experimental

Crystal of *S*-(trifluoromethyl)thianthrenium triflate (**1**) was obtained by suspending $TTCF_3^+OTf^-$ (**1**, 86.9 mg, 0.2 mmol) at room temperature in DCM (approximately 0.2 mL) until the solids are almost fully dissolved. The saturated solution was filtered through a syringe filter (PTFE, 0.2 µm) into a 4 mL vial held at room temperature. A few drops of Et_2O (approximately 0.5 mL) were added and the solution was stood at room temperature over 48 h while colorless crystals were formed.

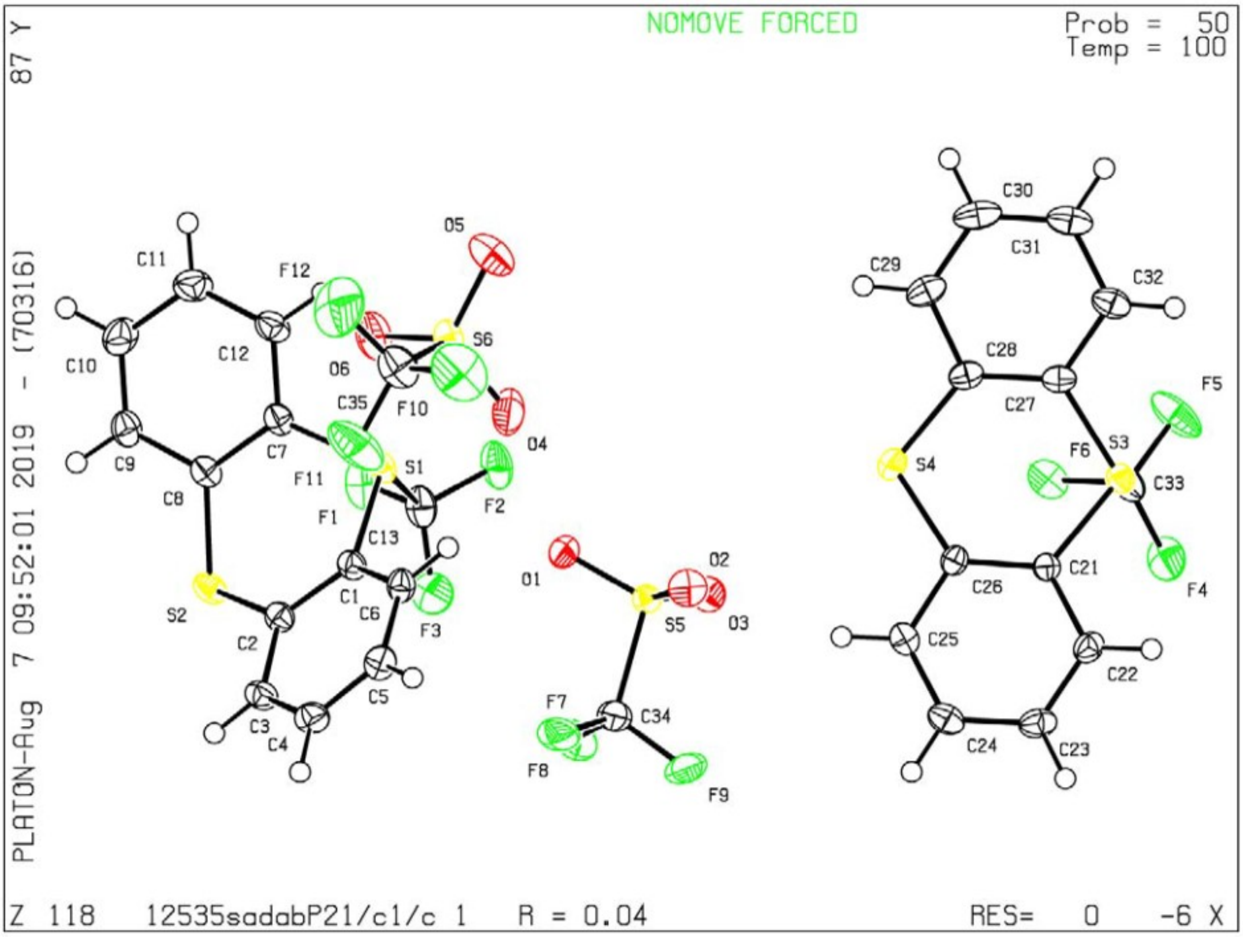

The atoms are depicted with 50% probability ellipsoids. The crystallographic data are summarized in the following table.

Table 25. Crystal data and structure refinement

Identification code	12535
Empirical formula	$C_{14} H_8 F_6 O_3 S_3$
Color	colourless
Formula weight	434.38 g · mol^{-1}
Temperature	100(2) K
Wavelength	0.71073 Å

Crystal system	MONOCLINIC	
Space group	**$P2_1/c$, (no. 14)**	
Unit cell dimensions	a = 10.2838(11) Å	α= 90°.
	b = 23.4654(12) Å	β= 106.954(9)°.
	c = 14.5614(14) Å	γ= 90°.
Volume	3361.1(5) $Å^3$	
Z	8	
Density (calculated)	1.717 Mg · m^{-3}	
Absorption coefficient	0.516 mm^{-1}	
F(000)	1744 e	
Crystal size	0.30 x 0.26 x 0.13 mm^3	
θ range for data collection	2.925 to 30.507°.	
Index ranges	$-14 \le h \le 14$, $-33 \le k \le 33$, $-20 \le l \le 20$	
Reflections collected	117468	
Independent reflections	10265 [R_{int} = 0.0595]	
Reflections with I>2σ (I)	8893	
Completeness to θ = 25.242°	99.8 %	
Absorption correction	Gaussian	
Max. and min. transmission	1.00 and 0.68	
Refinement method	Full-matrix least-squares on F^2	
Data / restraints / parameters	10265 / 0 / 469	
Goodness-of-fit on F^2	1.035	
Final R indices [I>2σ (I)]	R_1 = 0.0437	wR^2 = 0.1136
R indices (all data)	R_1 = 0.0520	wR^2 = 0.1196
Largest diff. peak and hole	1.4 and -1.0 e · $Å^{-}$	

Analysis

In the unit cell, the two different $TTCF_3^+OTf^-$ crystal structures show different S-CF_3 bond lengths (1.876 Å and 1.909 Å). The difference in bond lengths can be explained in the two different interaction pathways of the OTf anion. The S3 atom interacts with only one O3' atom of a OTf anion at a distance of 2.753 Å with a smaller dihedral angle with respect to the two benzene rings (17.19°) and a shorter S3-C33 bond length (1.867 Å). In the other S1 atom of this asymmetric unit, there is a different coordination sphere around the S1 atom, with respect to O1 and O4 atom from two independent OTf anions with a distance of 3.031 Å and 3.131 Å, respectively. The two interactions lengthen the S1-C13 bond length to 1.909 Å, and increase the dihedral angle (45.76°). (O3' is another O3 in the next unit cell, which stands in the below position of S3-C33 bond. O3' is not drawn in above Figure)

If the OTf^- counterion is replaced by BF_4^- (shown in the next section), there is no significant difference in the S-CF_3 bond lengths (1.871 Å and 1.873 Å).

S-(Trifluoromethyl)thianthrenium tetrafluoroborate (1', $TTCF_3^+BF_4^-$) (CCDC 2046669)

Experimental

Crystal of *S*-(trifluoromethyl)thianthrenium tetrafluoroborate (**1'**) was obtained by suspending $TTCF_3^+BF_4^-$ (**1'**, 74.4 mg, 0.2 mmol) at room temperature in DCM (approximately 0.4 mL) until the solids are almost fully dissolved. The saturated solution was filtered through a syringe filter (PTFE, 0.45 µm) into a 4 mL vial held at room temperature. A few drops of *n*-pentane (approximately 0.5 mL) were added and the solution was stood at room temperature over 48 h while colorless crystals were formed.

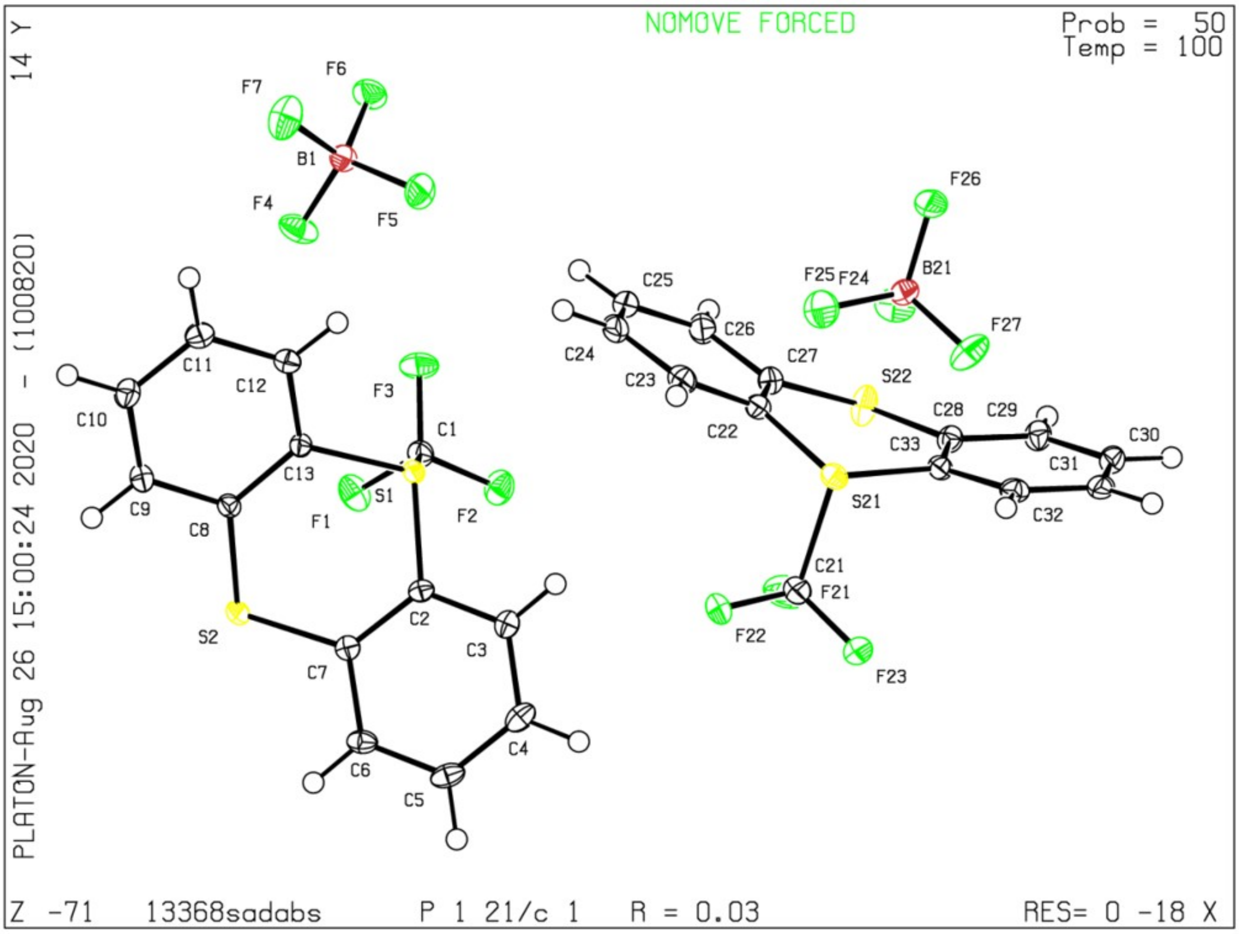

The atoms are depicted with 50% probability ellipsoids. The crystallographic data are summarized in the following table.

Table 26. Crystal data and structure refinement

Empirical formula	$C_{13}H_8BF_7S_2$
Color	colourless
Formula weight	372.12 g·mol^{-1}
Temperature	100(2) K
Wavelength	0.71073 Å
Crystal system	Monoclinic
Space group	*P* 2_1/c, (No. 14)

Unit cell dimensions	a = 9.8738(16) Å	α= 90°.
	b = 26.162(4) Å	β= 105.293(7)°.
	c = 11.4011(18) Å	γ= 90°.
Volume	2840.8(8) $Å^3$	
Z	8	
Density (calculated)	1.740 $Mg·m^{-3}$	
Absorption coefficient	0.447 mm^{-1}	
F(000)	1488 e	
Crystal size	0.053 x 0.048 x 0.042 mm^3	
θ range for data collection	1.557 to 30.997°.	
Index ranges	$-13 \leq h \leq 14, -37 \leq k \leq 37, -16 \leq l \leq 16$	
Reflections collected	82845	
Independent reflections	9068 [R_{int} = 0.0414]	
Reflections with I>2σ (I)	7471	
Completeness to θ = 25.242°	100.0 %	
Absorption correction	Gaussian	
Max. and min. transmission	0.98935 and 0.98089	
Refinement method	Full-matrix least-squares on F^2	
Data / restraints / parameters	9068 / 0 / 415	
Goodness-of-fit on F^2	1.024	
Final R indices [I>2σ (I)]	R_1 = 0.0296	wR^2 = 0.0693
R indices (all data)	R_1 = 0.0413	wR^2 = 0.0746
Extinction coefficient	n/a	
Largest diff. peak and hole	0.452 and -0.262 $e·Å^{-3}$	

5-(Trifluoromethyl)dibenzothiophenium triflate (*Umemoto*'s reagent) (CCDC 2046667)

Experimental

Crystal of *Umemoto*'s reagent (CAS registry number 129946-88-9) was obtained by suspending this reagent (80.5 mg, 0.2 mmol) at room temperature in DCM (approximately 0.3 mL) until the solids are almost fully dissolved. The saturated solution was filtered through a syringe filter (PTFE, 0.45 µm) into a 4 mL vial held at room temperature. A few drops of *n*-pentane (approximately 0.5 mL) were added and the solution was stood at room temperature over 48 h while colorless crystals were formed.

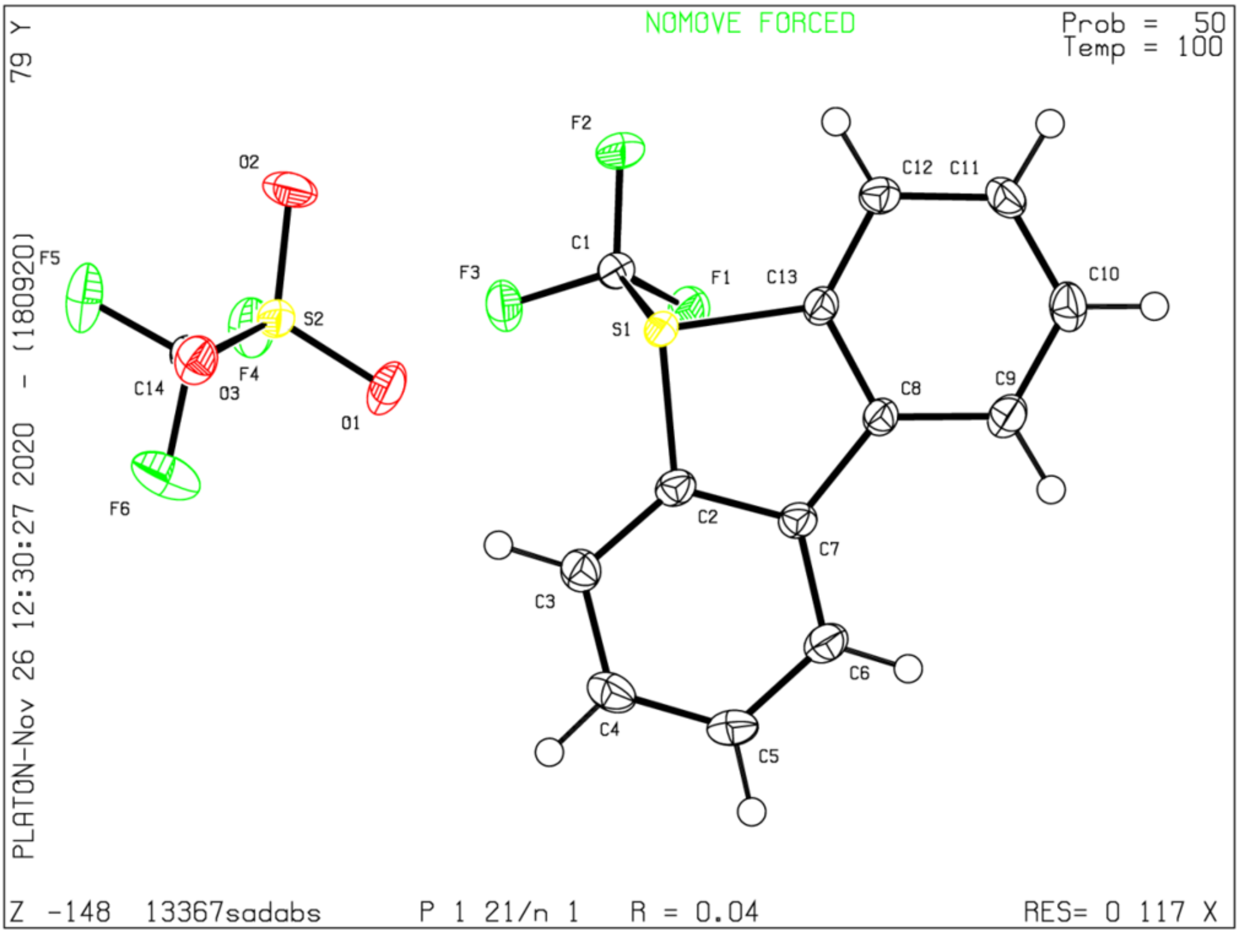

The atoms are depicted with 50% probability ellipsoids. The crystallographic data are summarized in the following table.

Table 27. Crystal data and structure refinement

Empirical formula	$C_{14} H_8 F_6 O_3 S_2$
Color	colourless
Formula weight	402.32 g·mol^{-1}
Temperature	100(2) K
Wavelength	1.54178 Å
Crystal system	Monoclinic
Space group	*P*2$_1$/n, (No. 14)

Unit cell dimensions	a = 10.1103(4) Å	α= 90°.
	b = 9.2541(3) Å	β= 101.953(2)°.
	c = 16.4268(6) Å	γ= 90°.
Volume	1503.60(10) $Å^3$	
Z	4	
Density (calculated)	1.777 $Mg \cdot m^{-3}$	
Absorption coefficient	4.018 mm^{-1}	
F(000)	808 e	
Crystal size	0.288 x 0.143 x 0.060 mm^3	
θ range for data collection	4.739 to 66.582°.	
Index ranges	$-11 \leq h \leq 11$, $-10 \leq k \leq 11$, $-19 \leq l \leq 18$	
Reflections collected	37790	
Independent reflections	2577 [R_{int} = 0.0716]	
Reflections with $I>2\sigma(I)$	2173	
Completeness to θ = 66.582°	96.9 %	
Absorption correction	Gaussian	
Max. and min. transmission	0.79084 and 0.49431	
Refinement method	Full-matrix least-squares on F^2	
Data / restraints / parameters	2577 / 0 / 226	
Goodness-of-fit on F^2	1.043	
Final R indices [$I>2\sigma(I)$]	R_1 = 0.0364	wR^2 = 0.0880
R indices (all data)	R_1 = 0.0472	wR^2 = 0.0949
Extinction coefficient	n/a	
Largest diff. peak and hole	0.621 and -0.403 $e \cdot Å^{-3}$	

SPECTROSCOPIC DATA

^{1}H NMR of *S*-(trifluoromethyl)thianthrenium triflate (1)

CDCl3, 25 ºC

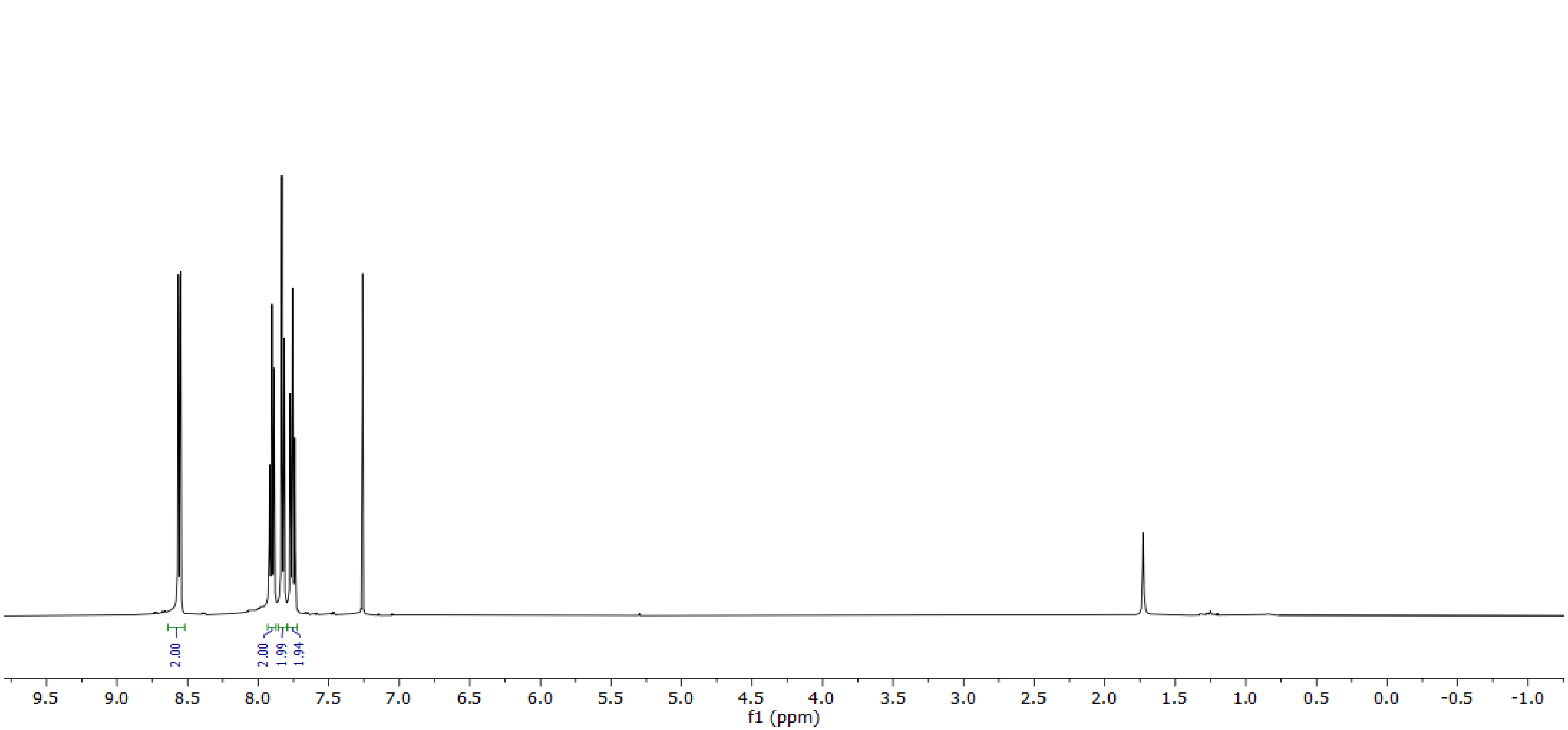

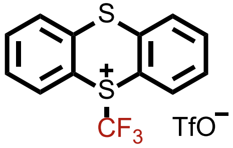

(1, $TTCF_3^+OTf^-$)

^{19}F NMR of *S*-(trifluoromethyl)thianthrenium triflate (1)

CDCl3, 25 ºC

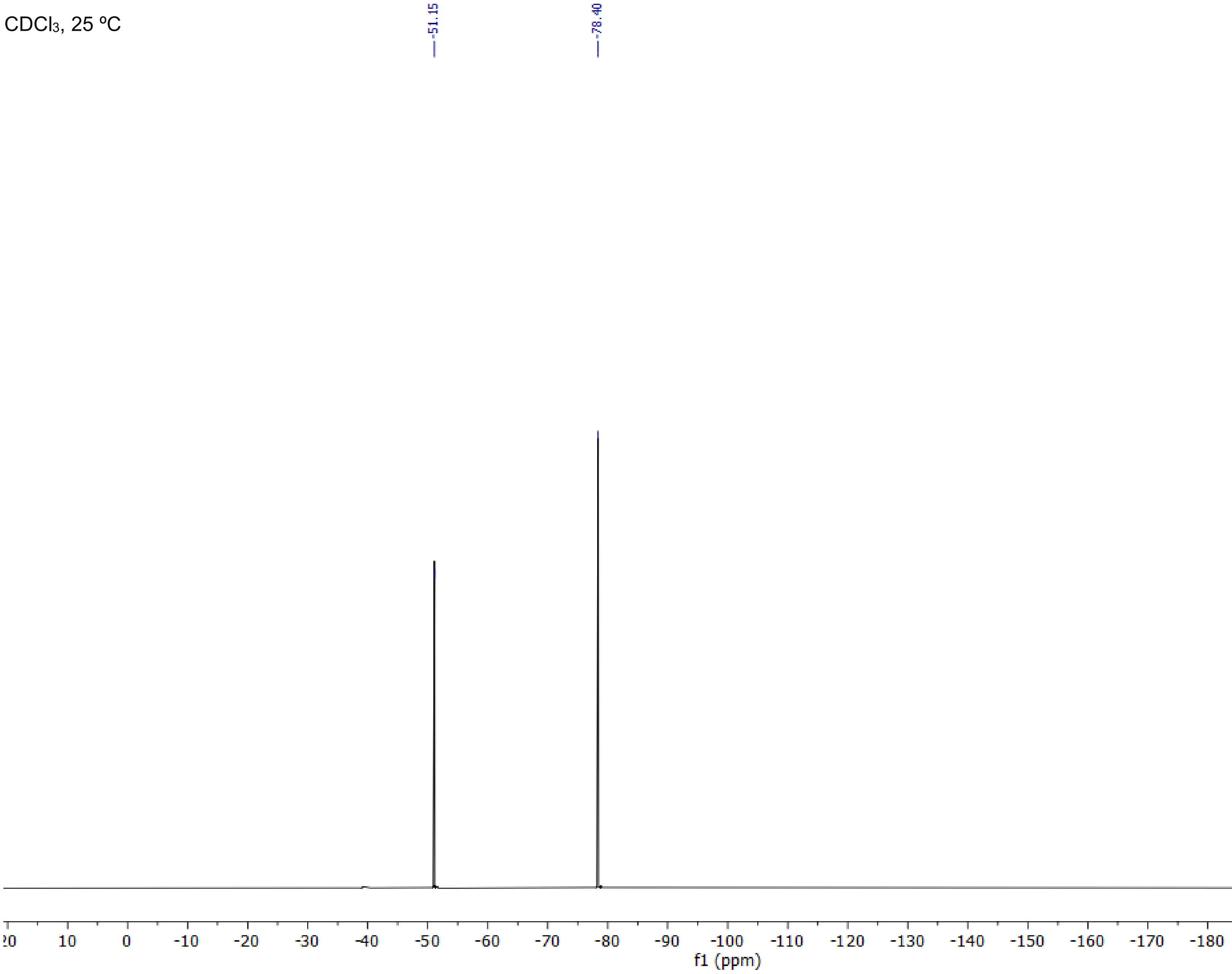

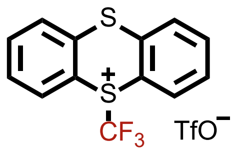

(1, $TTCF_3^+OTf^-$)

^{13}C NMR of *S*-(trifluoromethyl)thianthrenium triflate (1)

$CDCl_3$, 25 ºC

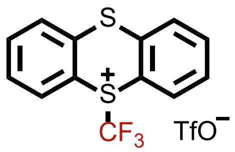

(1, $TTCF_3^+OTf^-$)

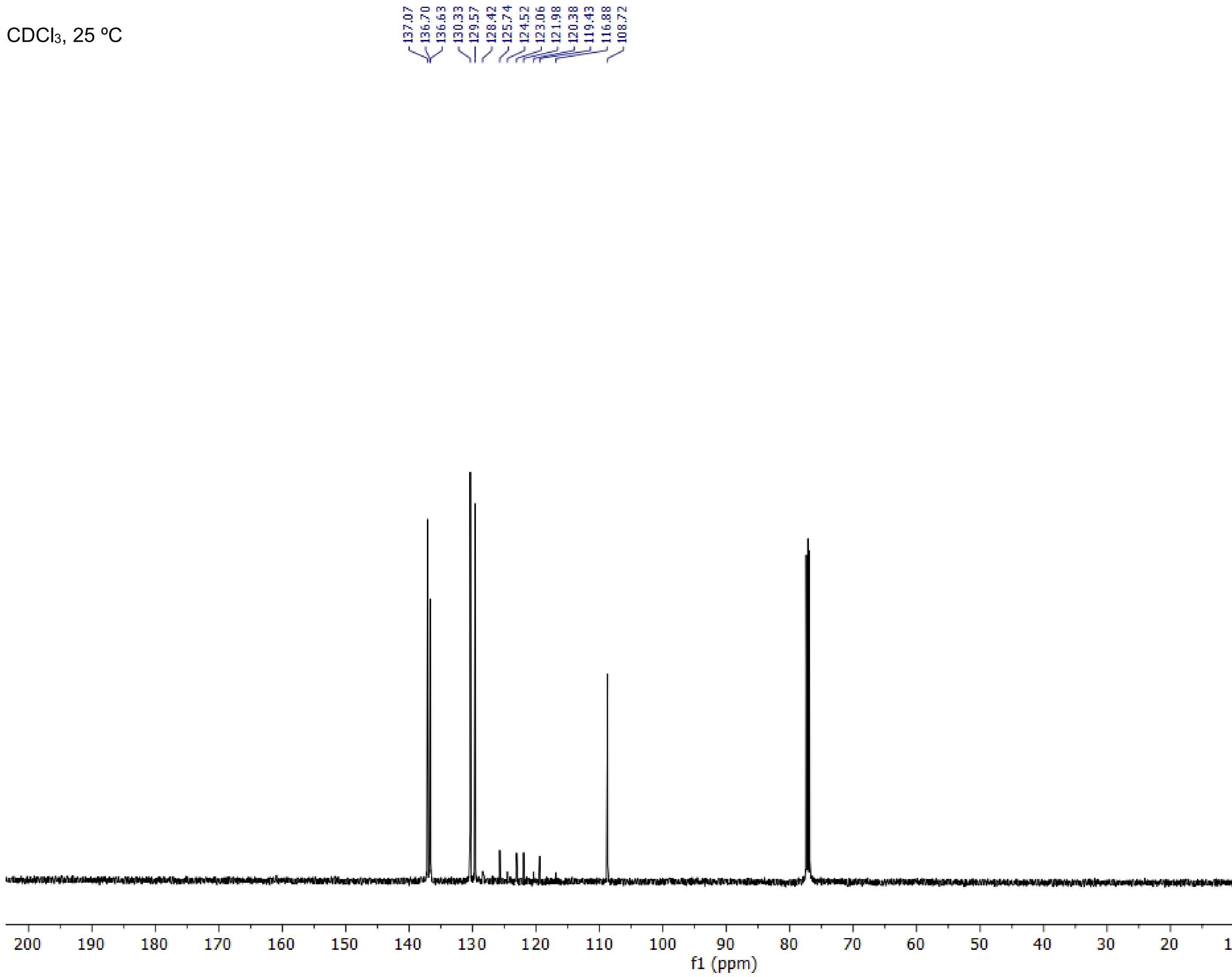

^{1}H NMR of *S*-(trifluoromethyl)thianthrenium tetrafluoroborate (1')

CD3CN, 25 ºC

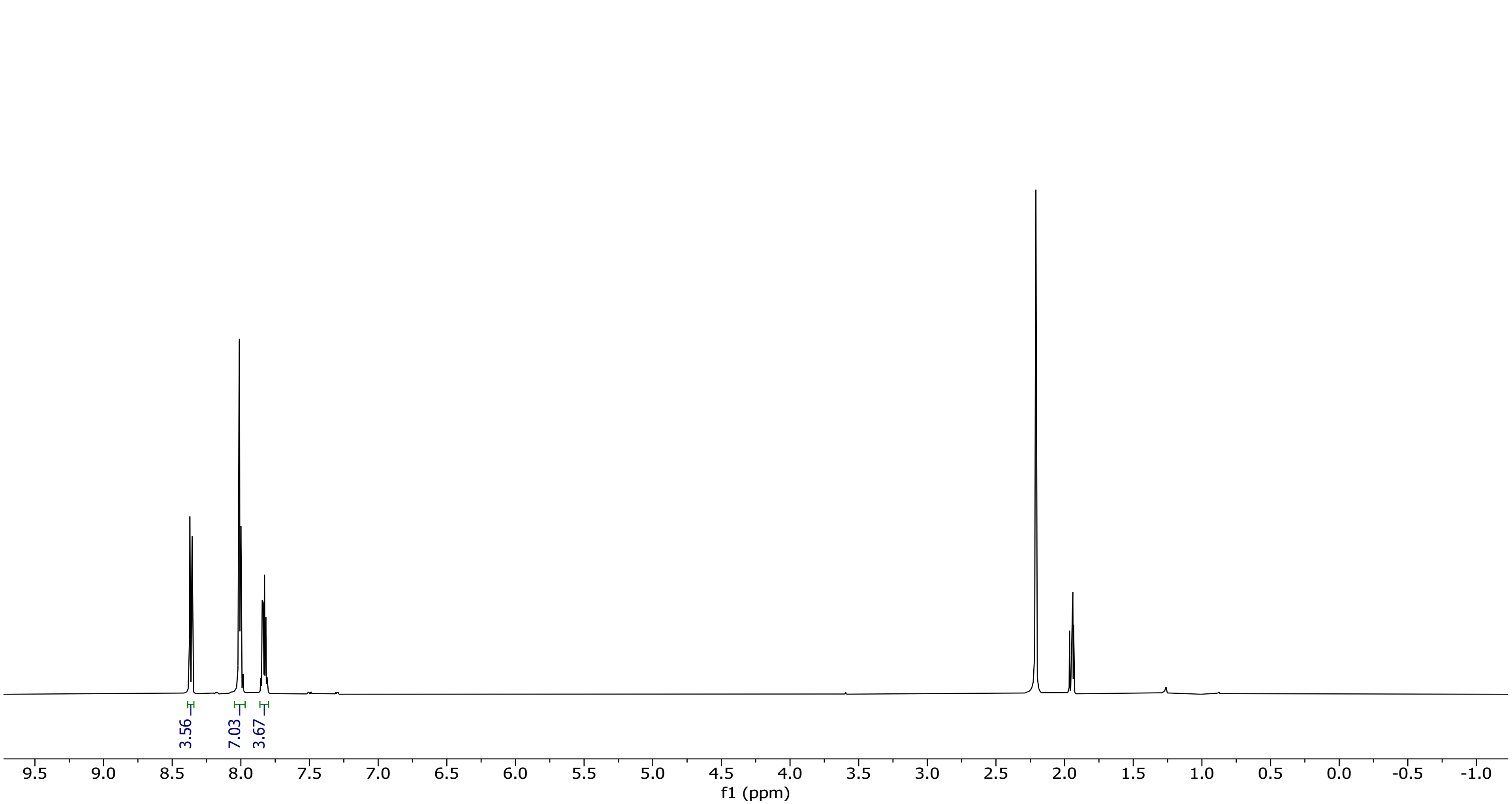

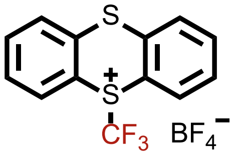

(1', TTCF3+BF4−)

^{19}F NMR of *S*-(trifluoromethyl)thianthrenium tetrafluoroborate (1')

CD$_3$CN, 25 ºC

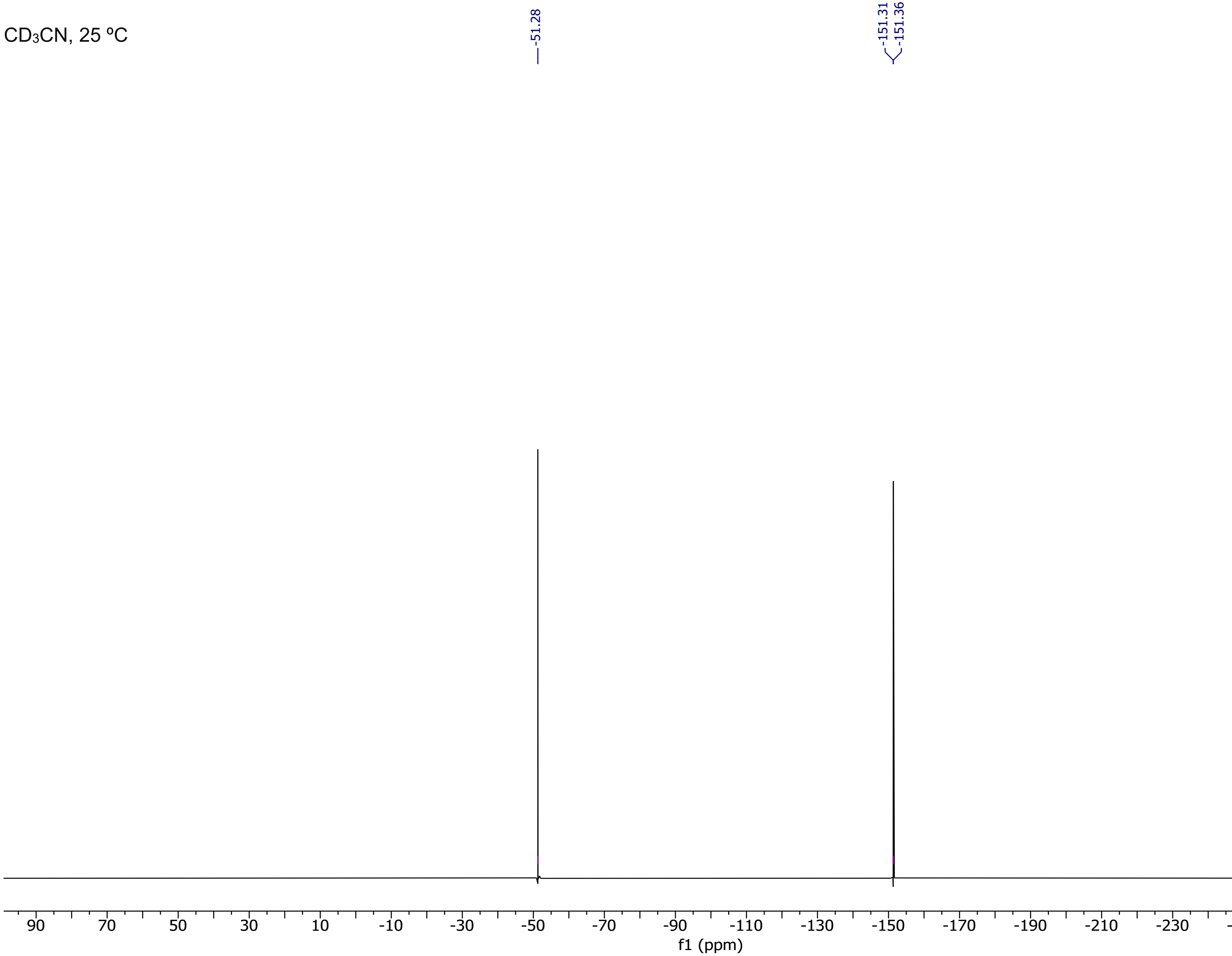

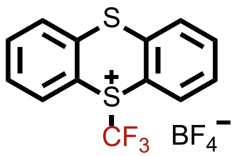

(1', TTCF$_3^+$BF$_4^-$)

^{13}C NMR of *S*-(trifluoromethyl)thianthrenium tetrafluoroborate (1')

CD3CN, 25 ºC

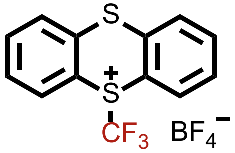

(1', $TTCF_3^+BF_4^-$)

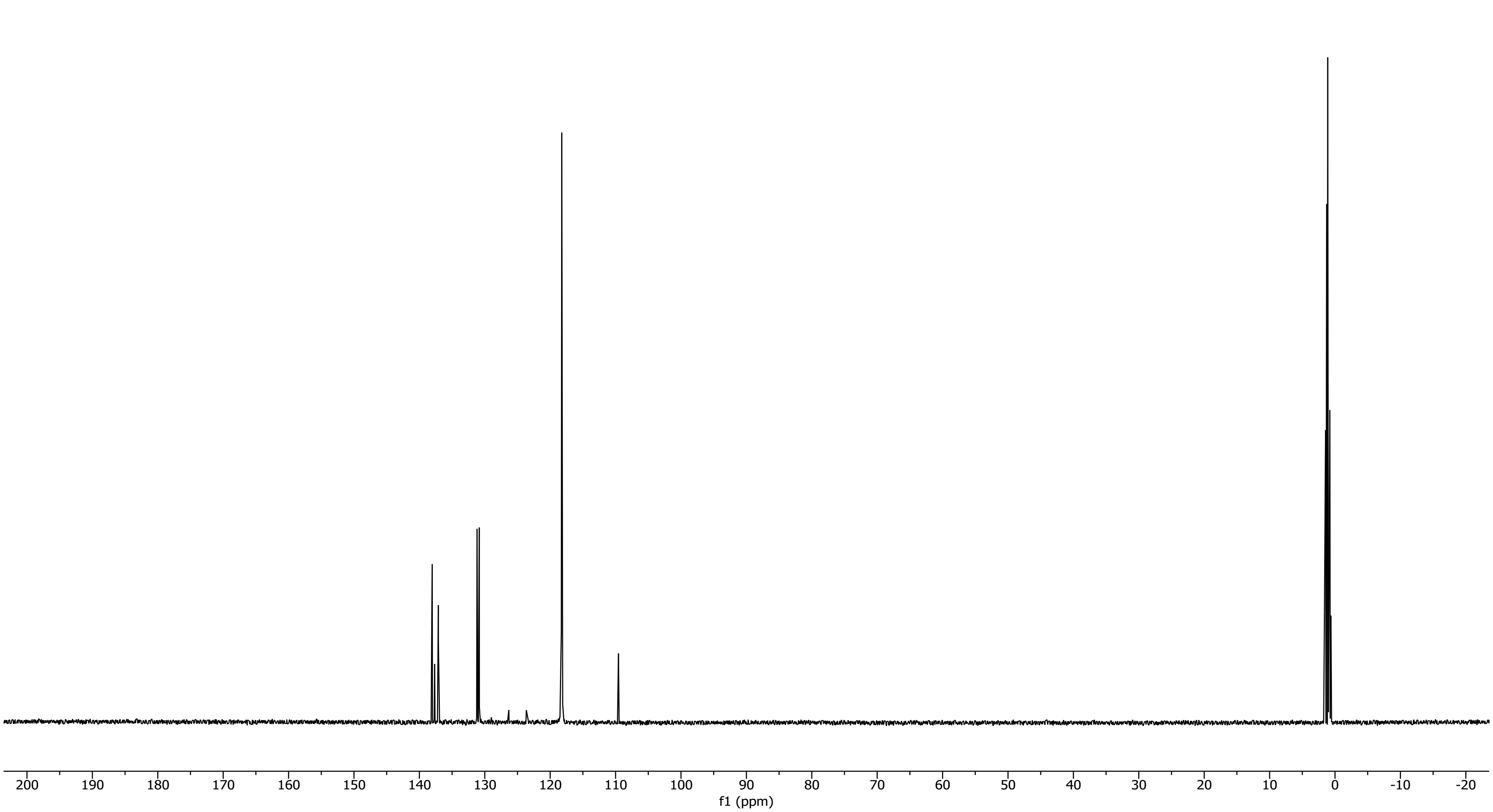

^{1}H NMR of racecadotril derivative S19

CDCl3, 25 ºC

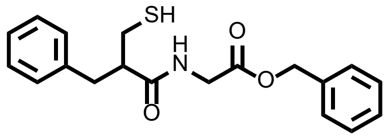

S19

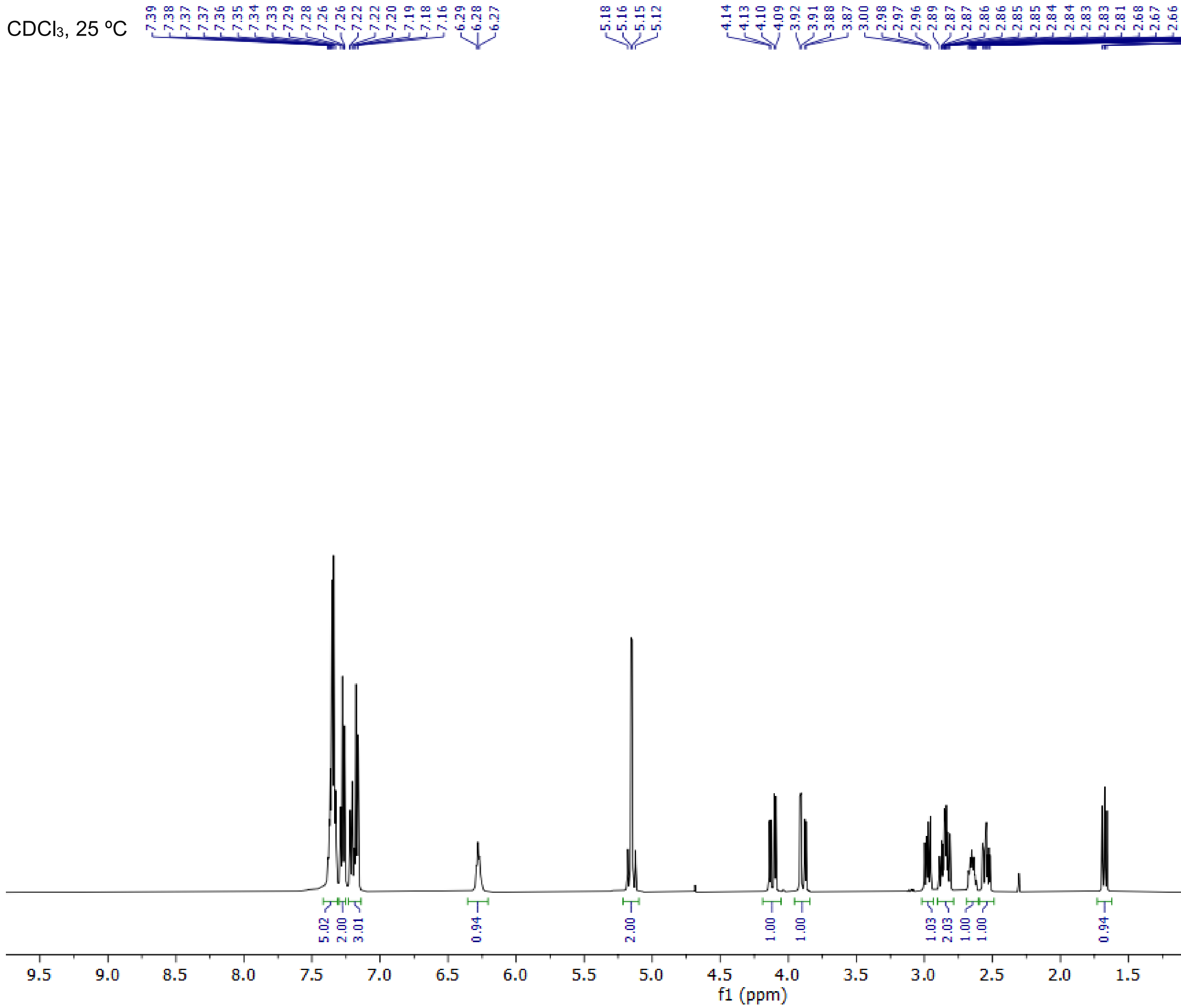

^{13}C NMR of racecadotril derivative S19

$CDCl_3$, 25 ºC

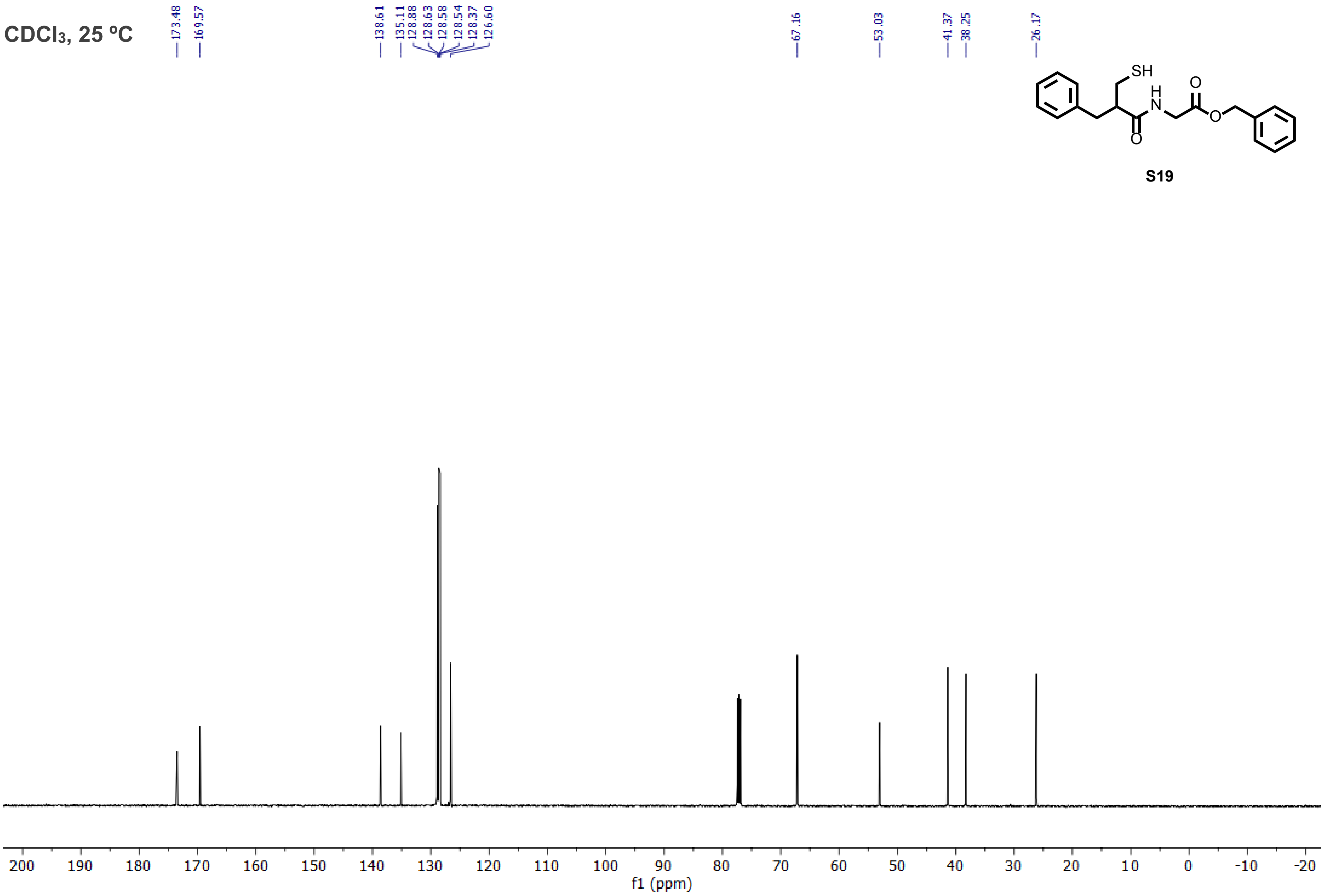

^{1}H NMR of *N*-(4-chloro-2-fluorophenyl)pent-4-enamide (S25)

CDCl$_3$, 25 ºC

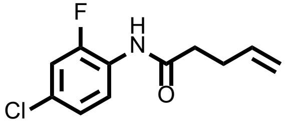

S25

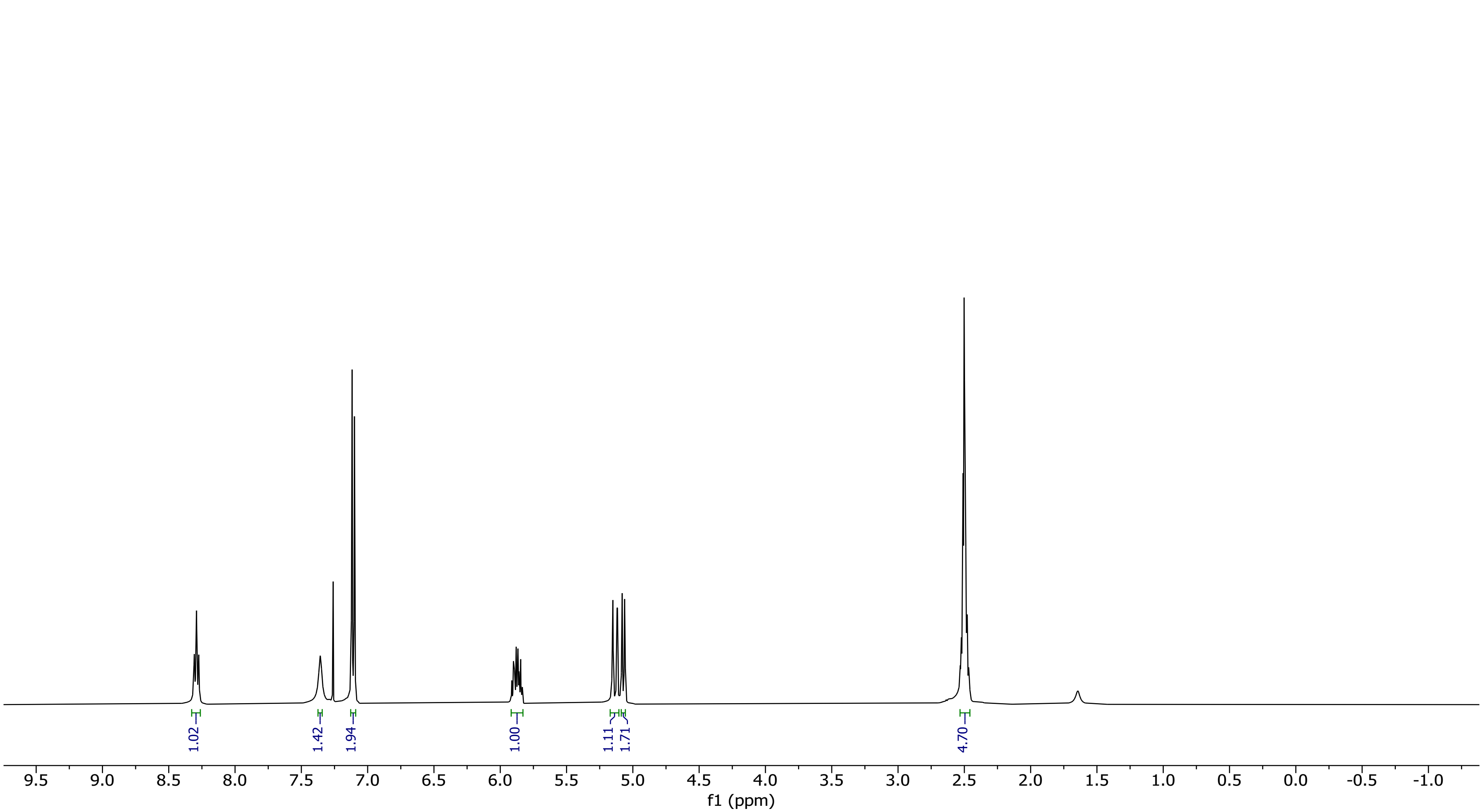

^{19}F NMR of *N*-(4-chloro-2-fluorophenyl)pent-4-enamide (S25)

$CDCl_3$, 25 ºC

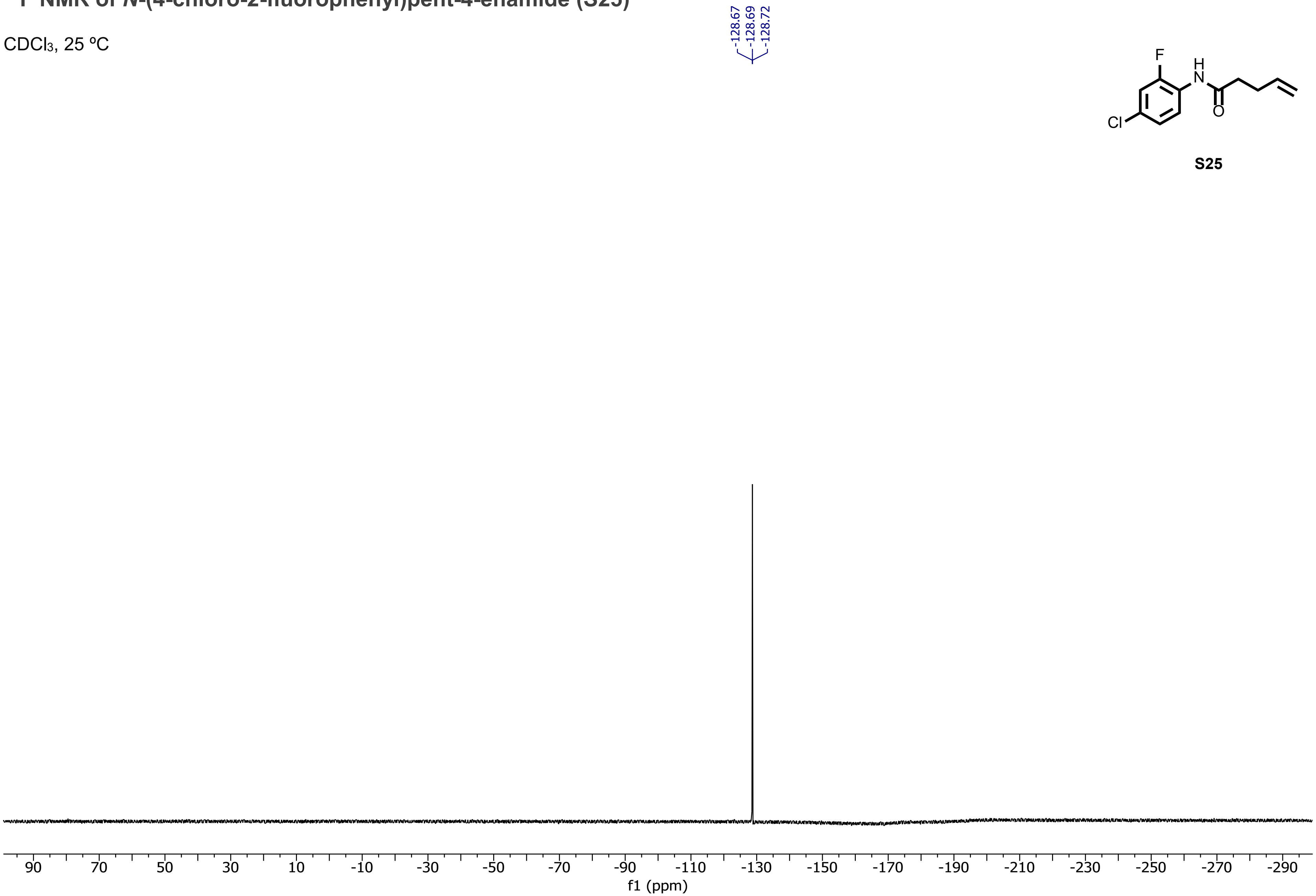

S25

^{13}C NMR of *N*-(4-chloro-2-fluorophenyl)pent-4-enamide (S25)

$CDCl_3$, 25 ºC

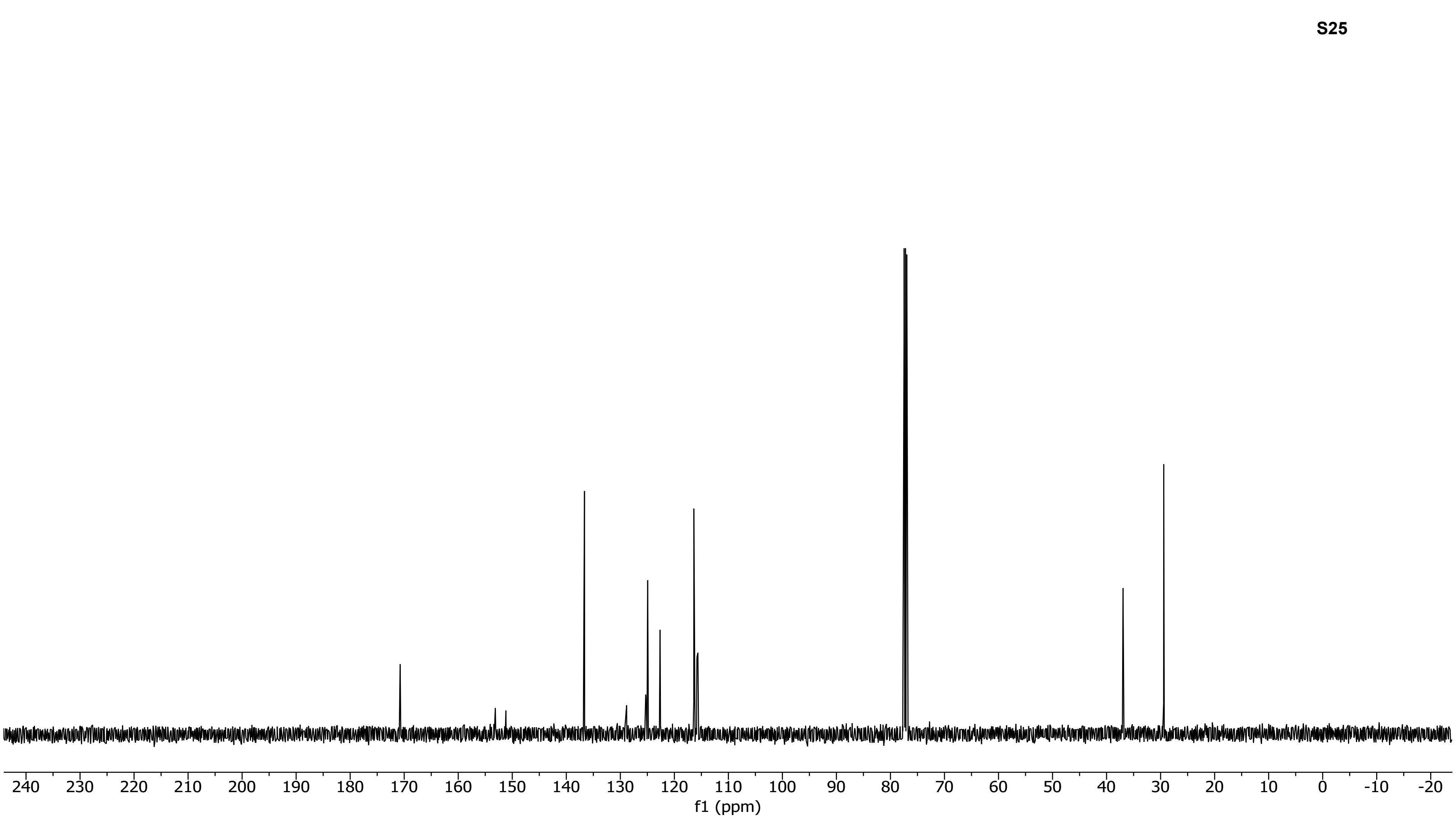

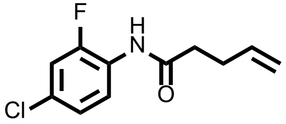

S25

^{1}H NMR of (1*S*)-10-camphorsulfonamide (S26)

CDCl$_3$, 25 ºC

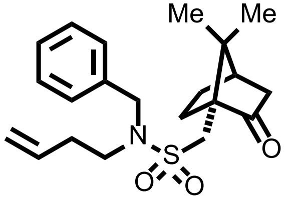

S26

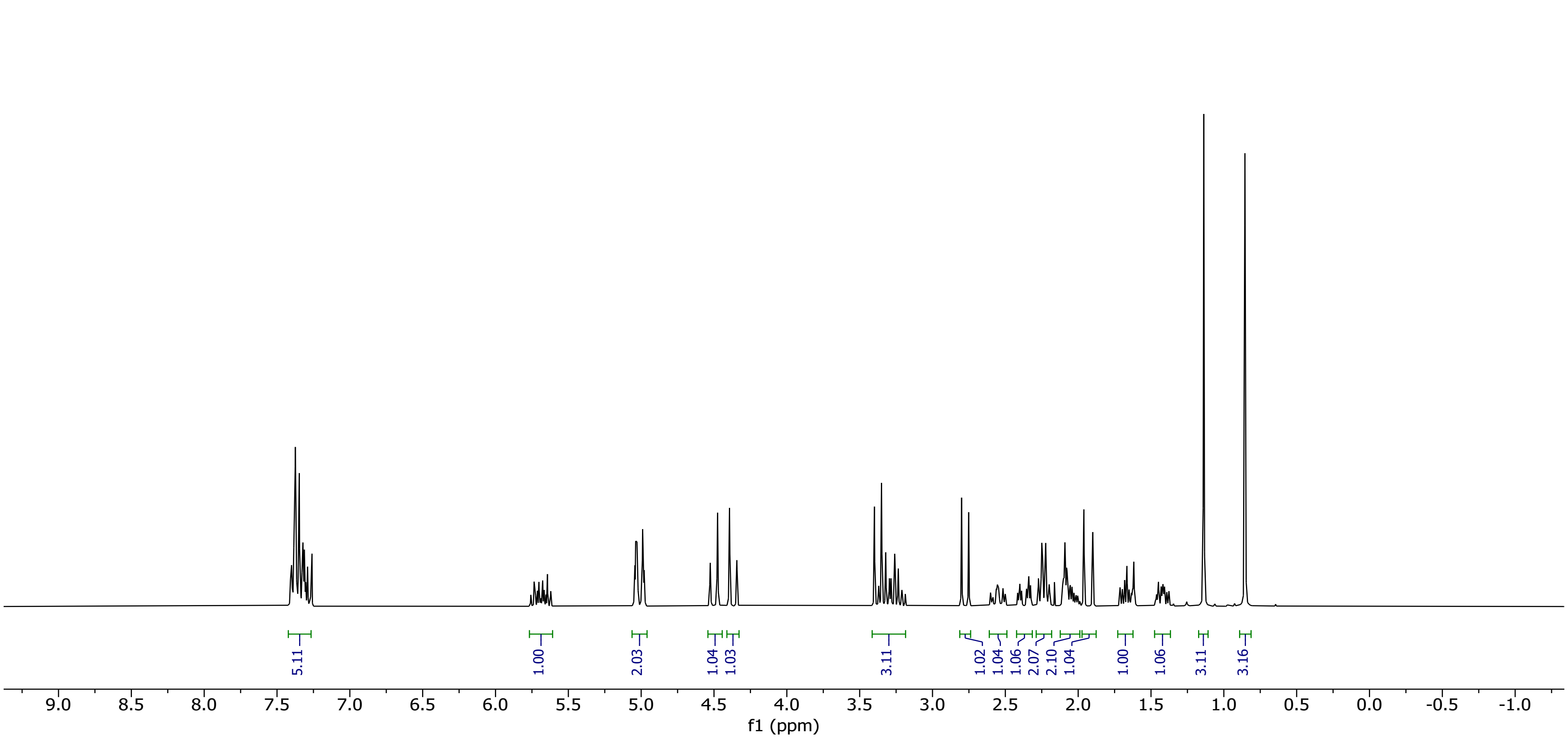

^{13}C NMR of (1*S*)-10-camphorsulfonamide (S26)

$CDCl_3$, 25 ºC

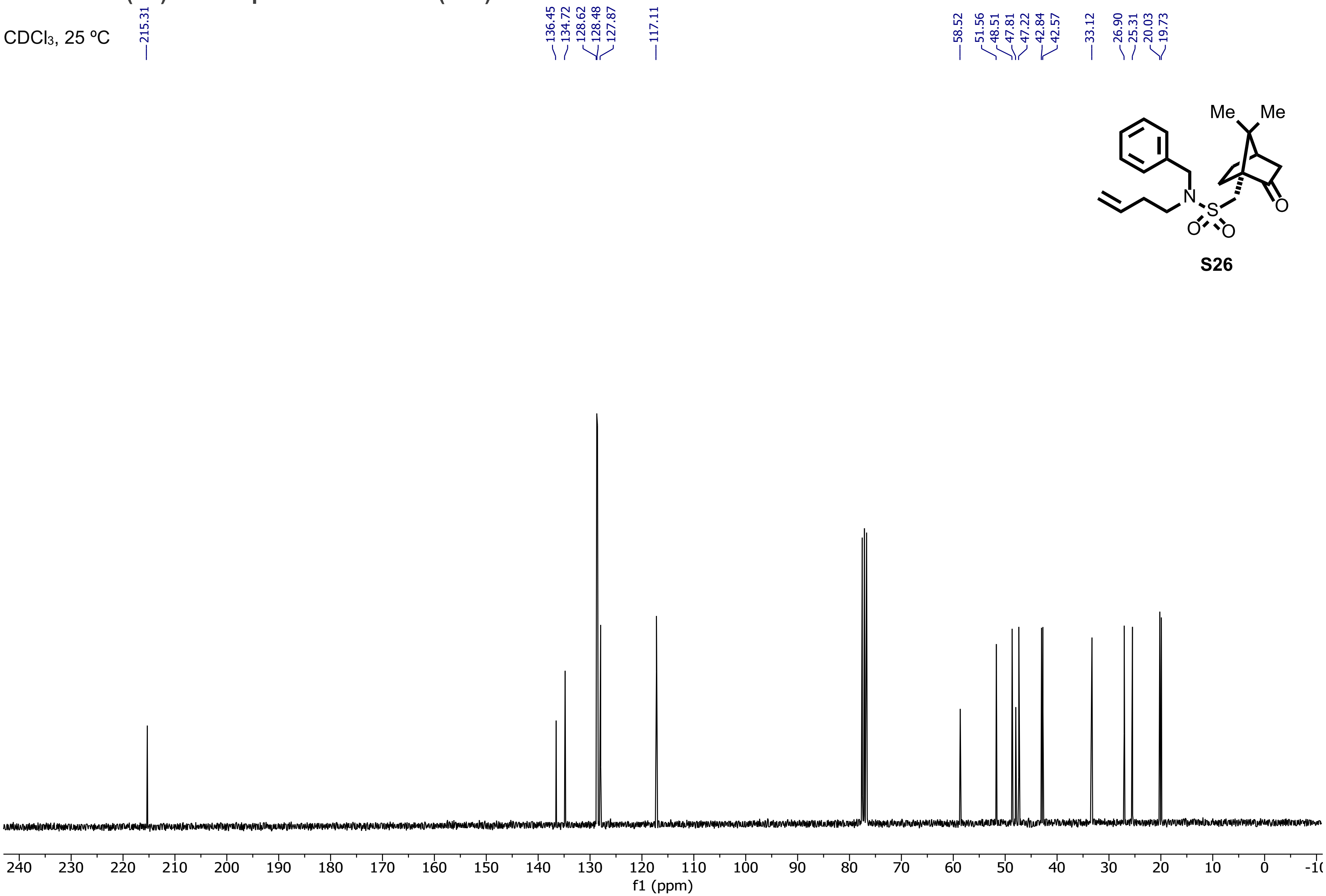

^{1}H NMR of Fmoc-L-Nle-OH derivative S27

CDCl3, 25 ºC

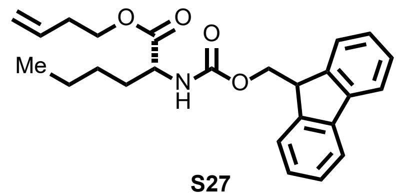

S27

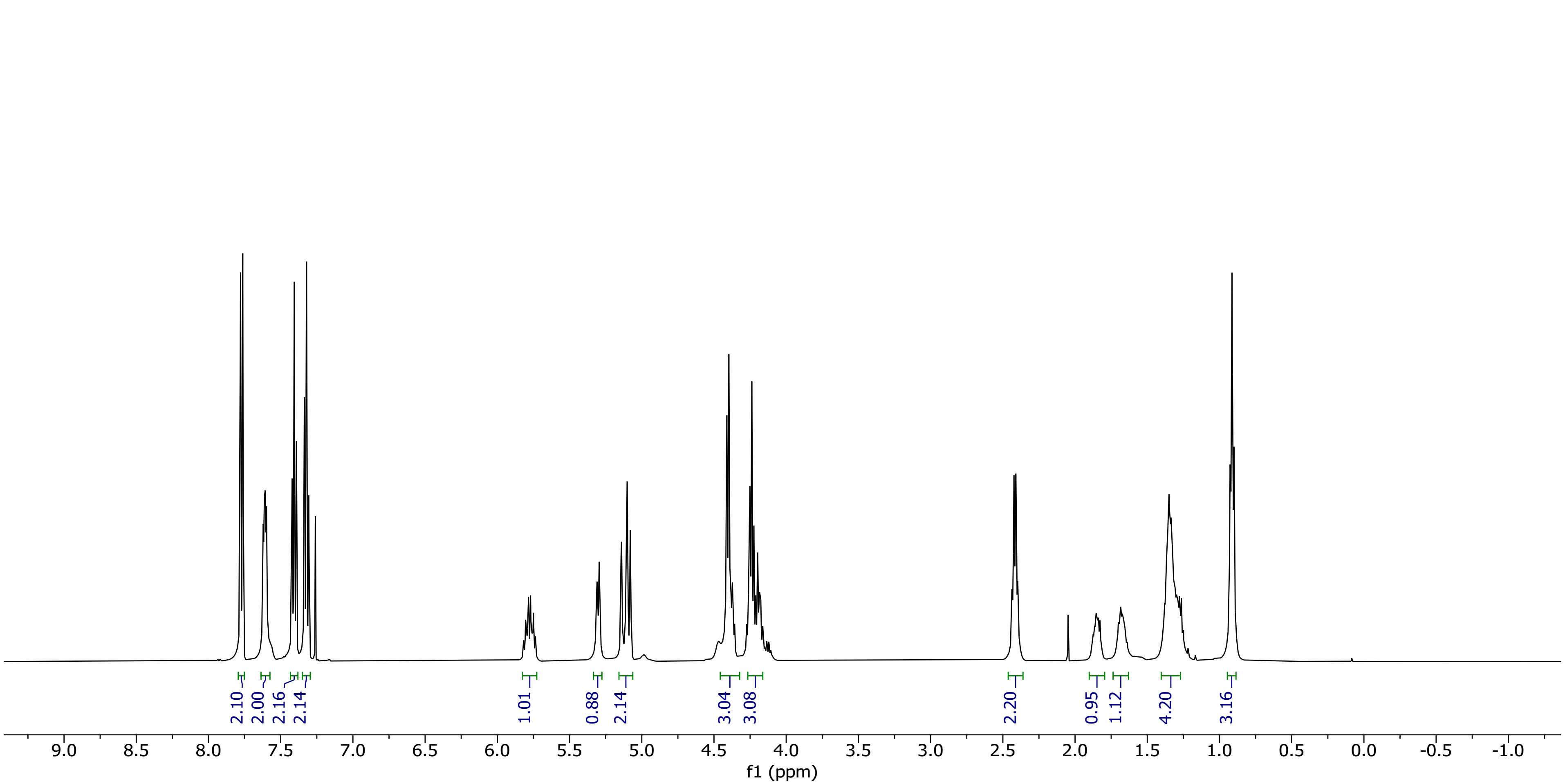

^{13}C NMR of Fmoc-L-Nle-OH derivative S27

CDCl₃, 25 ºC

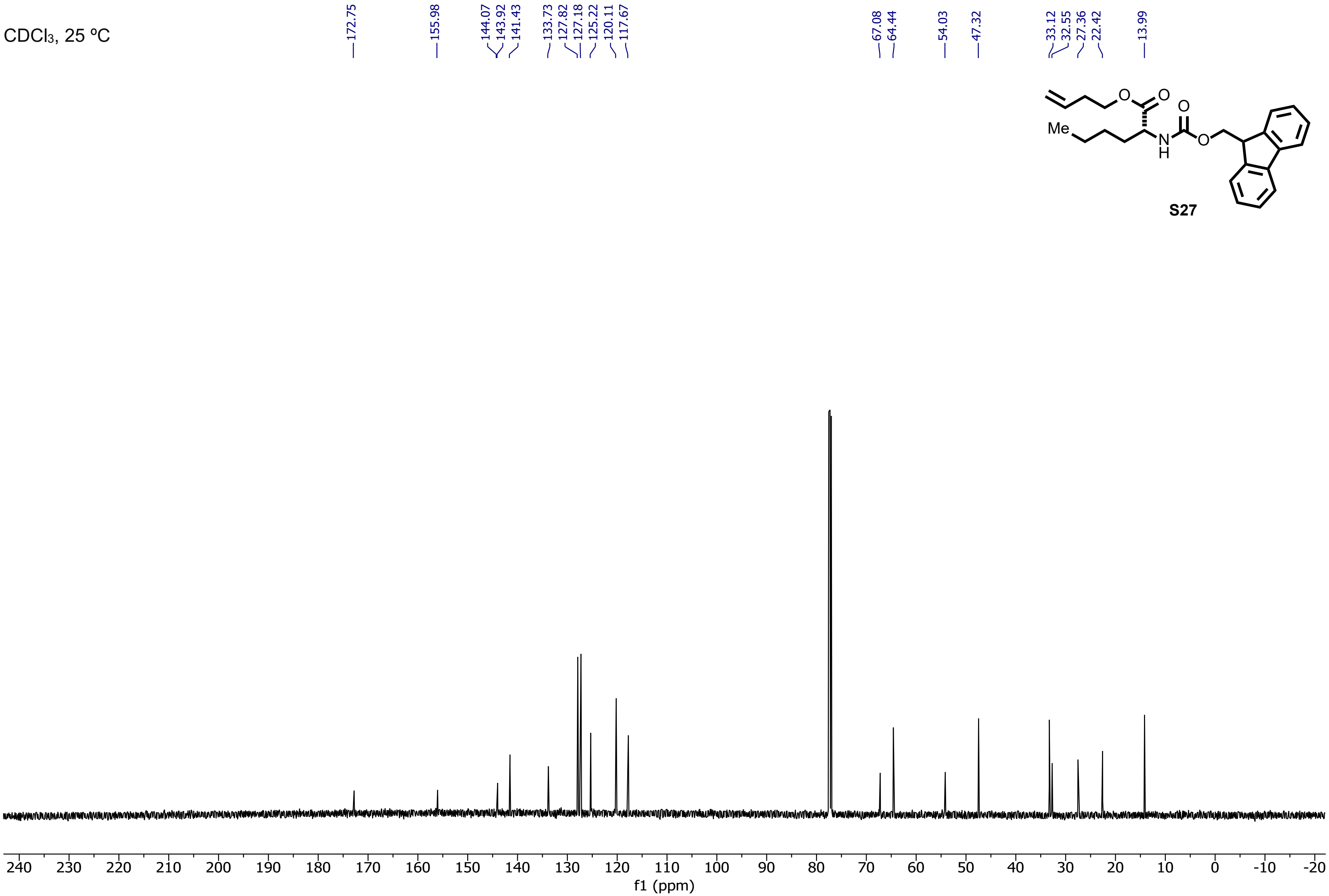

^{1}H NMR of allyl adapalene derivative S32

CDCl$_3$, 25 ºC

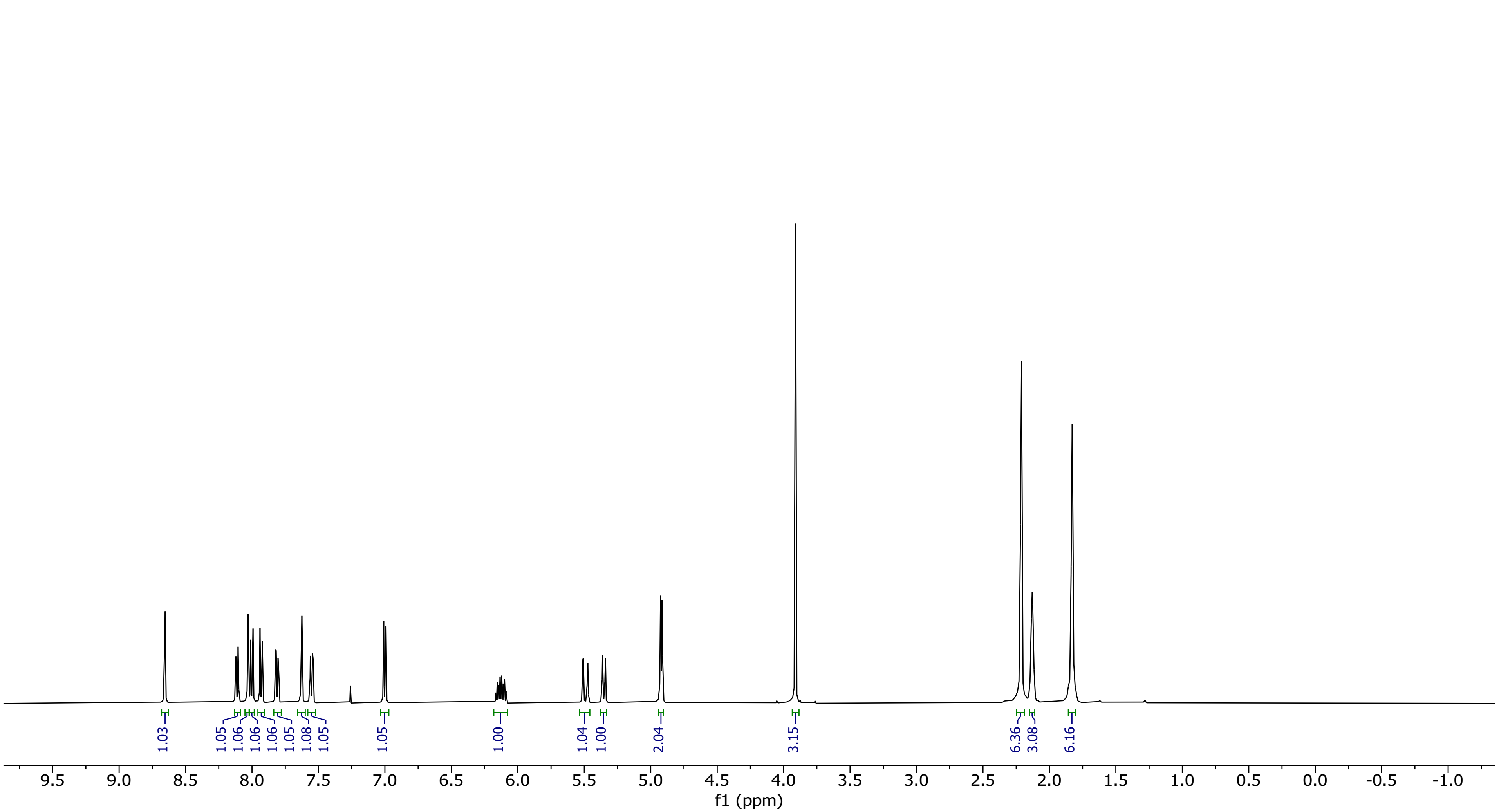

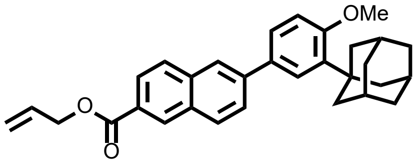

S32

^{13}C NMR of allyl adapalene derivative S32

$CDCl_3$, 25 ºC

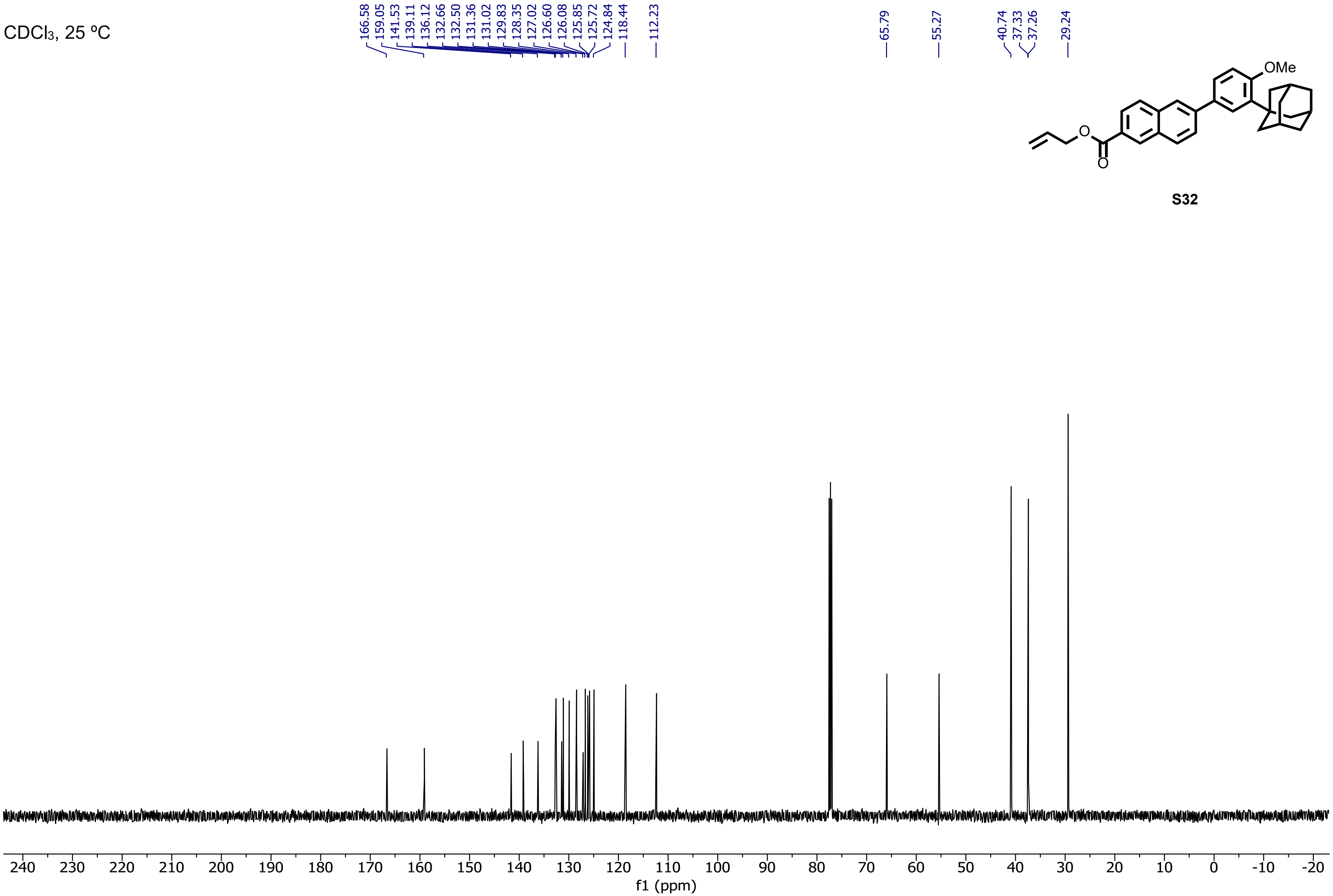

S32

^{1}H NMR of 4-(trifluoromethyl)-1,1'-biphenyl (3)

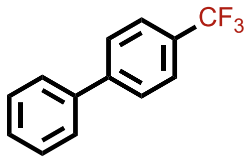

3

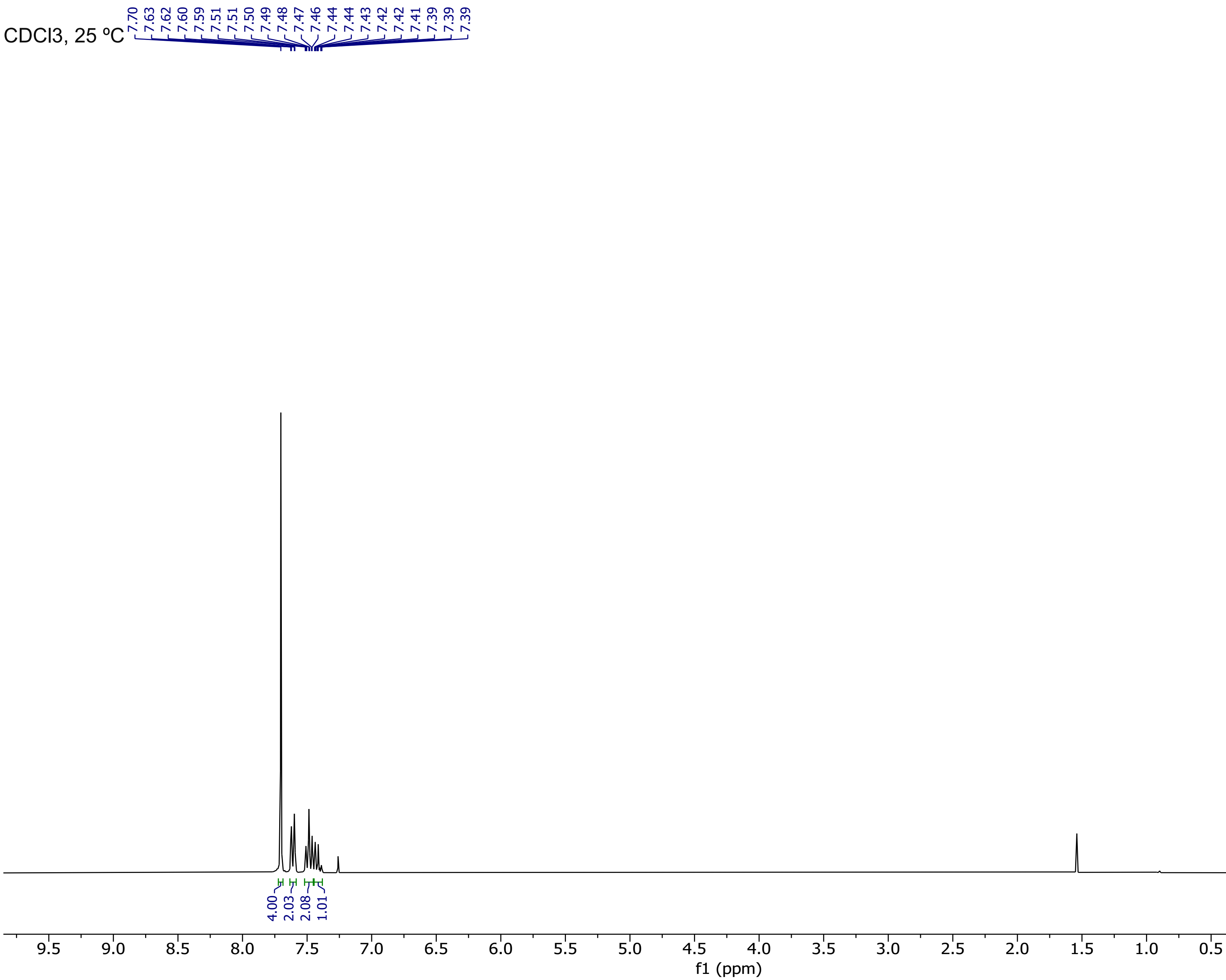

^{19}F NMR of 4-(trifluoromethyl)-1,1'-biphenyl (3)

$CDCl_3$, 25 ºC

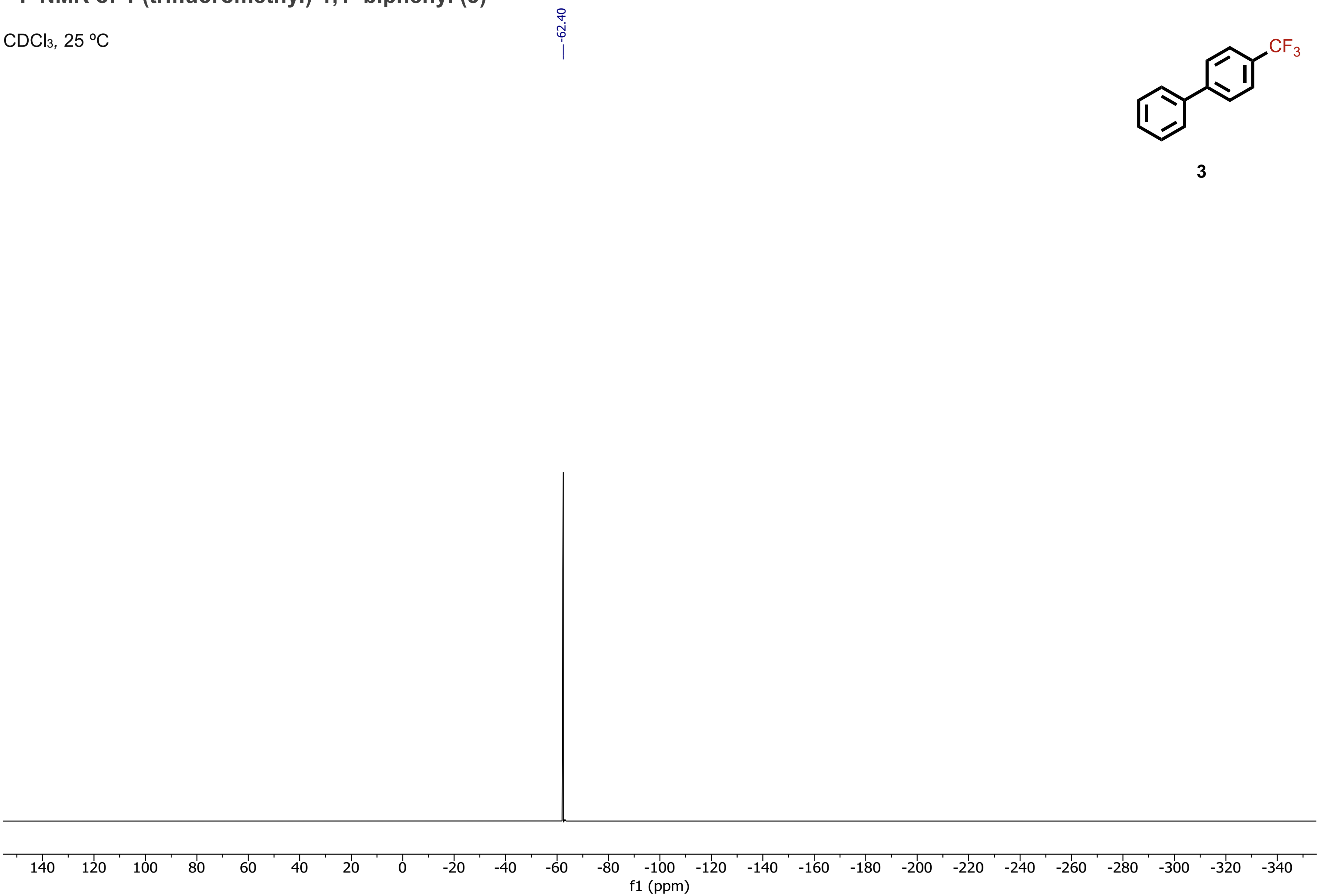

^{13}C NMR of 4-(trifluoromethyl)-1,1'-biphenyl (3)

$CDCl_3$, 25 ºC

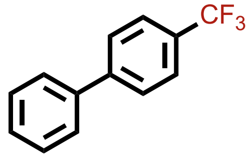

3

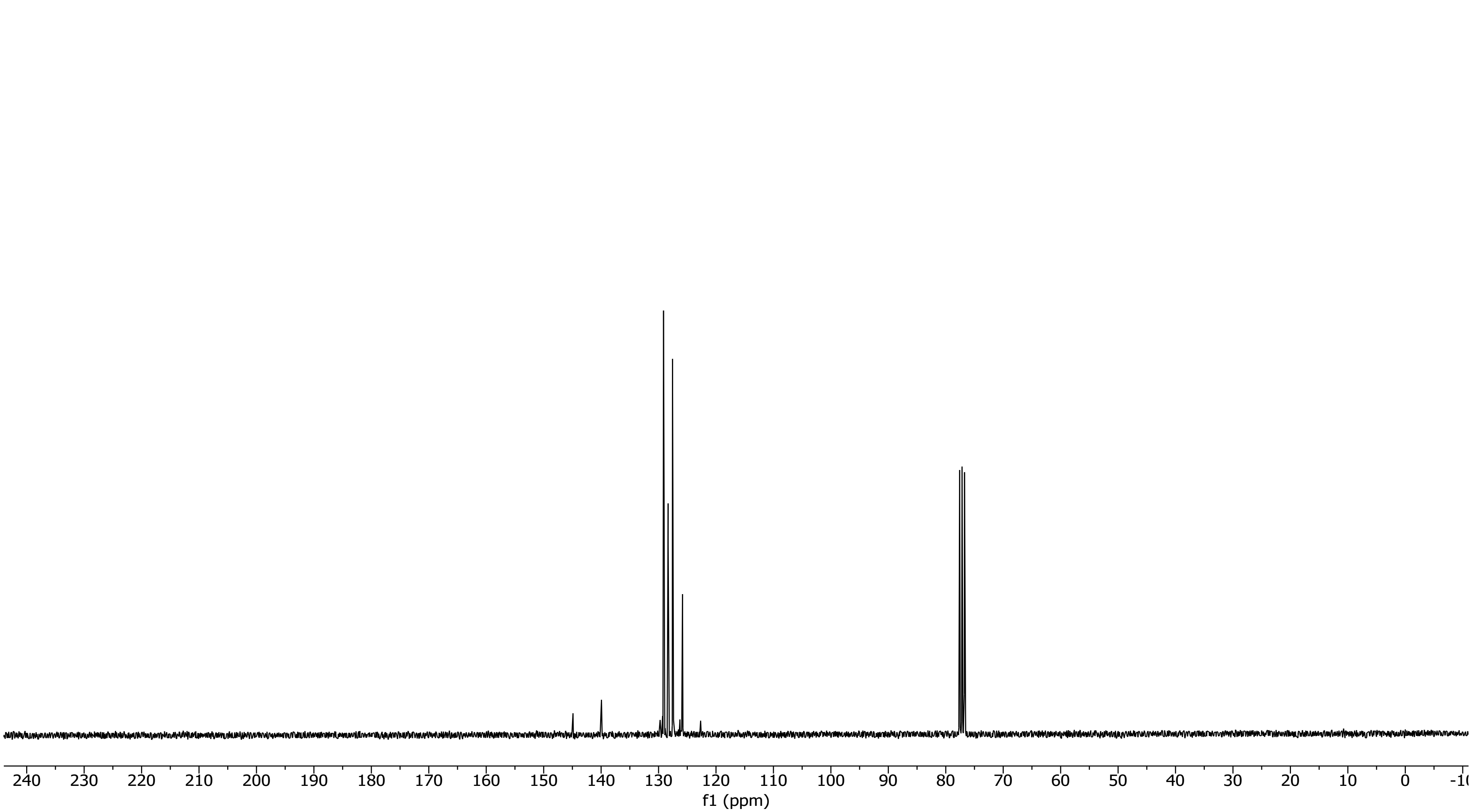

^{1}H NMR of 3-(trifluoromethyl)quinoline (4)

CDCl$_3$, 25 ºC

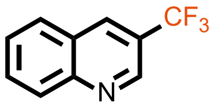

4

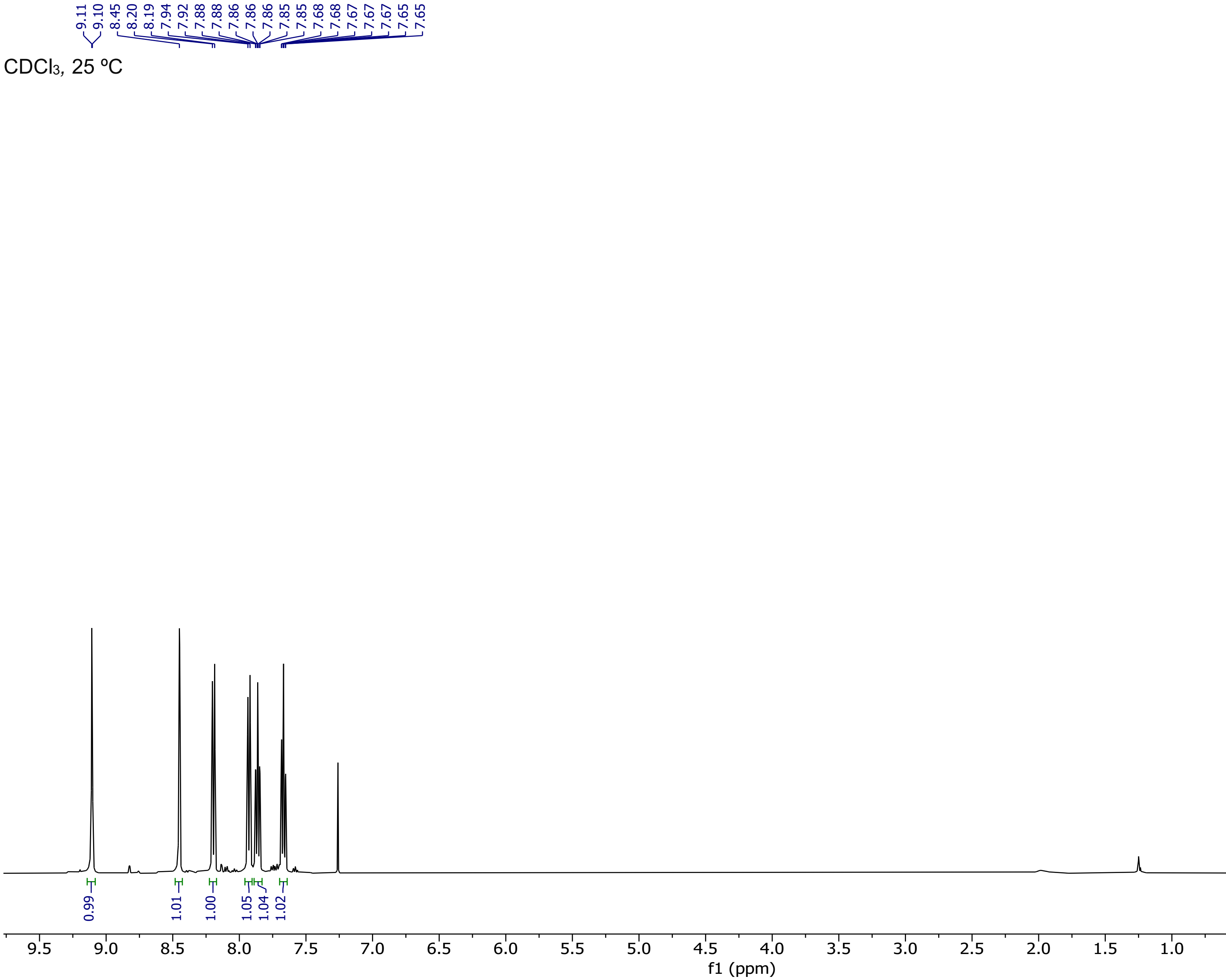

^{19}F NMR of 3-(trifluoromethyl)quinoline (4)

$CDCl_3$, 25 ºC

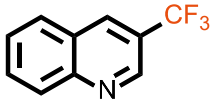

4

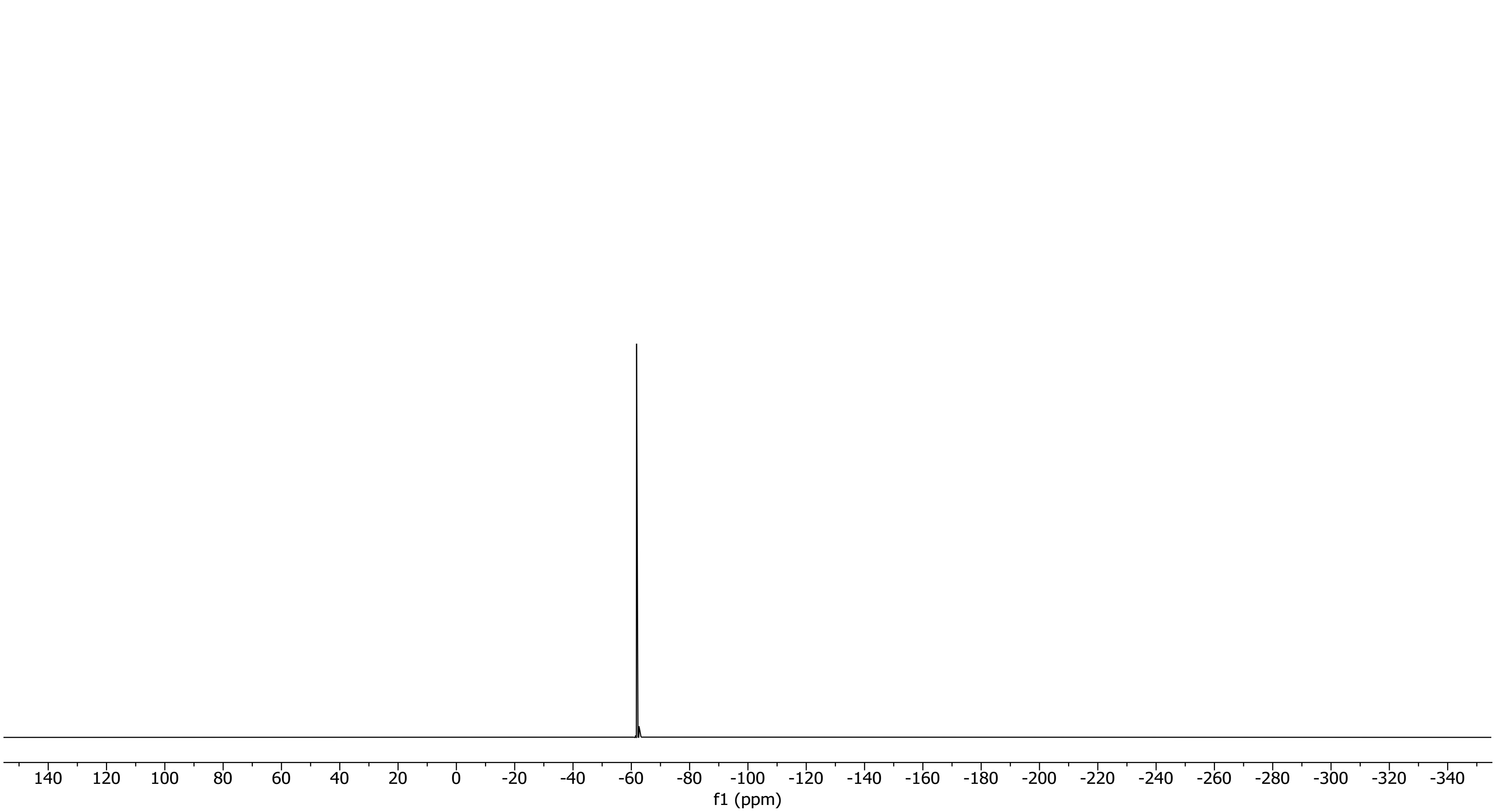

^{13}C NMR of 3-(trifluoromethyl)quinoline (4)

CDCl₃, 25 ºC

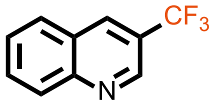

4

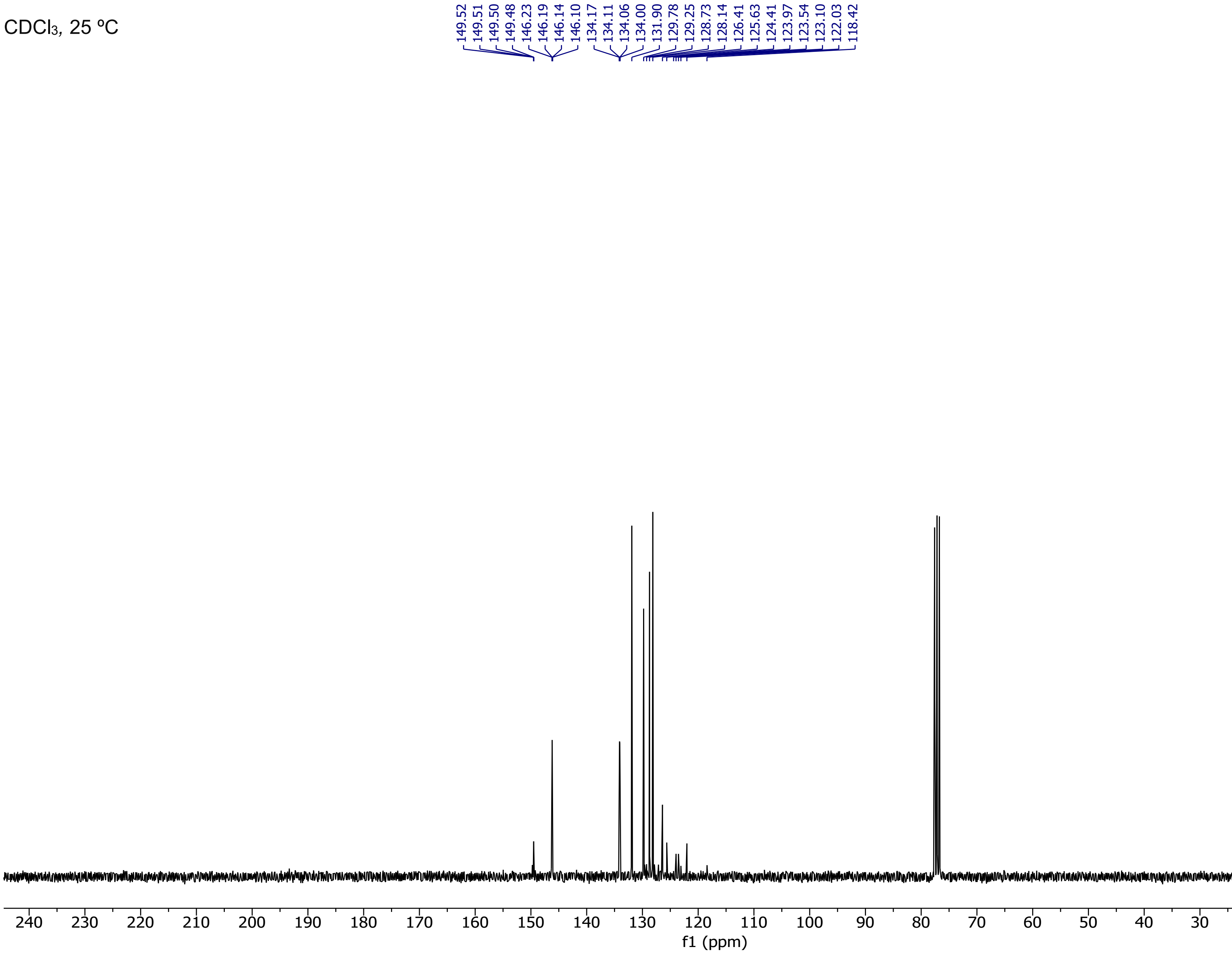

^{1}H NMR of 1-chloro-4-(trifluoromethyl)-2-((2-(trifluoromethyl)benzyl)oxy)benzene (5)

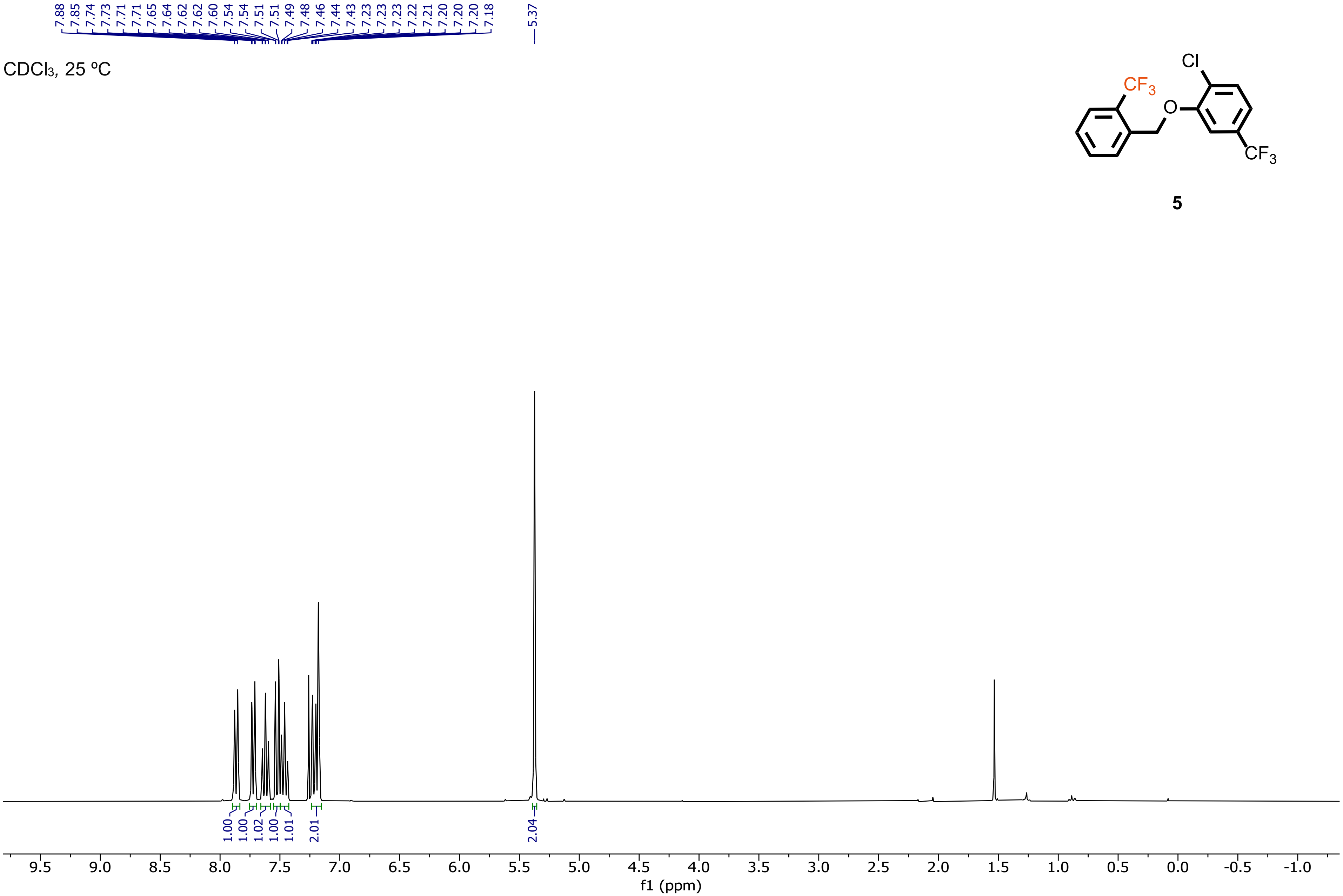

5

^{19}F NMR of 1-chloro-4-(trifluoromethyl)-2-((2-(trifluoromethyl)benzyl)oxy)benzene (5)

$CDCl_3$, 25 ºC

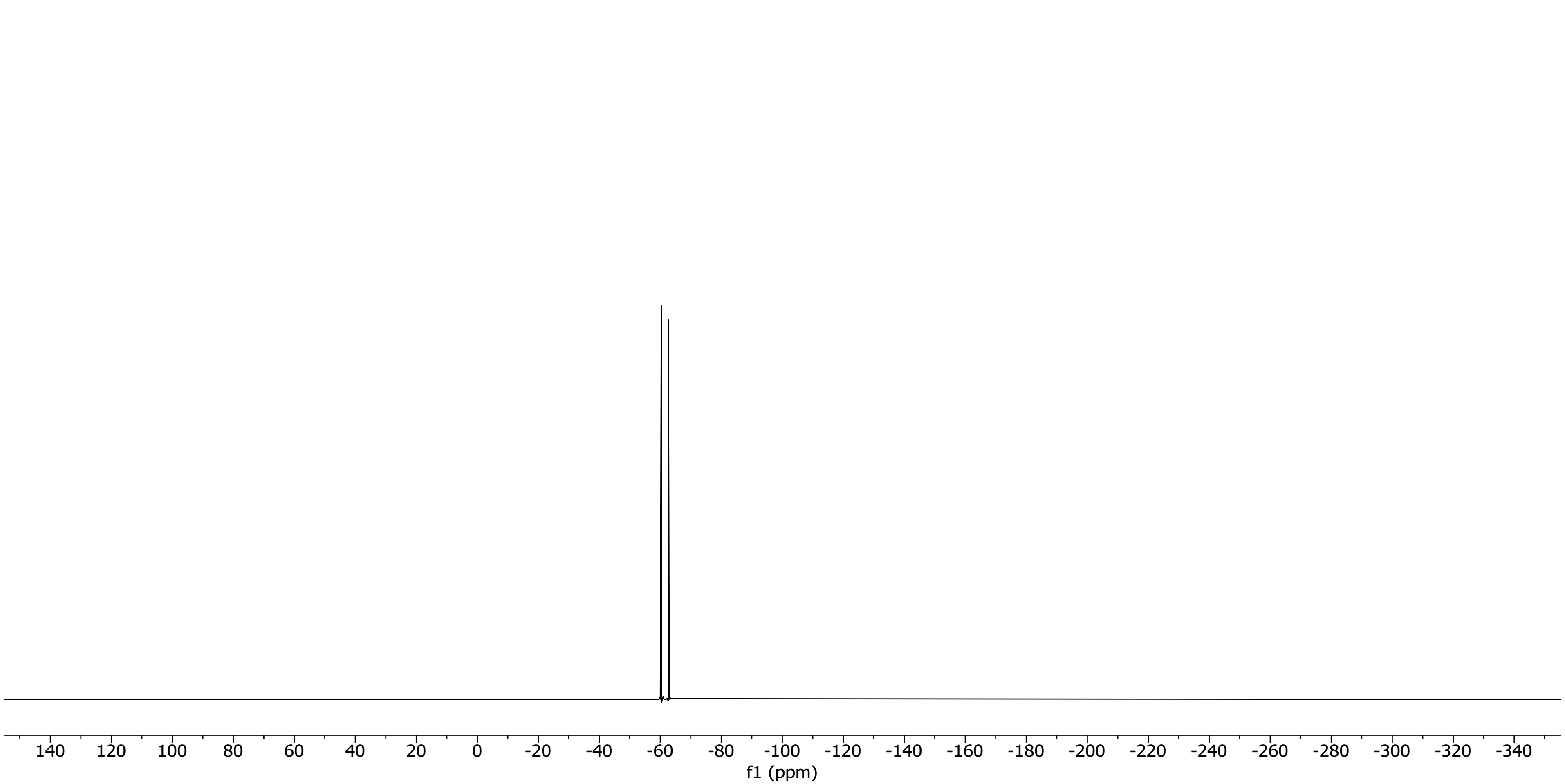

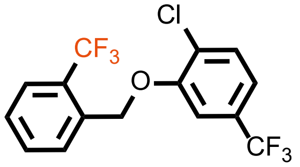

5

^{13}C NMR of 1-chloro-4-(trifluoromethyl)-2-((2-(trifluoromethyl)benzyl)oxy)benzene (5)

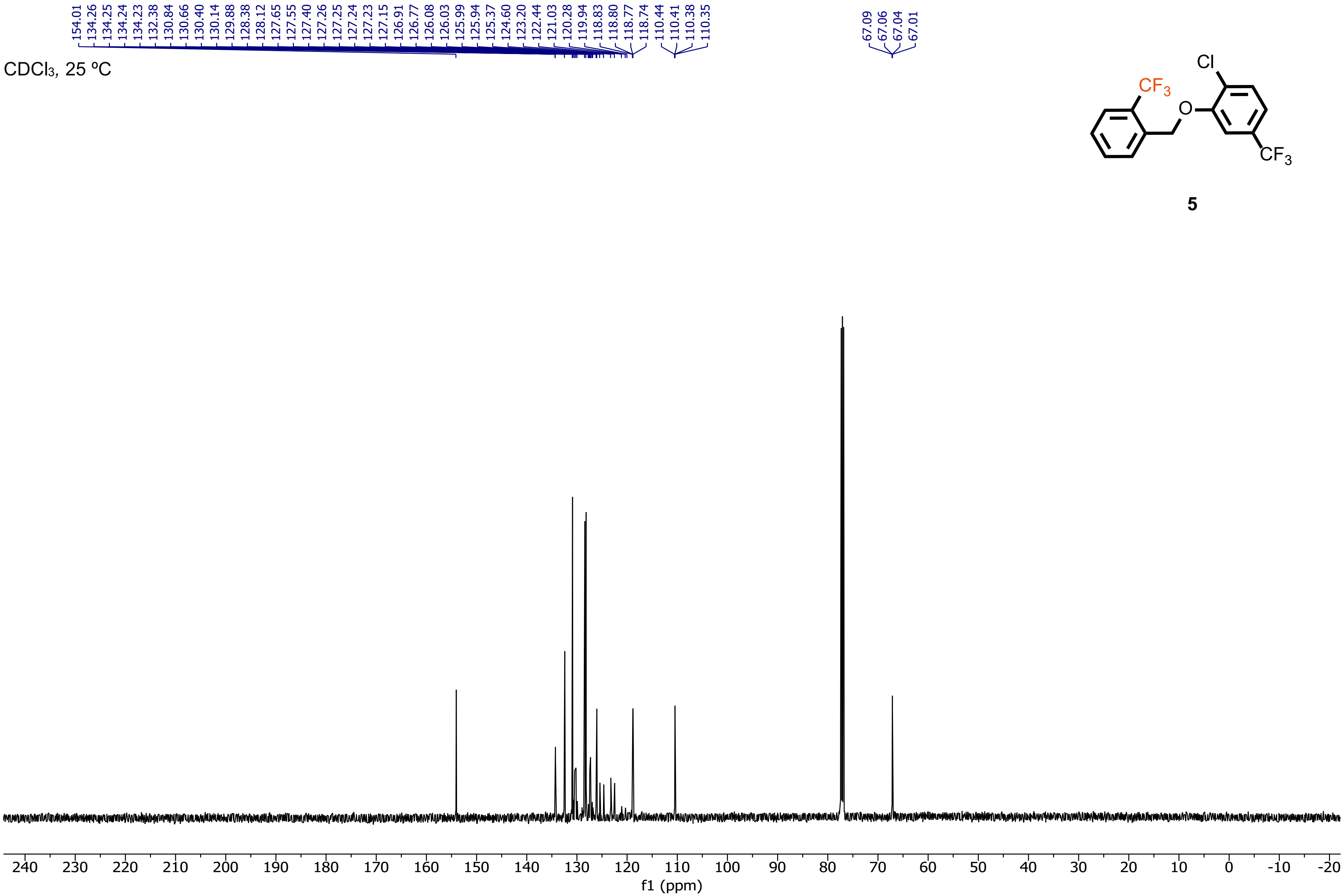

5

^{1}H NMR of 2-(trifluoromethyl)benzo[*b*]thiophene (6)

CDCl3, 25 ºC

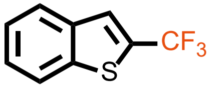

6

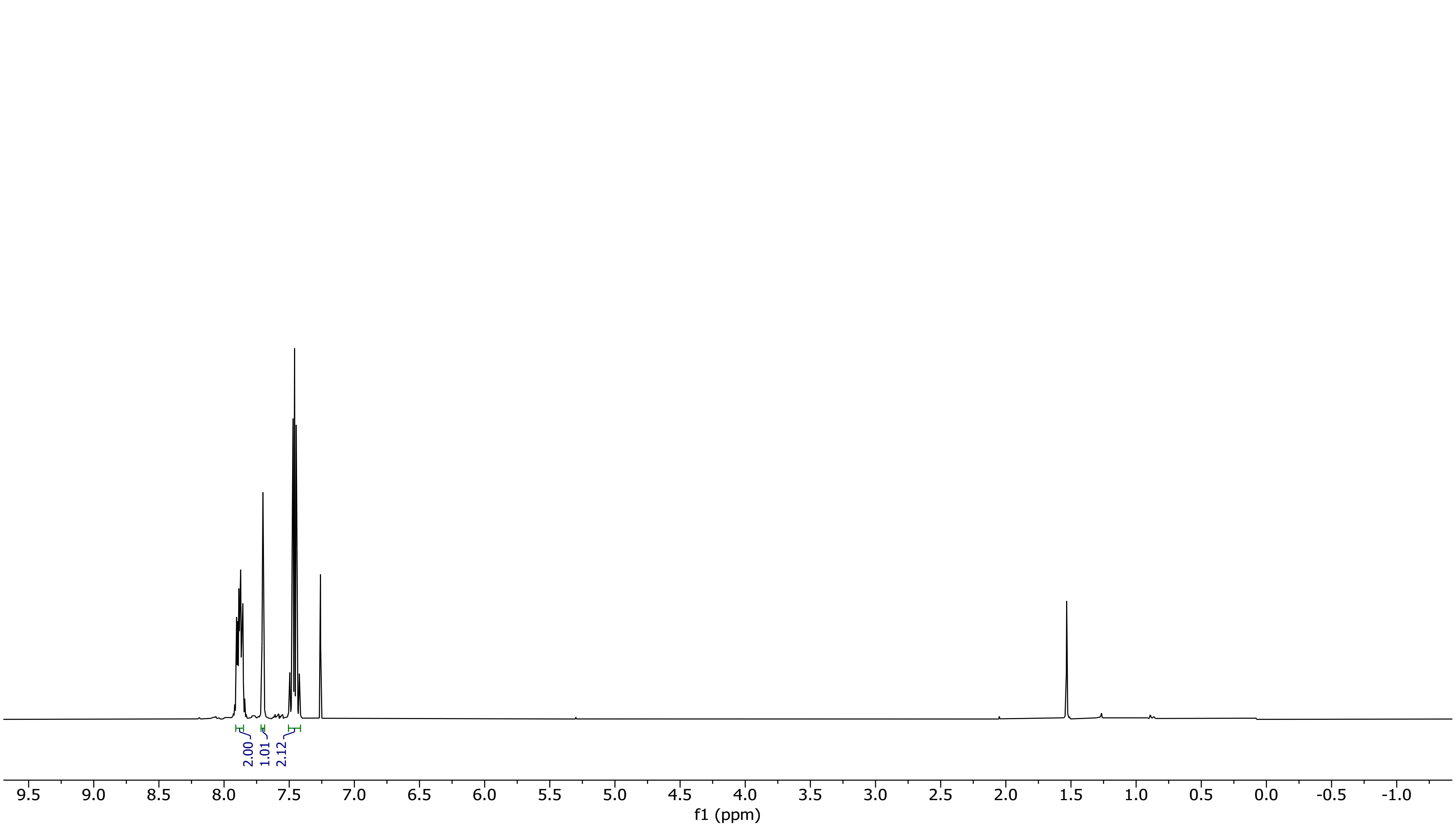

^{19}F NMR of 2-(trifluoromethyl)benzo[*b*]thiophene (6)

CDCl$_3$, 25 ºC

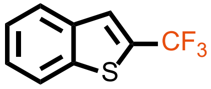

6

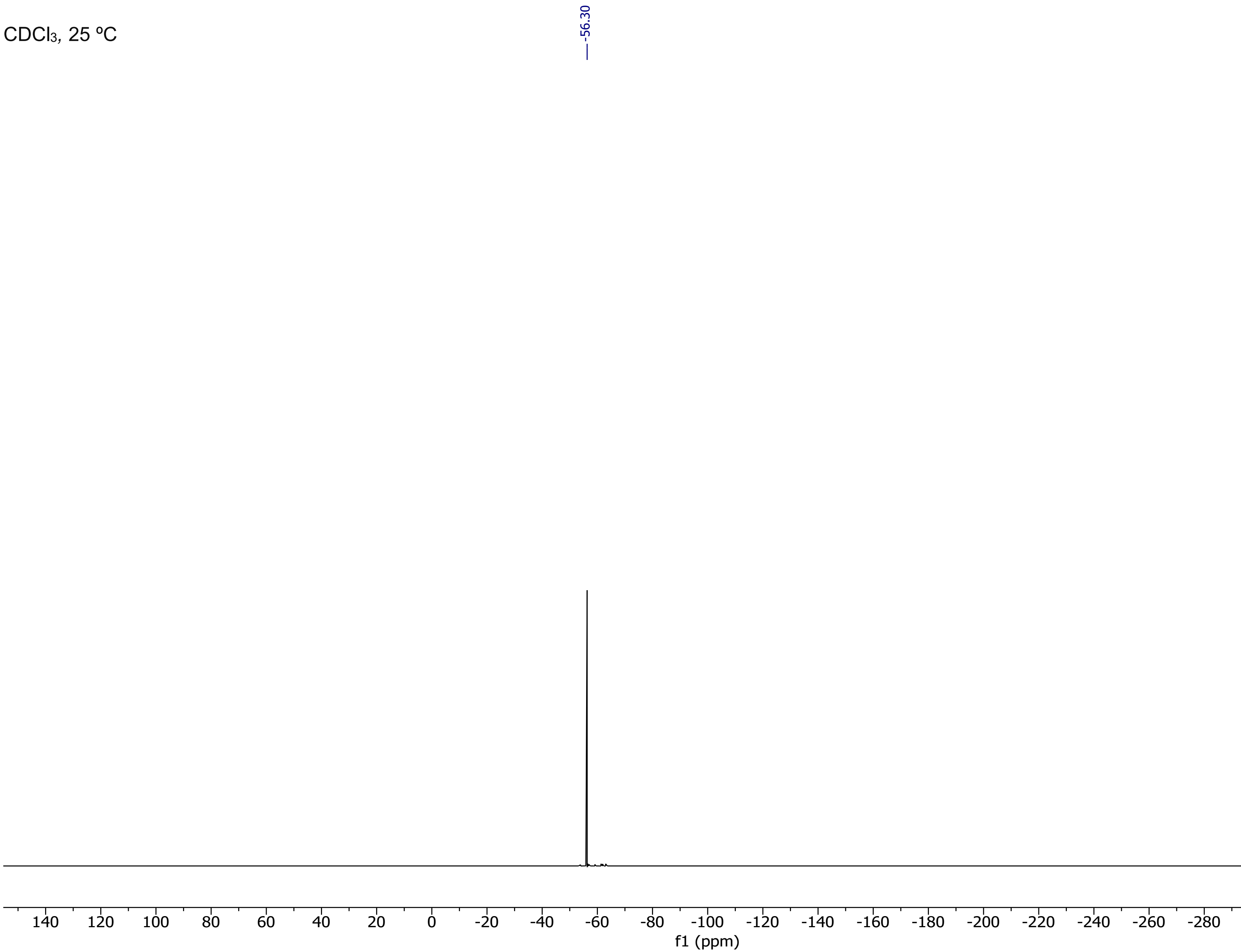

^{13}C NMR of 2-(trifluoromethyl)benzo[*b*]thiophene (6)

$CDCl_3$, 25 ºC

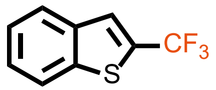

6

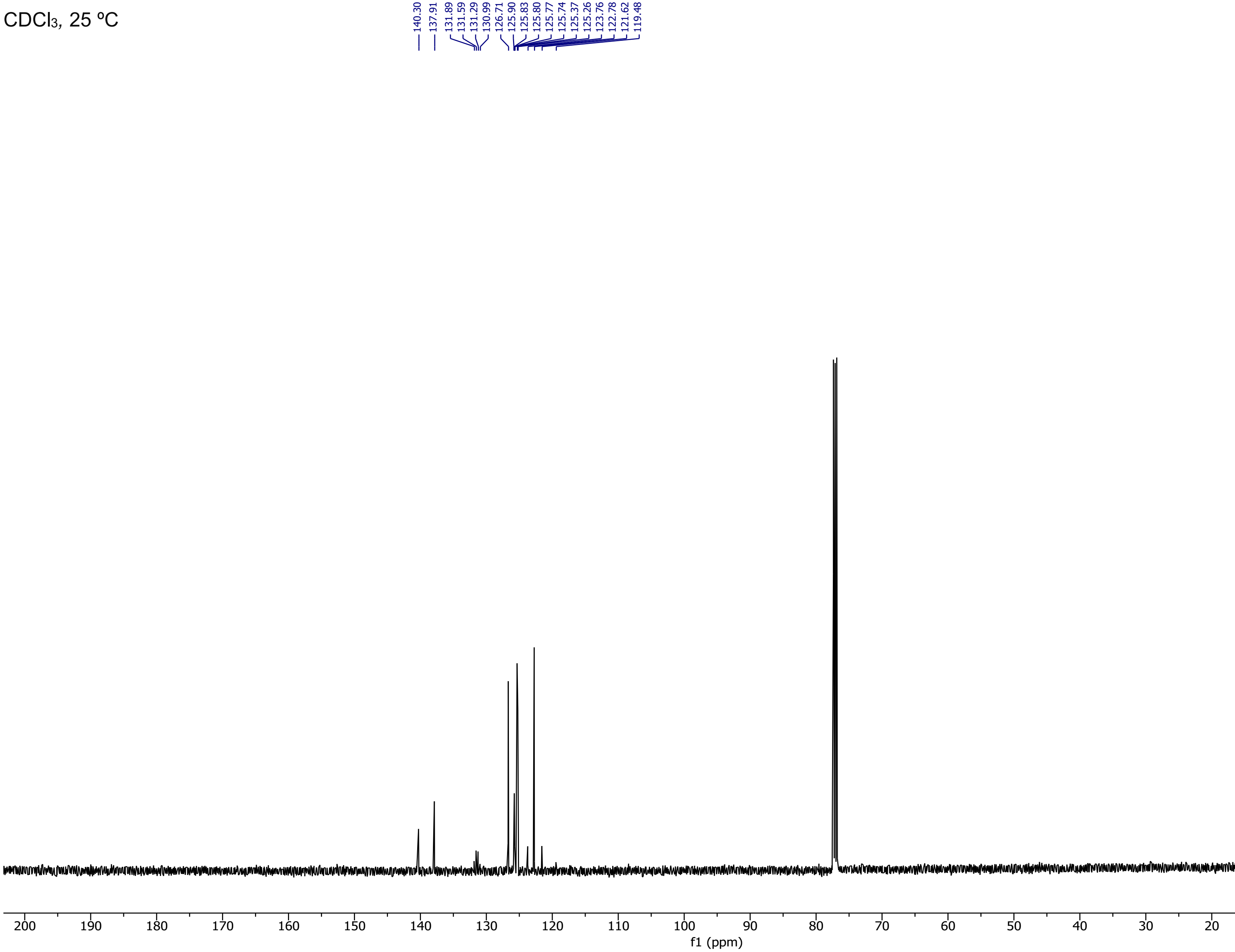

^{1}H NMR of caffeine derivative 7

CDCl$_3$, 25 ºC

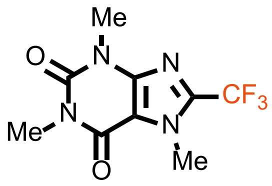

7

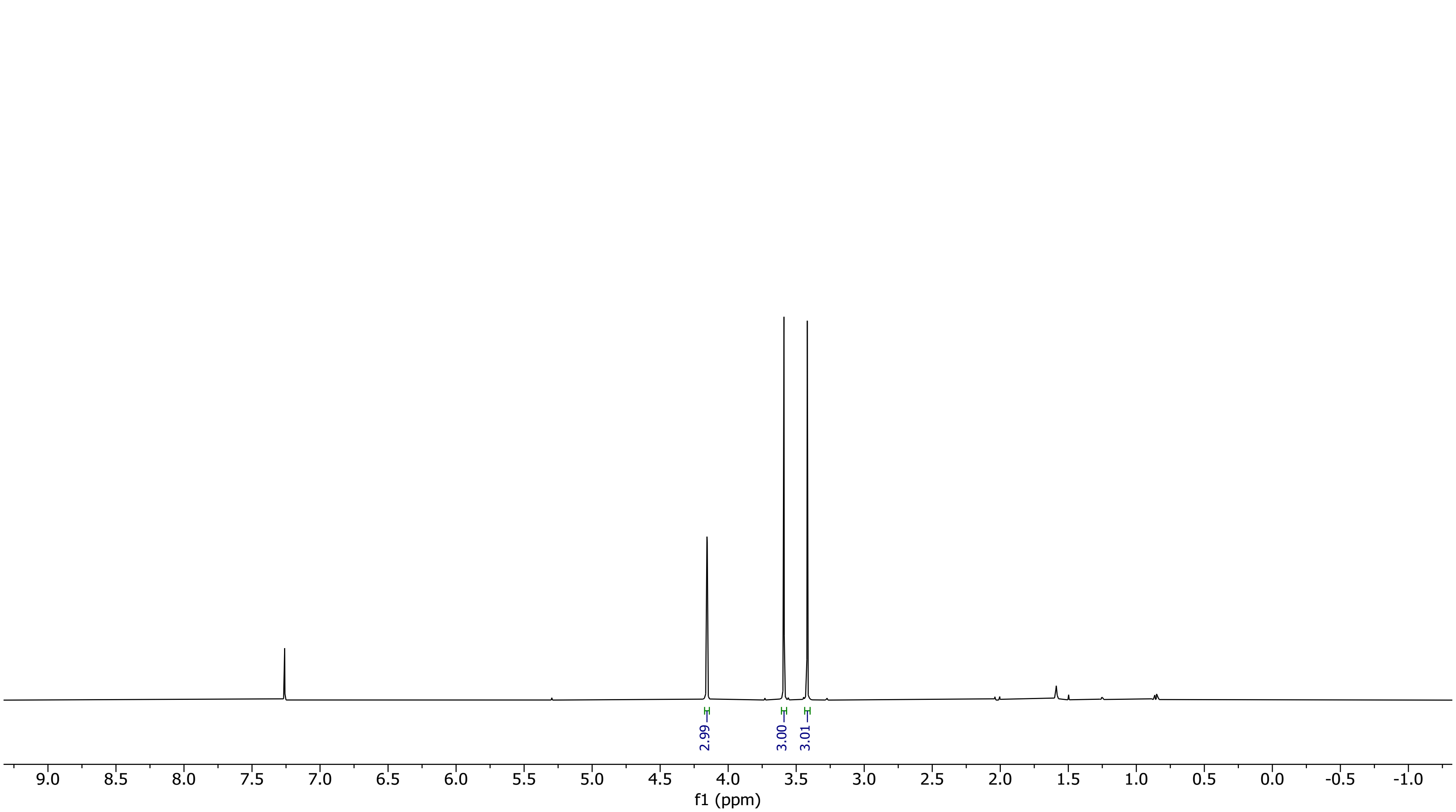

^{19}F NMR of caffeine derivative 7

CDCl$_3$, 25 ºC

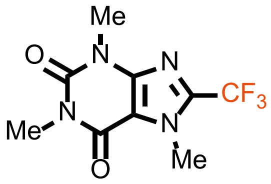

7

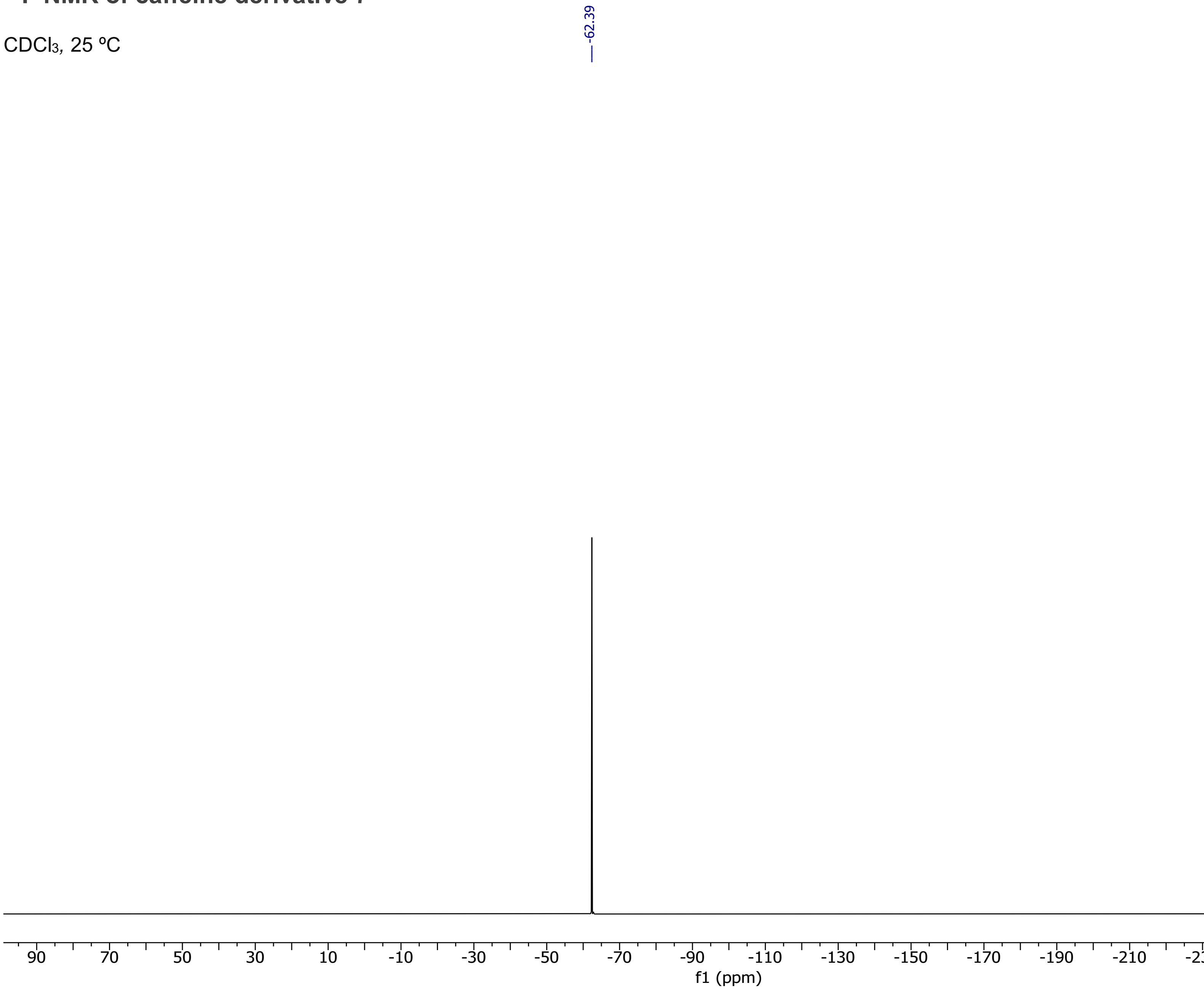

^{13}C NMR of caffeine derivative 7

CDCl$_3$, 25 ºC

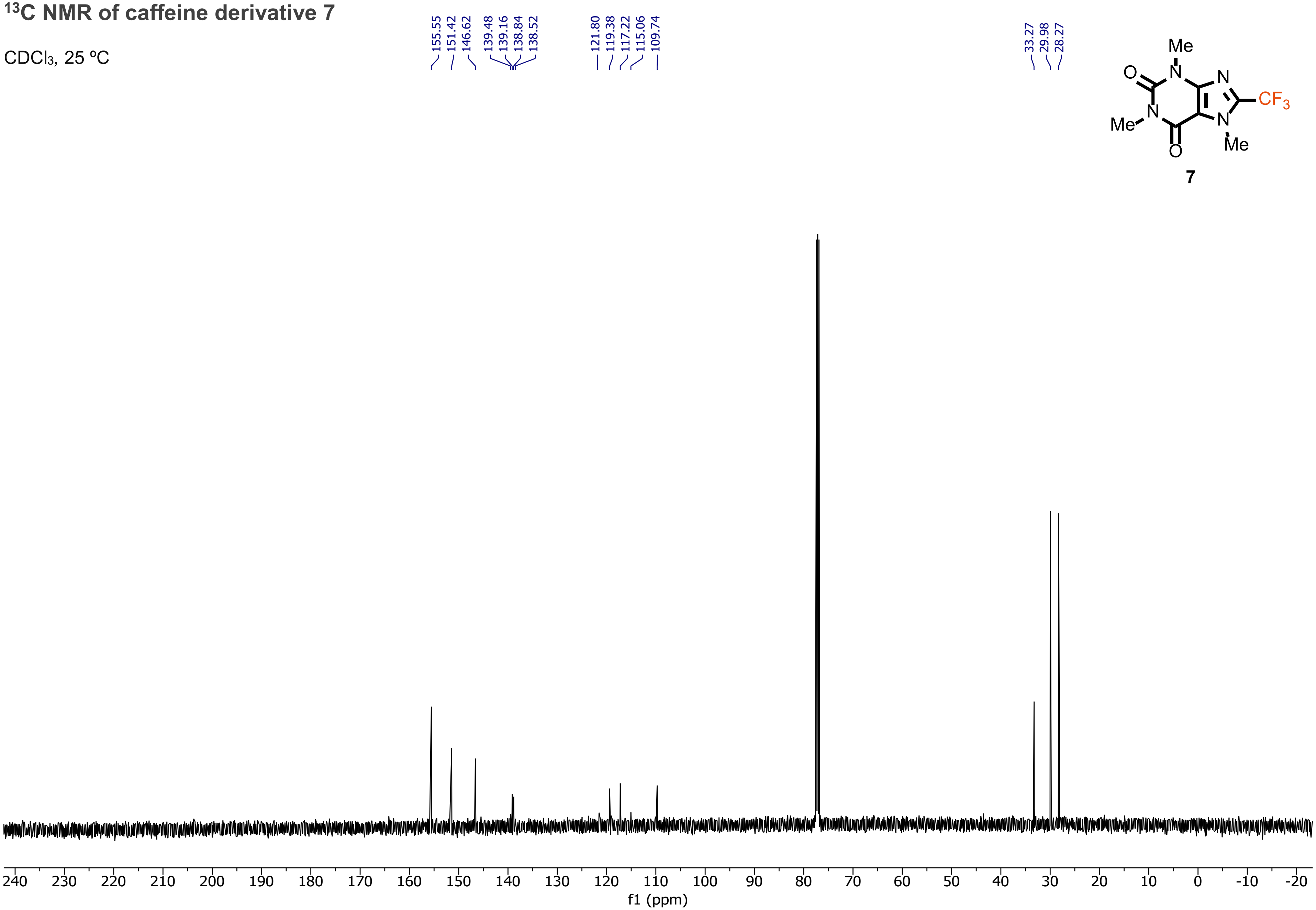

^{1}H NMR of mercaptopurine derivative 8

CD_3CN, 25 ºC

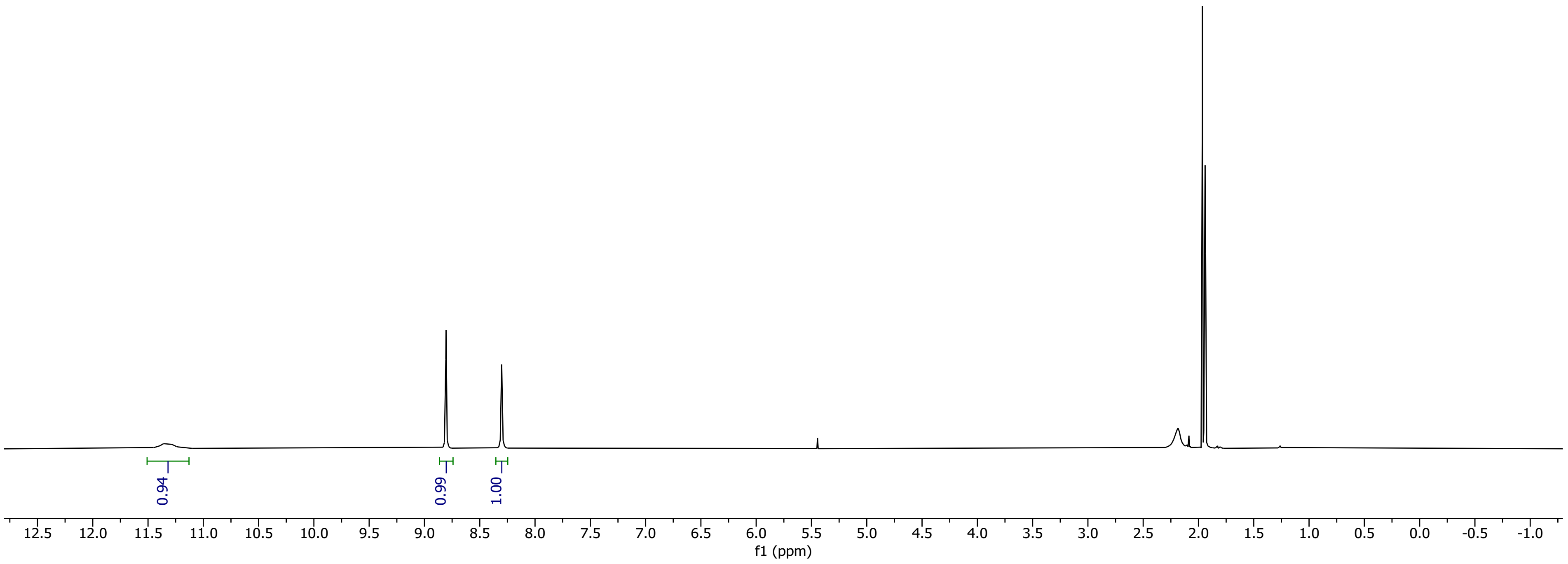

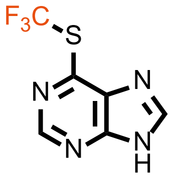

8

^{19}F NMR of mercaptopurine derivative 8

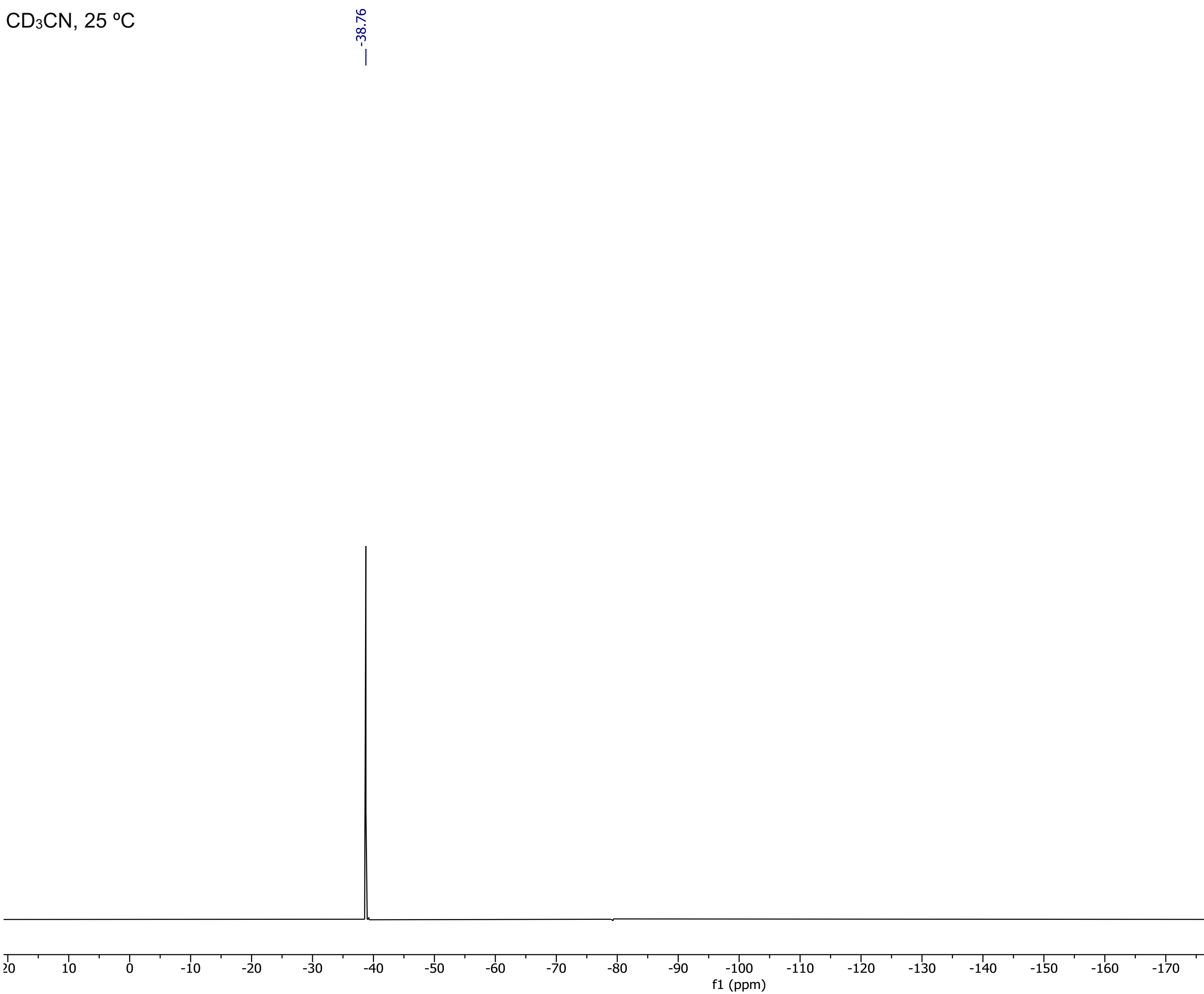

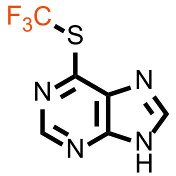

8

^{13}C NMR of mercaptopurine derivative 8

CD3CN, 25 ºC

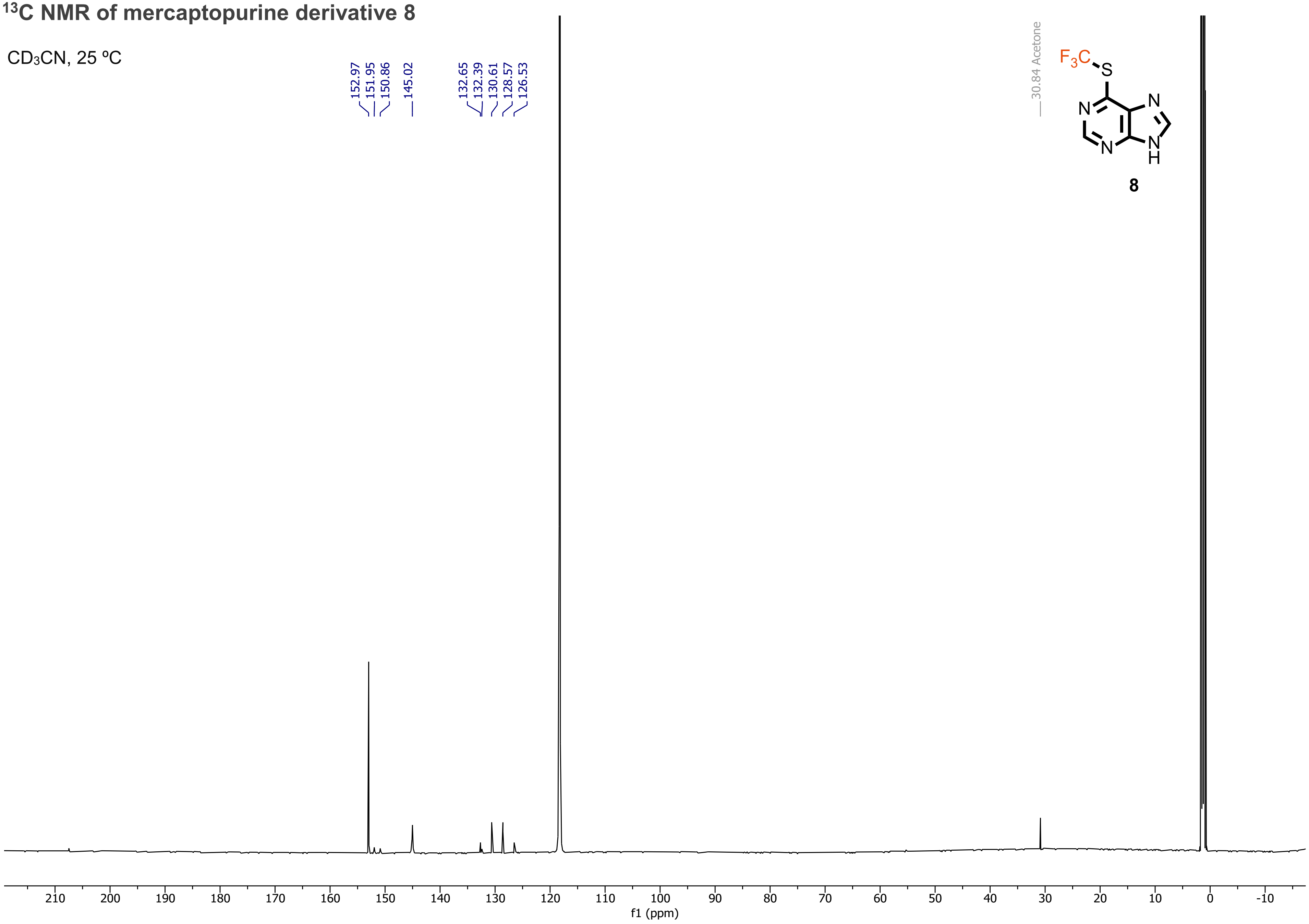

^{1}H NMR of visnagin derivative 9

CDCl$_3$, 25 ºC

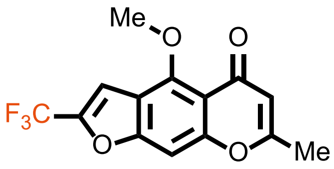

9

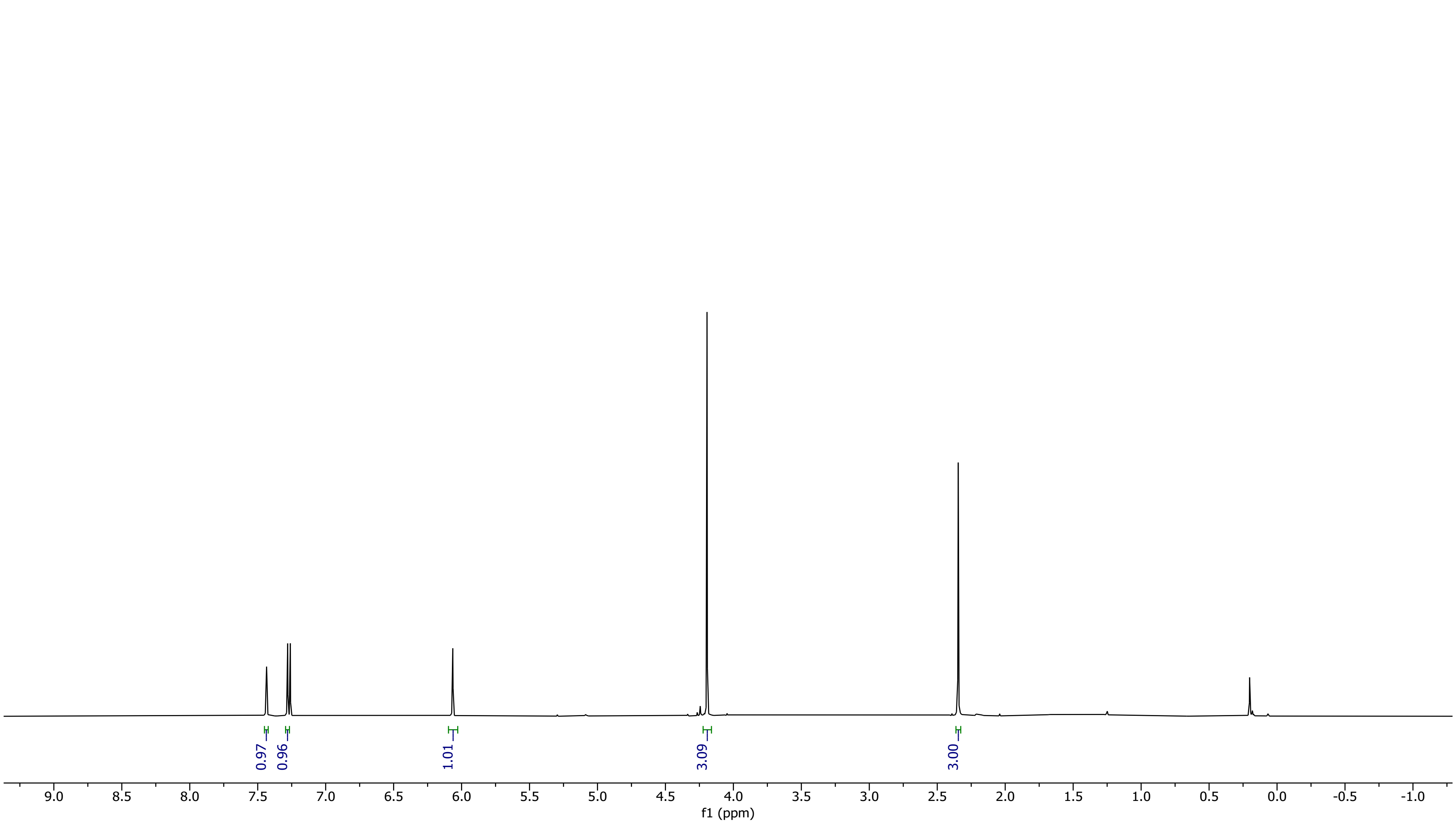

^{19}F NMR of visnagin derivative 9

CDCl$_3$, 25 ºC

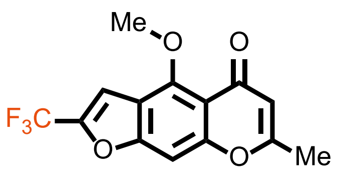

9

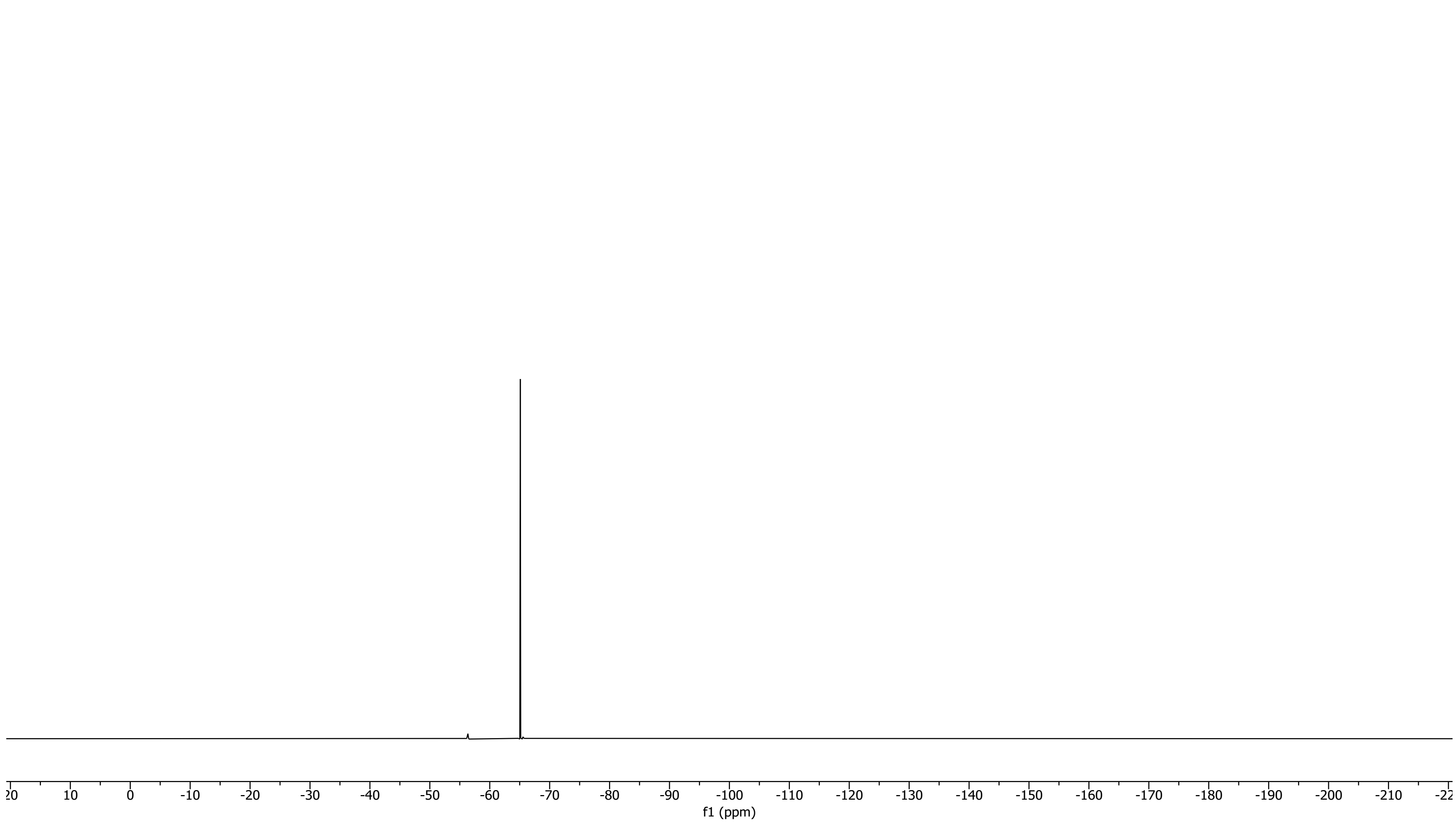

^{13}C NMR of visnagin derivative 9

CDCl$_3$, 25 ºC

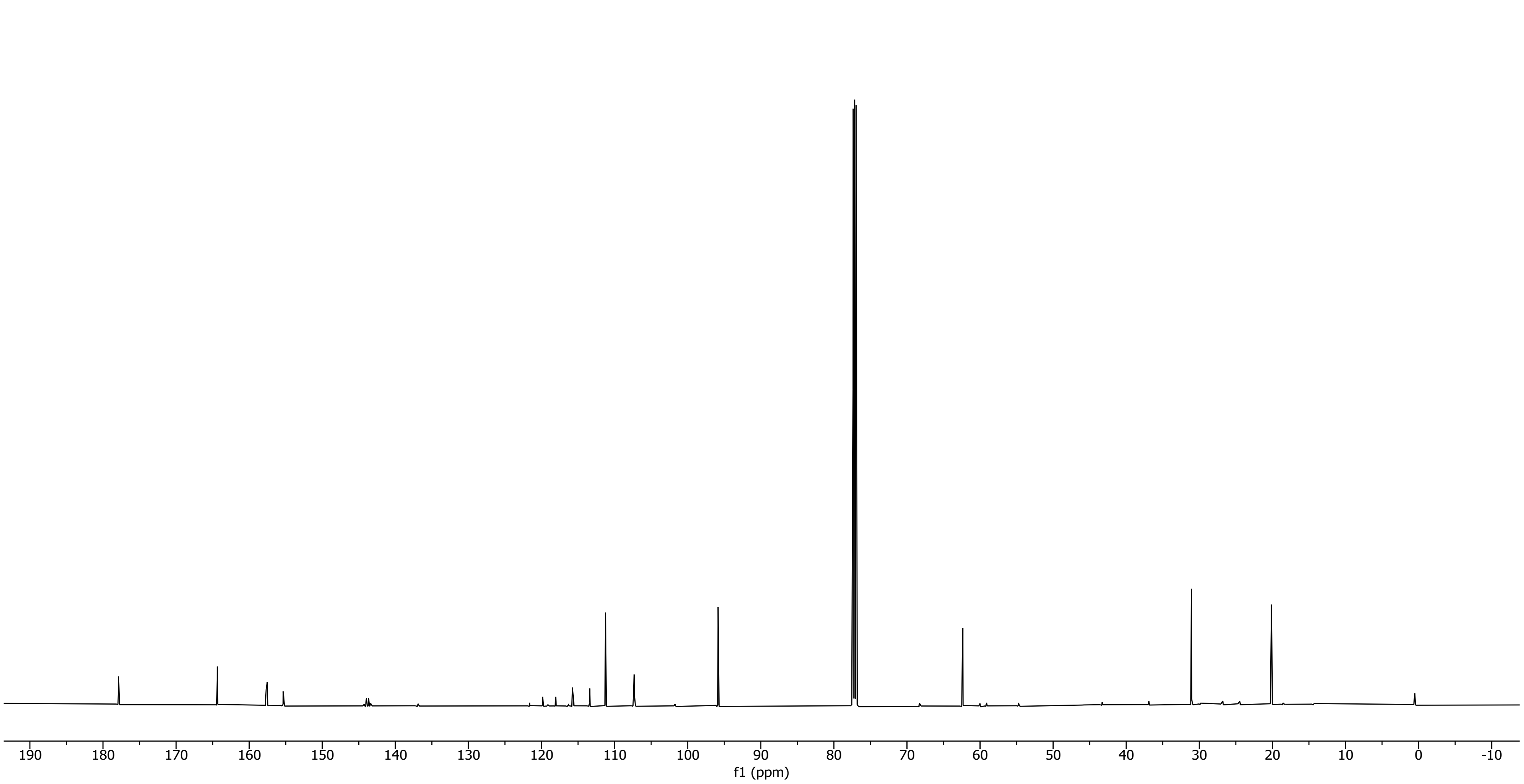

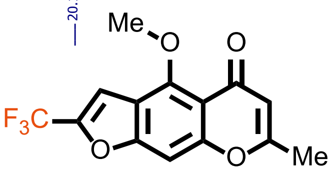

9

^{1}H NMR of 1,1,1,3,3,3-Hexafluoro-2-phenyl-2-propanol (11)

CDCl3, 25 ºC

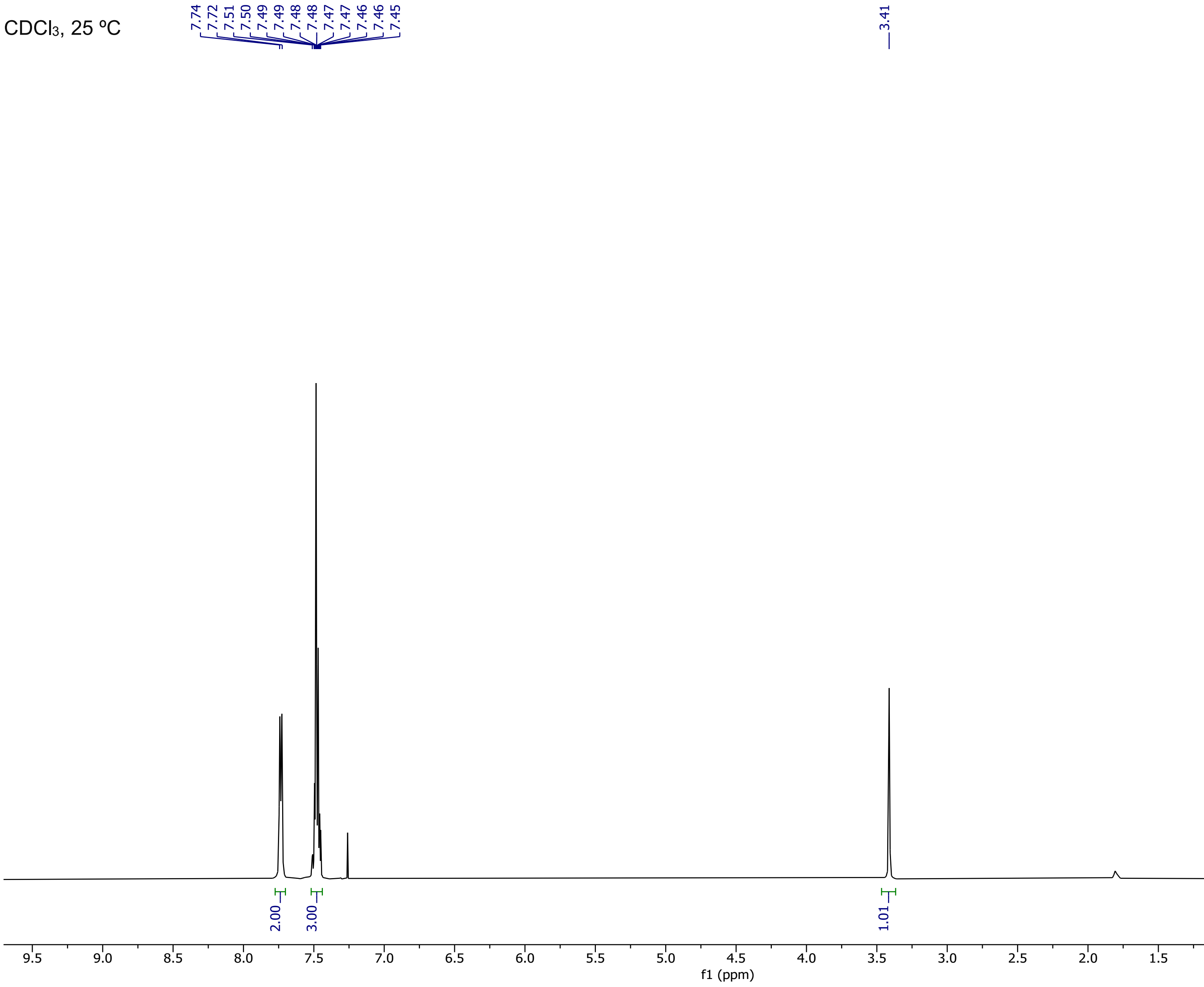

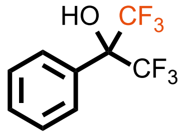

11

^{19}F NMR of 1,1,1,3,3,3-Hexafluoro-2-phenyl-2-propanol (11)

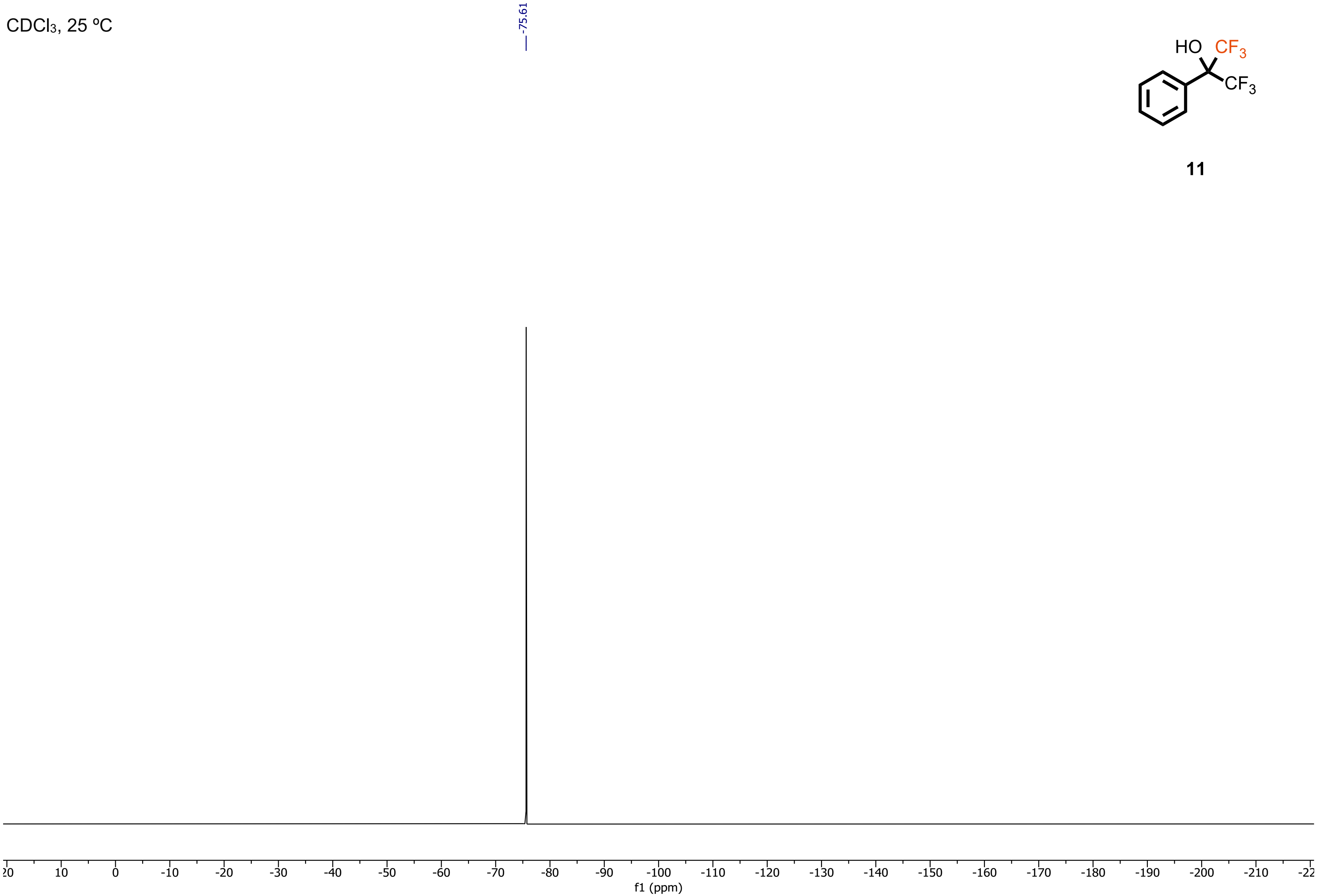

11

^{13}C NMR of 1,1,1,3,3,3-Hexafluoro-2-phenyl-2-propanol (11)

$CDCl_3$, 25 ºC

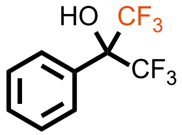

11

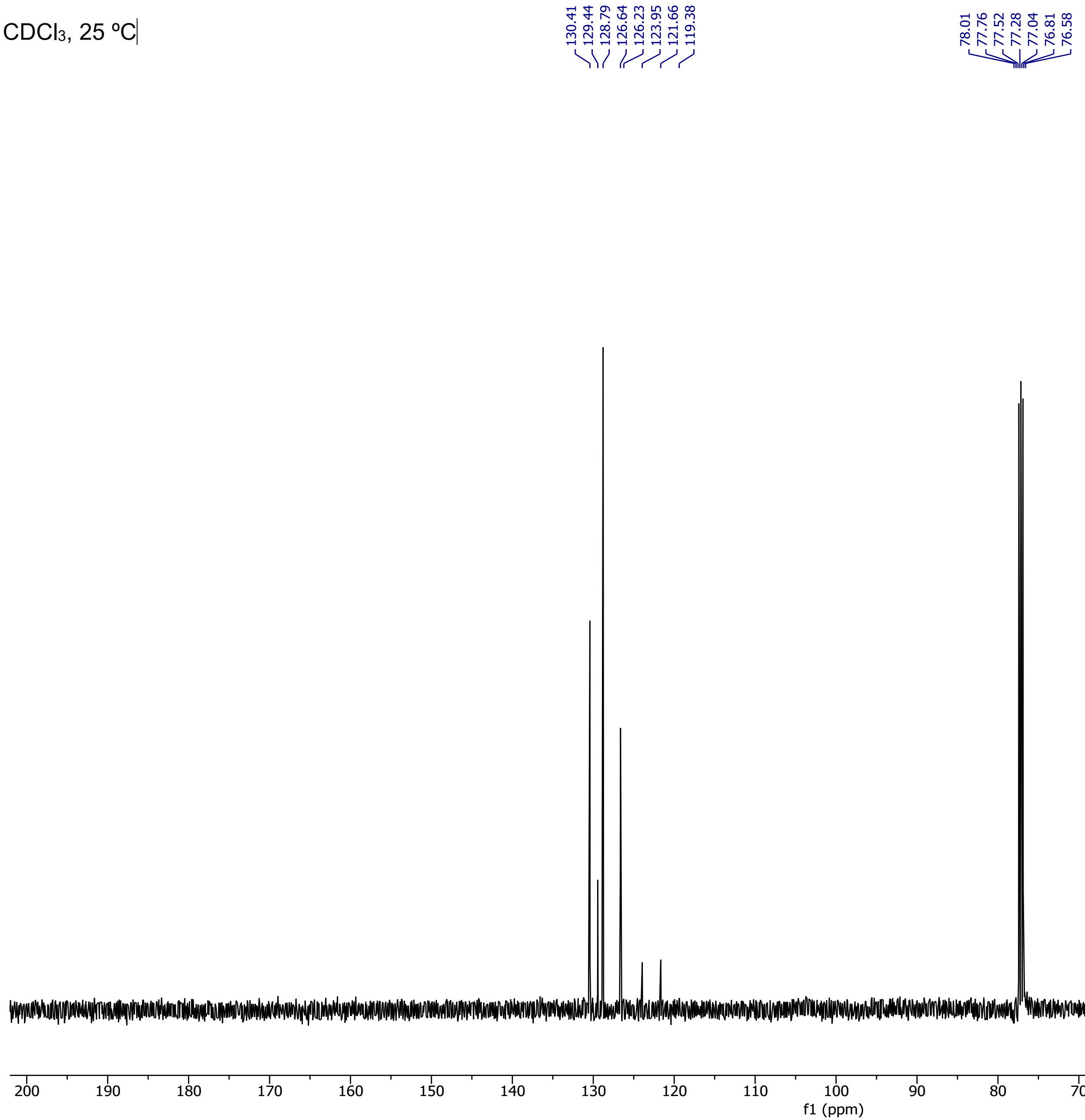

^{1}H NMR of methyl 4-(2,2,2-trifluoro-1-hydroxyethyl)benzoate (13)

$CDCl_3$, 25 ºC

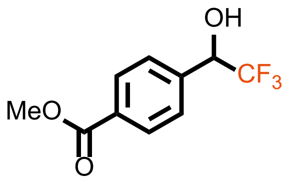

13

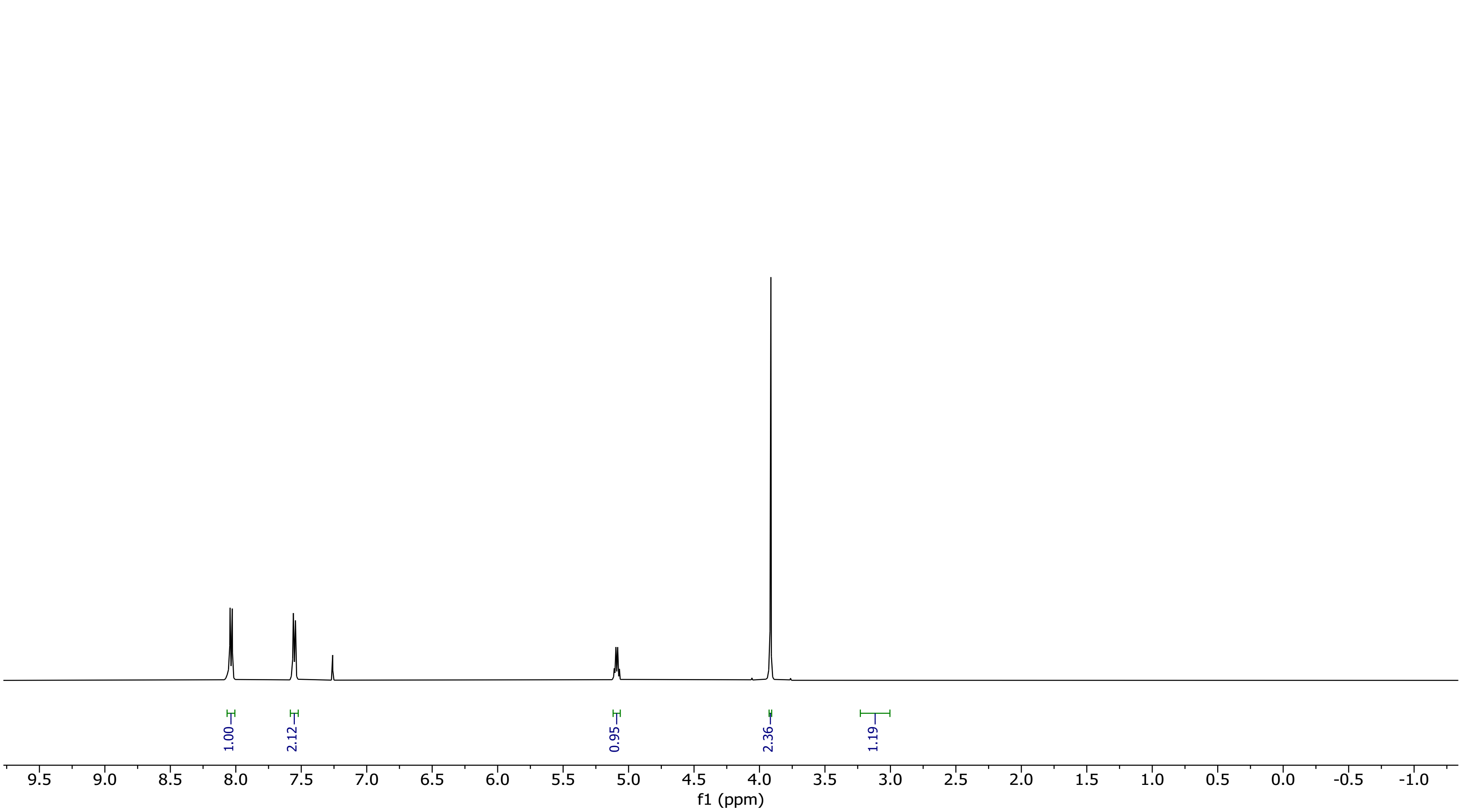

^{19}F NMR of methyl 4-(2,2,2-trifluoro-1-hydroxyethyl)benzoate (13)

$CDCl_3$, 25 ºC

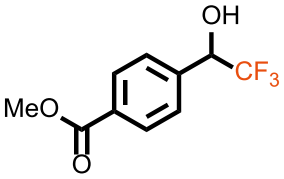

13

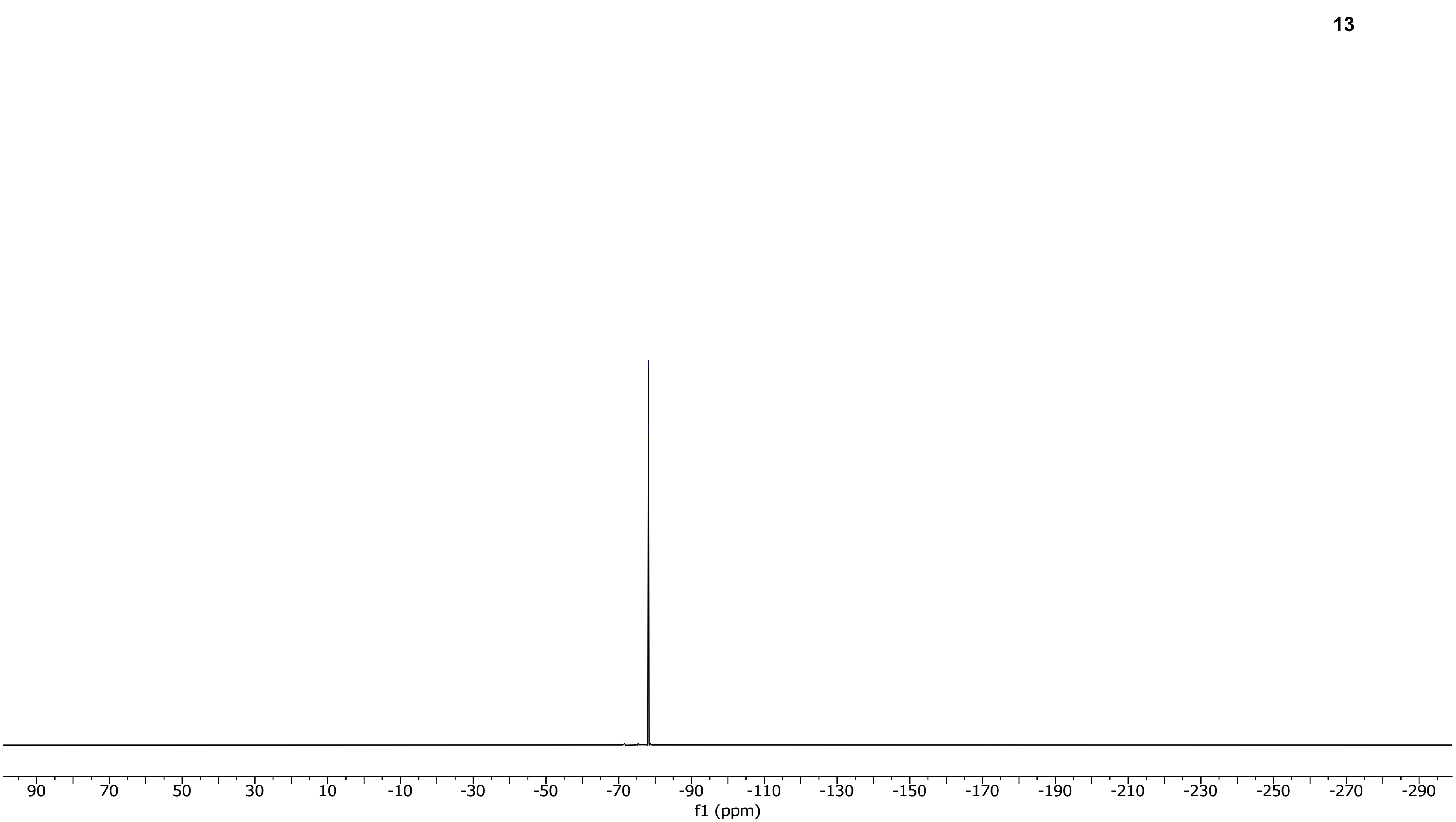

^{13}C NMR of methyl 4-(2,2,2-trifluoro-1-hydroxyethyl)benzoate (13)

CDCl3, 25 ºC

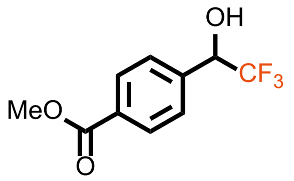

13

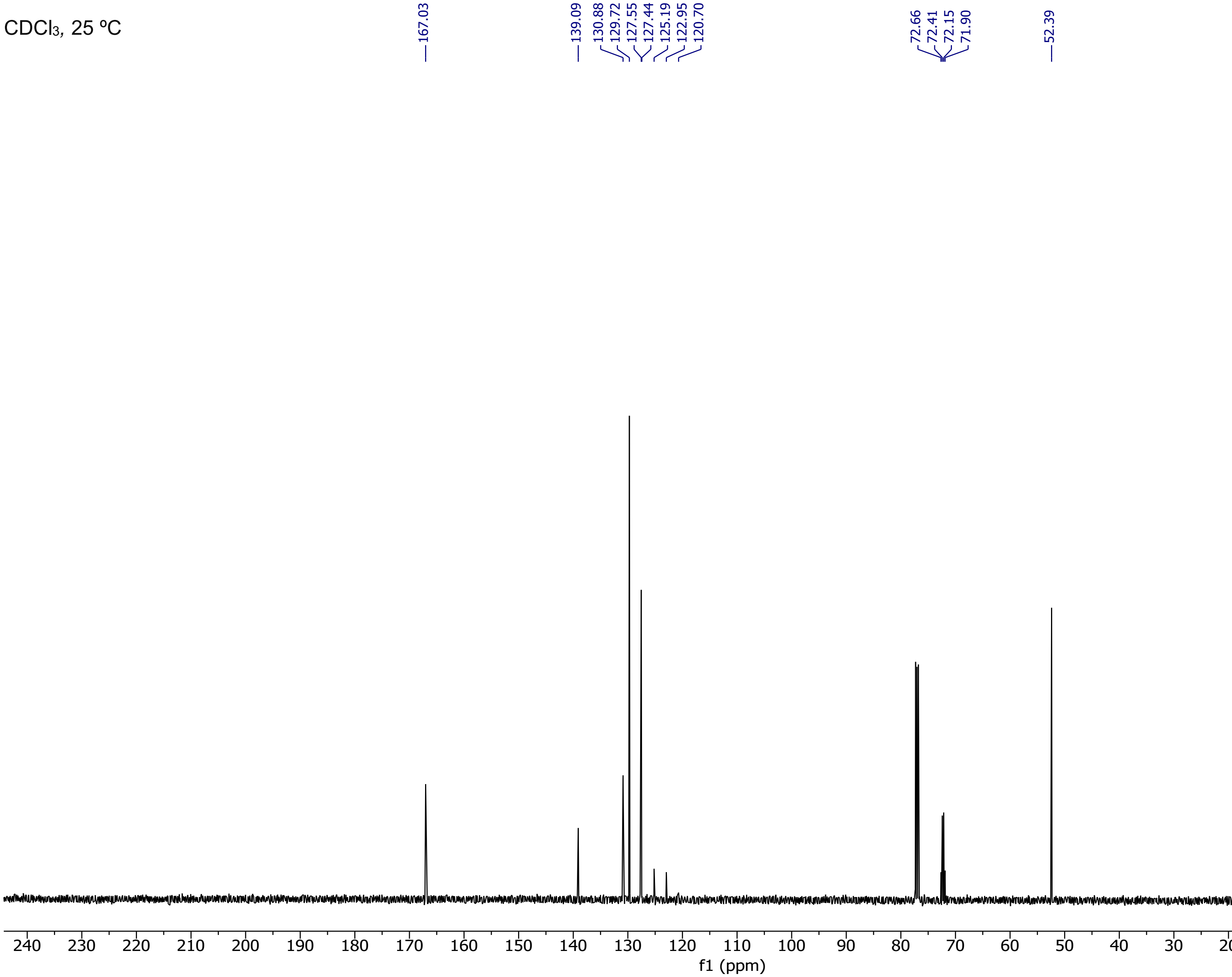

^{1}H NMR of 2-methyl-1-phenyl-2-(trifluoromethyl)butane-1,3-dione (14)

$CDCl_3$, 25 ºC

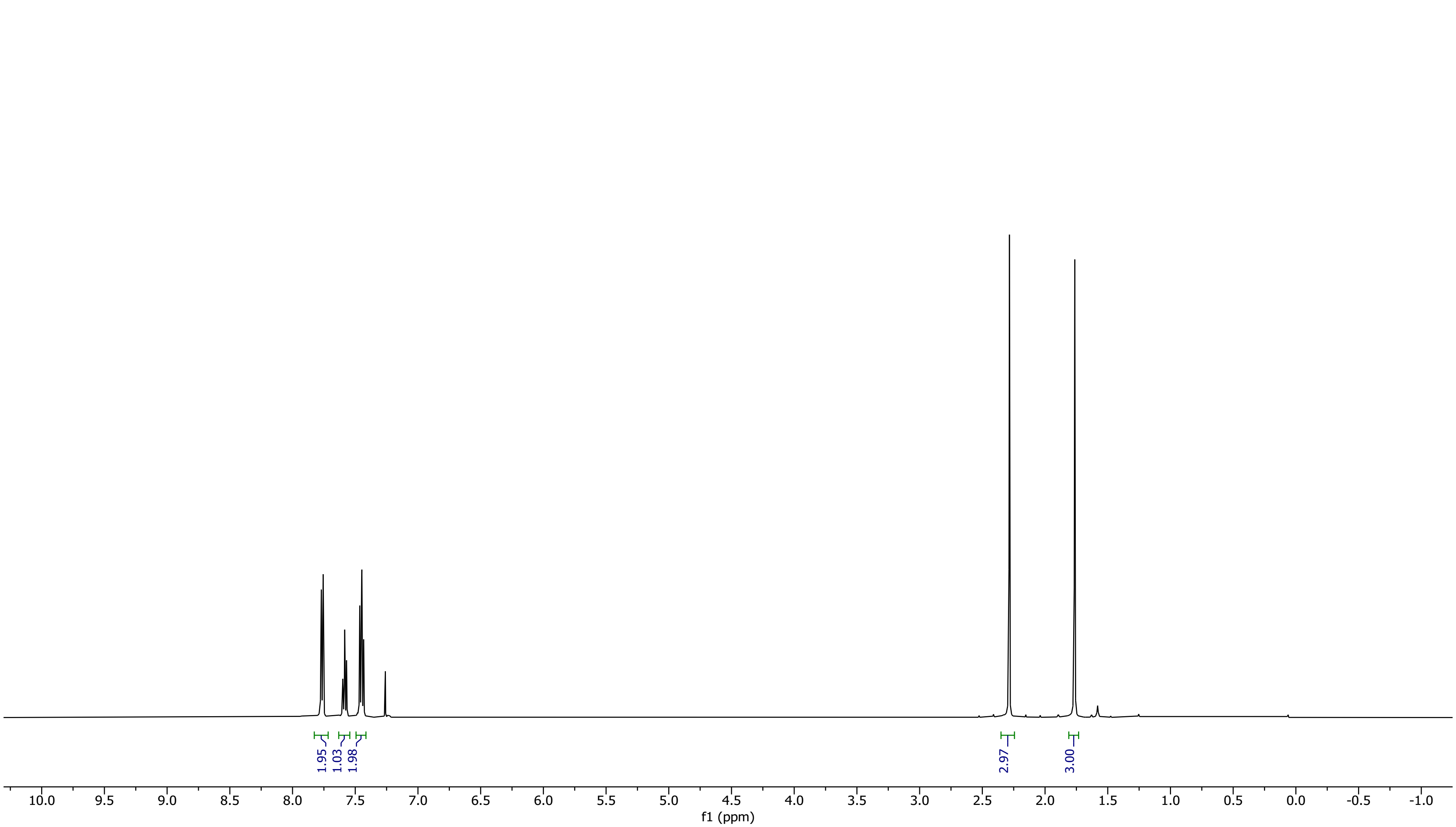

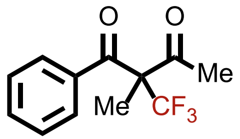

14

^{19}F NMR of 2-methyl-1-phenyl-2-(trifluoromethyl)butane-1,3-dione (14)

CDCl$_3$, 25 ºC

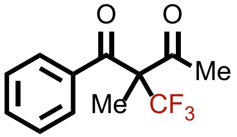

14

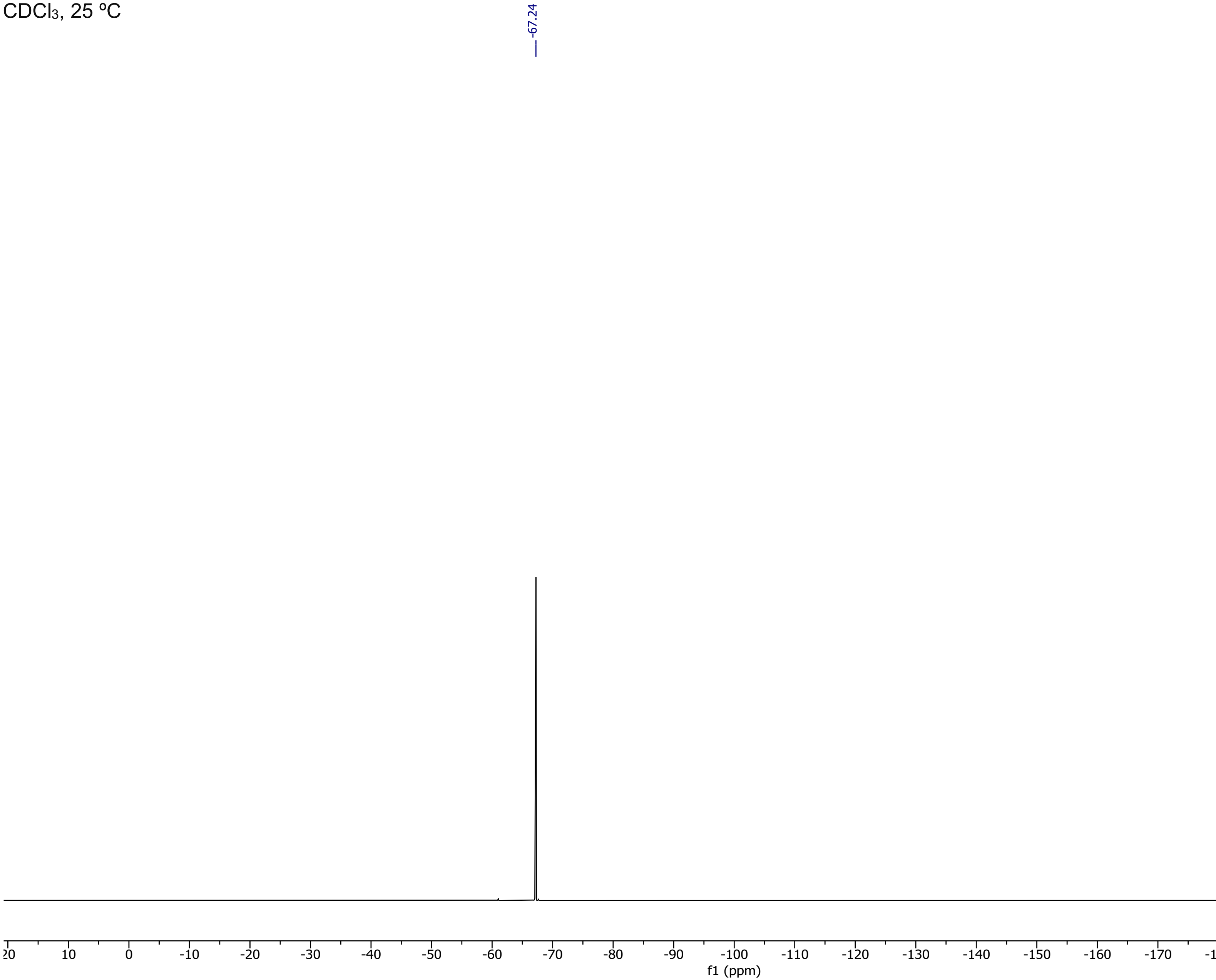

^{13}C NMR of 2-methyl-1-phenyl-2-(trifluoromethyl)butane-1,3-dione (14)

CDCl3, 25 ºC

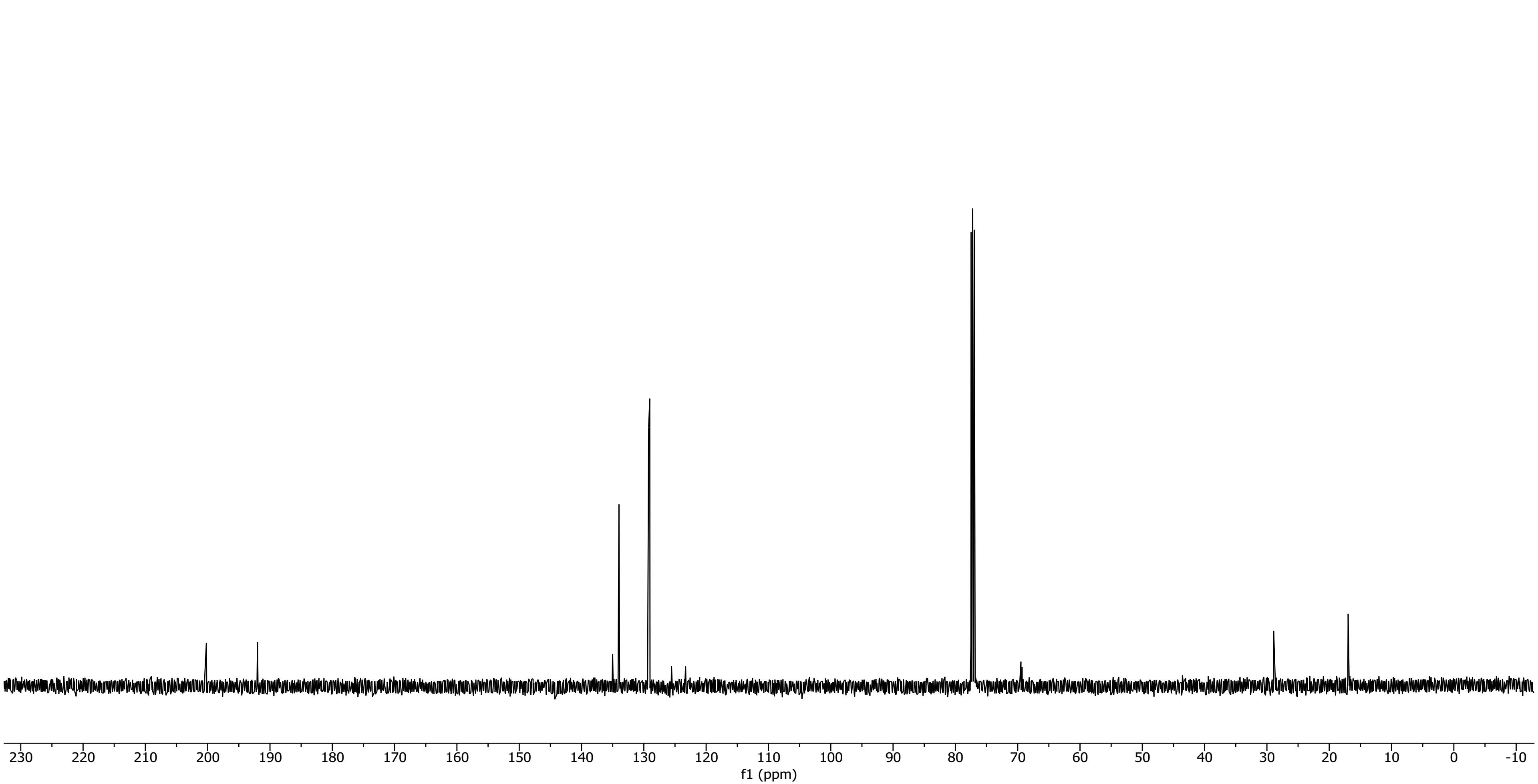

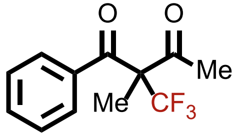

14

^{1}H NMR of 8-((trifluoromethyl)thio)quinolone (16)

CDCl₃, 25 ºC

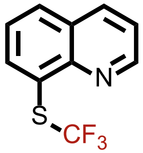

16

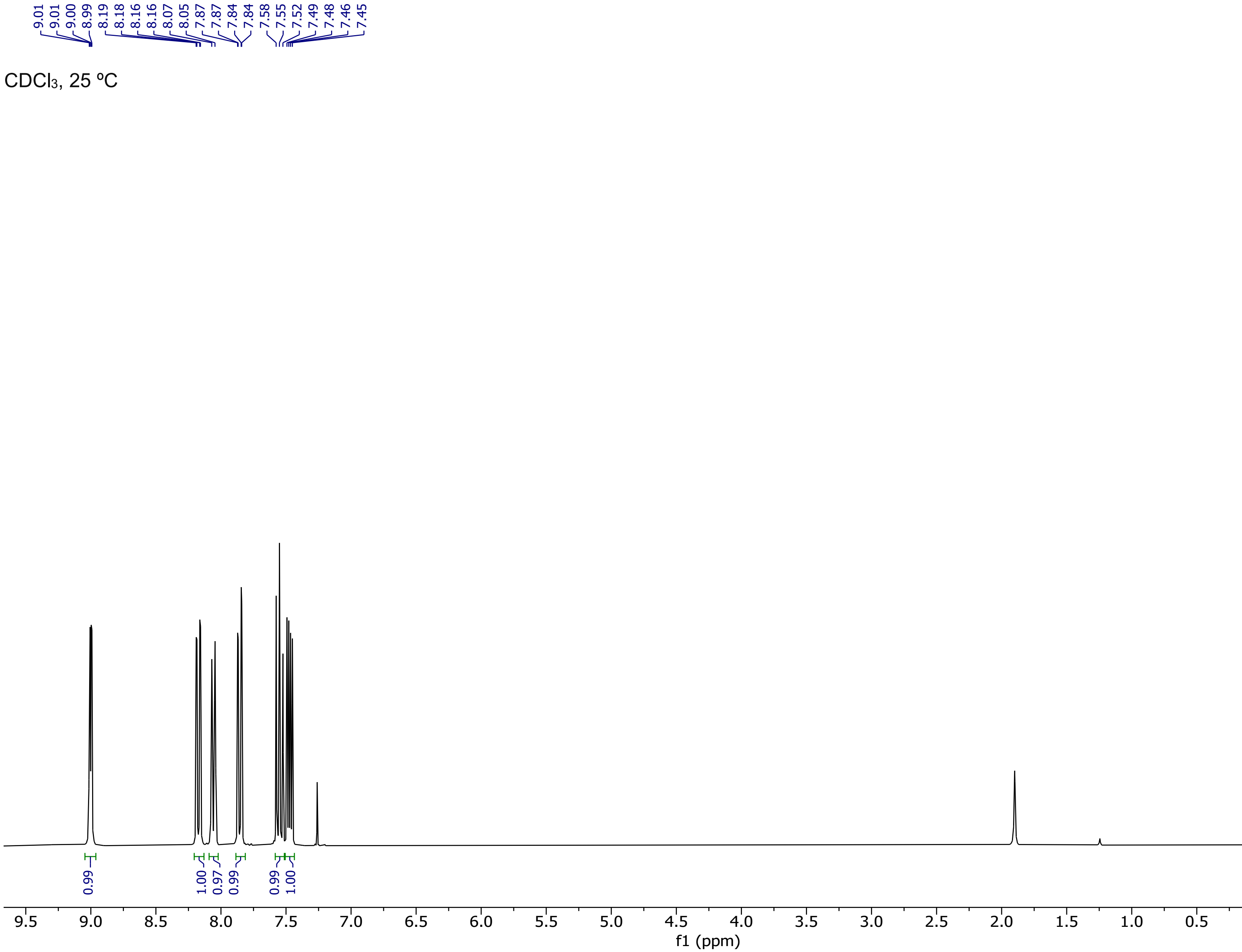

^{19}F NMR of 8-((trifluoromethyl)thio)quinolone (16)

CDCl$_3$, 25 ºC

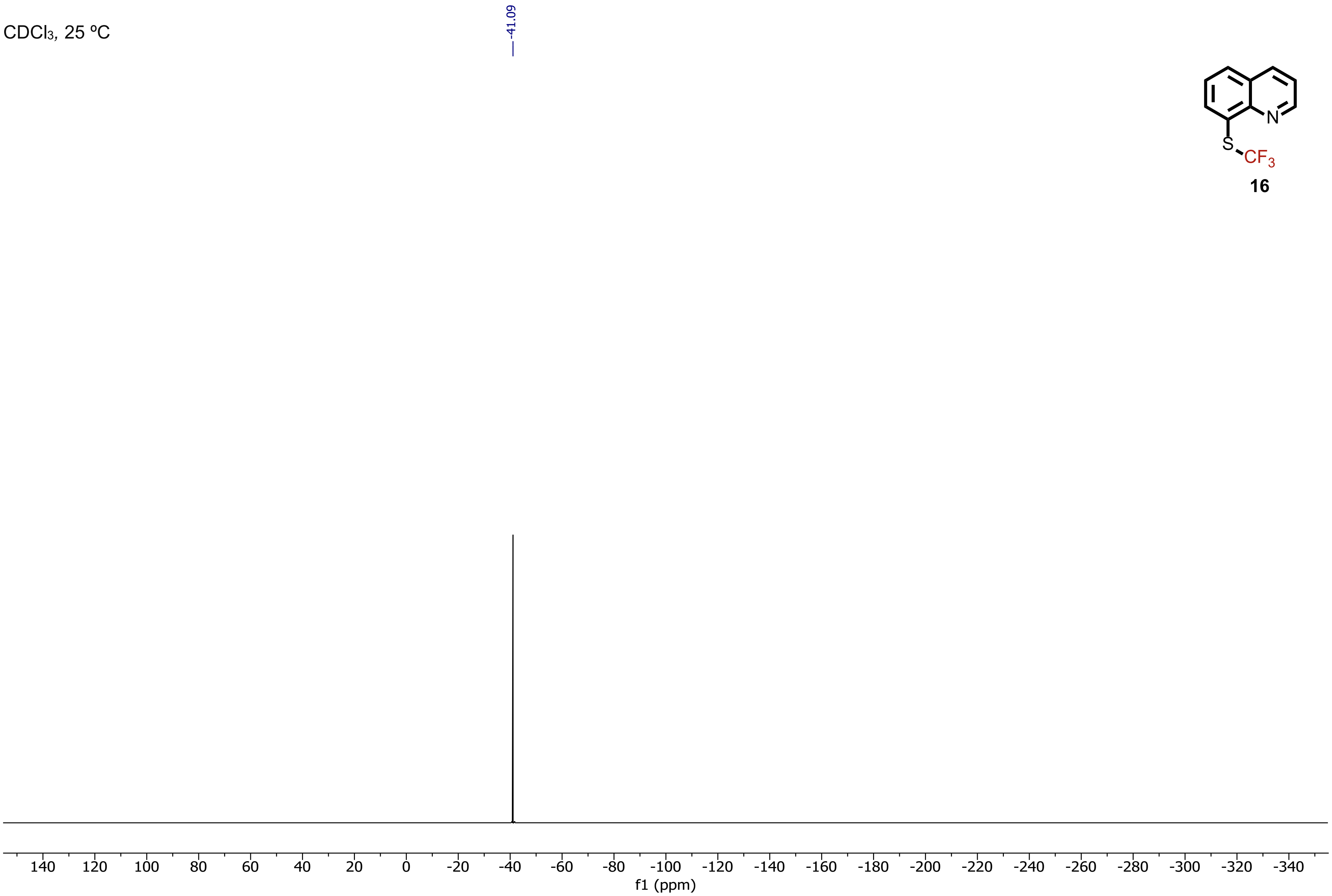

16

^{13}C NMR of 8-((trifluoromethyl)thio)quinolone (16)

CDCl3, 25 ºC

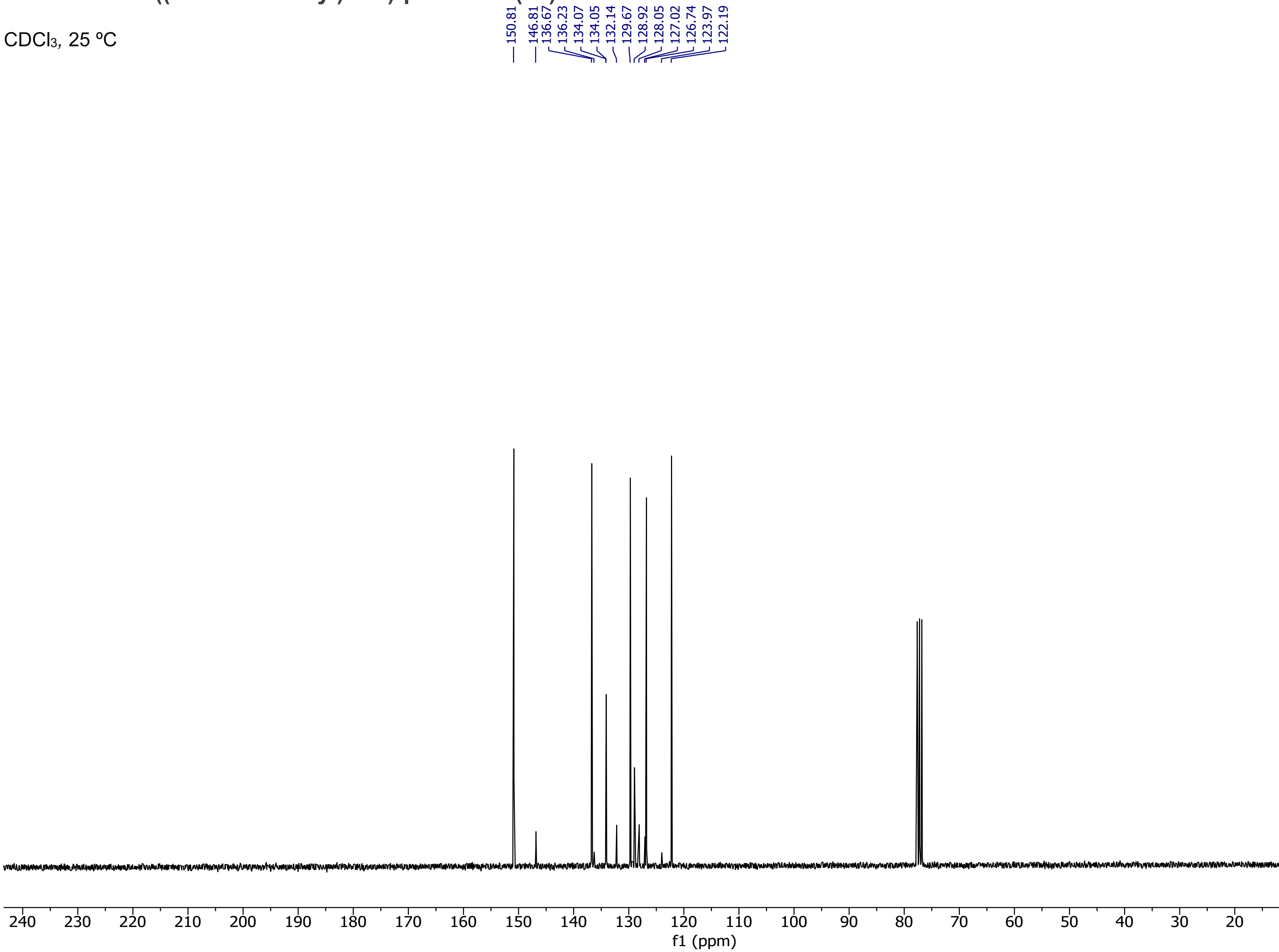

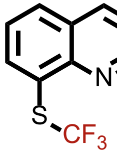

16

^{1}H NMR of 5-chloro-2-((trifluoromethyl)thio)benzo[*d*]thiazole (17)

$CDCl_3$, 25 ºC

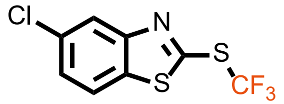

17

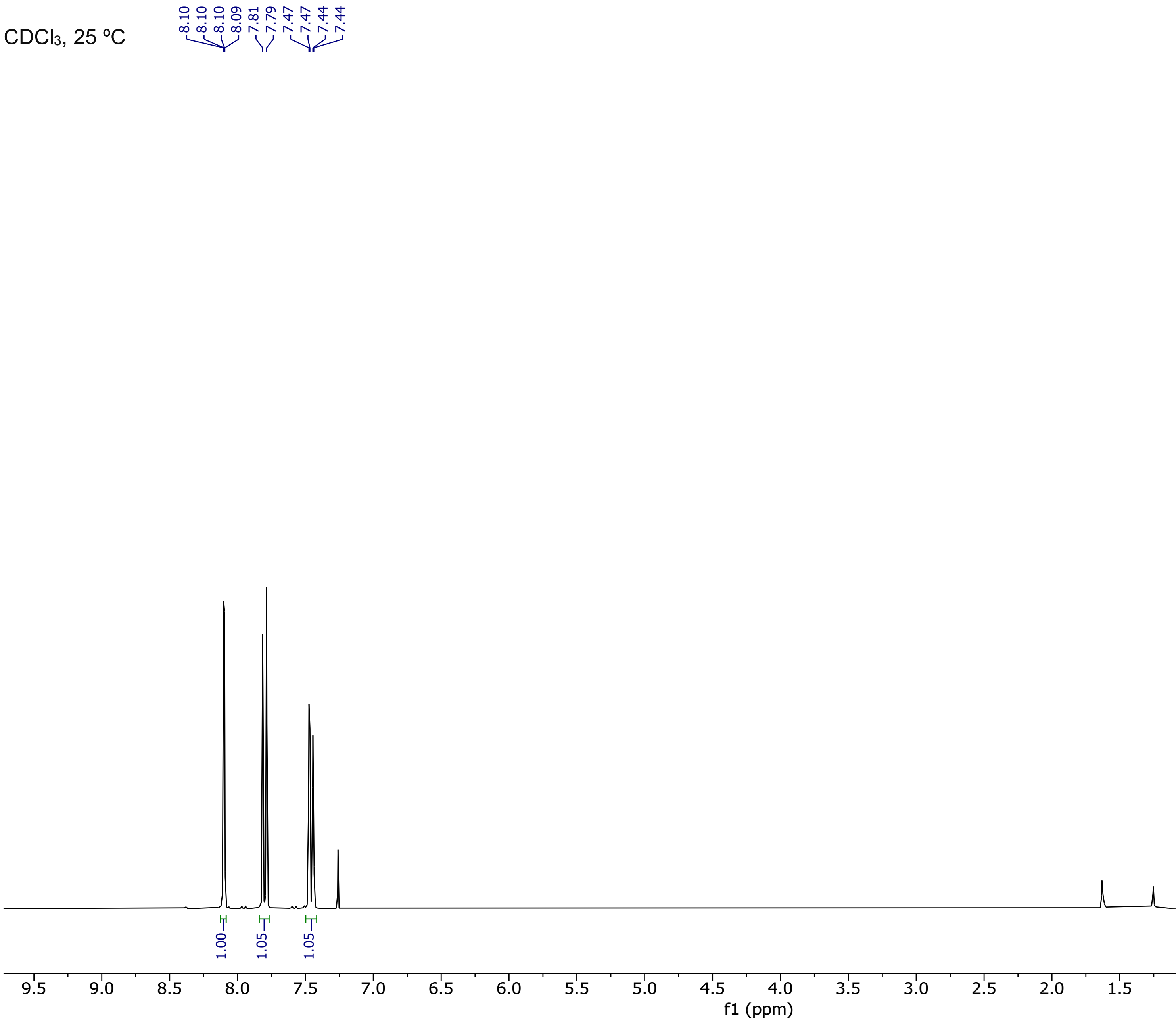

^{19}F NMR of 5-chloro-2-((trifluoromethyl)thio)benzo[*d*]thiazole (17)

CDCl3, 25 ºC

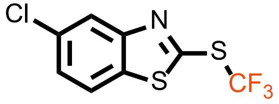

17

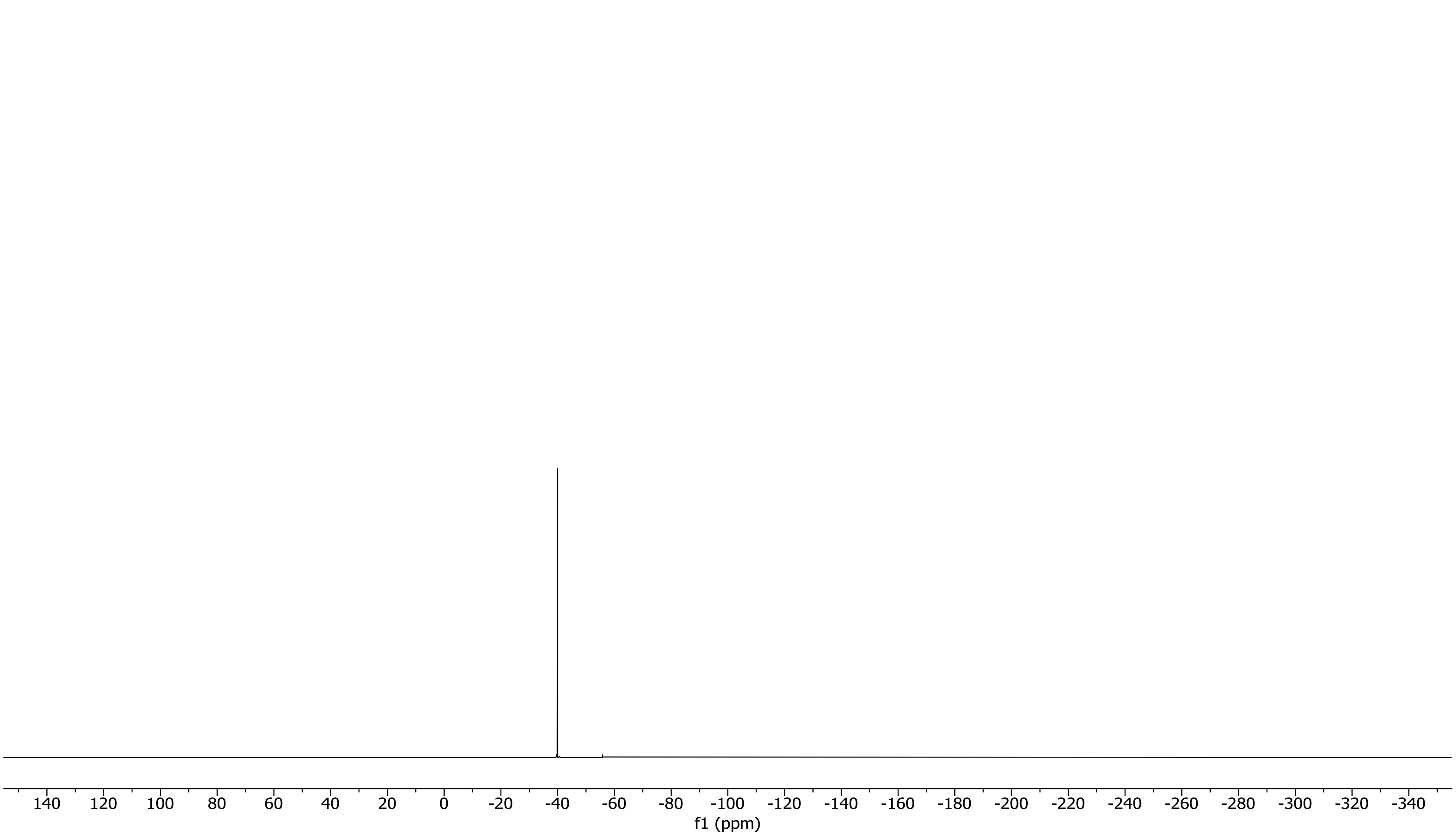

^{13}C NMR of 5-chloro-2-((trifluoromethyl)thio)benzo[*d*]thiazole (17)

CDCl$_3$, 25 ºC

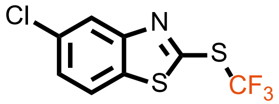

17

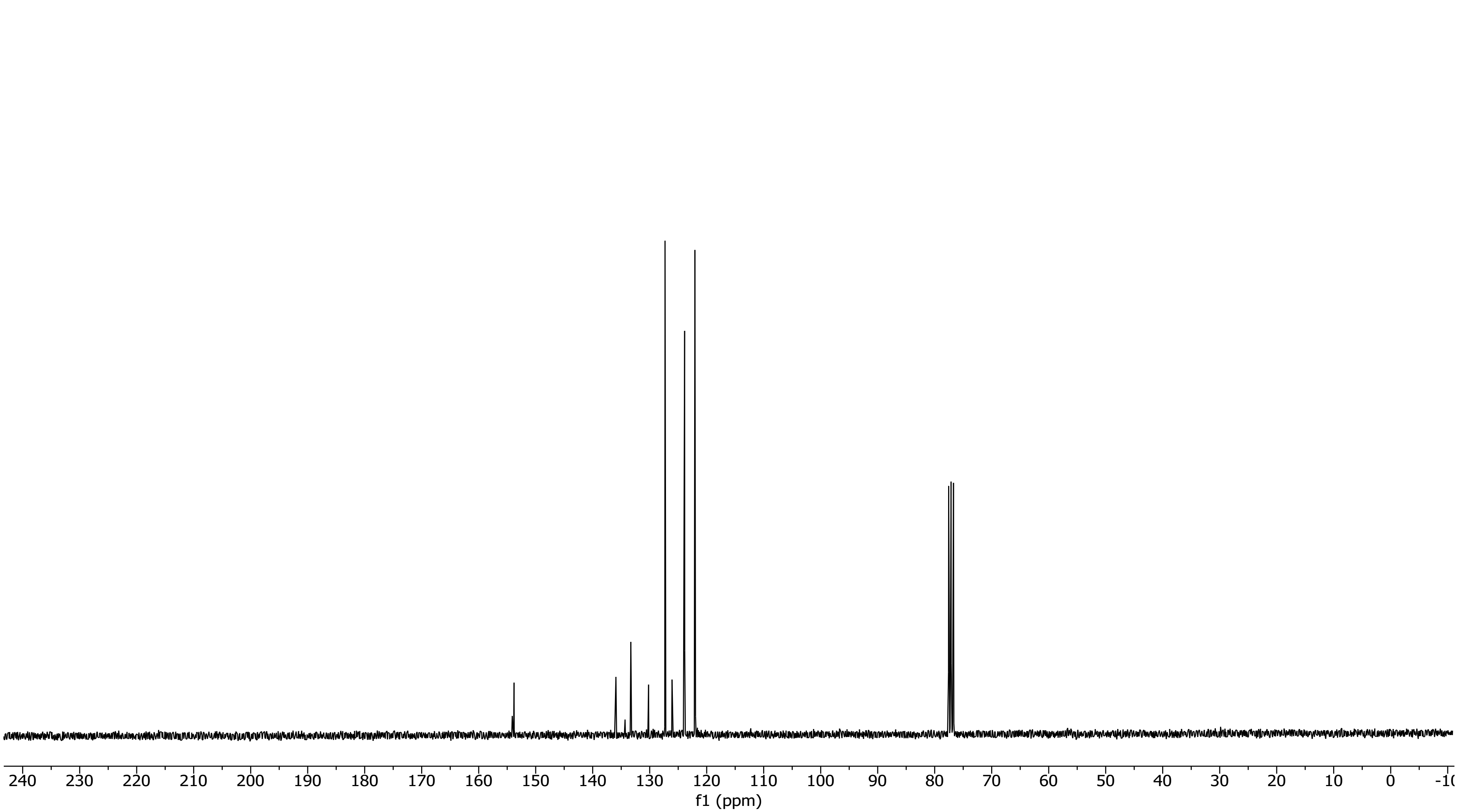

^{1}H NMR of methyl *N*-acetyl-*S*-(trifluoromethyl)-*L*-cysteinate (18)

CDCl₃, 25 ºC

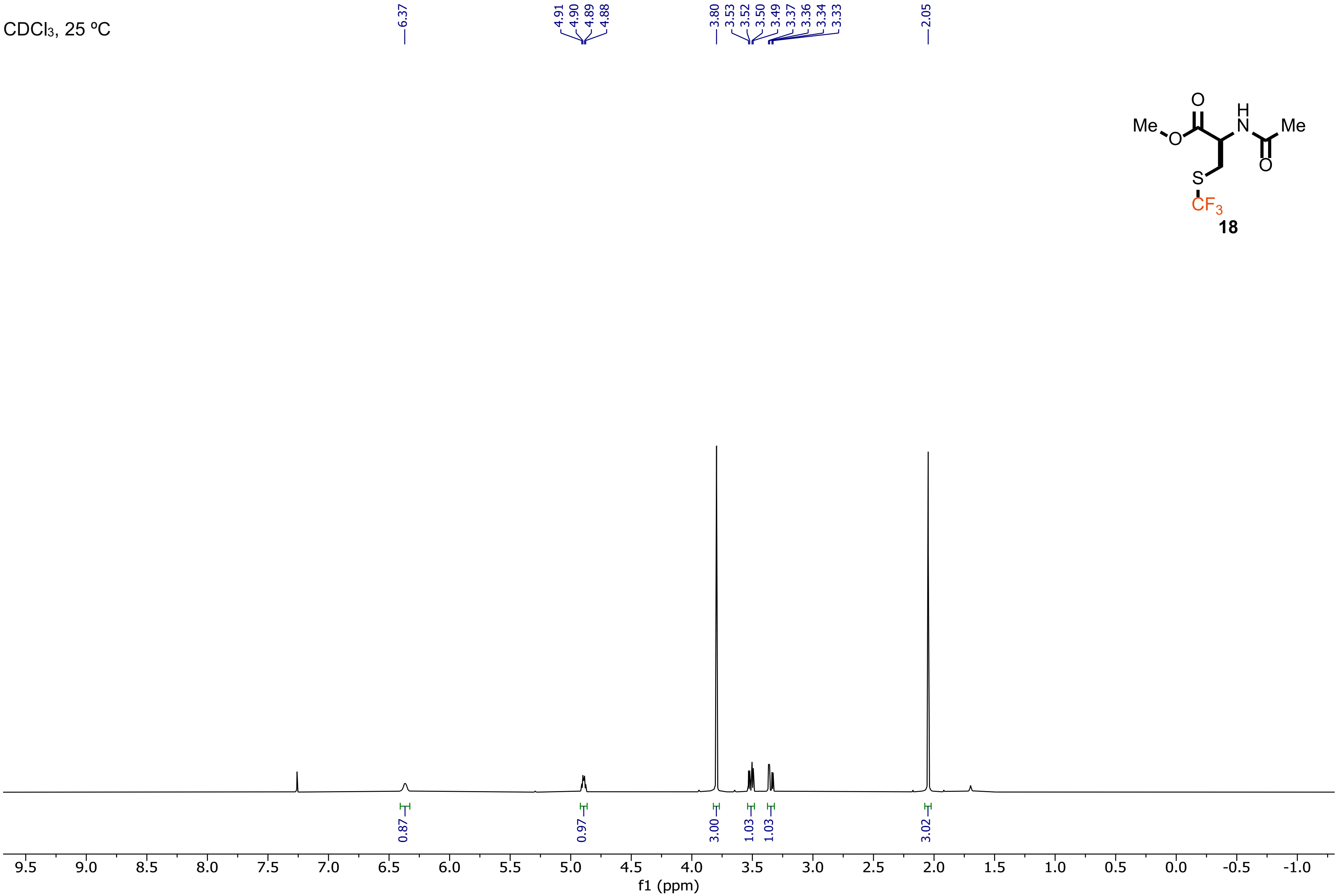

^{19}F NMR of methyl *N*-acetyl-*S*-(trifluoromethyl)-*L*-cysteinate (18)

$CDCl_3$, 25 ºC

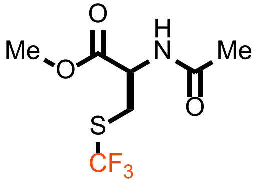

18

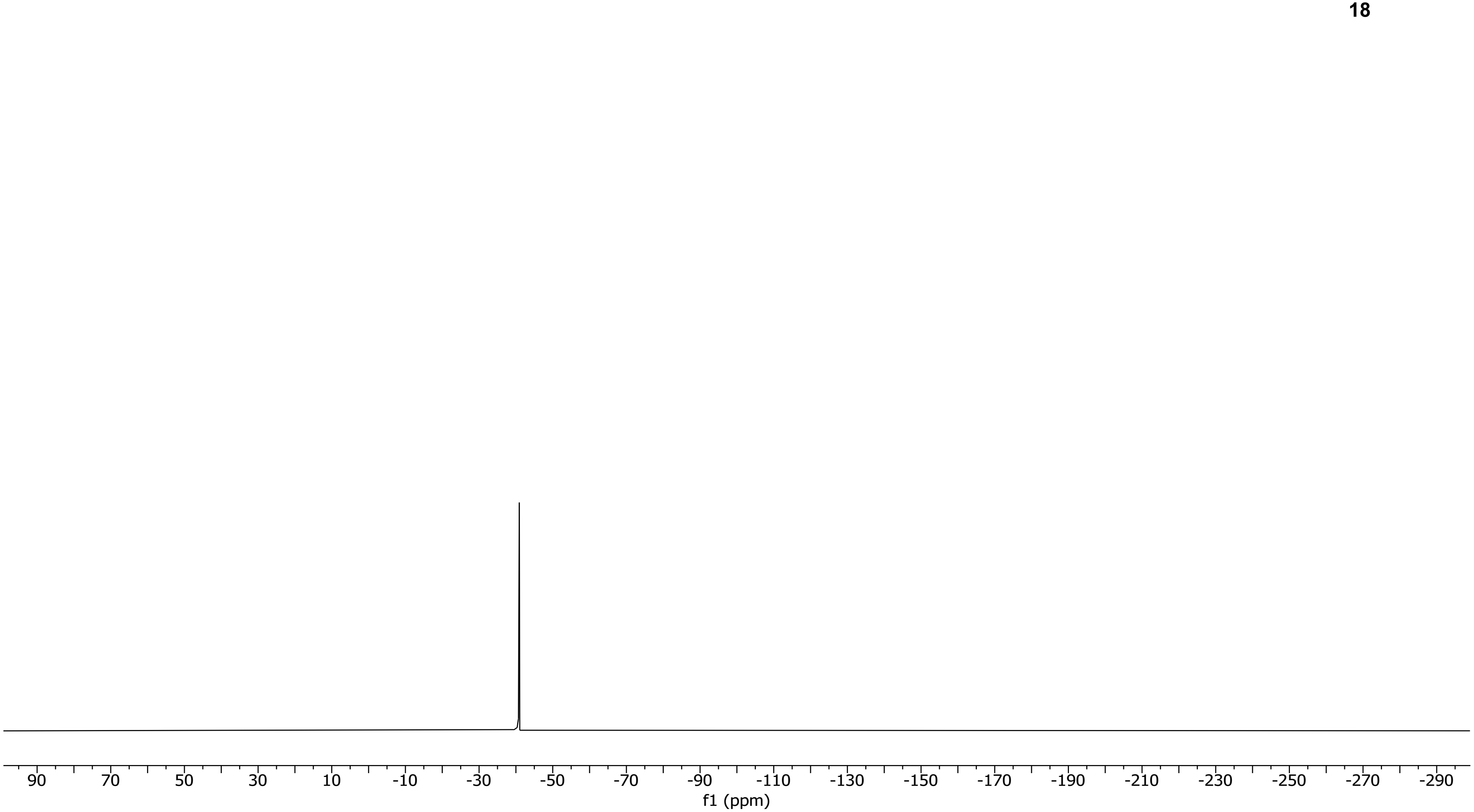

^{13}C NMR of methyl *N*-acetyl-*S*-(trifluoromethyl)-*L*-cysteinate (18)

CDCl$_3$, 25 ºC

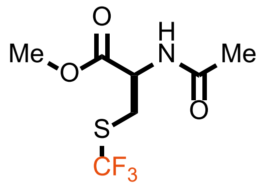

18

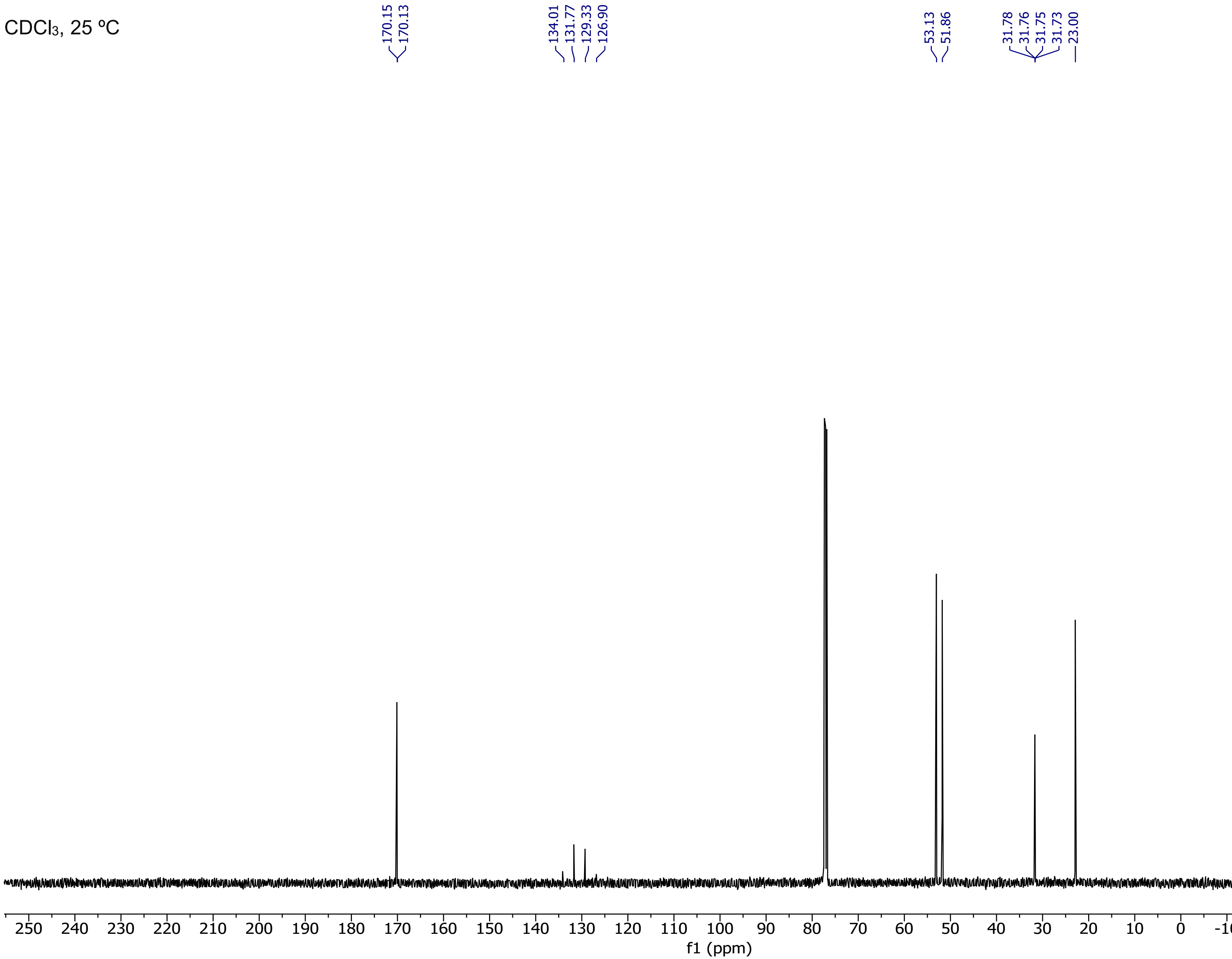

^{1}H NMR of racecadotril derivative 19

CDCl$_3$, 25 ºC

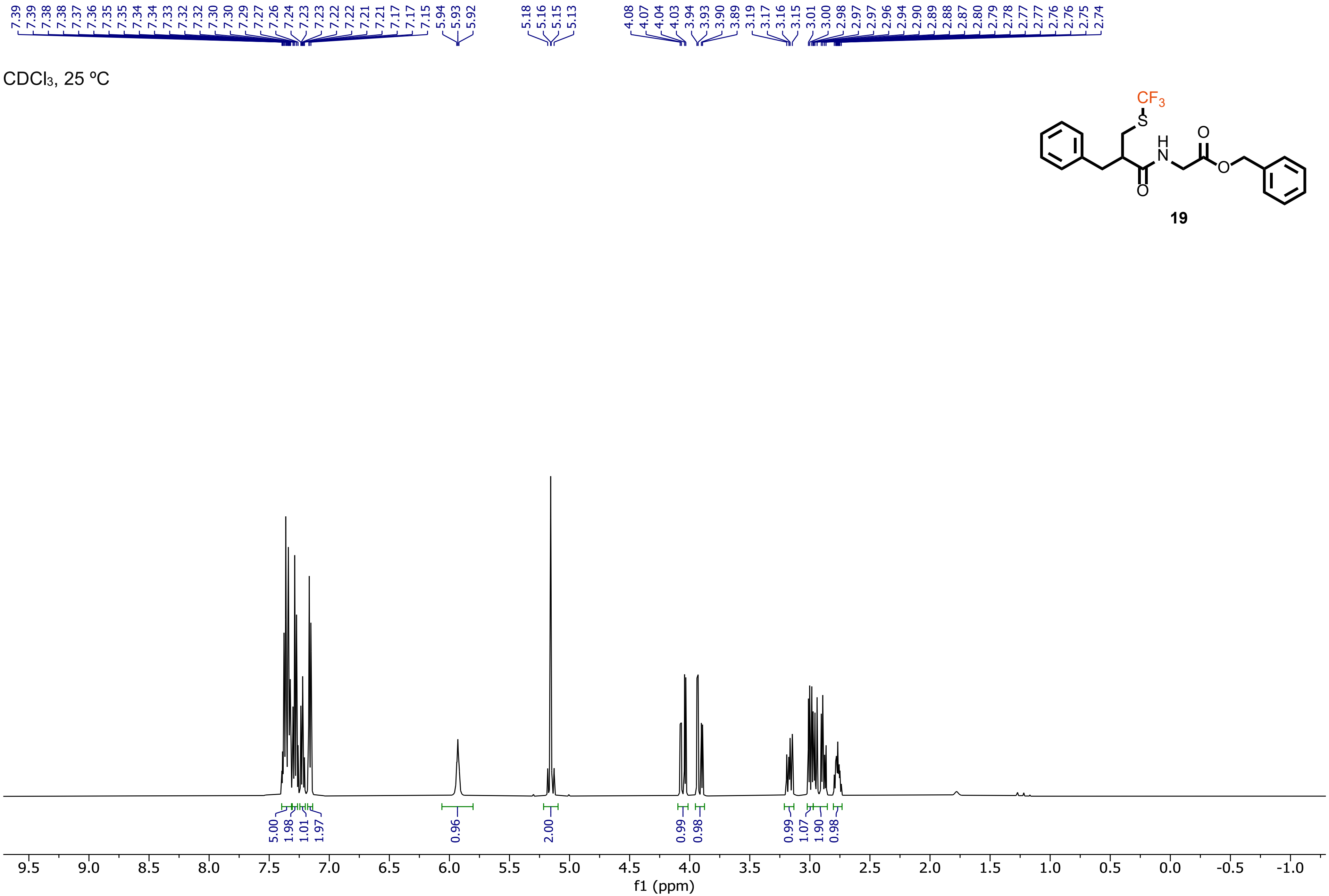

^{19}F NMR of racecadotril derivative 19

CDCl$_3$, 25 ºC

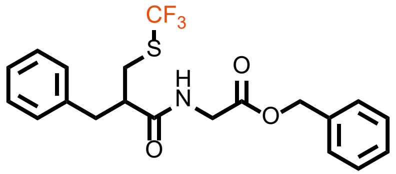

19

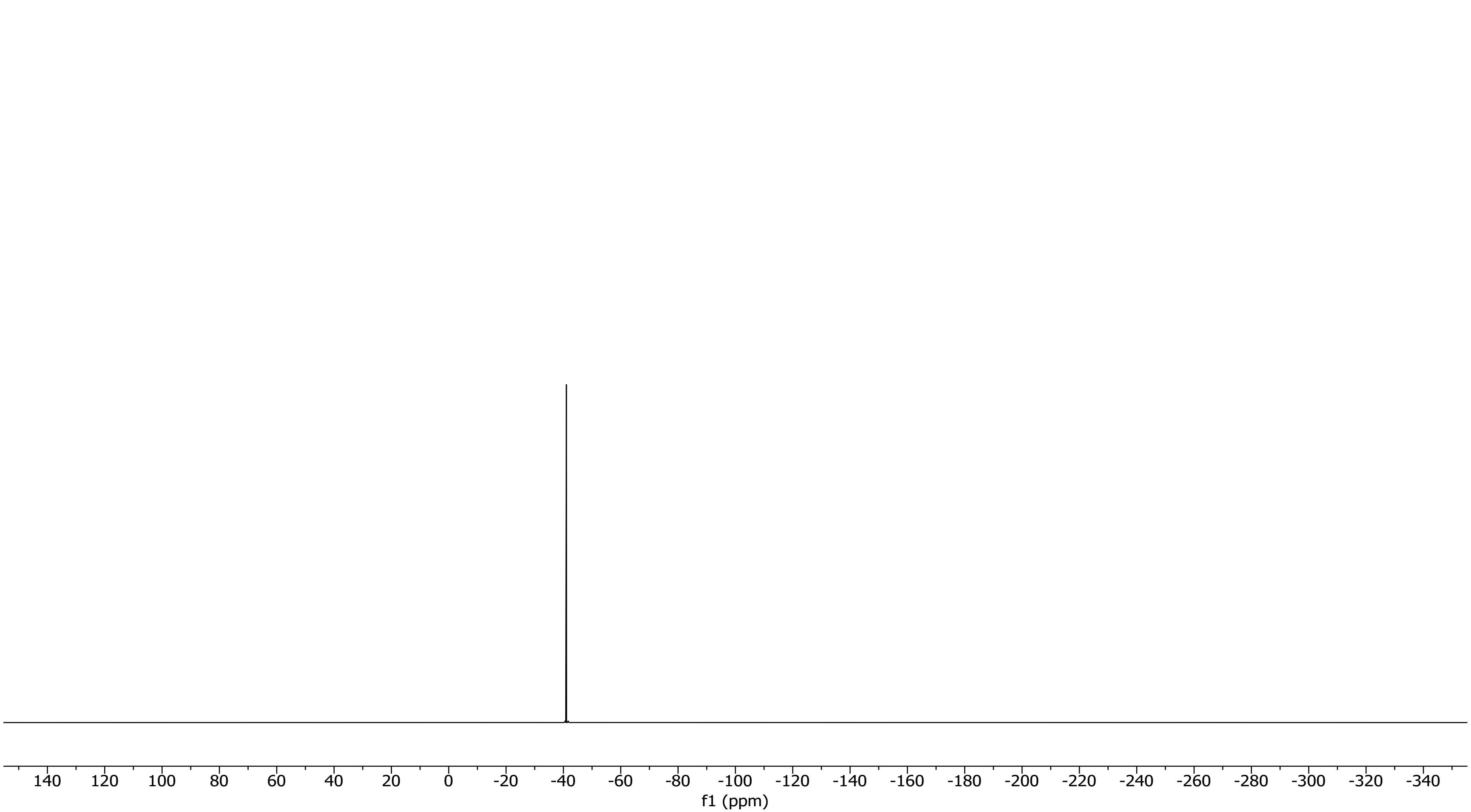

^{13}C NMR of racecadotril derivative 19

CDCl₃, 25 ºC

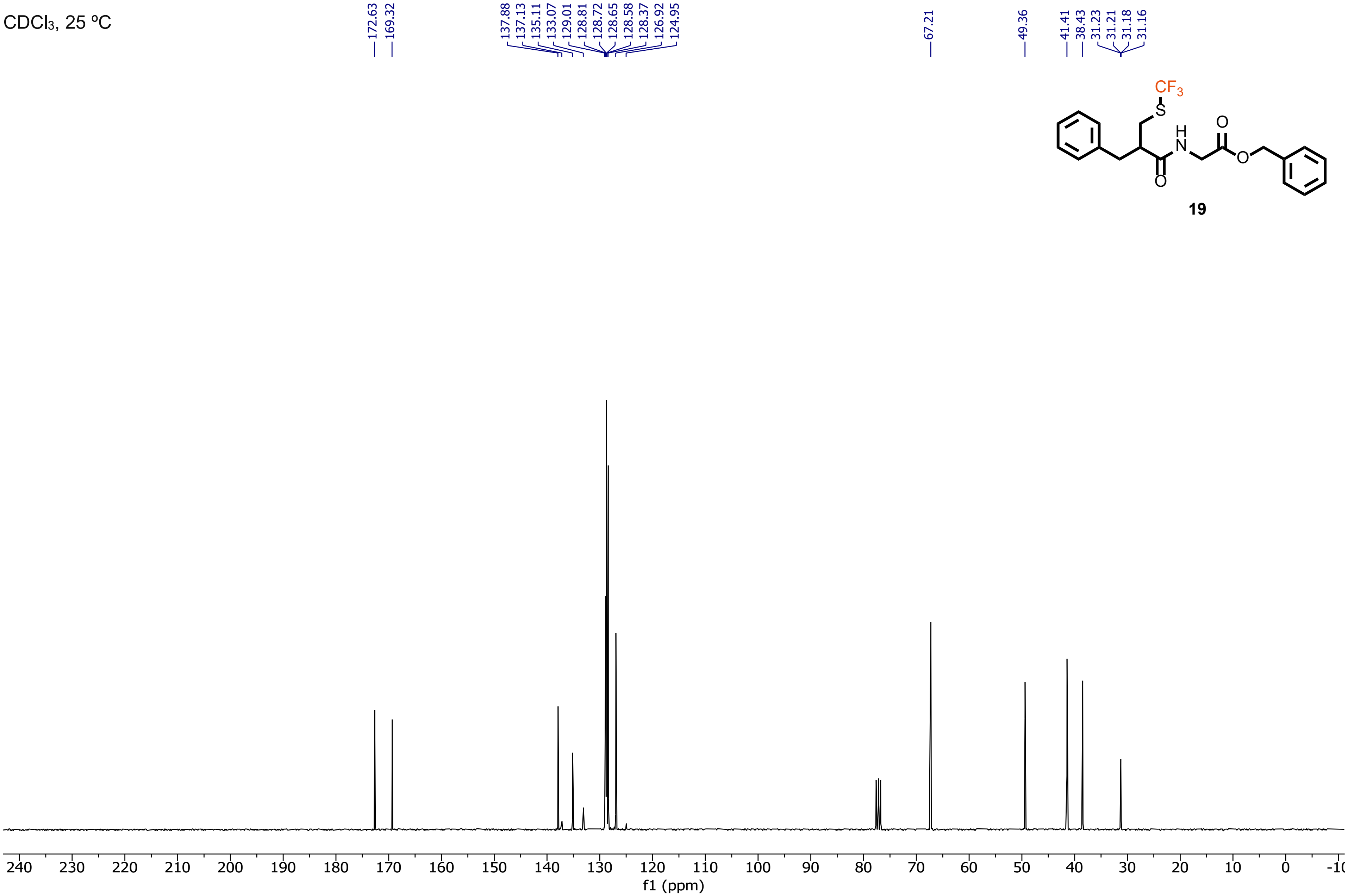

19

1H NMR of 2-(6,6,6-trifluorohexyl)isoindoline-1,3-dione (23)

$CDCl_3$, 25 ºC

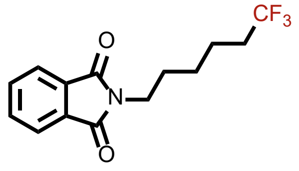

23

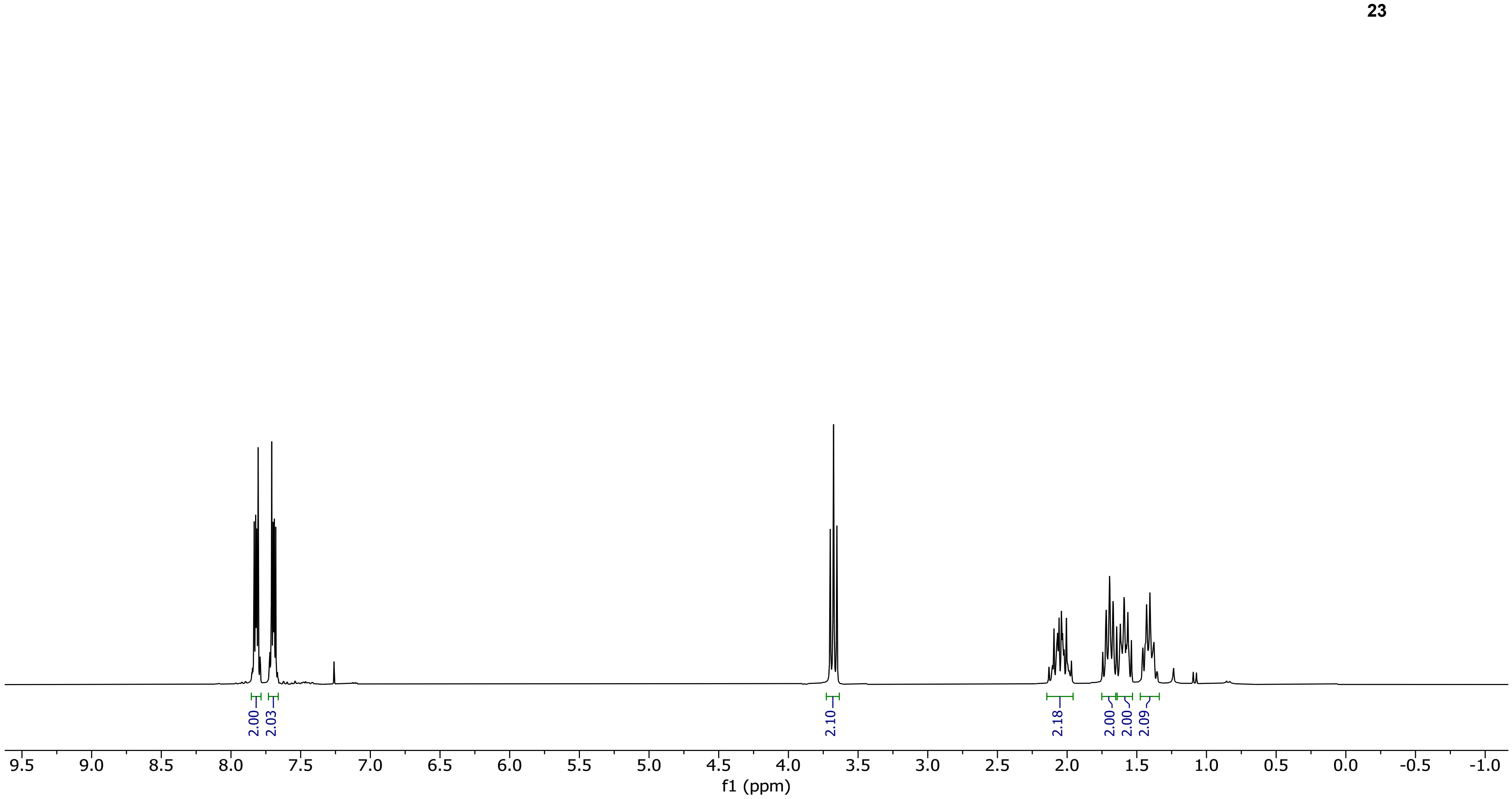

^{19}F NMR of 2-(6,6,6-trifluorohexyl)isoindoline-1,3-dione (23)

CDCl$_3$, 25 ºC

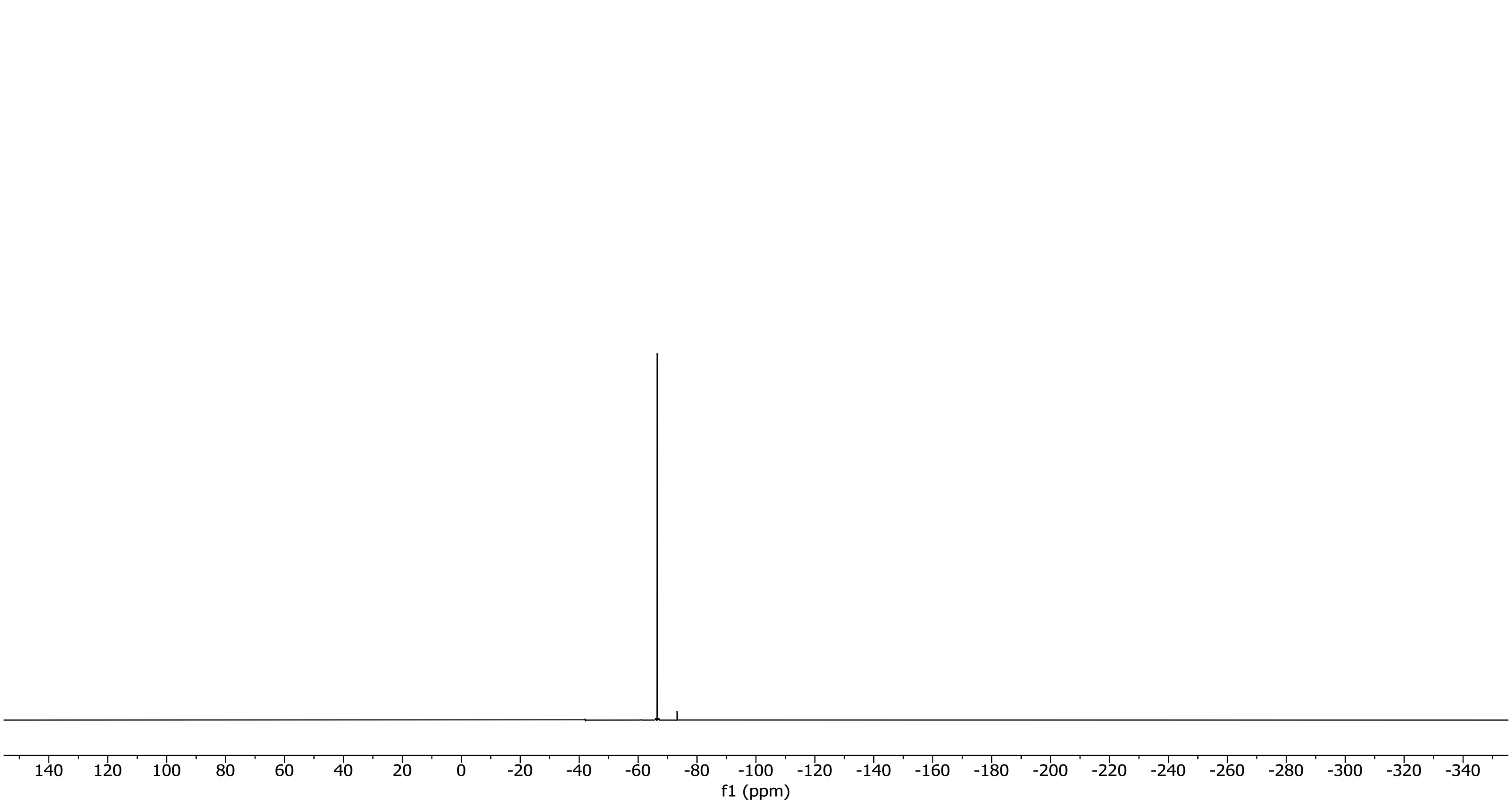

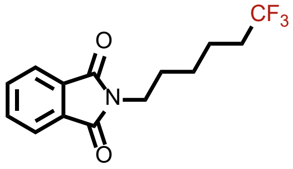

23

^{13}C NMR of 2-(6,6,6-trifluorohexyl)isoindoline-1,3-dione (23)

$CDCl_3$, 25 ºC

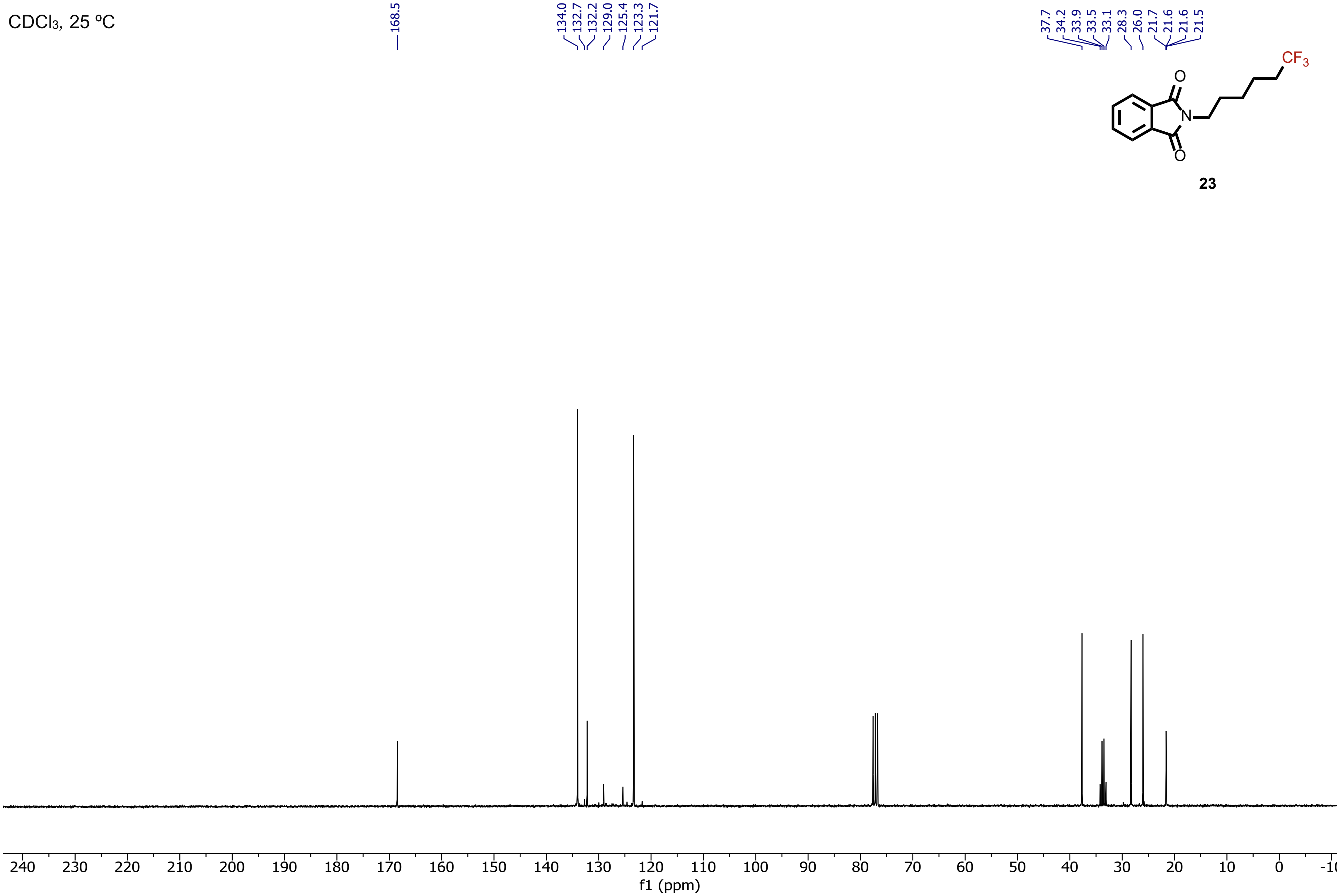

^{1}H NMR of *N*-(5,5,5-trifluoropentyl)benzamide (24)

CDCl3, 25 ºC

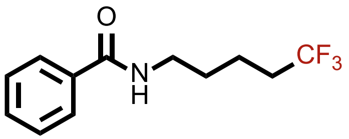

24

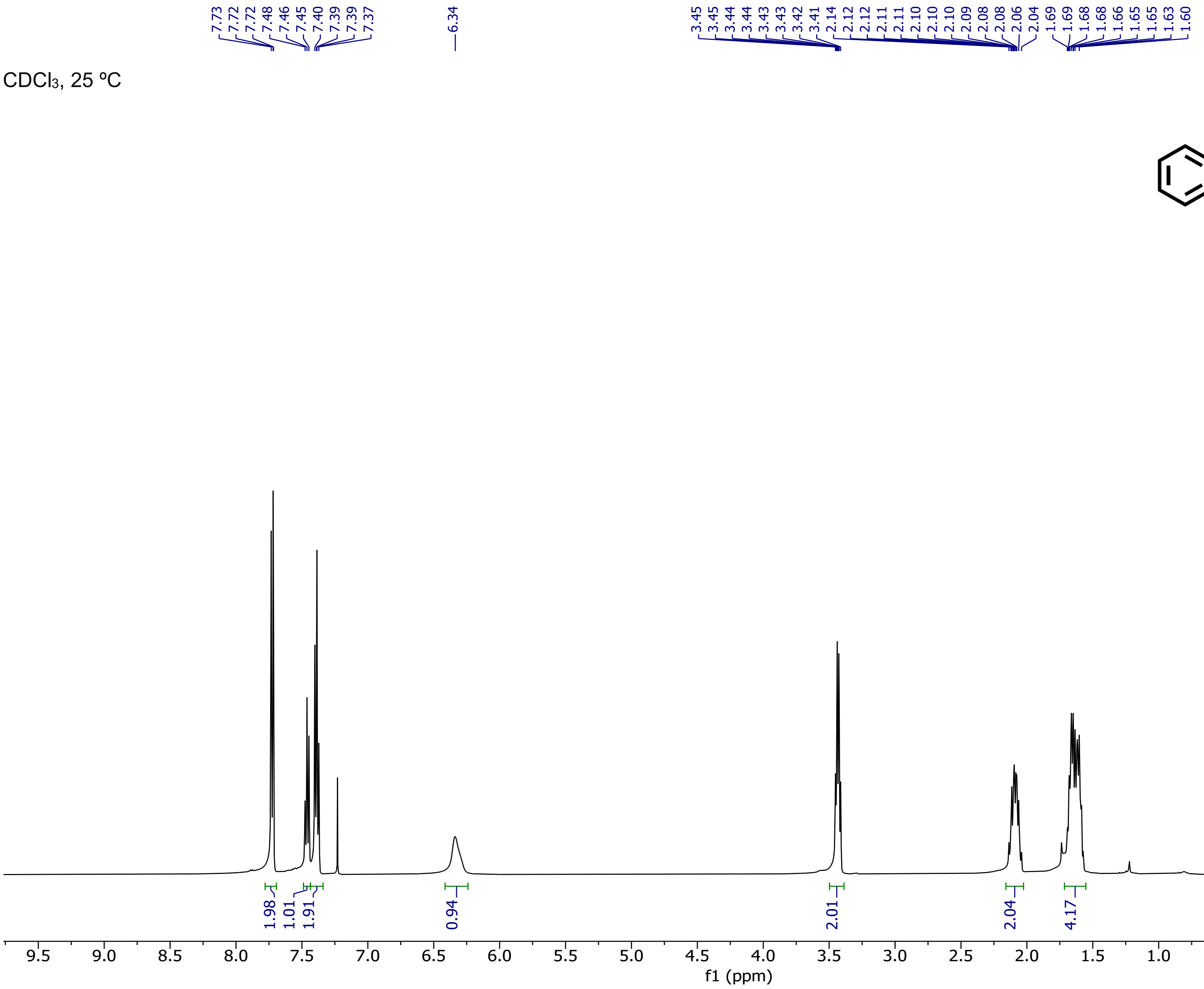

^{19}F NMR of *N*-(5,5,5-trifluoropentyl)benzamide (24)

$CDCl_3$, 25 ºC

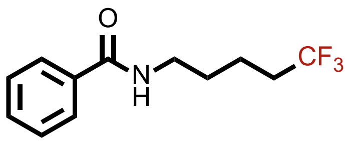

24

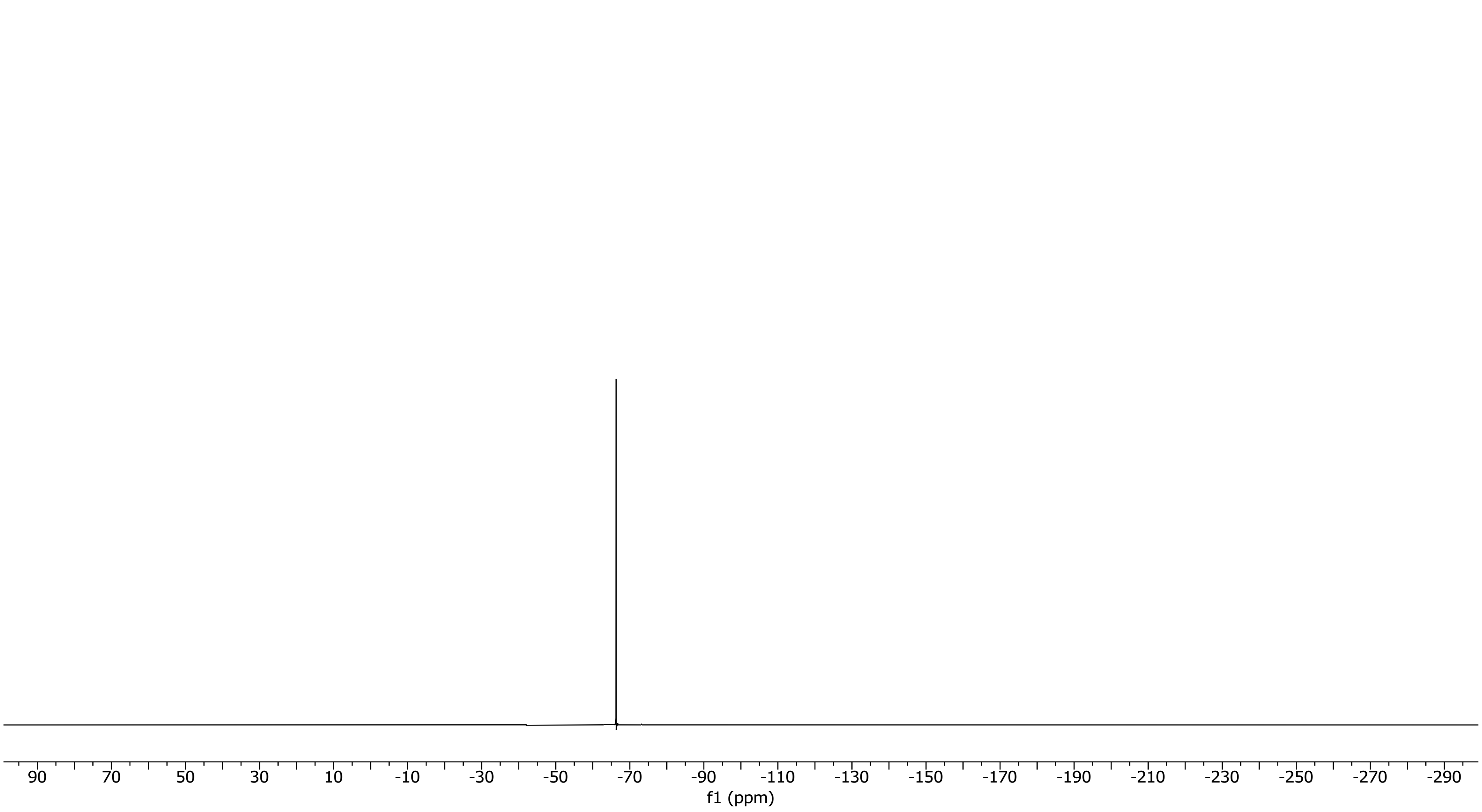

^{13}C NMR of *N*-(5,5,5-trifluoropentyl)benzamide (24)

$CDCl_3$, 25 ºC

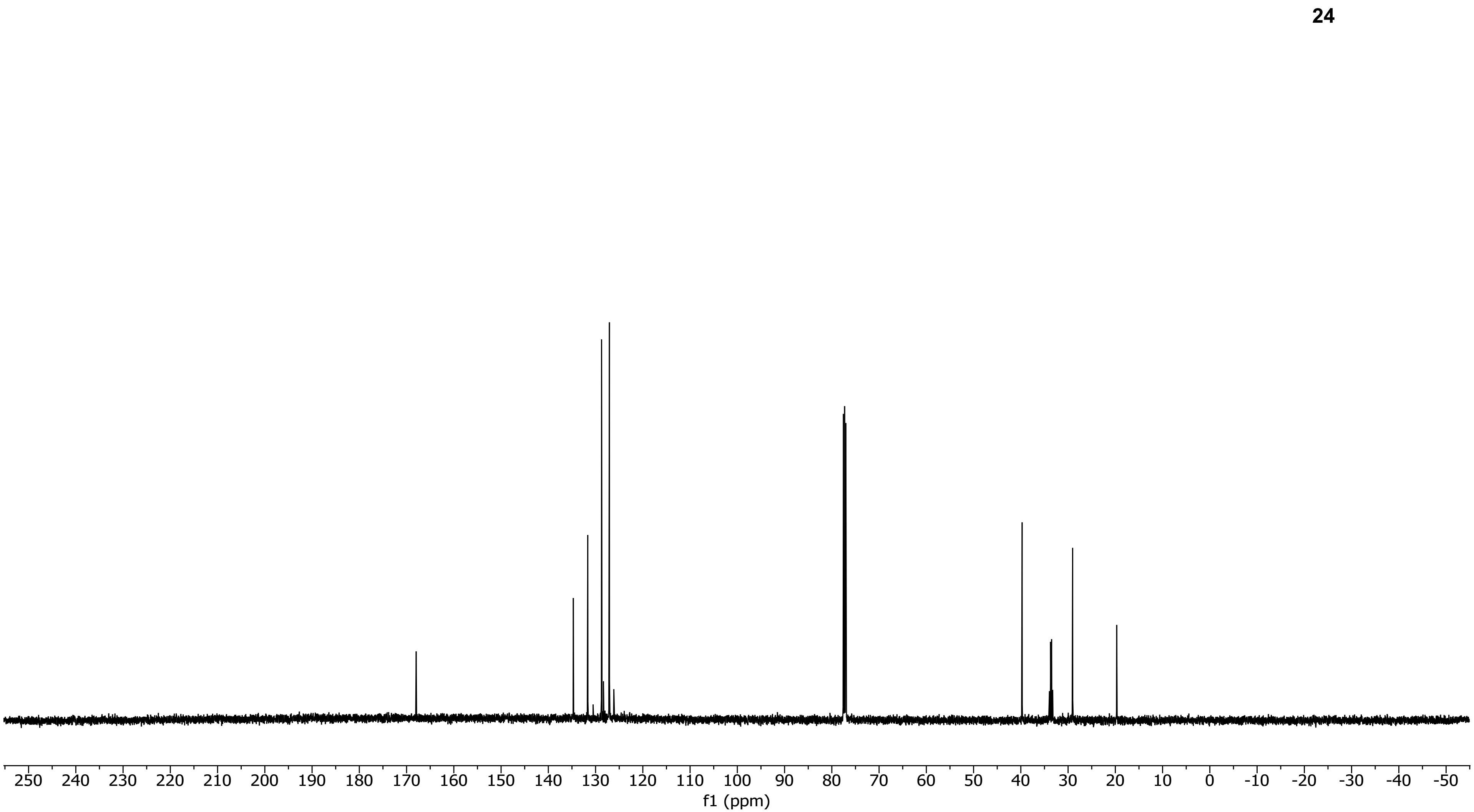

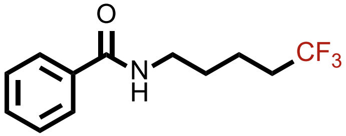

24

^{1}H NMR of *N*-(4-chloro-2-fluorophenyl)-6,6,6-trifluorohexanamide (25)

$CDCl_3$, 25 ºC

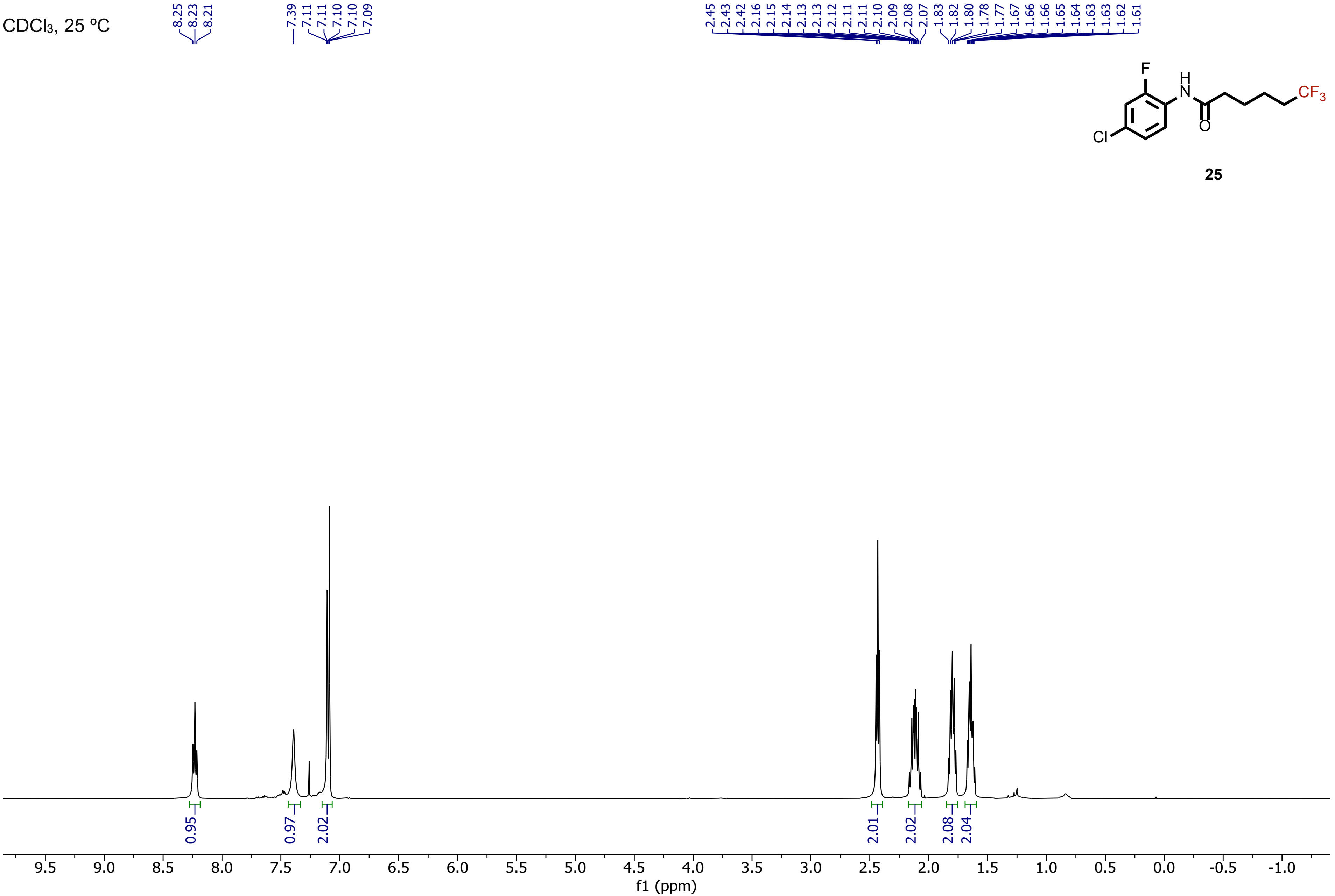

25

^{19}F NMR of *N*-(4-chloro-2-fluorophenyl)-6,6,6-trifluorohexanamide (25)

$CDCl_3$, 25 ºC

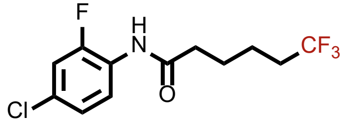

25

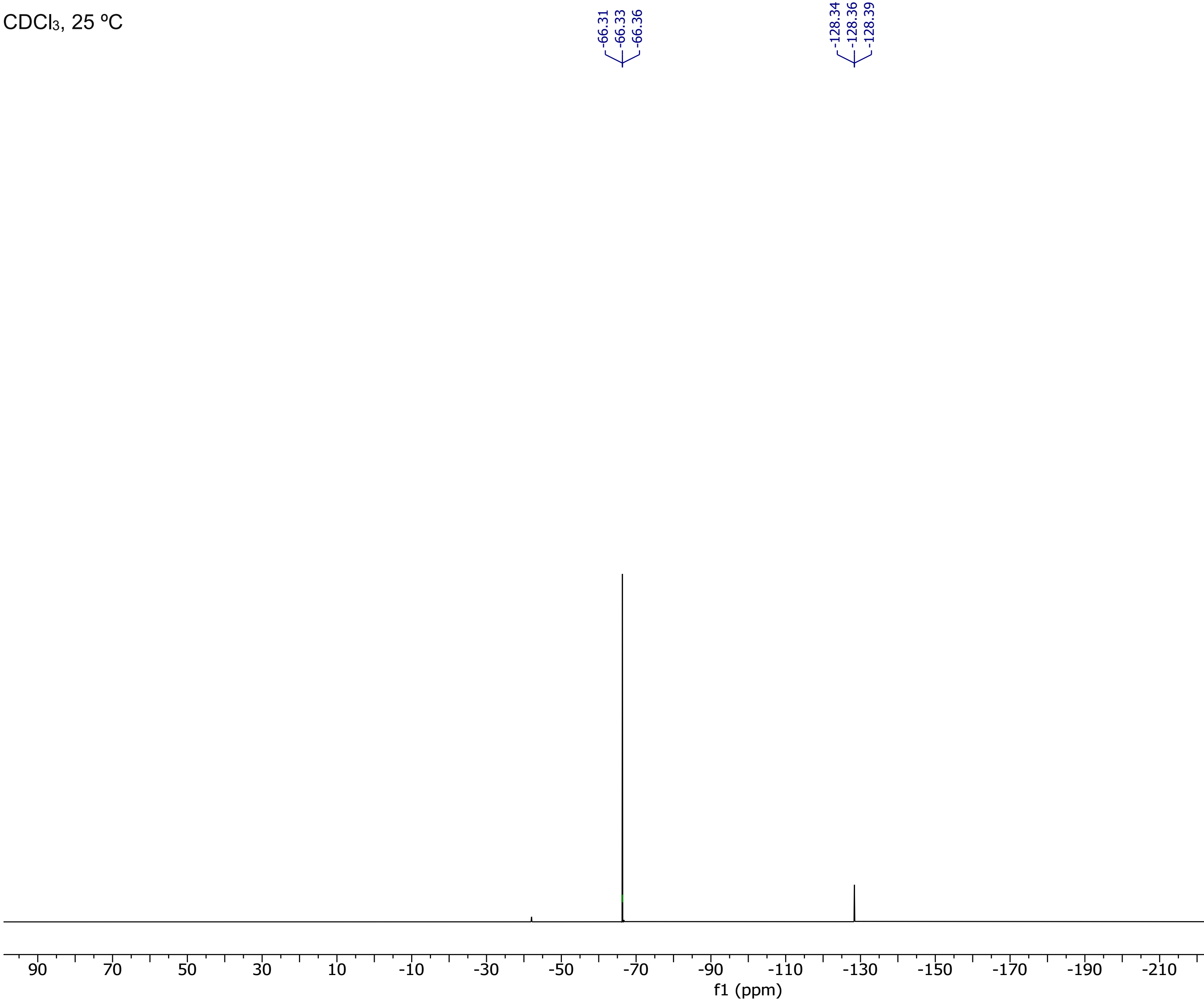

^{13}C NMR of *N*-(4-chloro-2-fluorophenyl)-6,6,6-trifluorohexanamide (25)

CDCl$_3$, 25 ºC

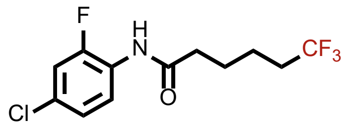

25

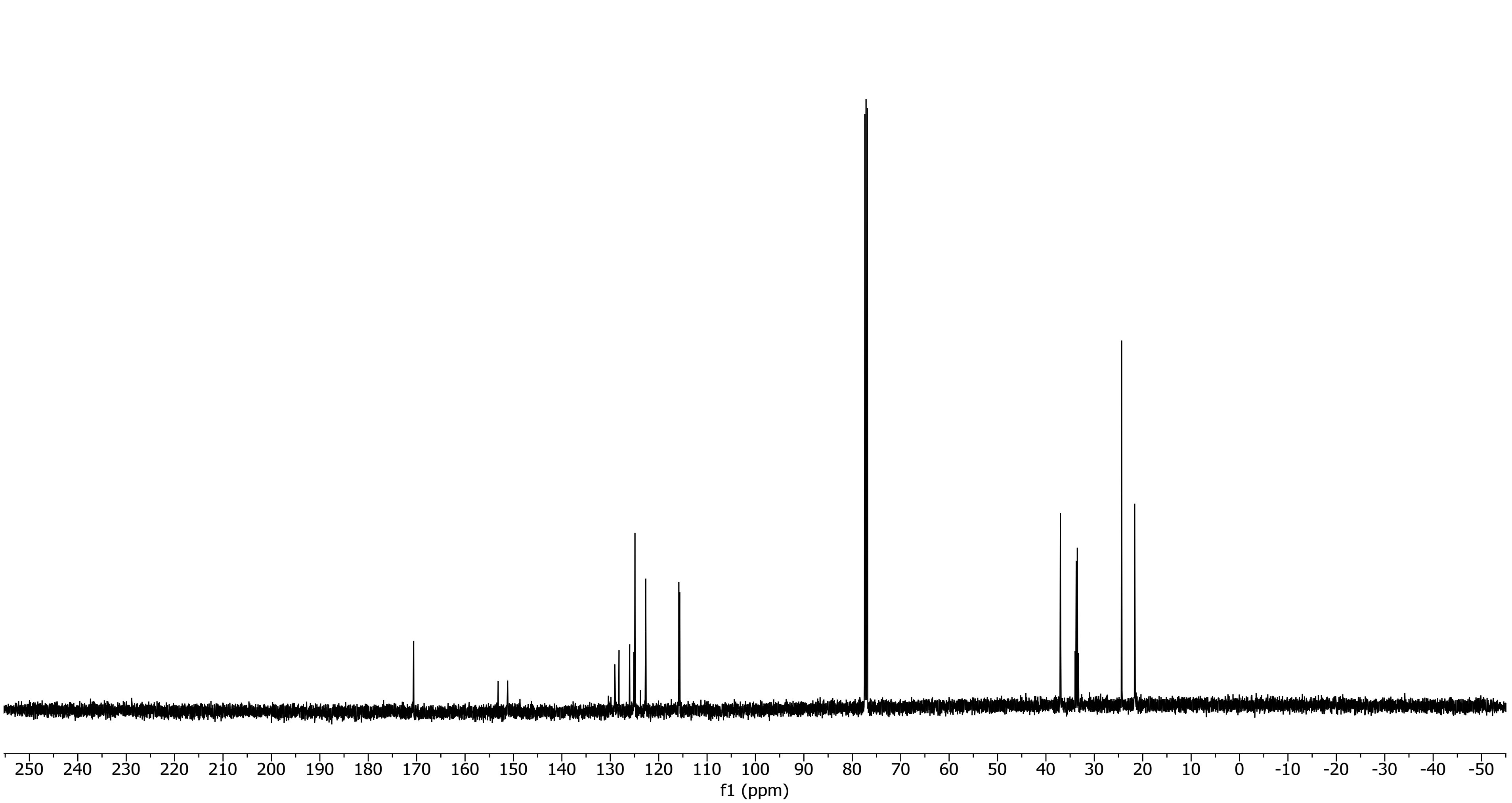

^{1}H NMR of CF_3-(1*S*)-10-camphorsulfonamide (26)

CDCl$_3$, 25 ºC

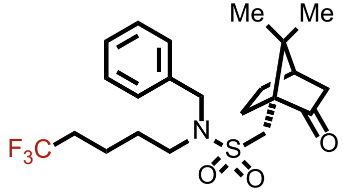

26

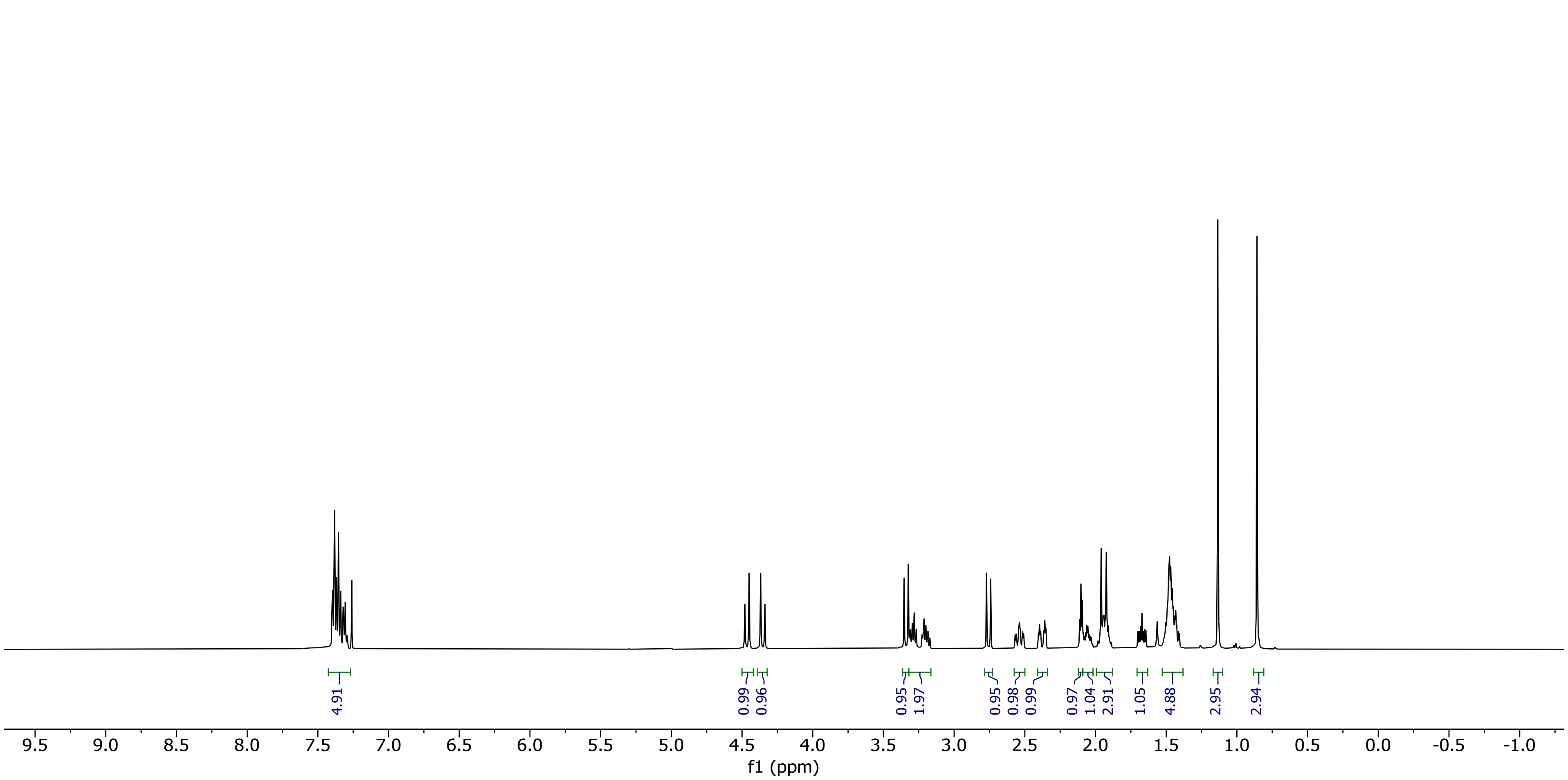

^{19}F NMR of CF_3-(1*S*)-10-camphorsulfonamide (26)

$CDCl_3$, 25 ºC

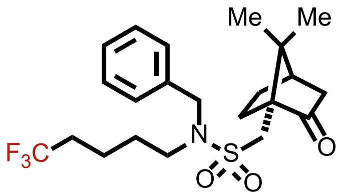

26

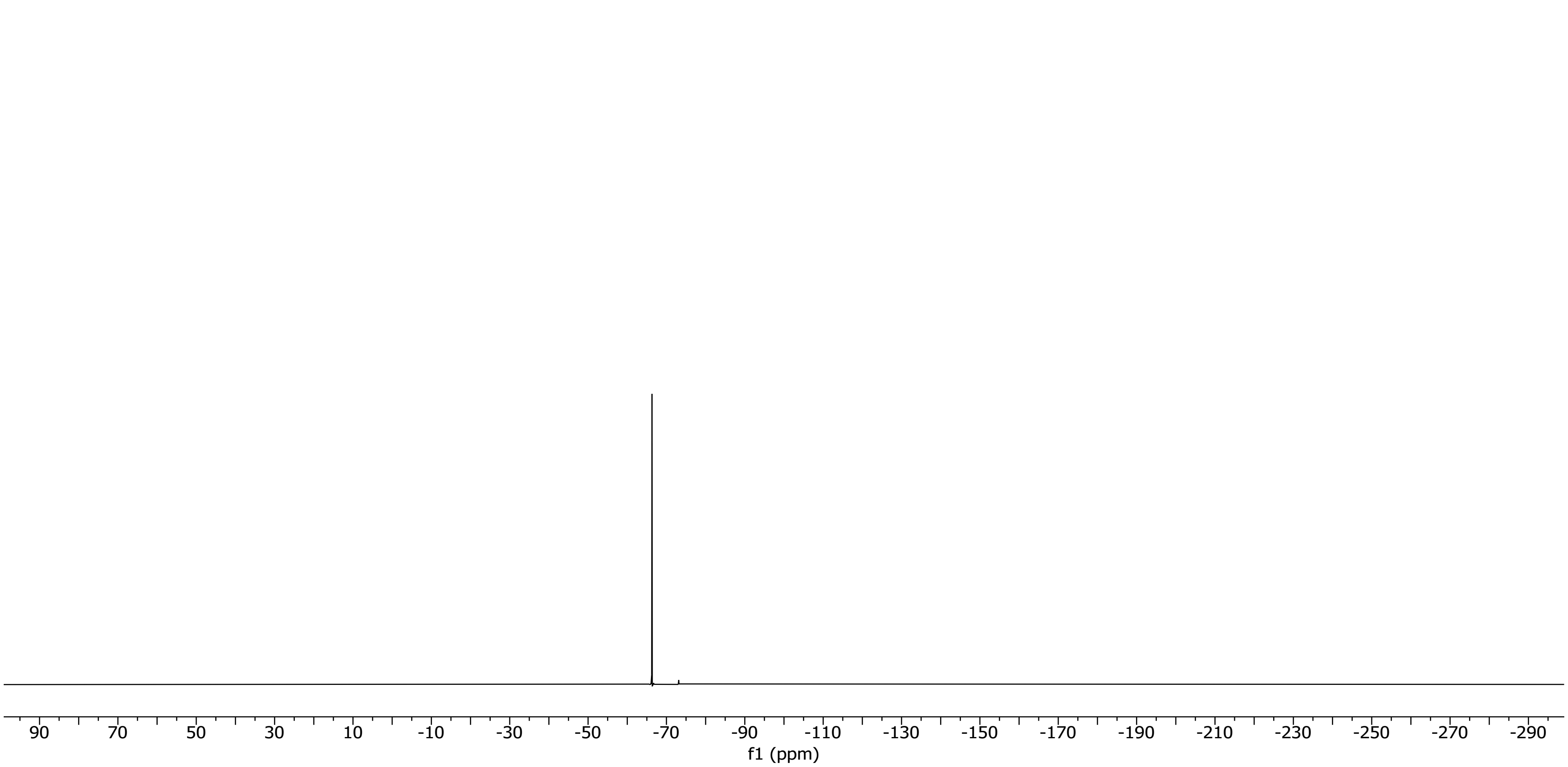

^{13}C NMR of CF_3-(1*S*)-10-camphorsulfonamide (26)

CDCl$_3$, 25 ºC

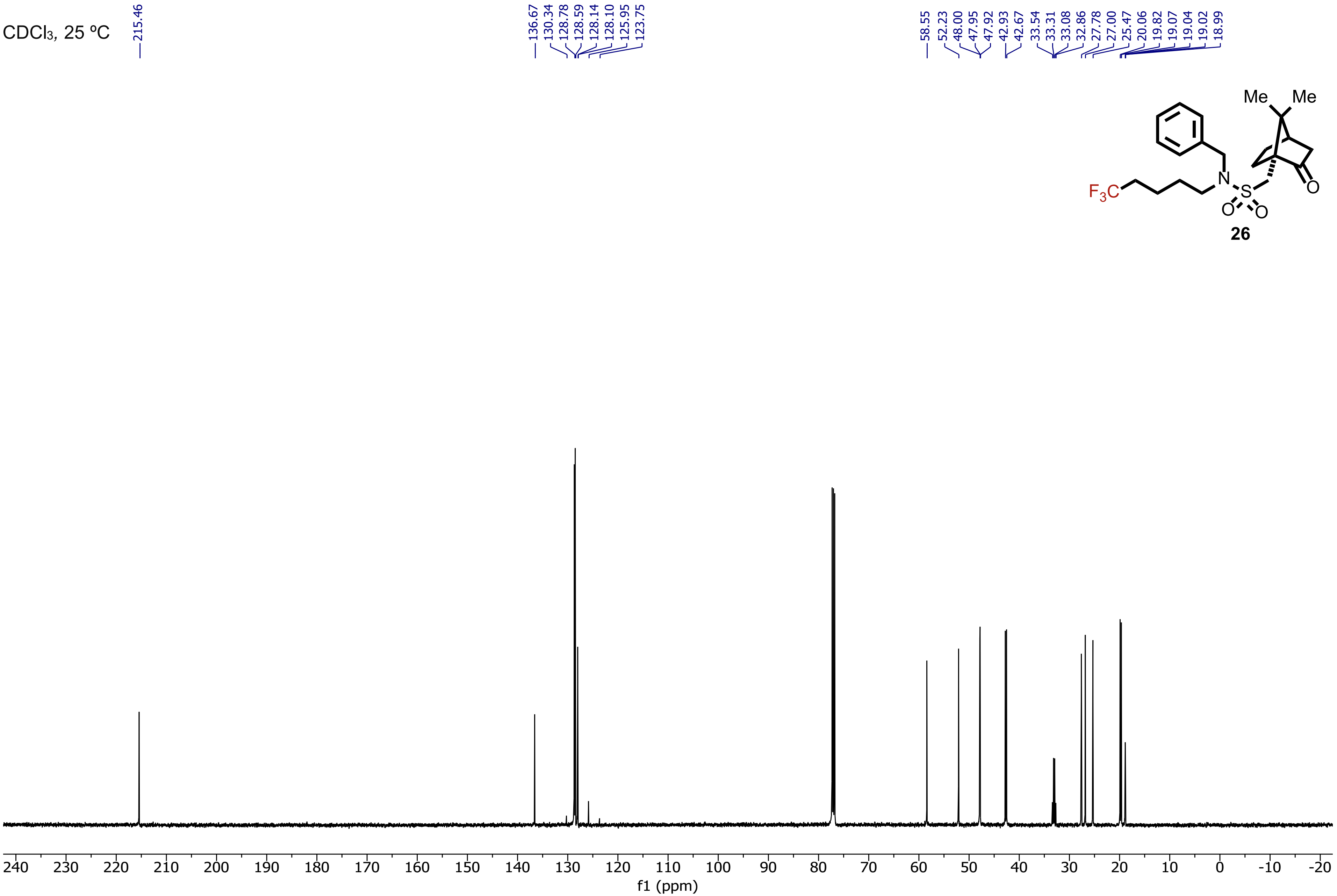

^{1}H NMR of CF_3-Fmoc-L-Nle-OH derivative 27

$CDCl_3$, 25 ºC

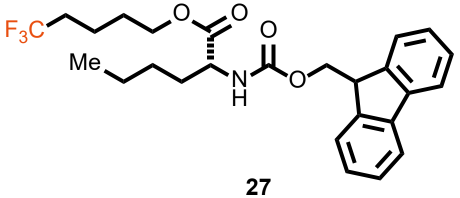

27

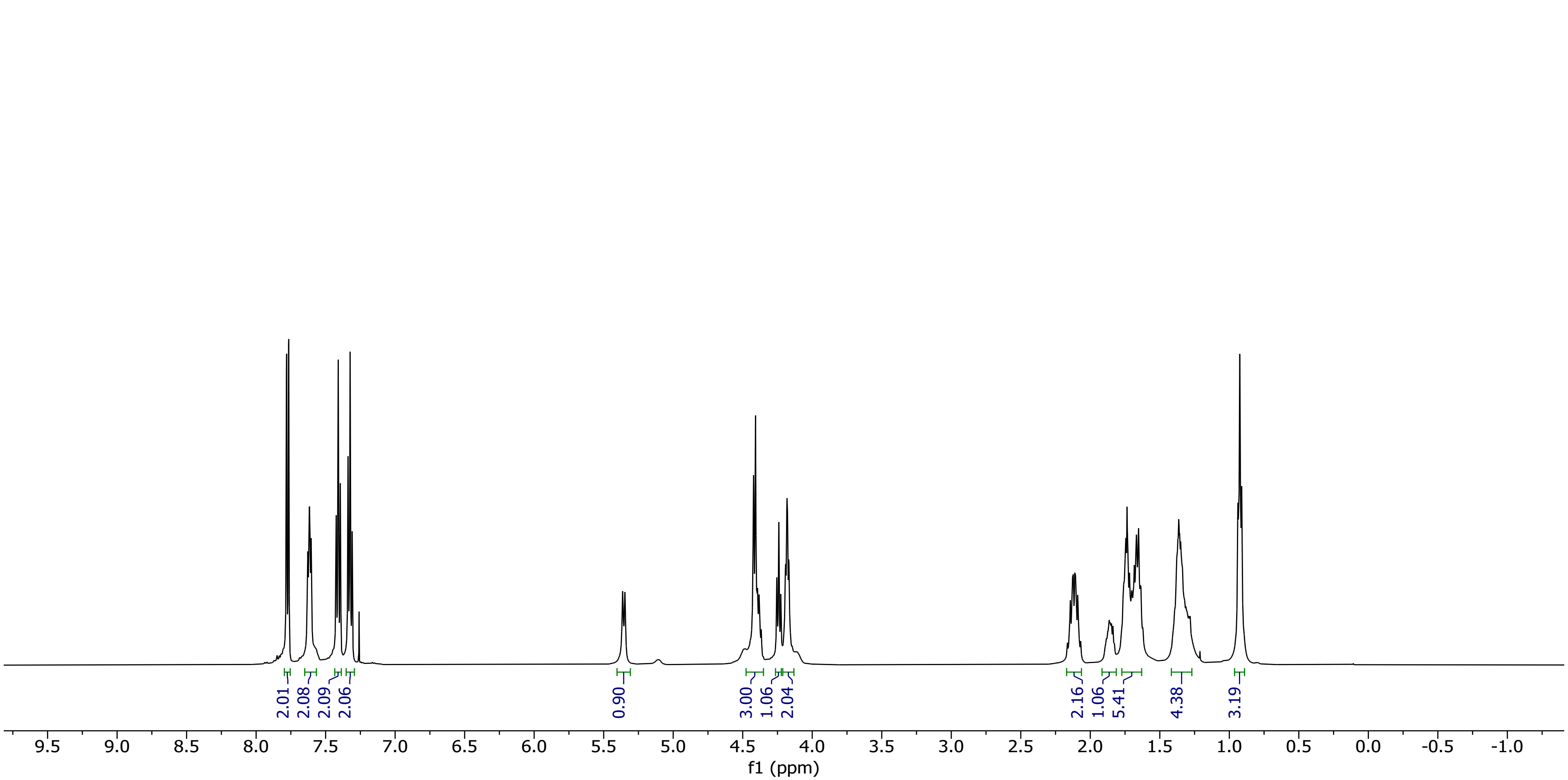

^{19}F NMR of CF_3-Fmoc-L-Nle-OH derivative 27

$CDCl_3$, 25 ºC

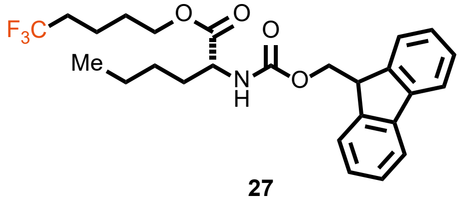

27

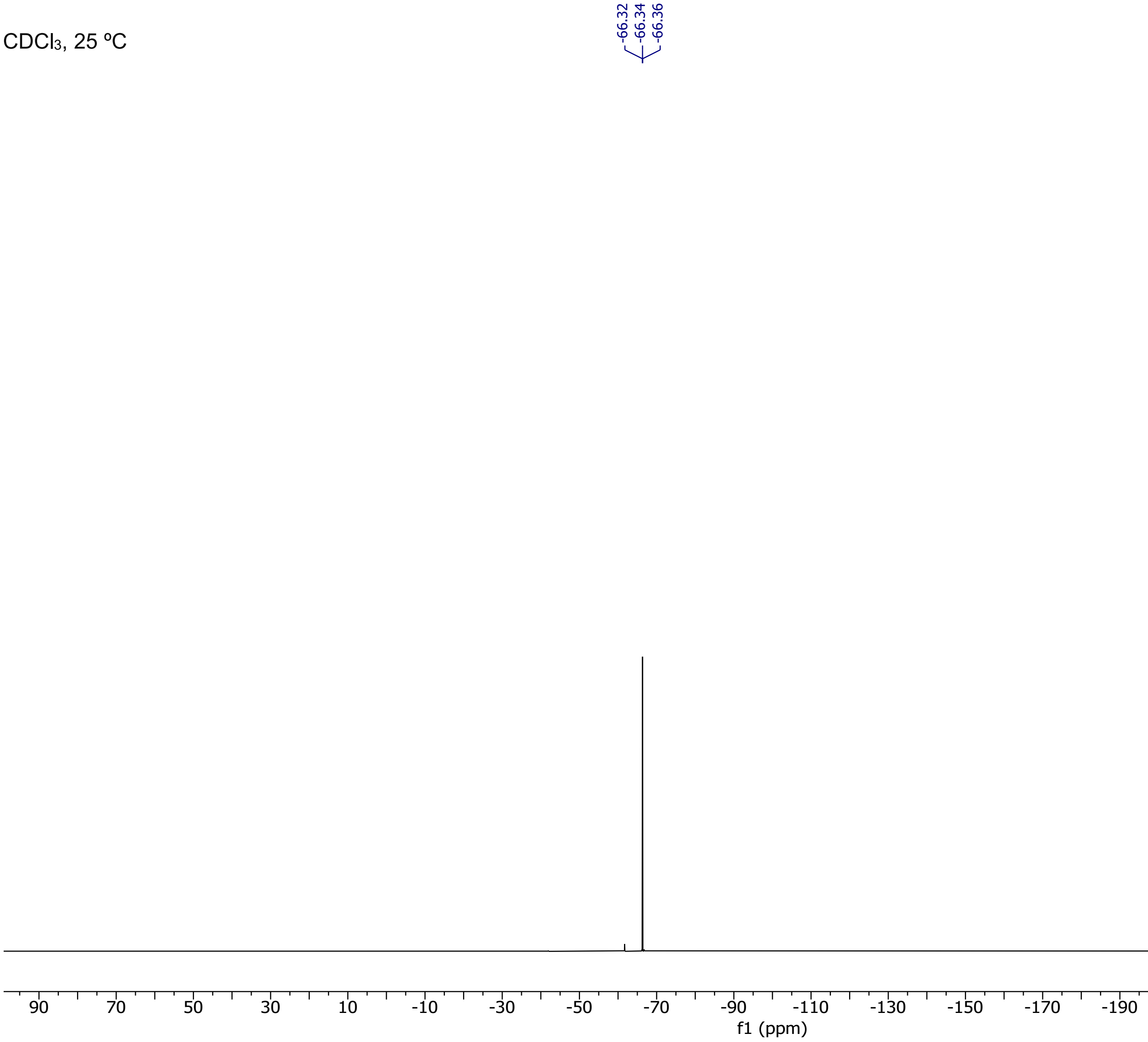

^{13}C NMR of CF_3-Fmoc-L-Nle-OH derivative 27

$CDCl_3$, 25 ºC

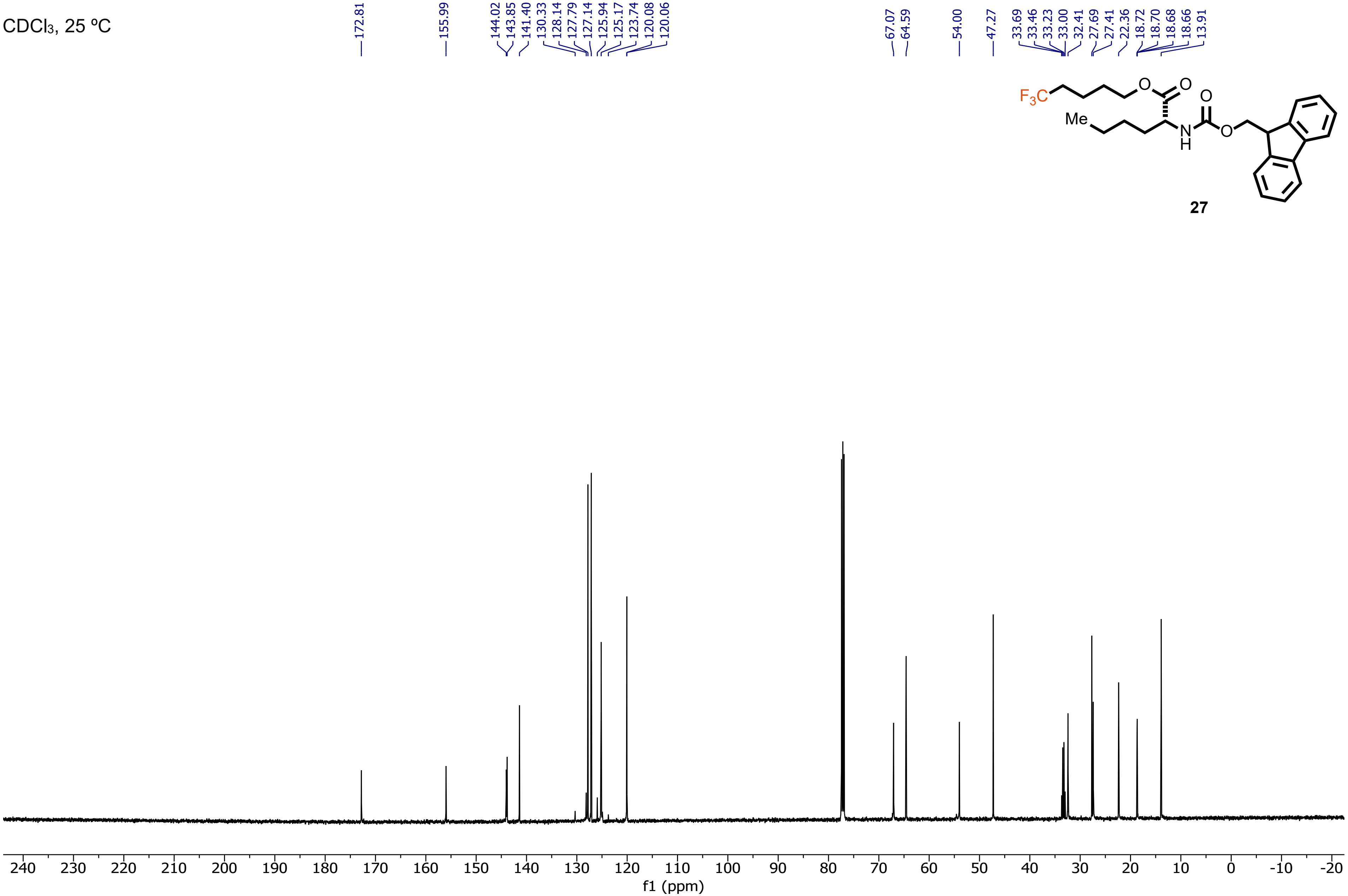

27

^{1}H NMR of rotenone derivative 28a

CDCl₃, 25 ºC

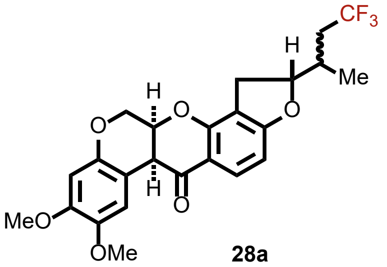

28a

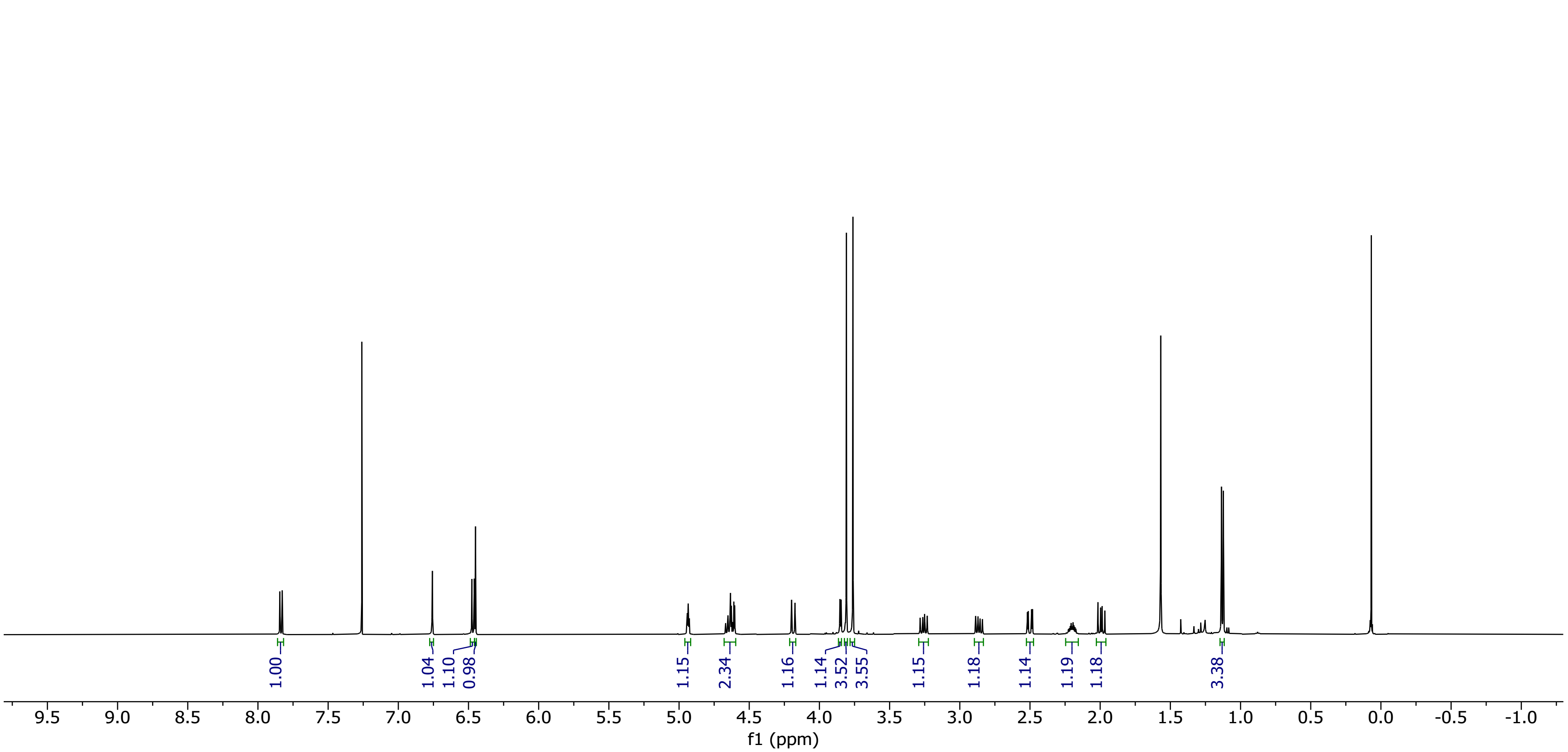

^{19}F NMR of rotenone derivative 28a

$CDCl_3$, 25 ºC

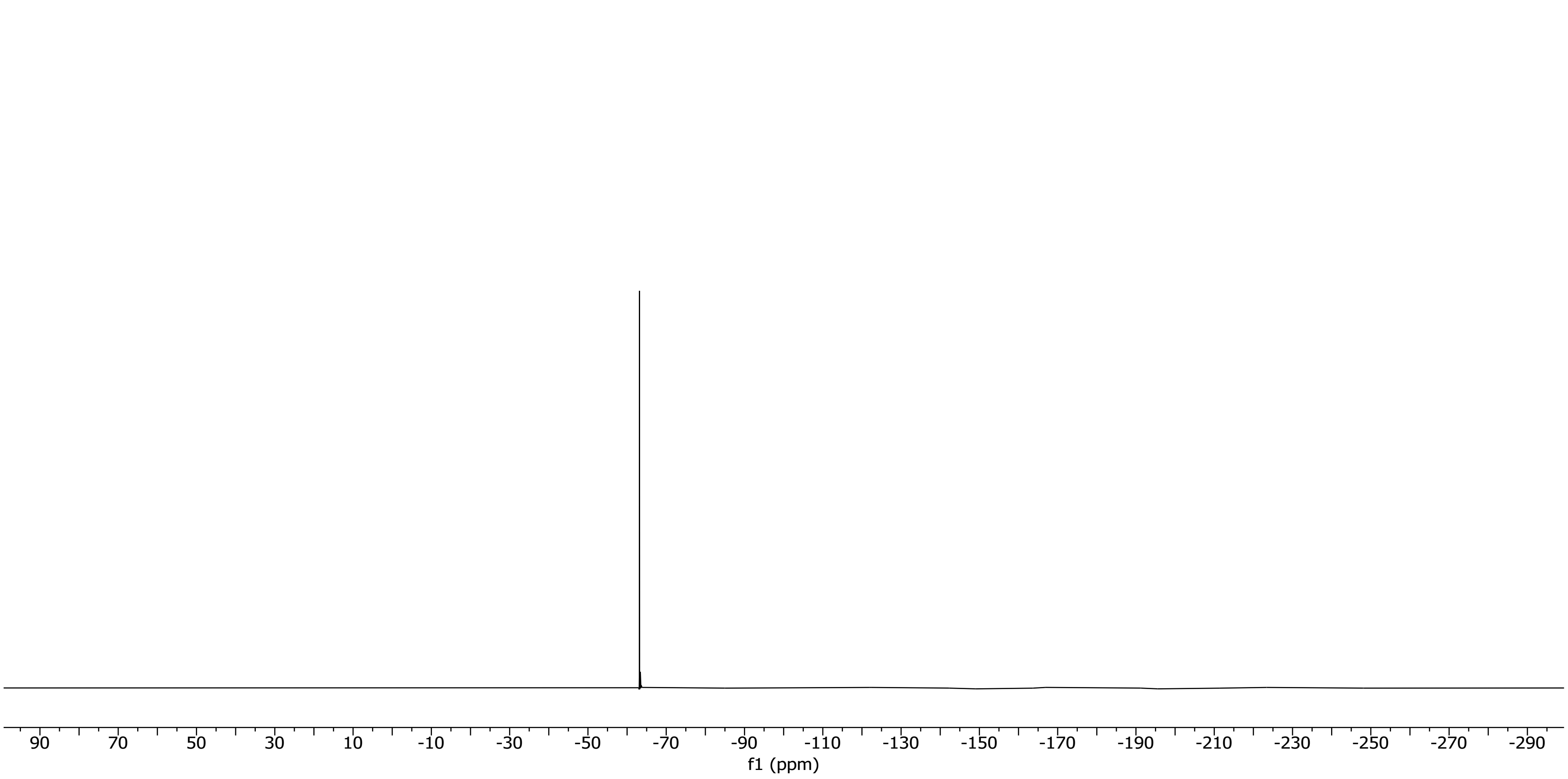

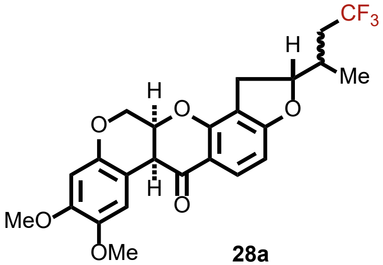

28a

^{13}C NMR of rotenone derivative 28a

CDCl$_3$, 25 ºC

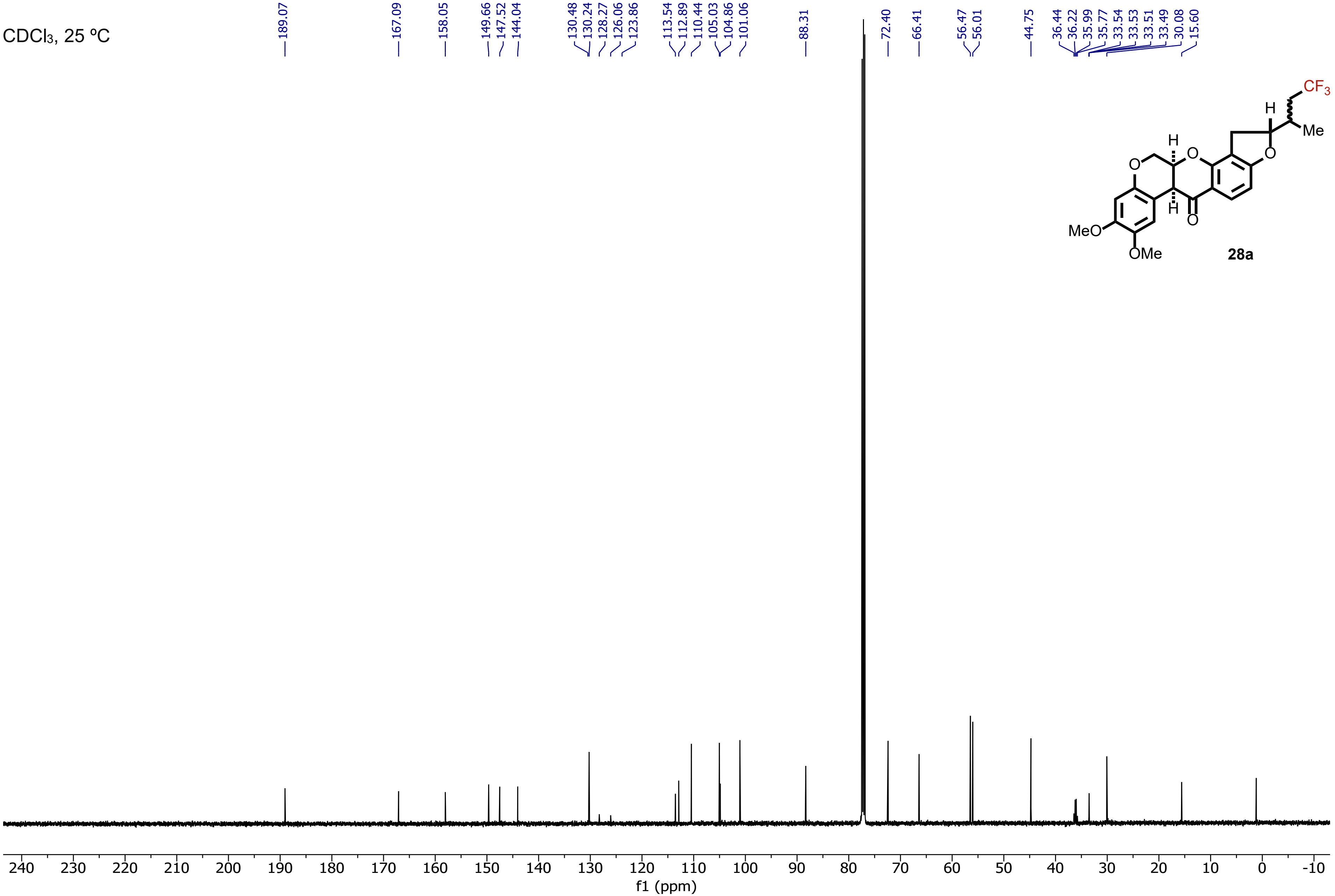

28a

HMQC of rotenone derivative 28a

CDCl3, 25 ºC

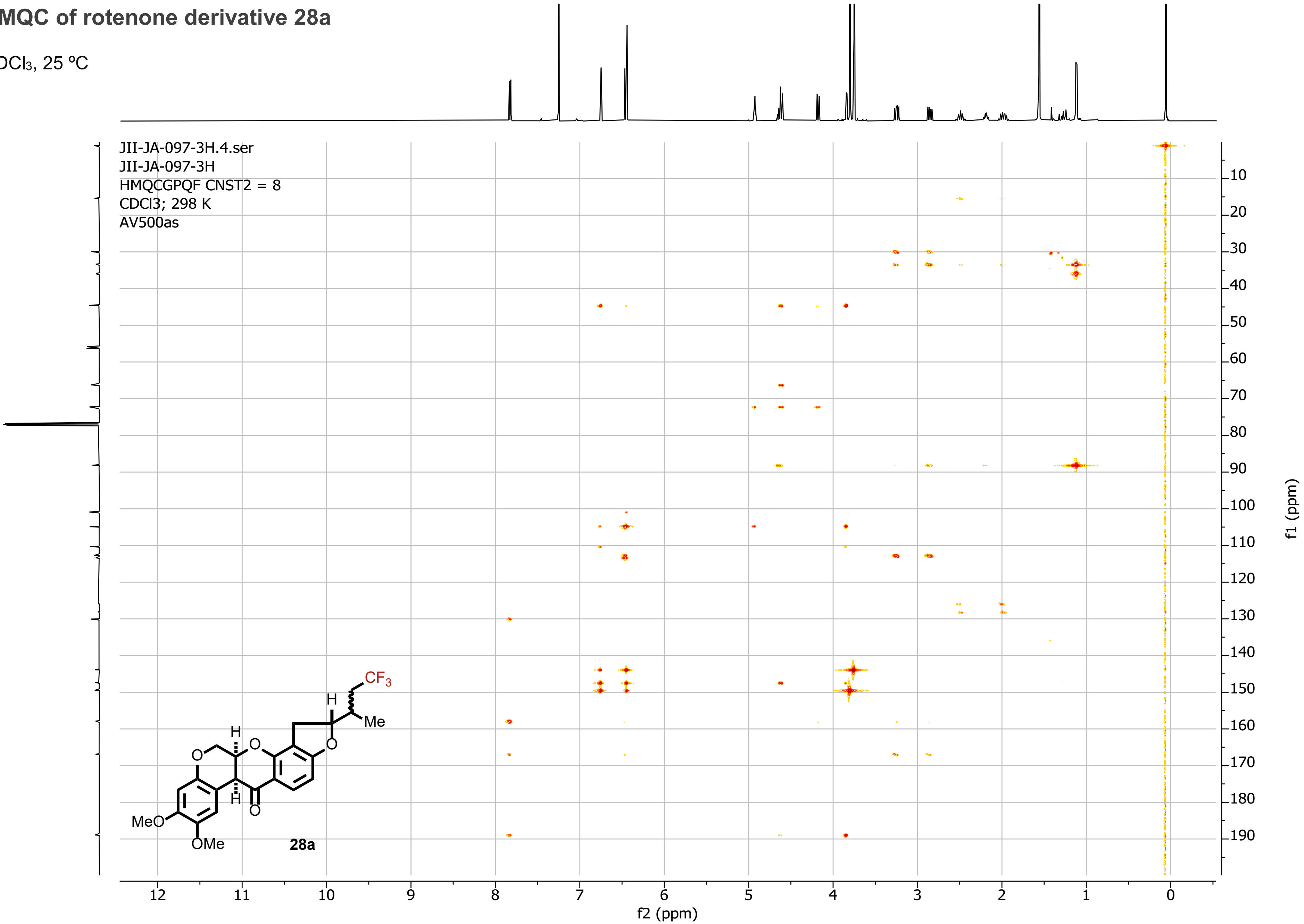

HSQC of rotenone derivative 28a

CDCl₃, 25 ºC

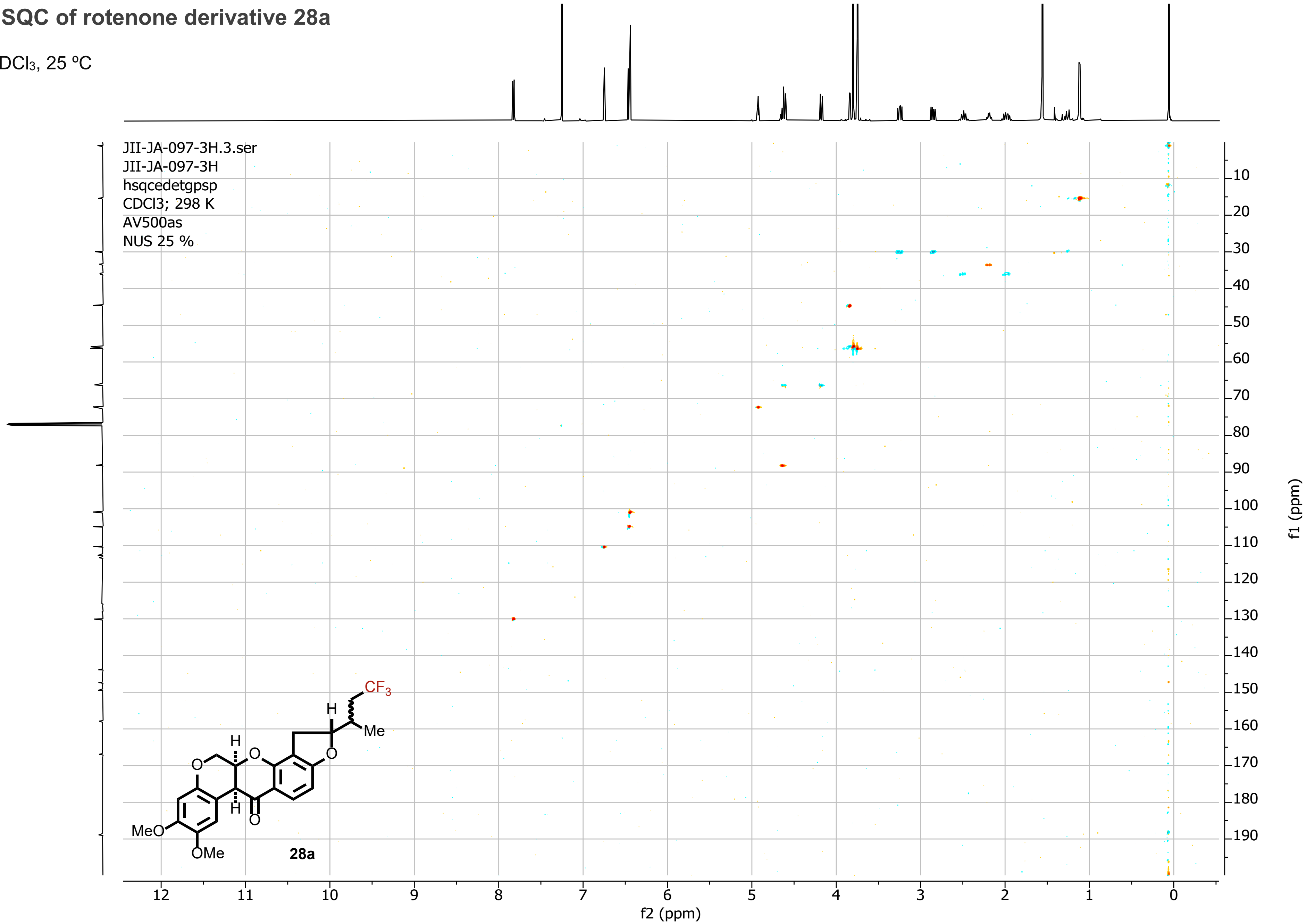

COSY of rotenone derivative 28a

$CDCl_3$, 25 ºC

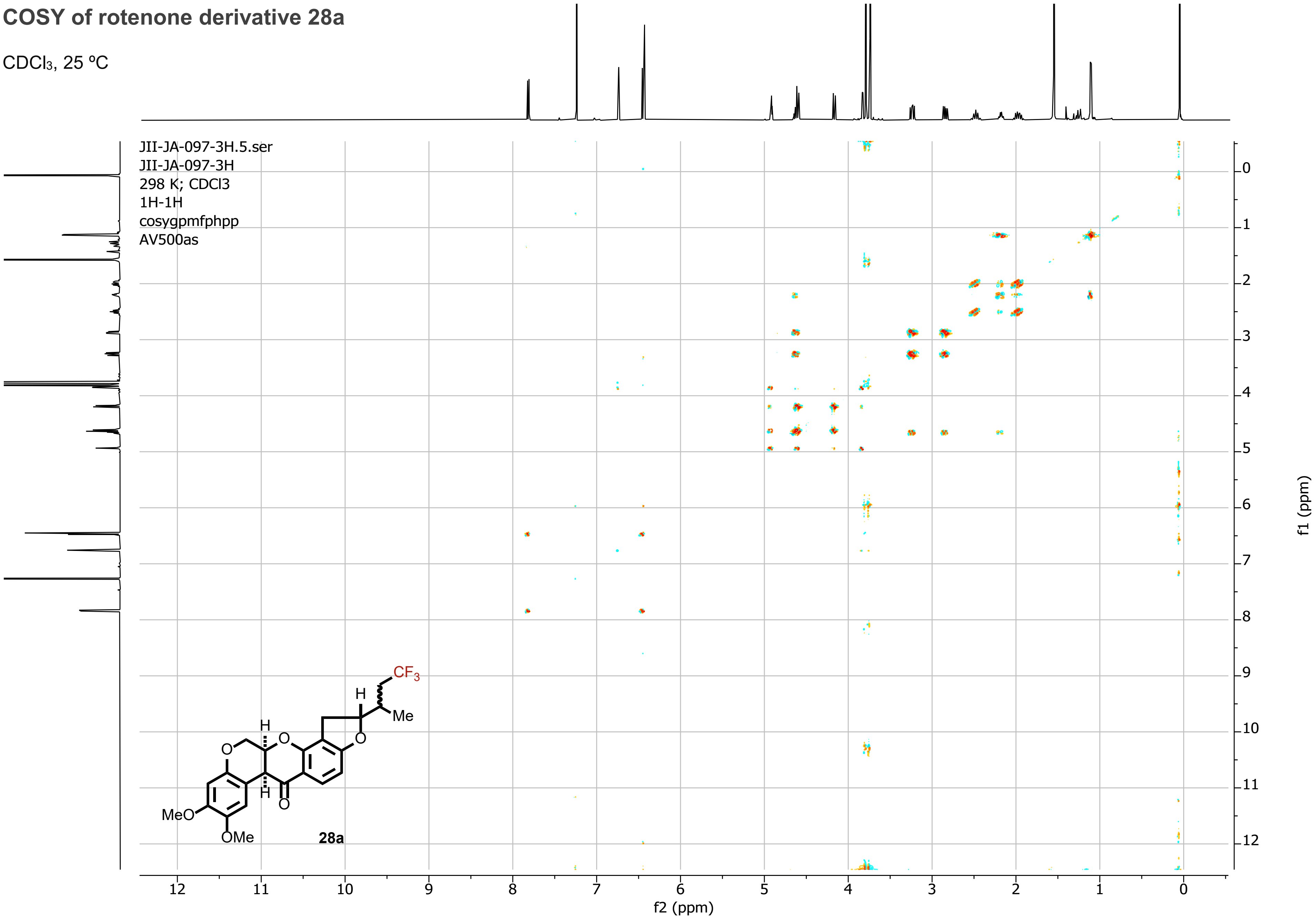

NOESY of rotenone derivative 28a

CDCl3, 25 ºC

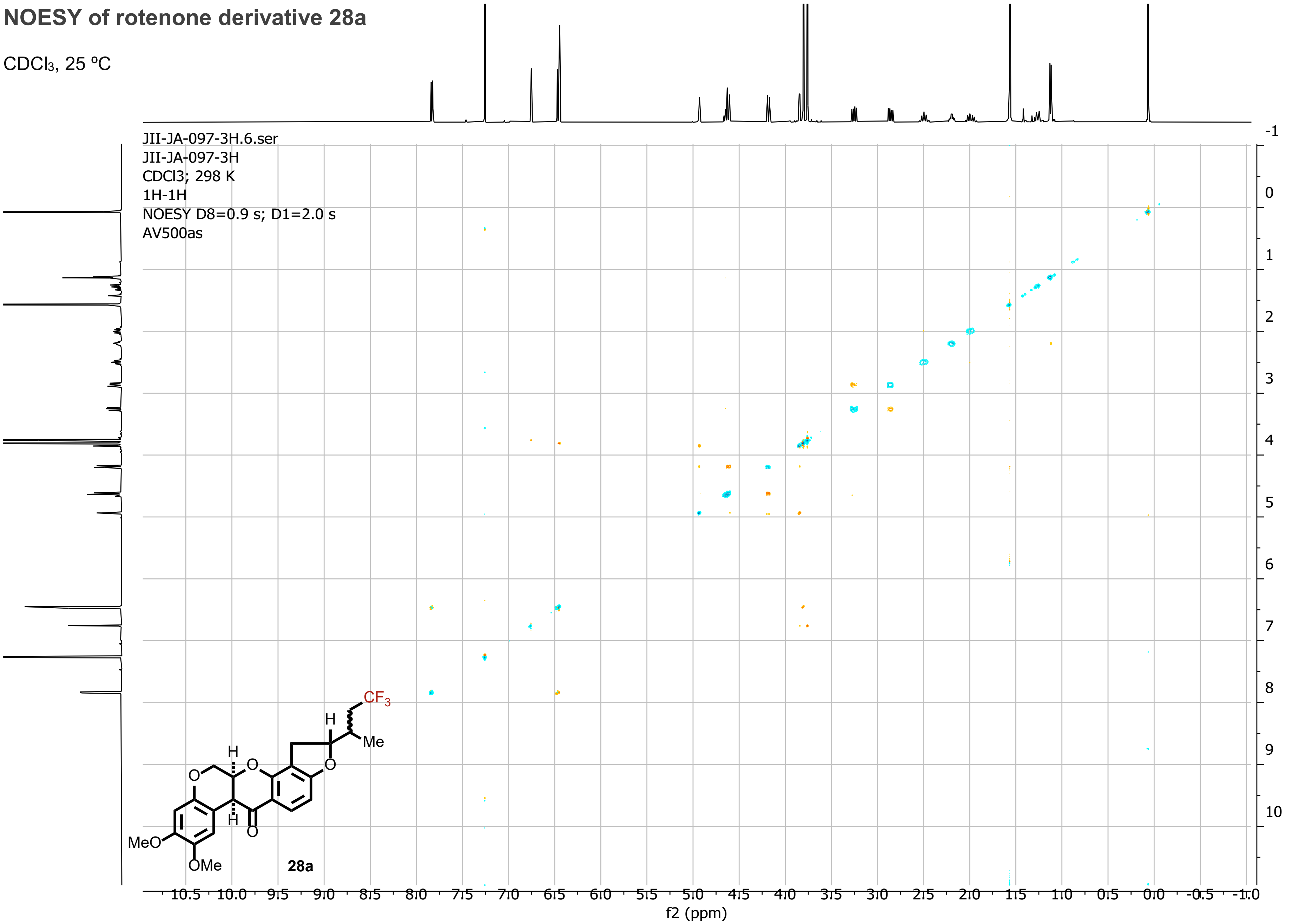

HOESY of rotenone derivative 28a

CDCl3, 25 ºC

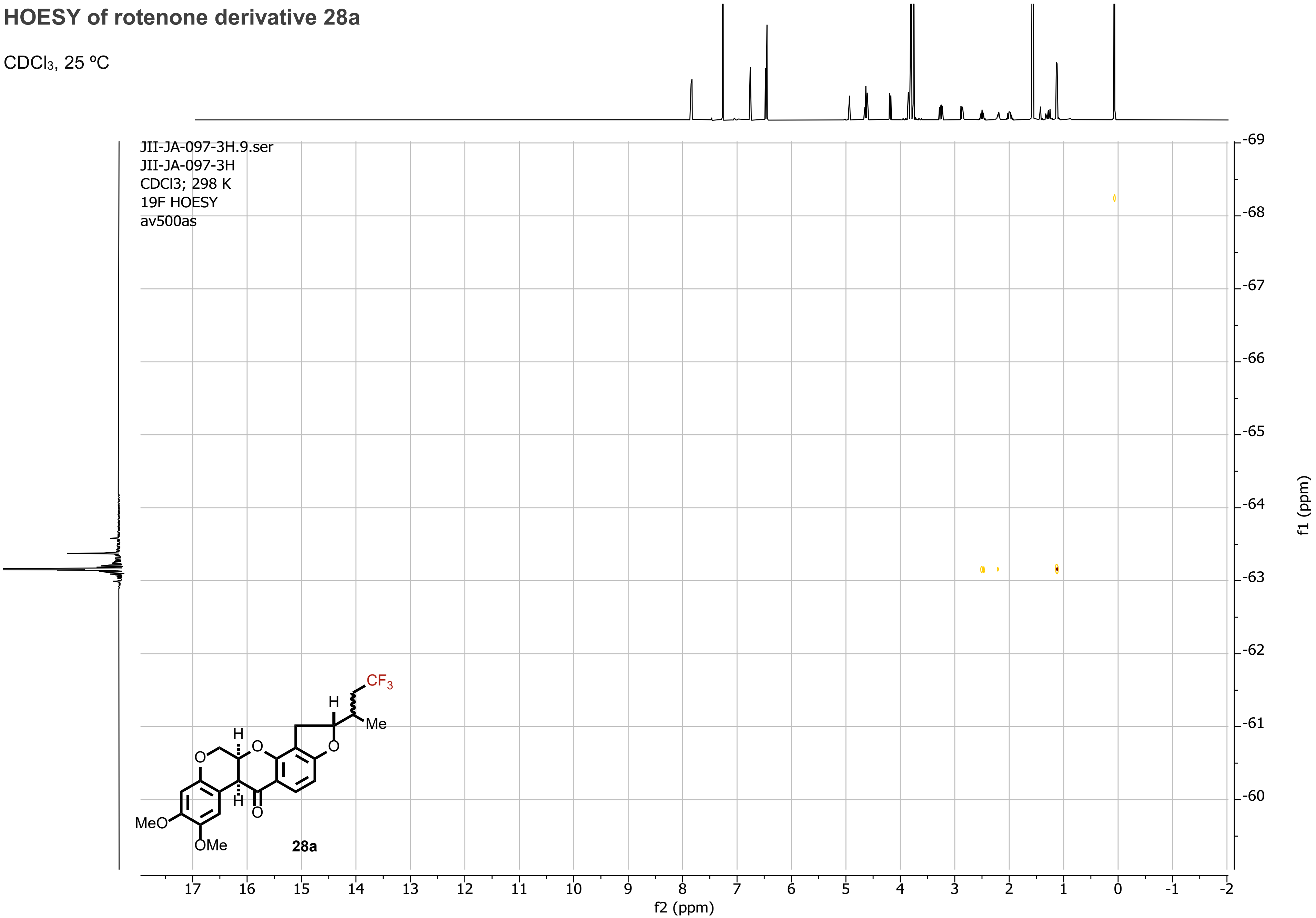

^{1}H NMR of rotenone derivative 28b

CDCl$_3$, 25 ºC

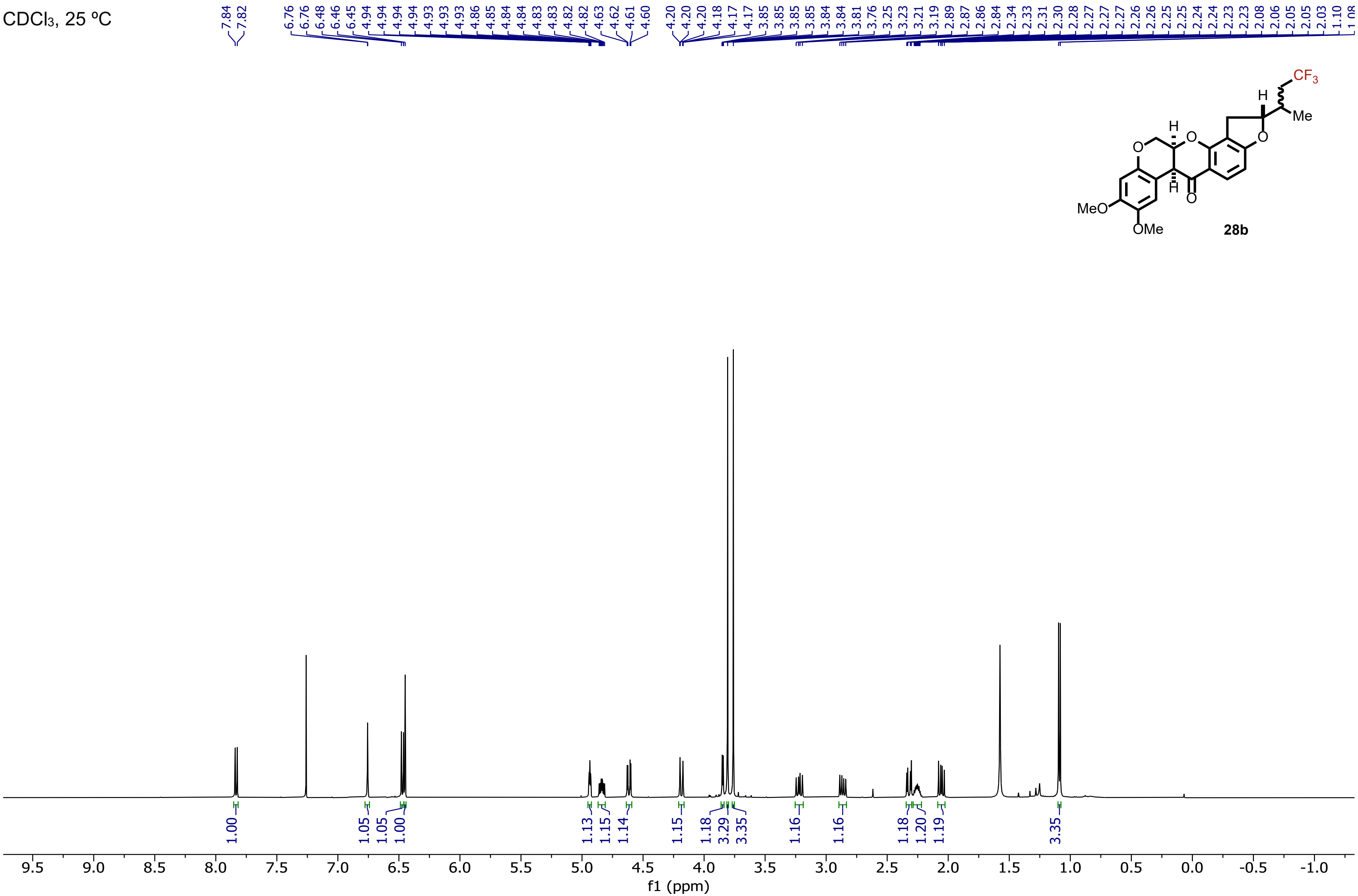

^{19}F NMR of rotenone derivative 28b

$CDCl_3$, 25 ºC

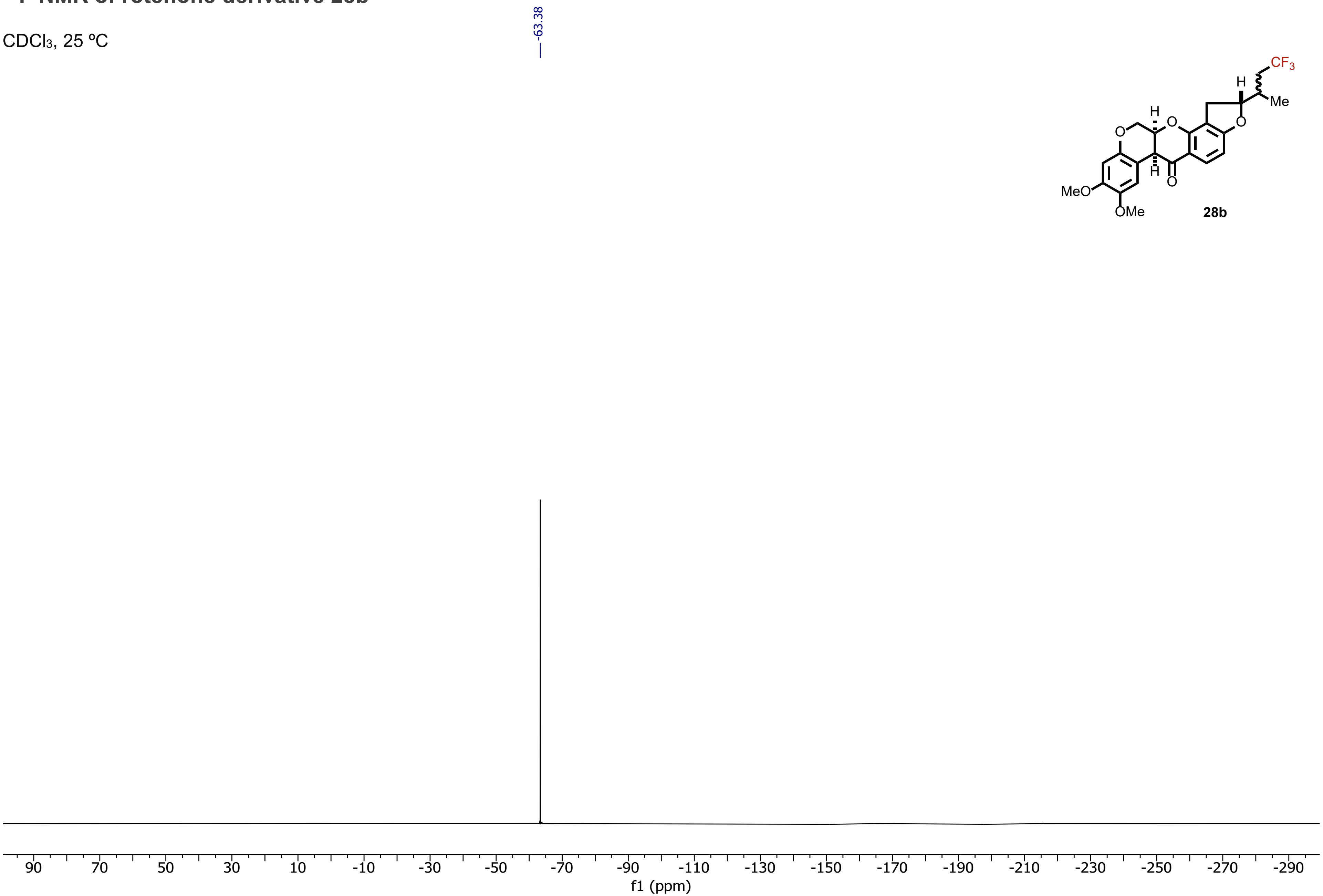

28b

^{13}C NMR of rotenone derivative 28b

CDCl₃, 25 ºC

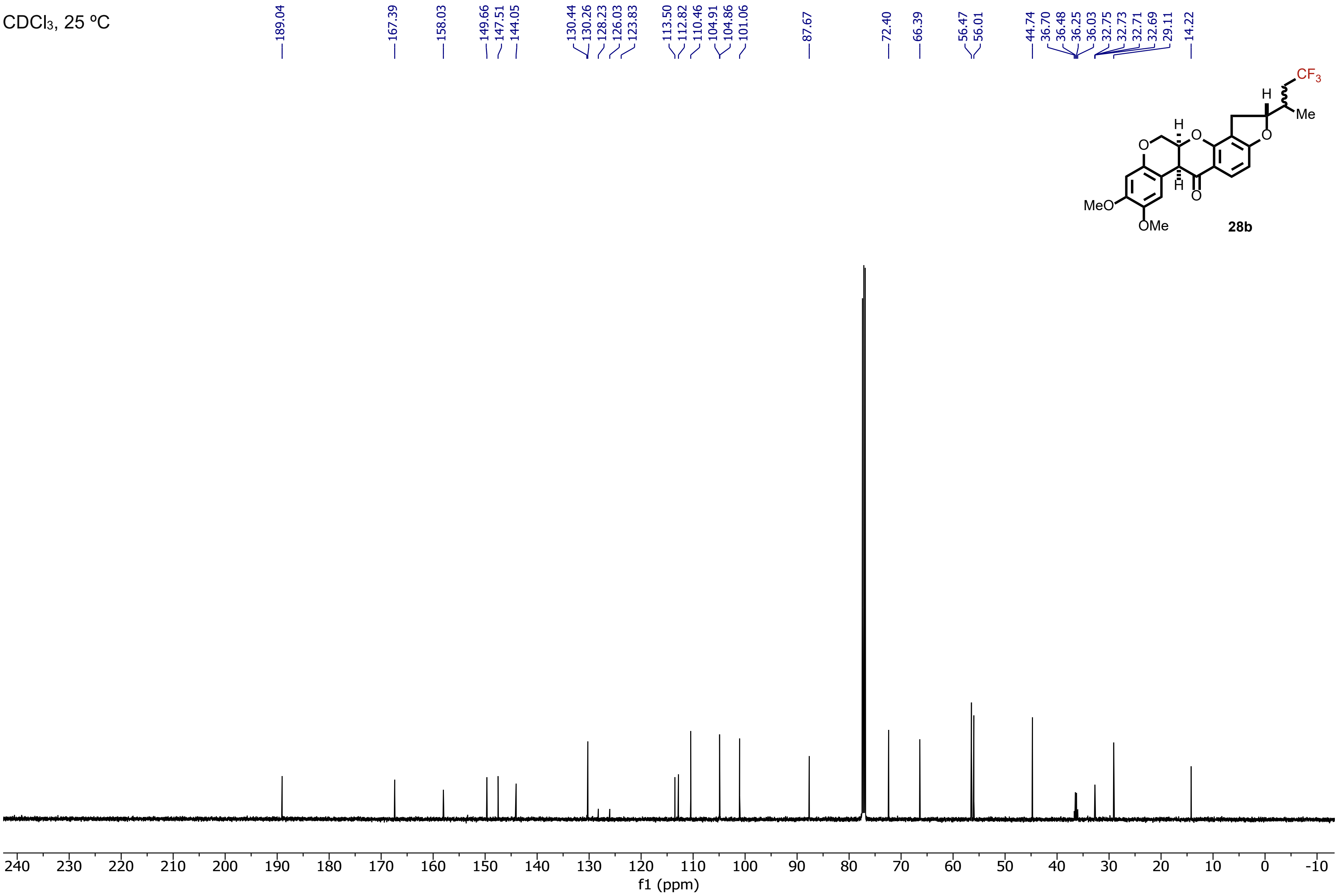

HSQC of rotenone derivative 28b

CDCl3, 25 ºC

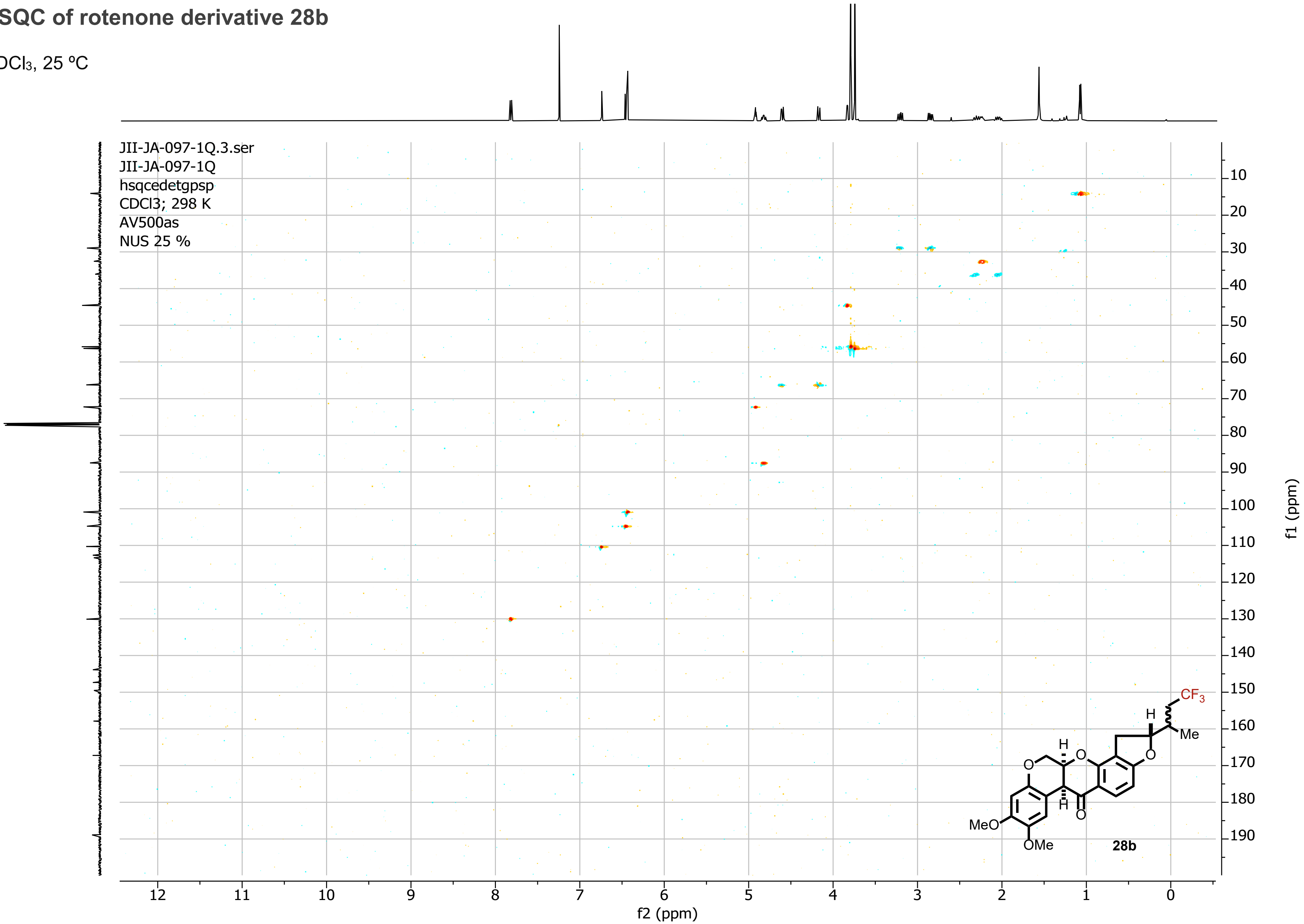

HMQC of rotenone derivative 28b

CDCl3, 25 ºC

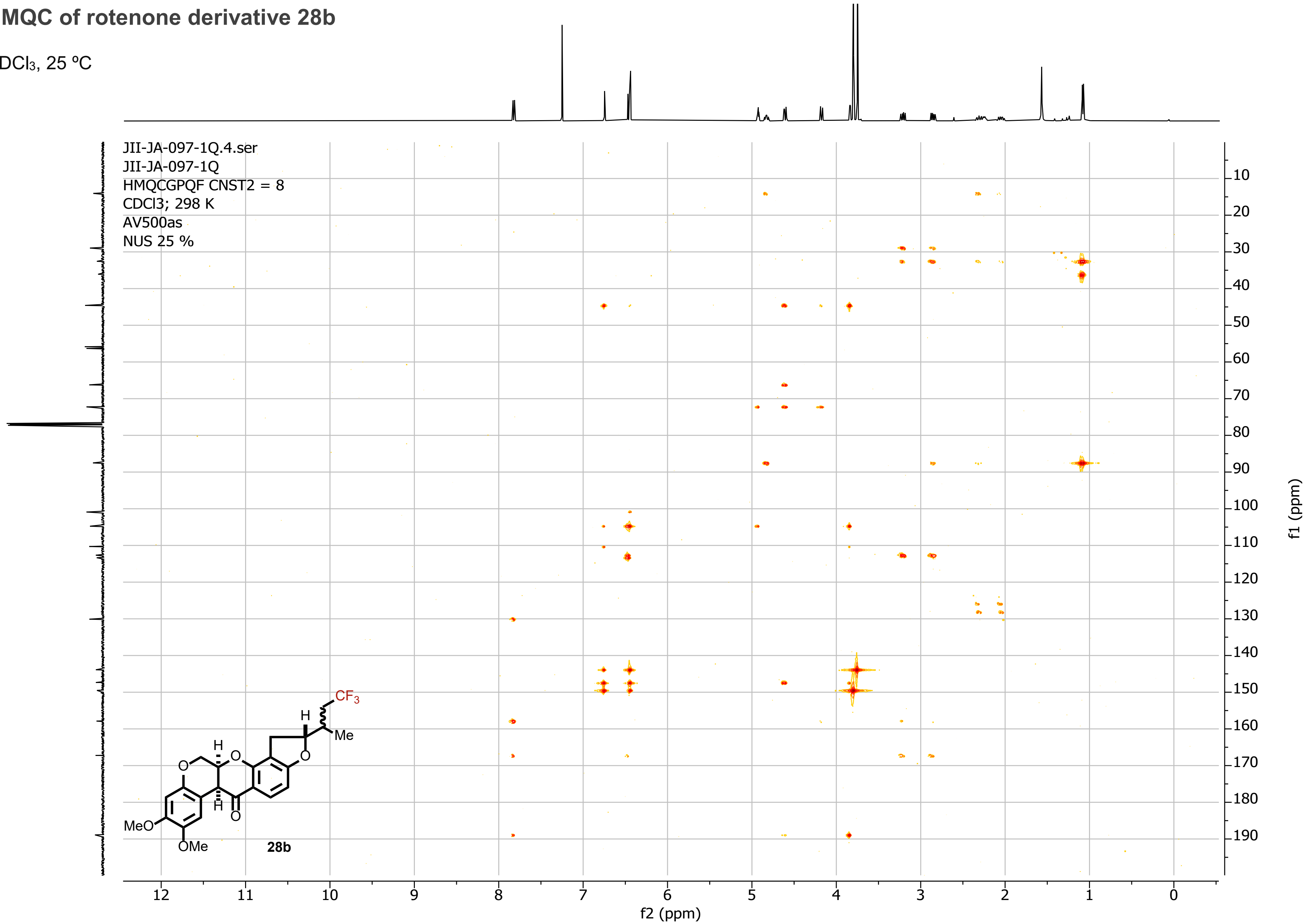

COSY of rotenone derivative 28b

CDCl3, 25 ºC

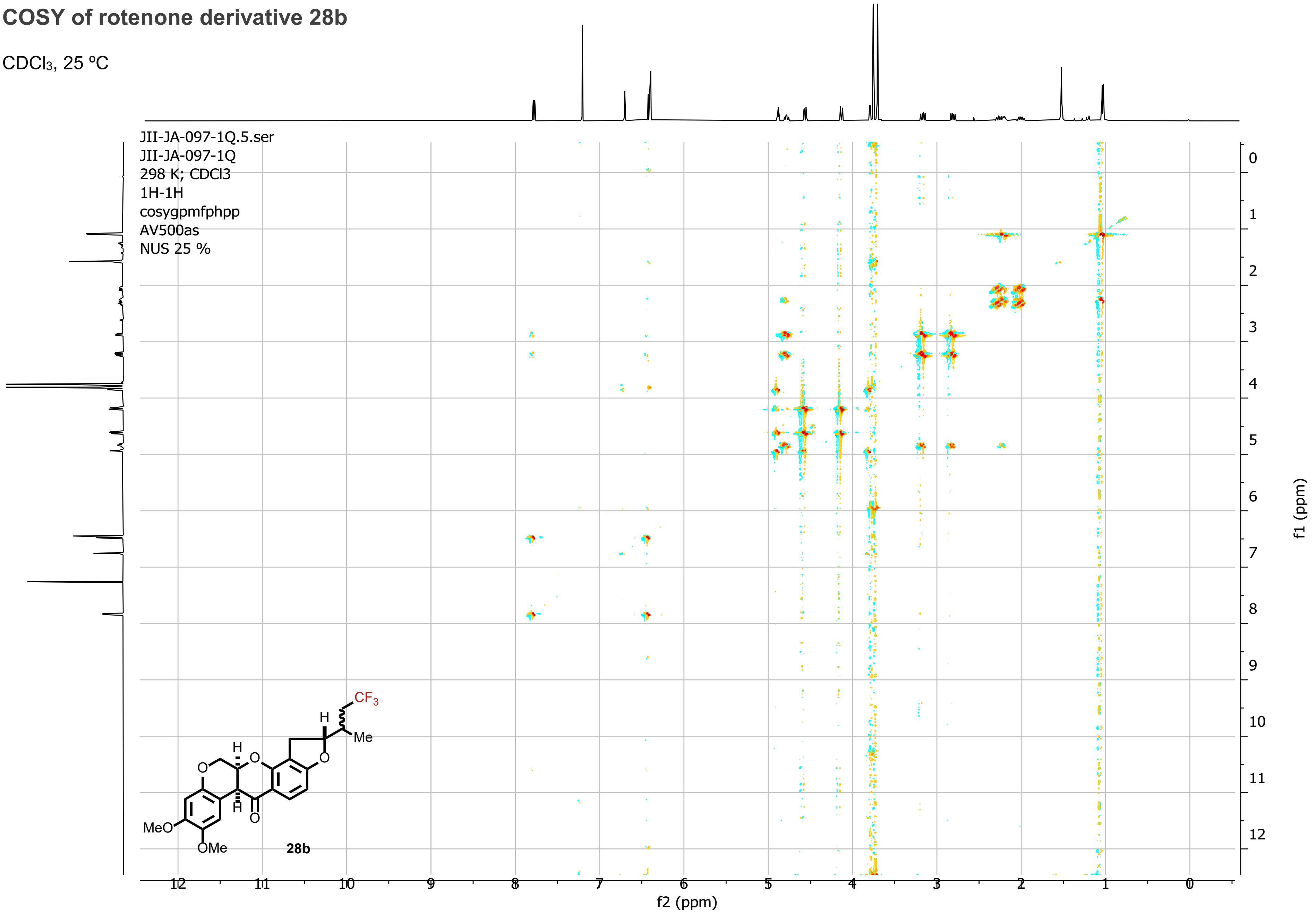

NOESY of rotenone derivative 28b

CDCl₃, 25 ºC

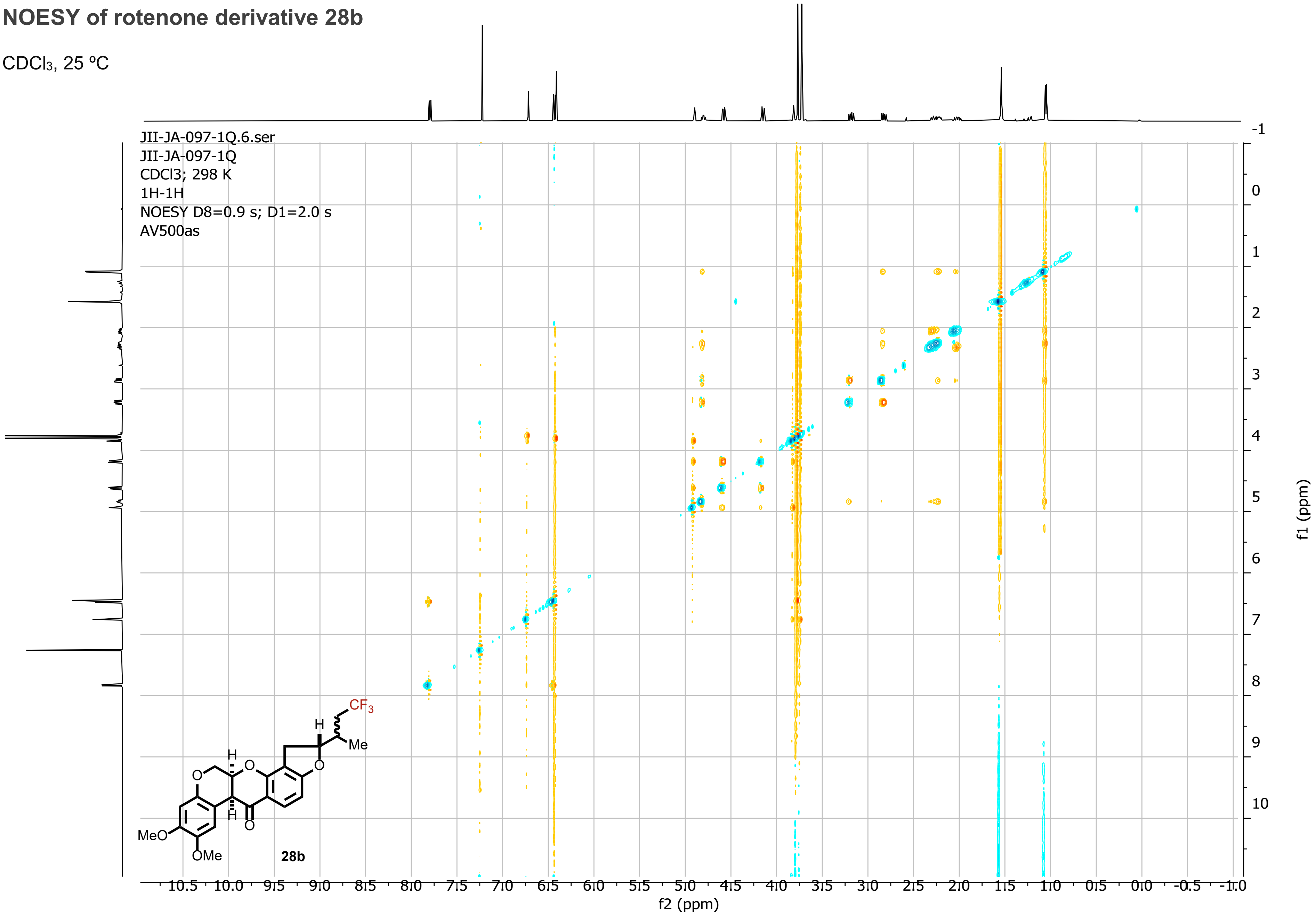

HOESY of rotenone derivative 28b

CDCl₃, 25 ºC

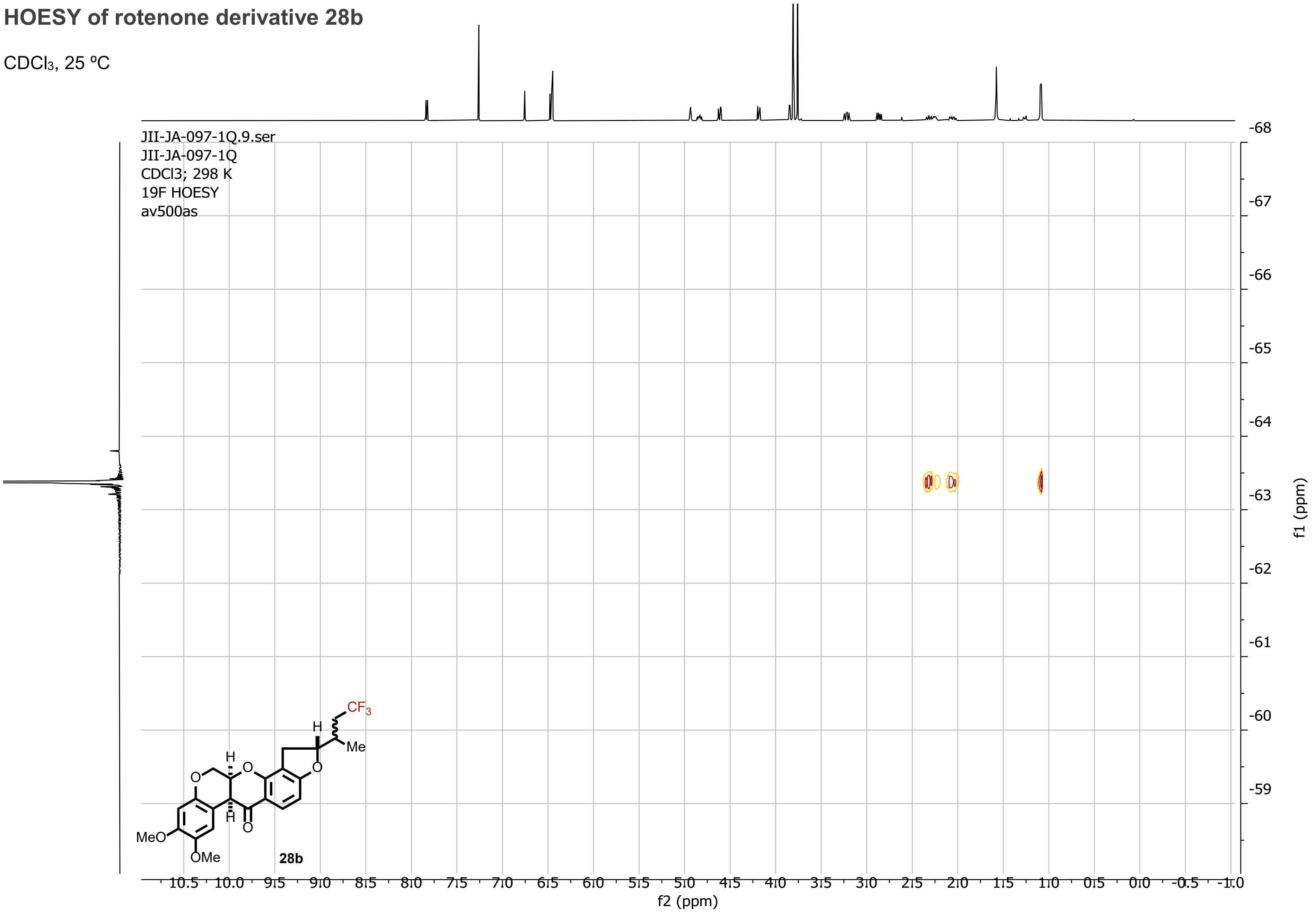

^{1}H NMR of epiandrosterone derivative 29

CDCl3, 25 ºC

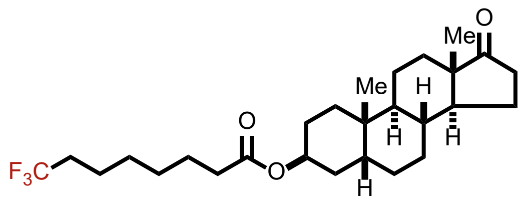

29

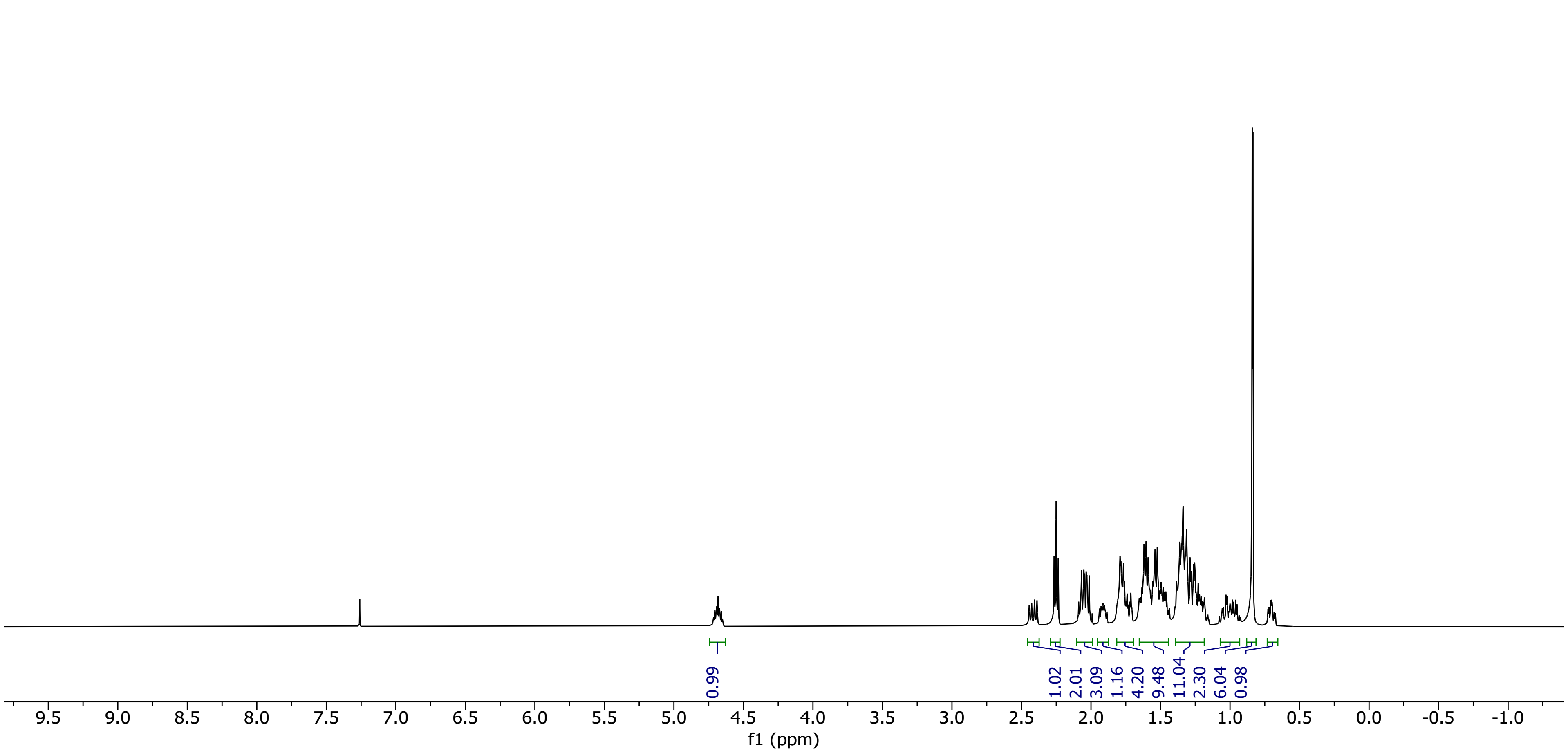

^{19}F NMR of epiandrosterone derivative 29

CDCl3, 25 ºC

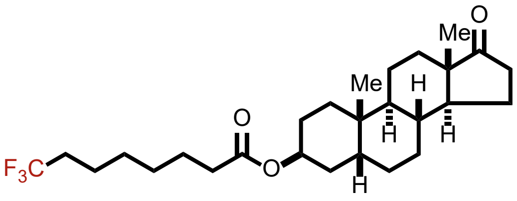

29

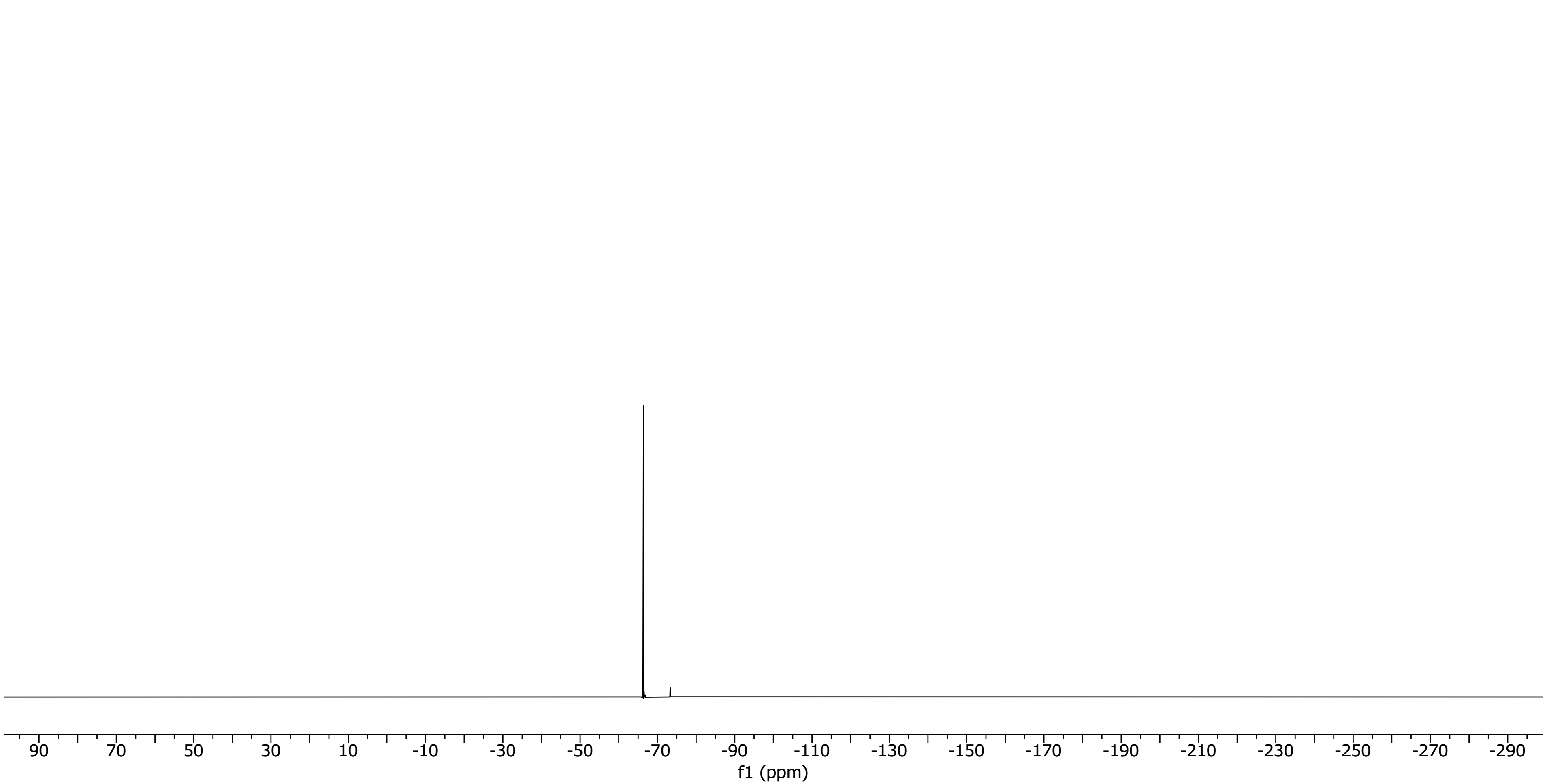

^{13}C NMR of epiandrosterone derivative 29

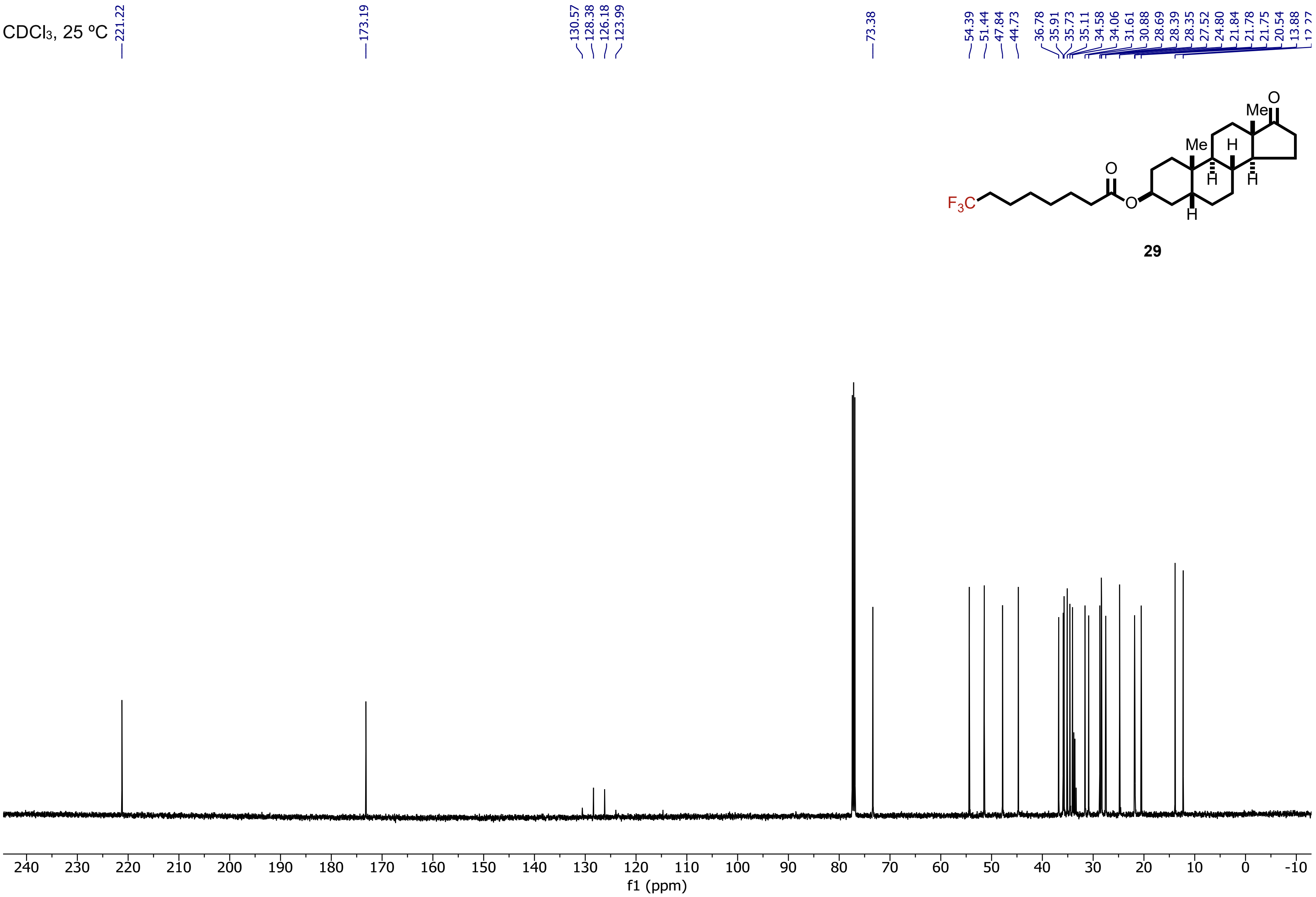

^{1}H NMR of D-glucose derivative 30

CDCl$_3$, 25 ºC

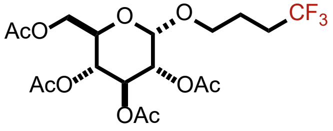

30

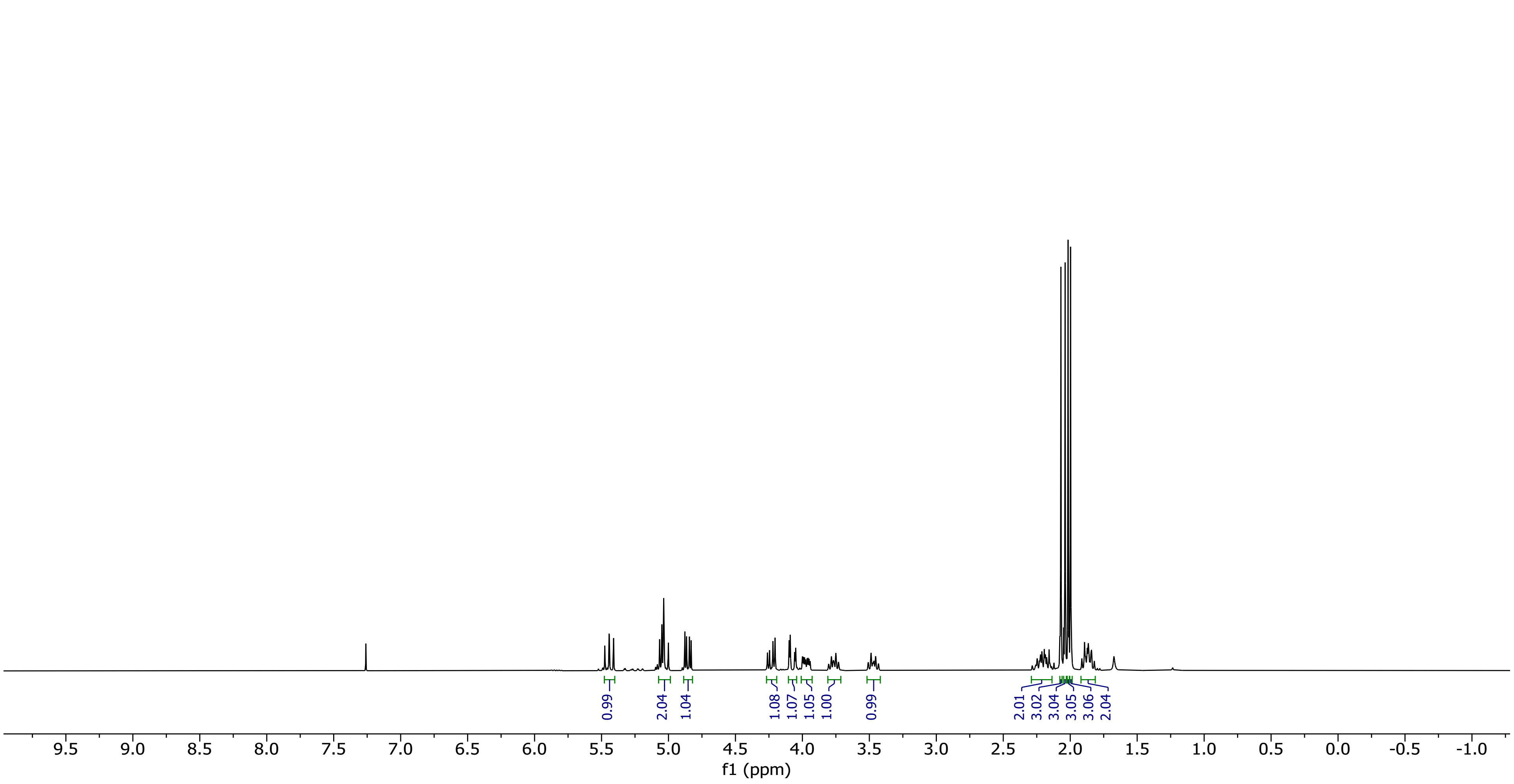

^{19}F NMR of D-glucose derivative 30

CDCl3, 25 ºC

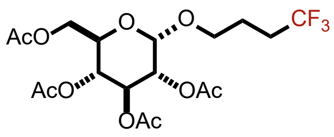

30

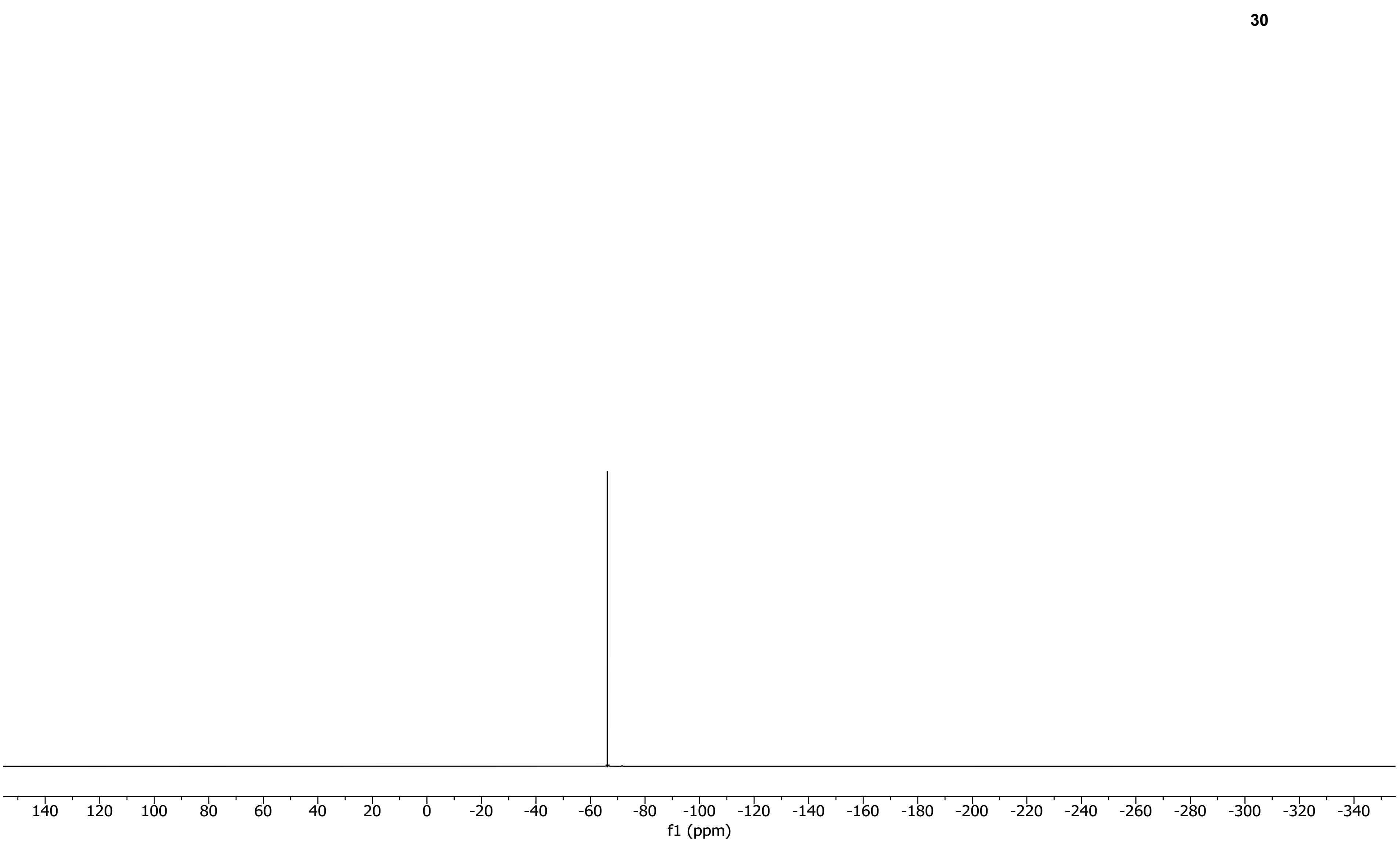

^{13}C NMR of D-glucose derivative 30

CDCl3, 25 ºC

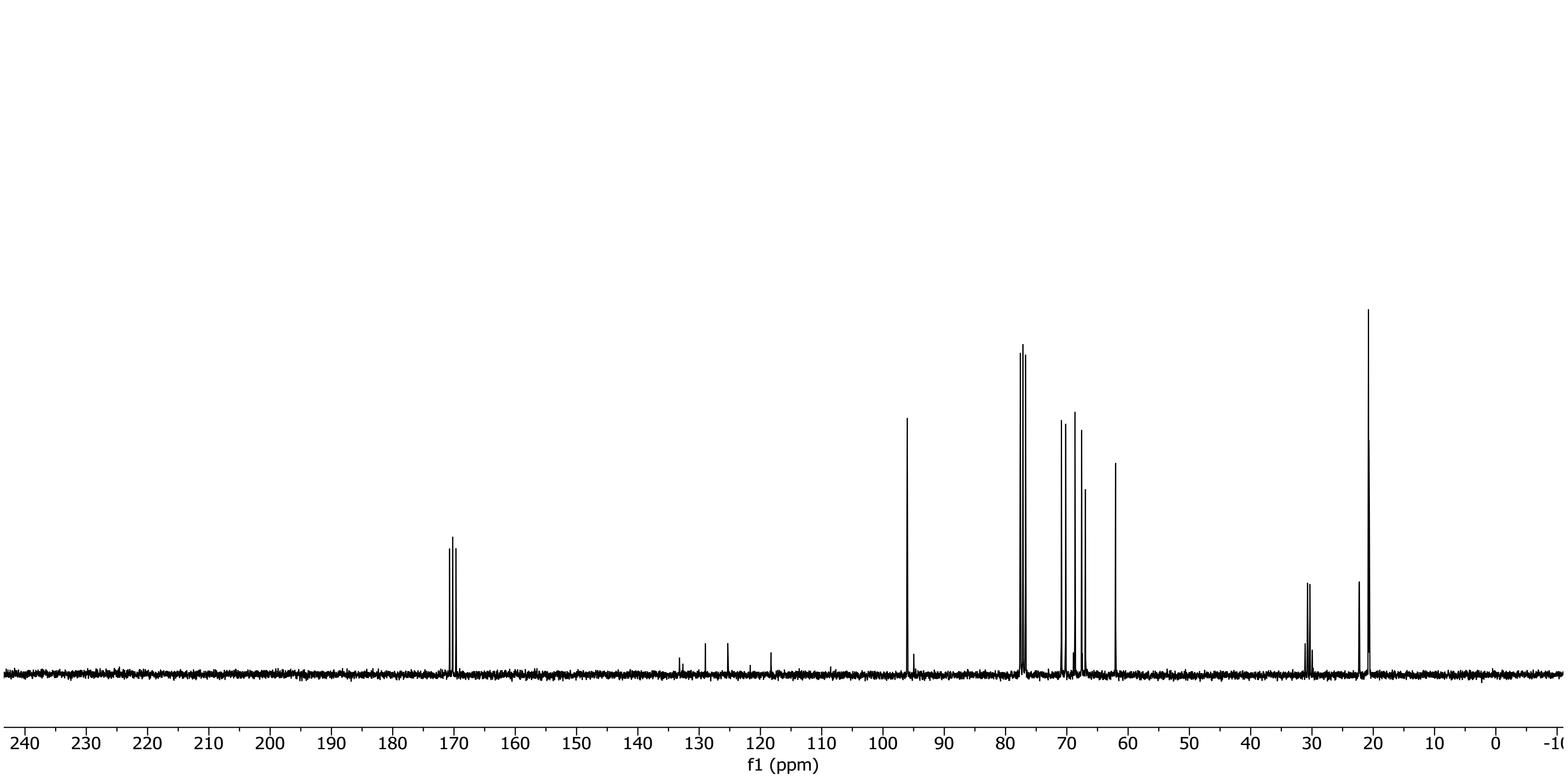

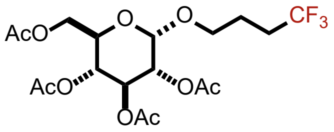

30

^{1}H NMR of quinine derivative 31

CD$_3$CN, 25 ºC

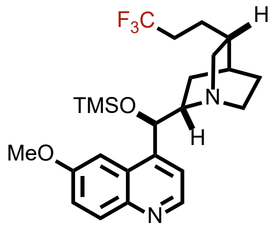

31

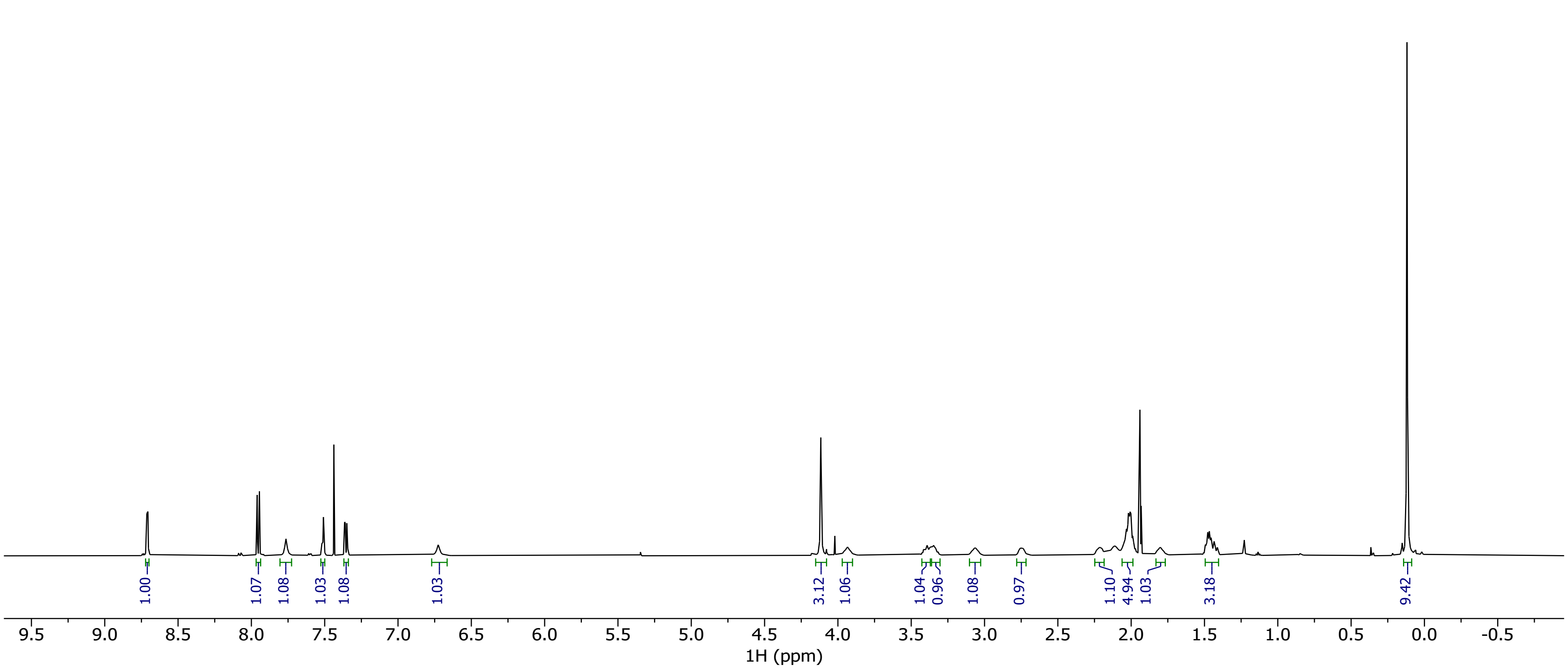

^{19}F NMR of quinine derivative 31

CD_3CN, 25 ºC

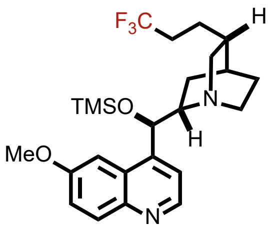

31

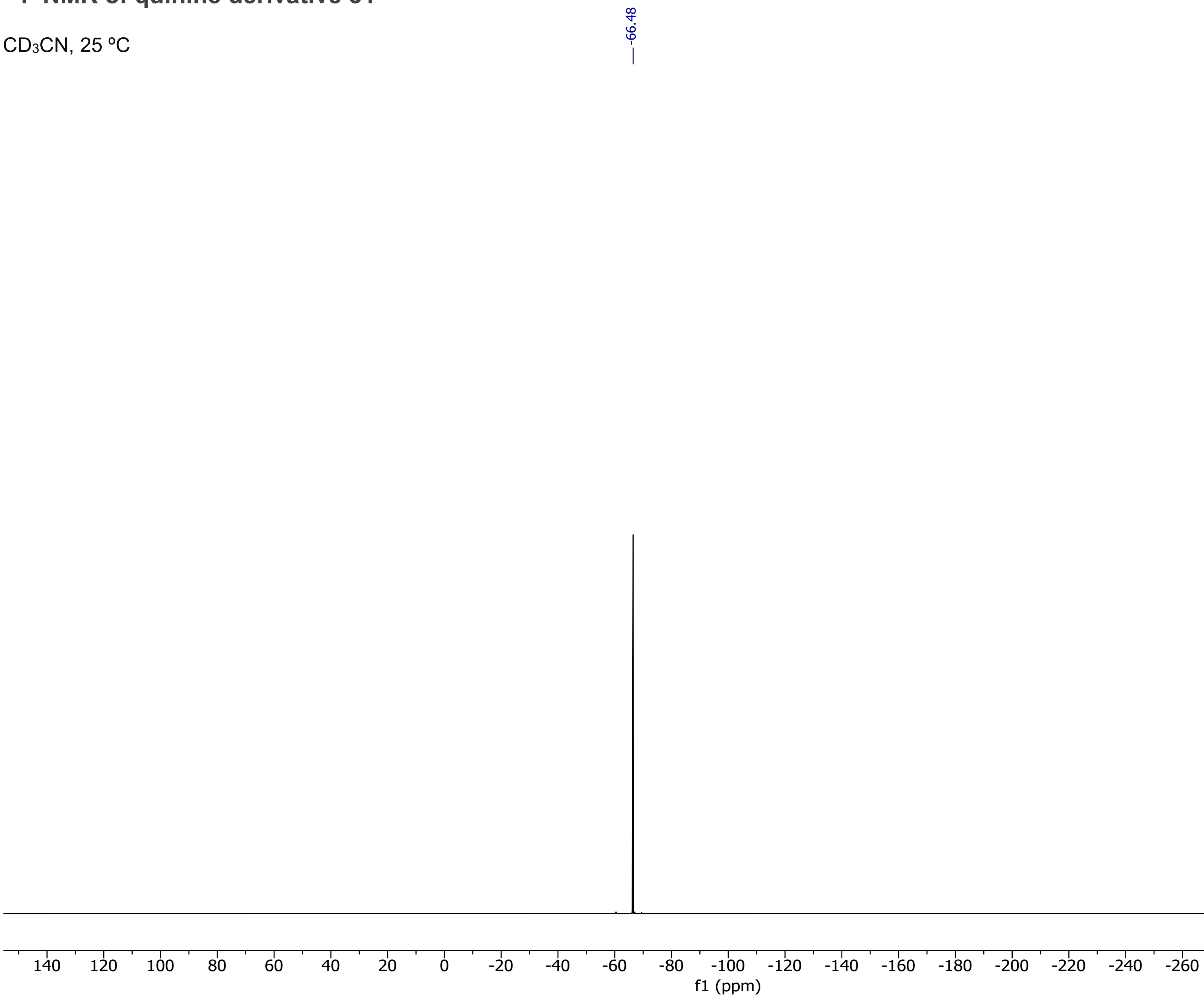

^{13}C NMR of quinine derivative 31

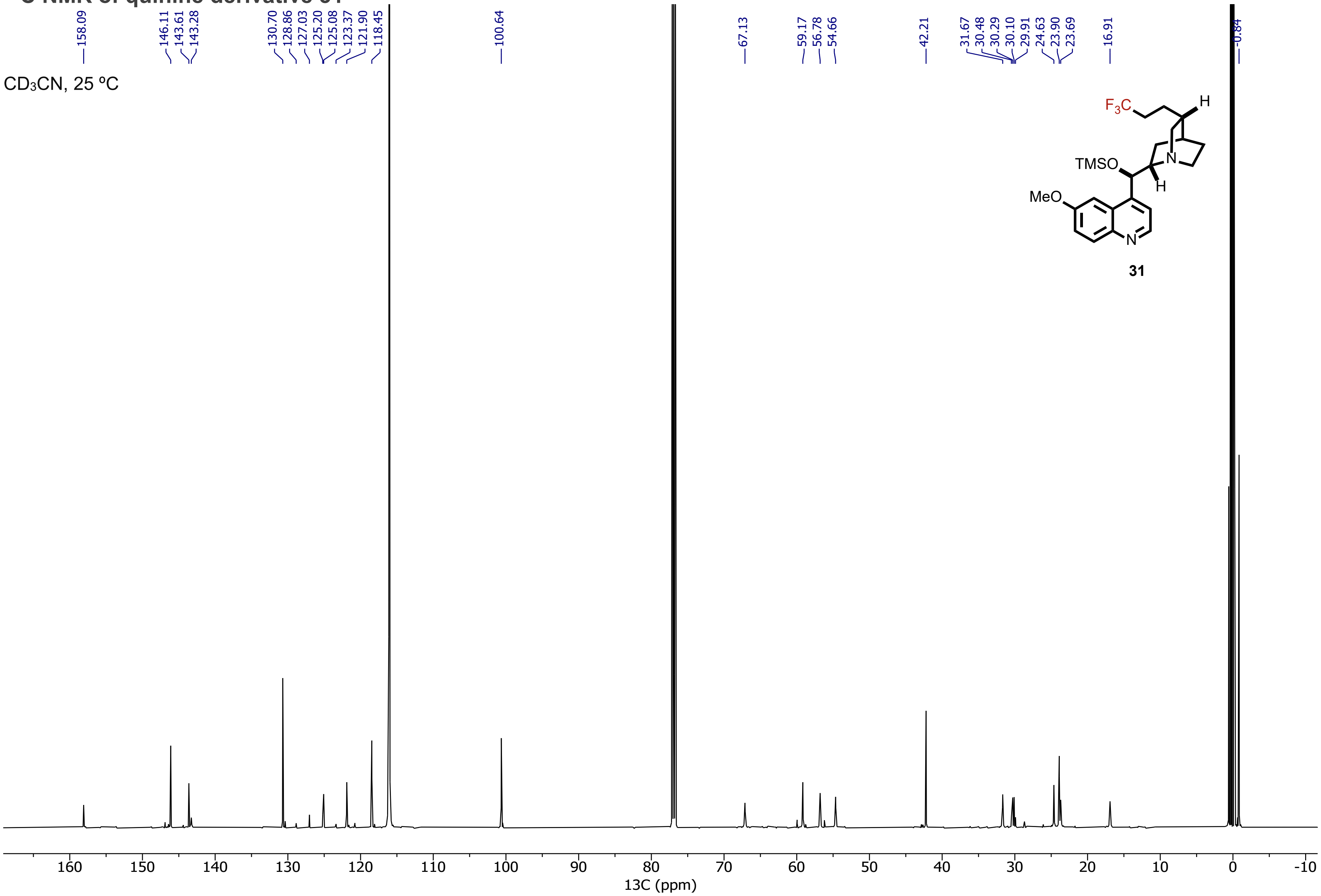

31

^{1}H NMR of adapalene derivative 32

CDCl$_3$, 25 ºC

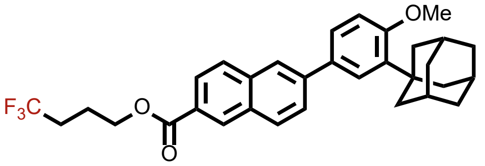

32

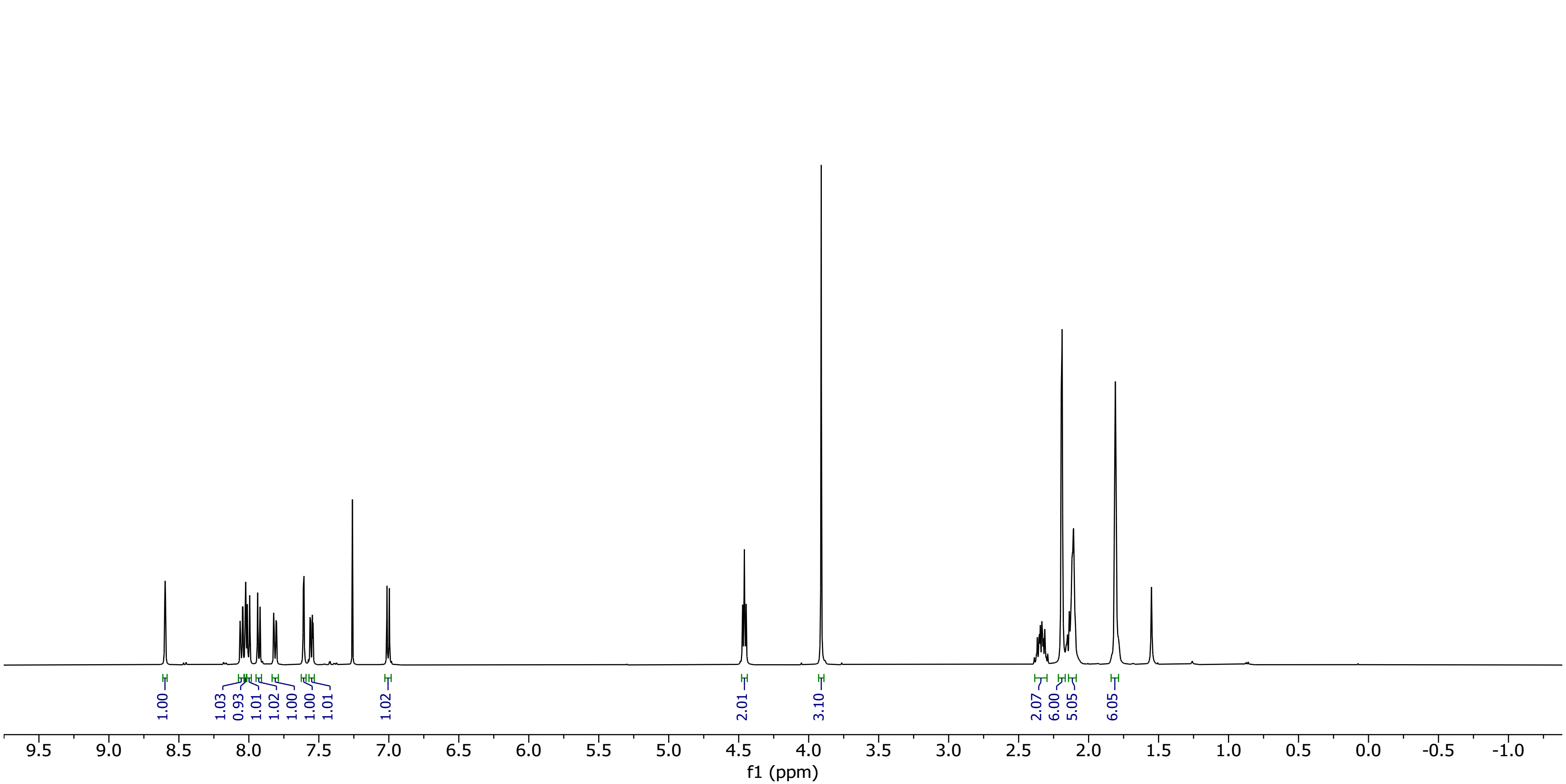

^{19}F NMR of adapalene derivative 32

$CDCl_3$, 25 ºC

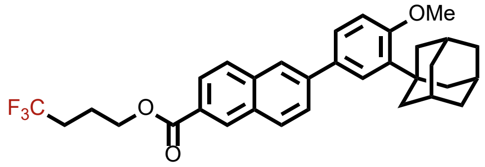

32

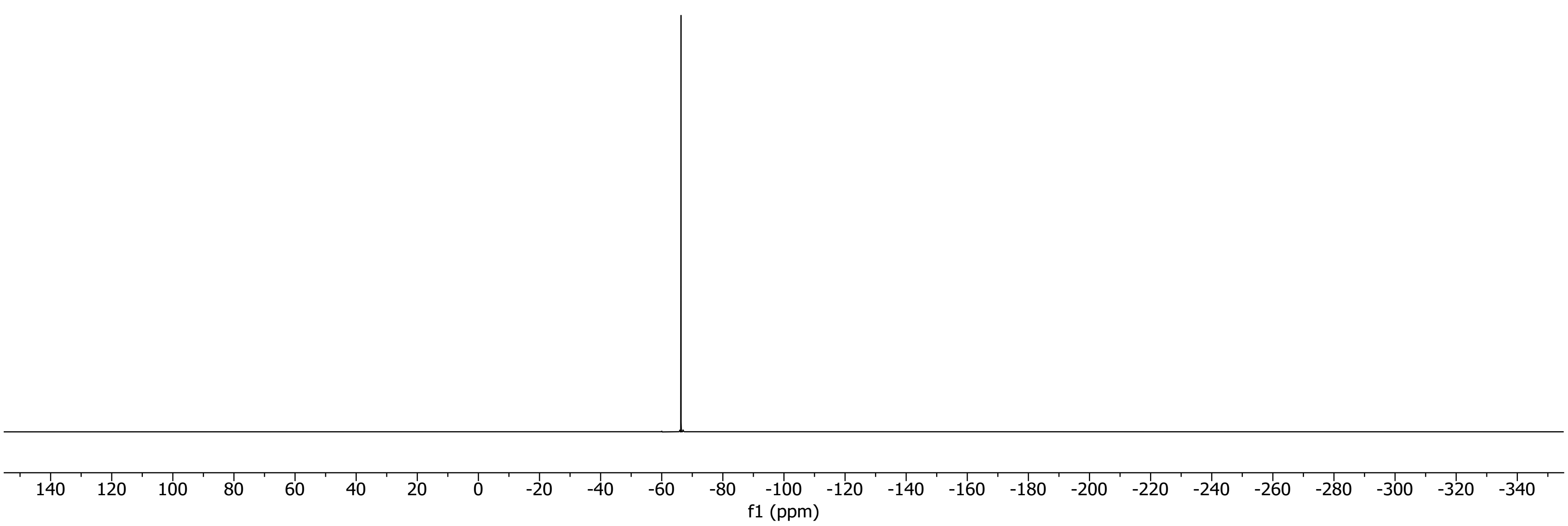

^{13}C NMR of adapalene derivative 32

$CDCl_3$, 25 ºC

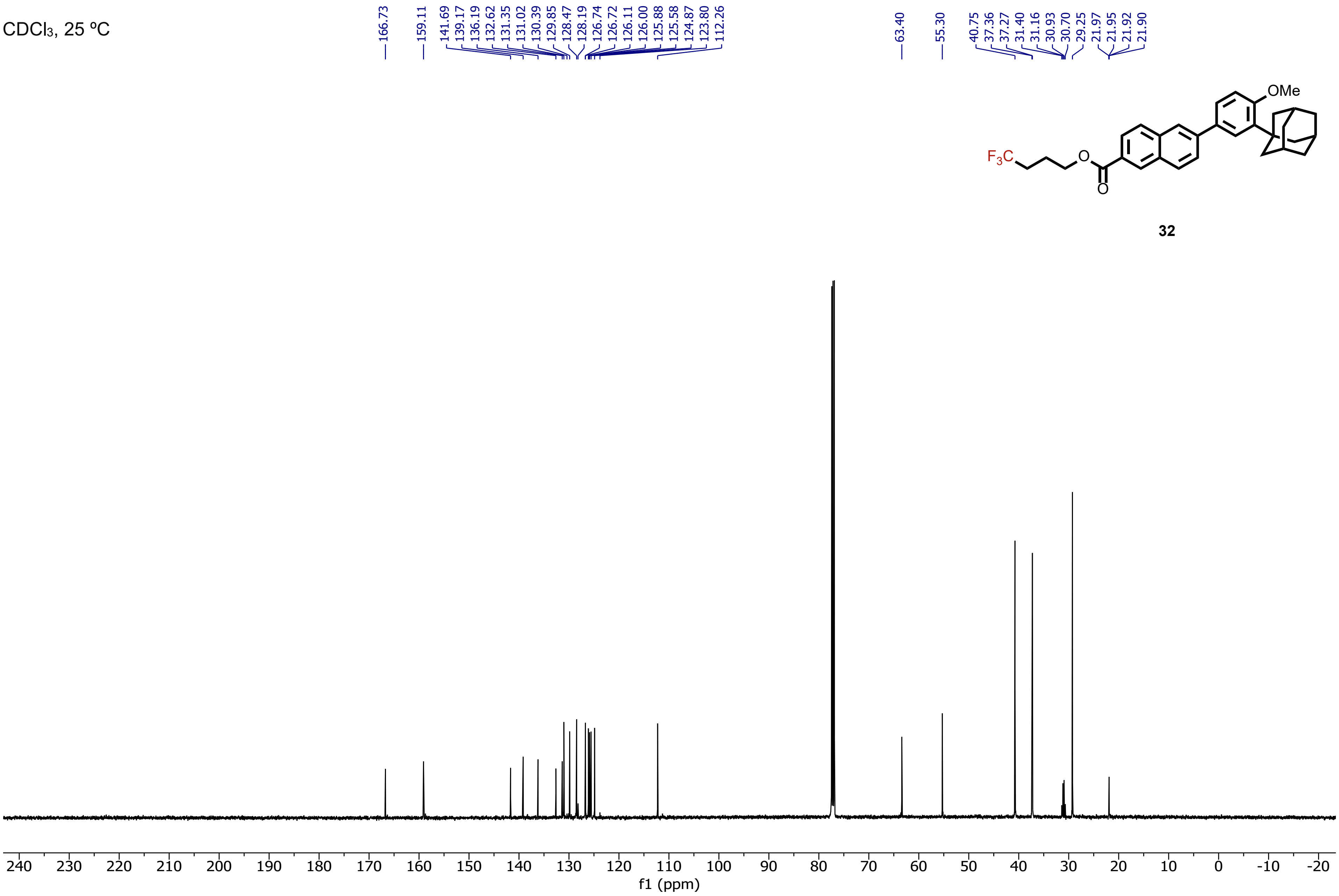

32

^{1}H NMR of lithocholic acid derivative 33

CDCl$_3$, 25 ºC

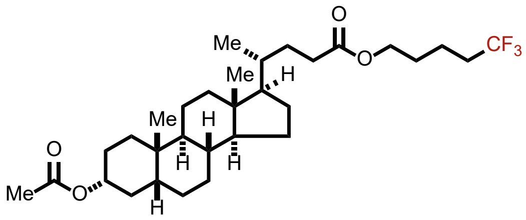

33

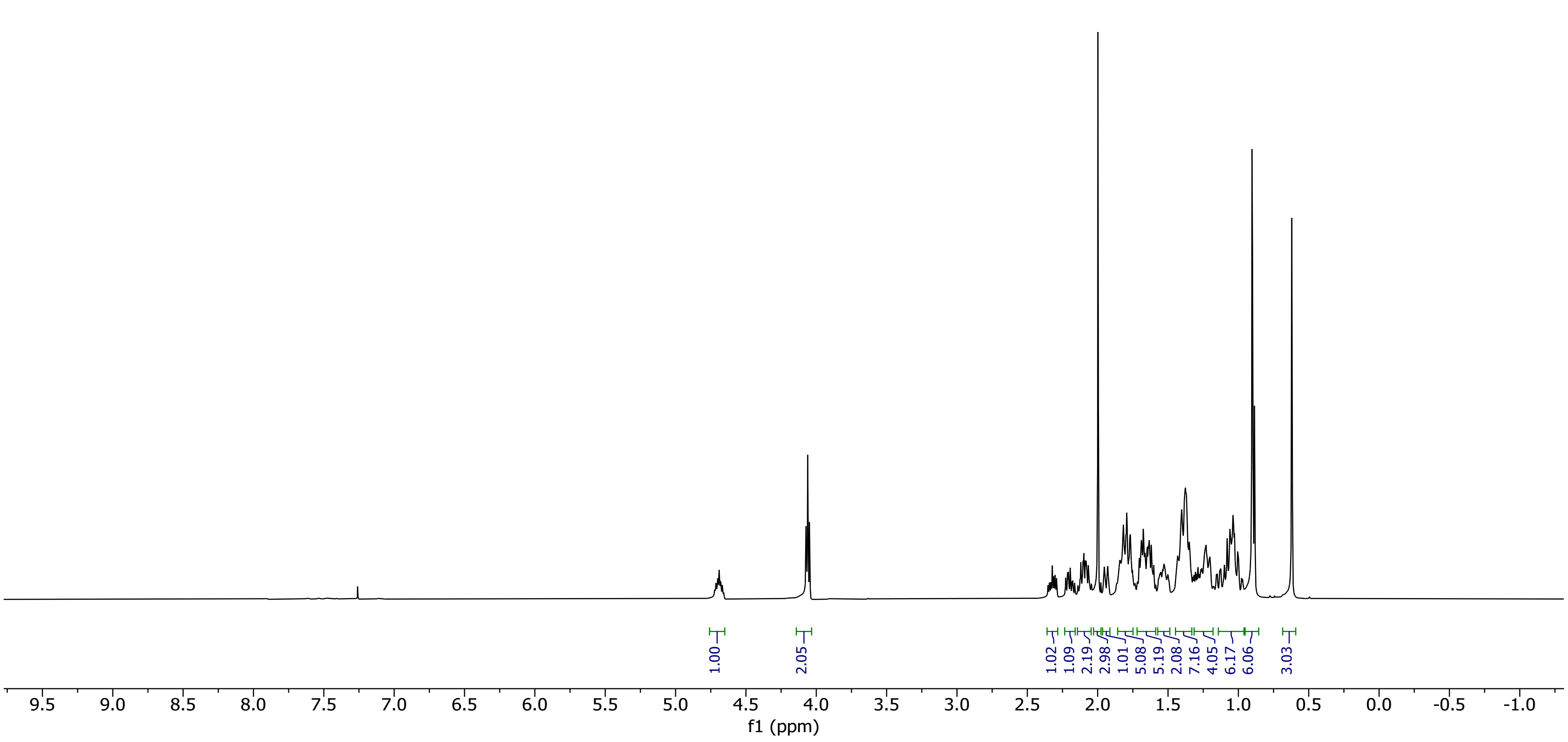

^{19}F NMR of lithocholic acid derivative 33

$CDCl_3$, 25 ºC

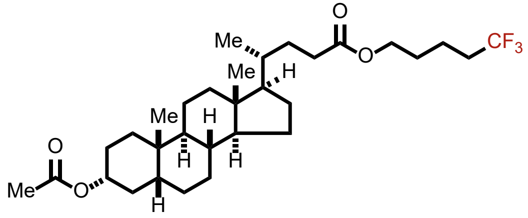

33

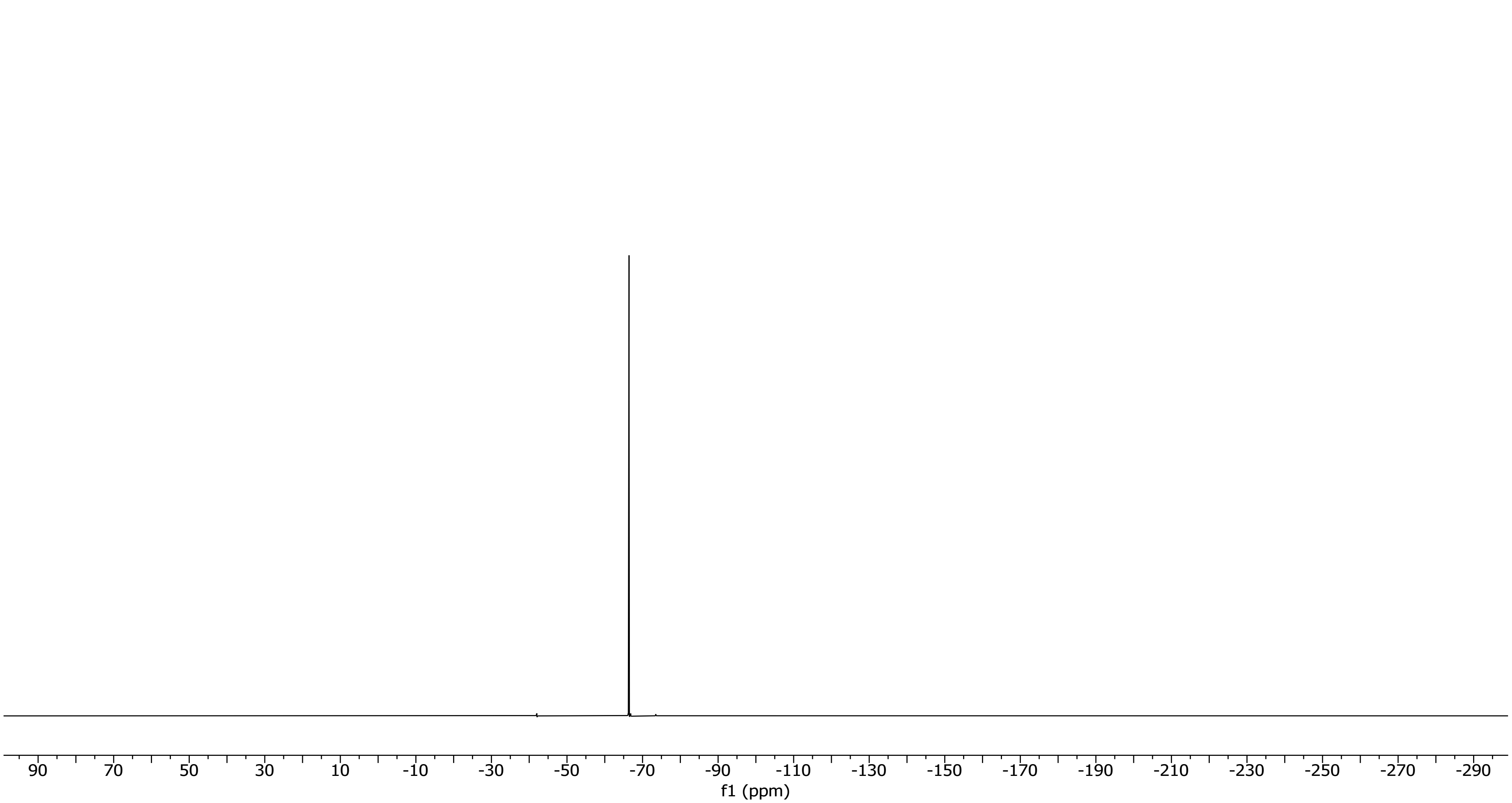

^{13}C NMR of lithocholic acid derivative 33

CDCl$_3$, 25 ºC

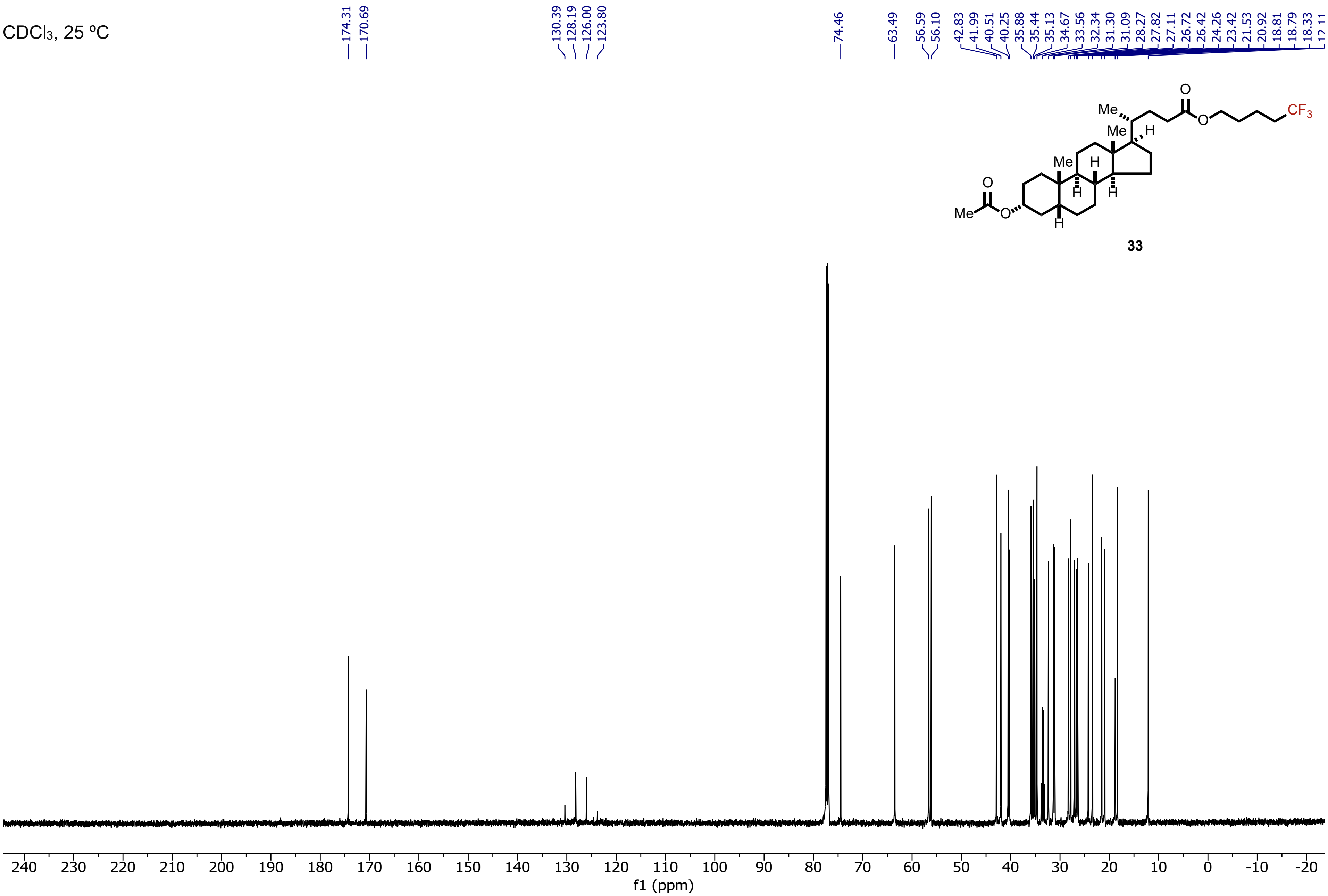

^{1}H NMR of diethyl 3-methyl-4-(2,2,2-trifluoroethyl)cyclopentane-1,1-dicarboxylate (34)

CDCl3, 25 ºC

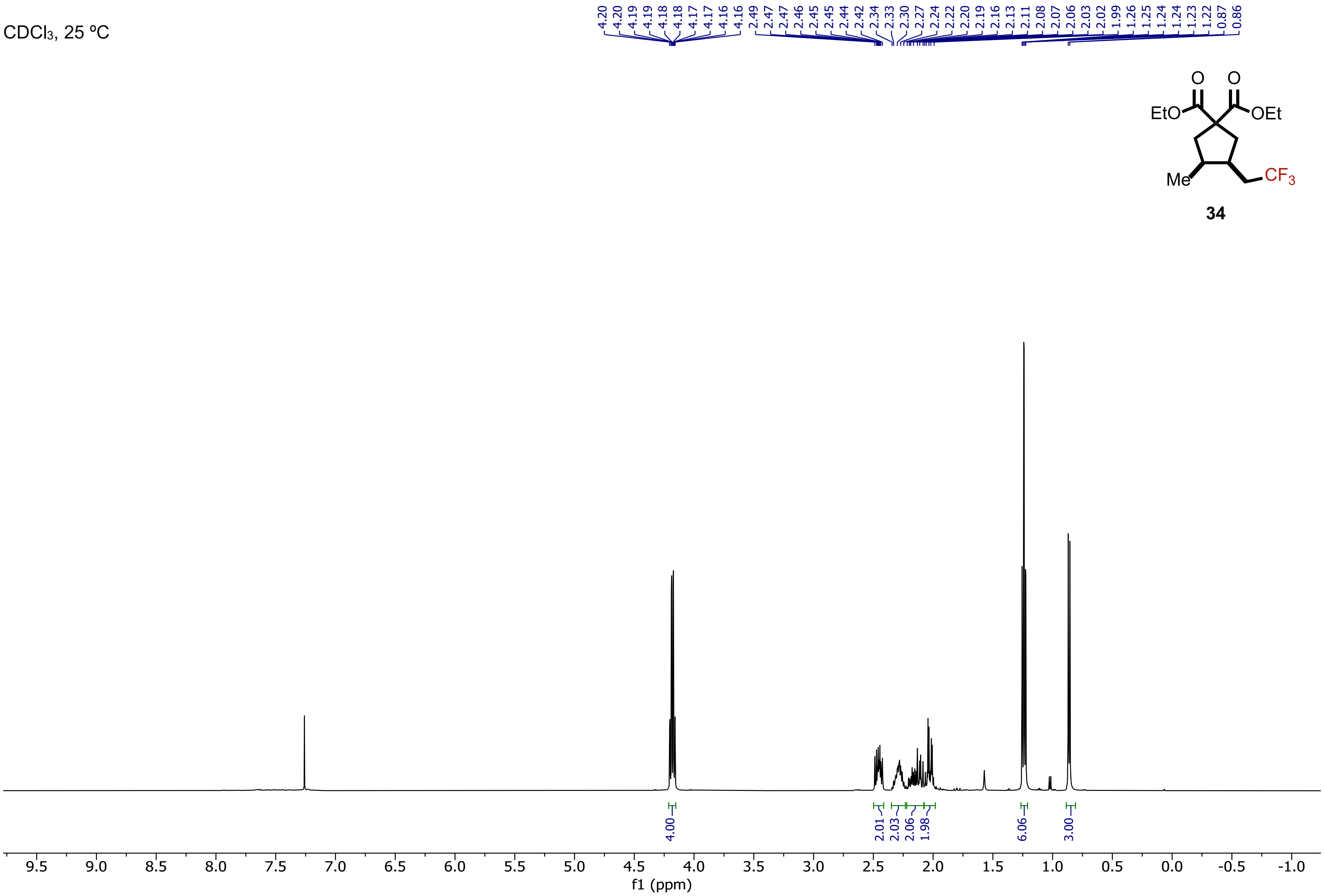

34

^{19}F NMR of diethyl 3-methyl-4-(2,2,2-trifluoroethyl)cyclopentane-1,1-dicarboxylate (34)

$CDCl_3$, 25 ºC

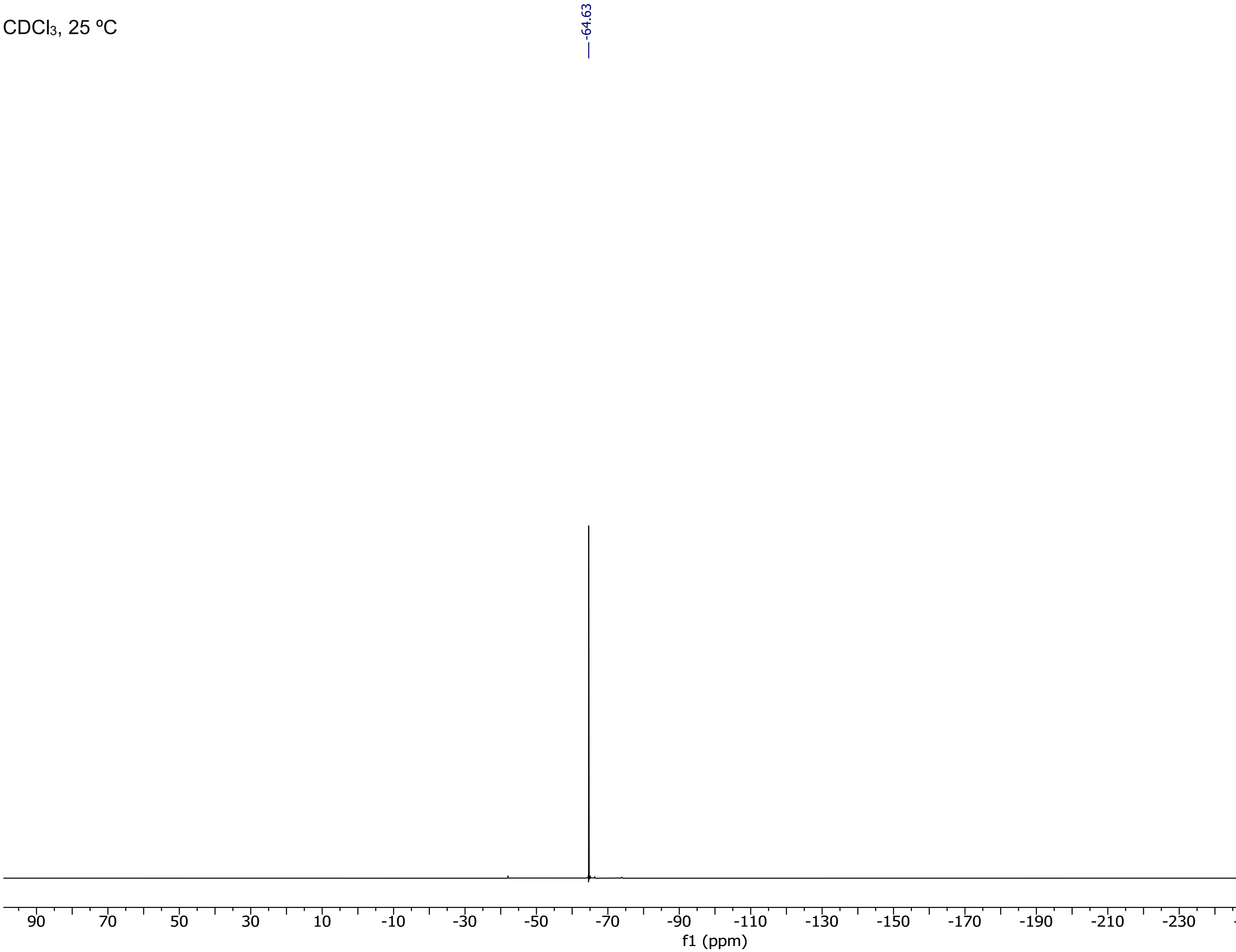

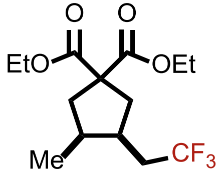

34

^{13}C NMR of diethyl 3-methyl-4-(2,2,2-trifluoroethyl)cyclopentane-1,1-dicarboxylate (34)

$CDCl_3$, 25 ºC

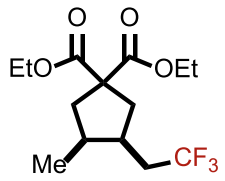

34

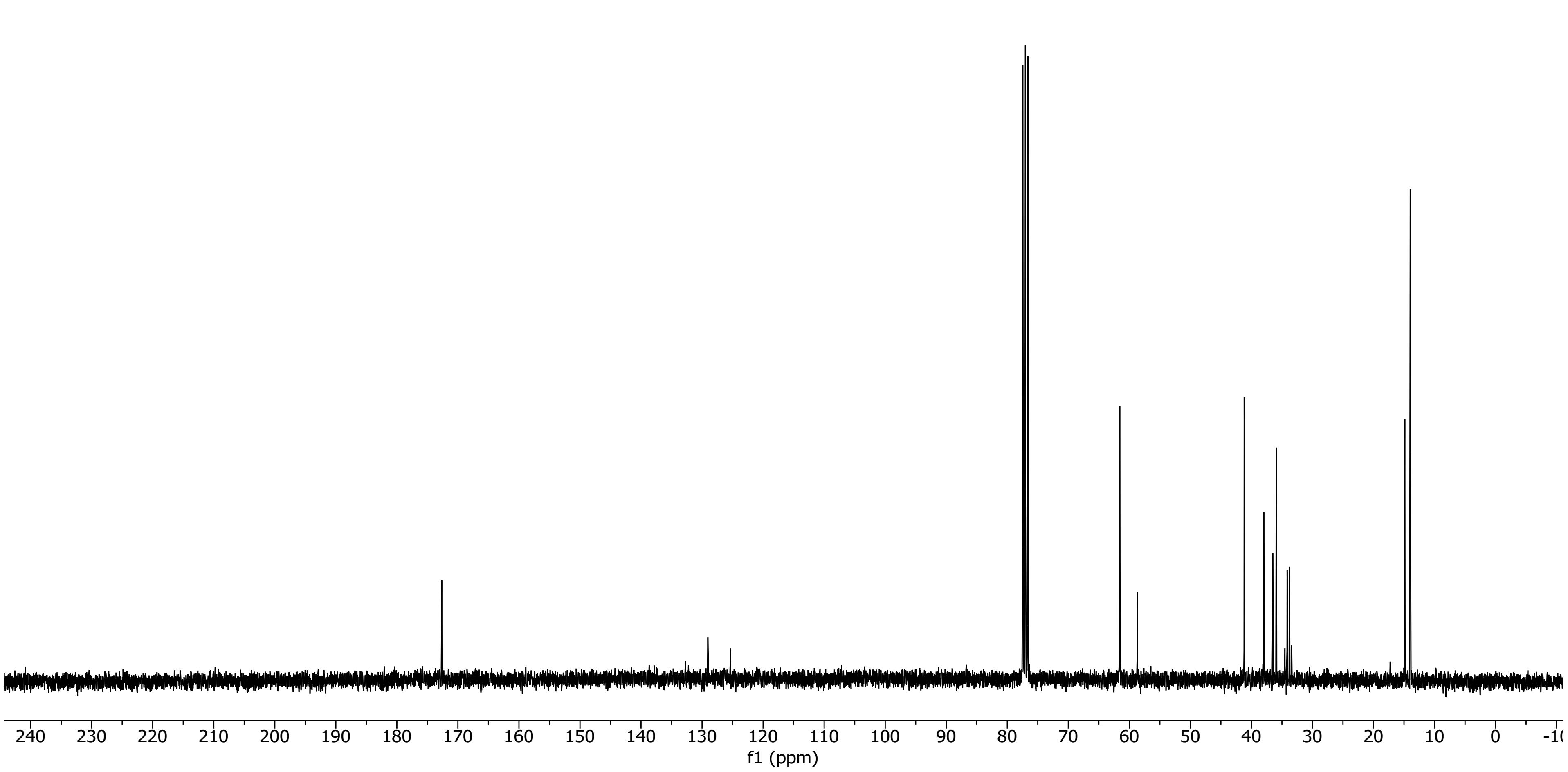

REFERENCES

1. Fulmer, G. R.; Miller, A. J. M.; Sherden, N. H.; Gottlieb, H. E.; Nudelman, A.; Stoltz, B. M.; Bercaw, J. E.; Goldberg, K. I. NMR Chemical Shifts of Trace Impurities: Common Laboratory Solvents, Organics, and Gases in Deuterated Solvents Relevant to the Organometallic Chemist. *Organometallics* **2010**, *29*, 2176-2179.
2. Harris, R. K.; Becker, E. D.; Cabral de Menezes, S. M.; Goodfellow, R.; Granger, P. NMR Nomenclature: Nuclear Spin Properties and Conventions for Chemical Shifts. IUPAC Recommendations 2001. *Solid State Nucl. Magn. Reson.* **2002**, *22*, 458-483.
3. Chen, J.; Li, J.; Plutschack, M. B.; Berger, F.; Ritter, T. Regio- and Stereoselective Thianthrenation of Olefins to Access Versatile Alkenyl Electrophiles. *Angew. Chem. Int. Ed.* **2020,** *59*, 5616-5620.
4. Piccirillo, G.; Pepe, A.; Bedini, E.; Bochicchio, B. Photoinduced Thiol-ene Chemistry Applied to the Synthesis of Self-Assembling Elastin-Inspired Glycopeptides. *Chem. Eur. J.* **2017,** *23*, 2648-2659.
5. Mir, R.; Dudding, T. Phase-Transfer Catalyzed O-Silyl Ether Deprotection Mediated by a Cyclopropenium Cation. *J. Org. Chem.* **2017,** *82*, 709-714.
6. Dittrich, B.; Hübschle, C. B.; Pröpper, K.; Dietrich, F.; Stolper, T.; Holstein, J. J. The Generalized Invariom Database (GID). *Acta Crystallogr., Sect. B: Struct. Sci., Cryst. Eng. Mater.* **2013**, *69*, 91-104.
7. Mizuta, S.; Verhoog, S.; Engle, K. M.; Khotavivattana, T.; O'Duill, M.; Wheelhouse, K.; Rassias, G.; Médebielle, M.; Gouverneur, V. Catalytic Hydrotrifluoromethylation of Unactivated Alkenes. *J. Am. Chem. Soc.* **2013,** *135*, 2505-2508.
8. Umemoto, T.; Zhang, B.; Zhu, T.; Zhou, X.; Zhang, P.; Hu, S.; Li, Y. Powerful, Thermally Stable, One-Pot-Preparable, and Recyclable Electrophilic Trifluoromethylating Agents: 2,8-Difluoro- and 2,3,7,8-Tetrafluoro-*S*-(trifluoromethyl)dibenzothio-phenium Salts. *J. Org. Chem.* **2017,** *82*, 7708-7719.
9. Wu, L.-H.; Zhao, K.; Shen, Z.-L.; Loh, T.-P. Copper-catalyzed Trifluoromethylation of Styrene Derivatives with CF_3SO_2Na. *Org. Chem. Front.* **2017,** *4*, 1872-1875.
10. Li, J.; Chen, J.; Sang, R.; Ham, W.-S.; Plutschack, M. B.; Berger, F.; Chabbra, S.; Schnegg, A.; Genicot, C.; Ritter, T. Photoredox Catalysis with Aryl Sulfonium Salts Enables Site-selective Late-stage Fluorination. *Nat. Chem.* **2020,** *12*, 56-62.
11. Neese, F. The ORCA program system. *WIREs Comput. Mol. Sci.* **2012**, *2*, 73–78.
12. Becke, A. D. Density - functional thermochemistry. III. The role of exact exchange. *J. Chem. Phys*. **1993**, *98*, 5648–5652.
13. Lee, C., Yang, W. & Parr, R. G. Development of the Colle-Salvetti Correlation-energy Formula into a Functional of The Electron Density. *Phys. Rev. B* **1988**, *37*, 785–789.
14. Grimme, S., Antony, J., Ehrlich, S. & Krieg, H. A Consistent and Accurate ab Initio Parametrization of Density Functional Dispersion Correction (DFT-D) for The 94 Elements H–Pu. *J. Chem. Phys.* **2010**, *132*, 154104.

15. Grimme, S., Ehrlich, S. & Goerigk, L. Effect of the Damping Function in Dispersion Corrected Density Functional Theory. *J. Comput. Chem.* **2011**, *32*, 1456–1465.
16. Weigend, F. Accurate Coulomb-fitting Basis Sets for H to Rn. *Phys. Chem. Chem. Phys.* **2006**, *8*, 1057–1065.
17. Weigend, F. & Ahlrichs, R. Balanced Basis Sets of Split Valence, Triple Zeta Valence and Quadruple Zeta Valence Quality for H to Rn: Design and Assessment of Accuracy, *Phys. Chem. Chem. Phys.* **2005**, *7*, 3297–3305.
18. Valeev, E. F. Libint: A Library for The Evaluation of Molecular Integrals of Many-body Operators over Gaussian Functions, *http://libint.valeyev.net/*
19. Barone, V. & Cossi, M. Quantum Calculation of Molecular Energies and Energy Gradients in Solution by A Conductor Solvent Model, *J. Phys. Chem. A* **1998**, *102*, 1995–2001.
20. Hanwell, M. D. *et al.* Avogadro: An Advanced Semantic Chemical Editor, Visualization, and Analysis Platform. *J. Cheminf.* **2012**, *4*, 17
